# Supplementary material for: RNase III-mediated processing of a trans-acting bacterial sRNA and its cis-encoded antagonist
Source: eLife. 2021 Nov 29;10:e69064. doi: 10.7554/eLife.69064 (PMC8687705; doi:10.7554/eLife.69064)
Supplement: Figure 1—source data 1. [file elife-69064-fig1-data1.zip › Source data - Figure 1/Source data - Figure 1.docx]

**Source data for Figure 1**

**Panel B**

**
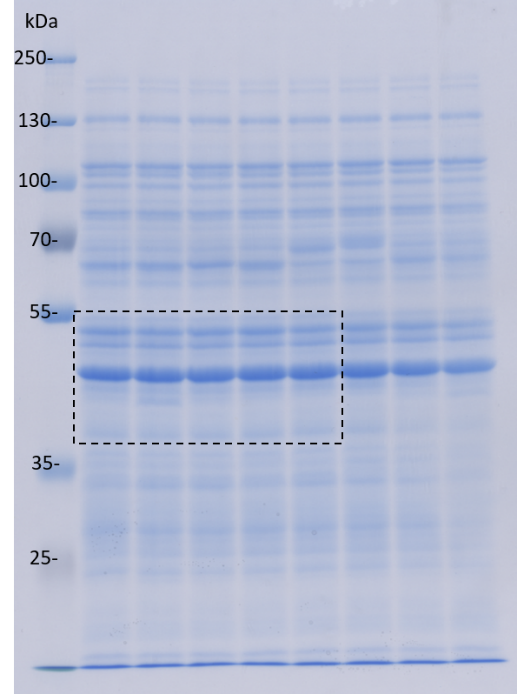
**

NB76

**
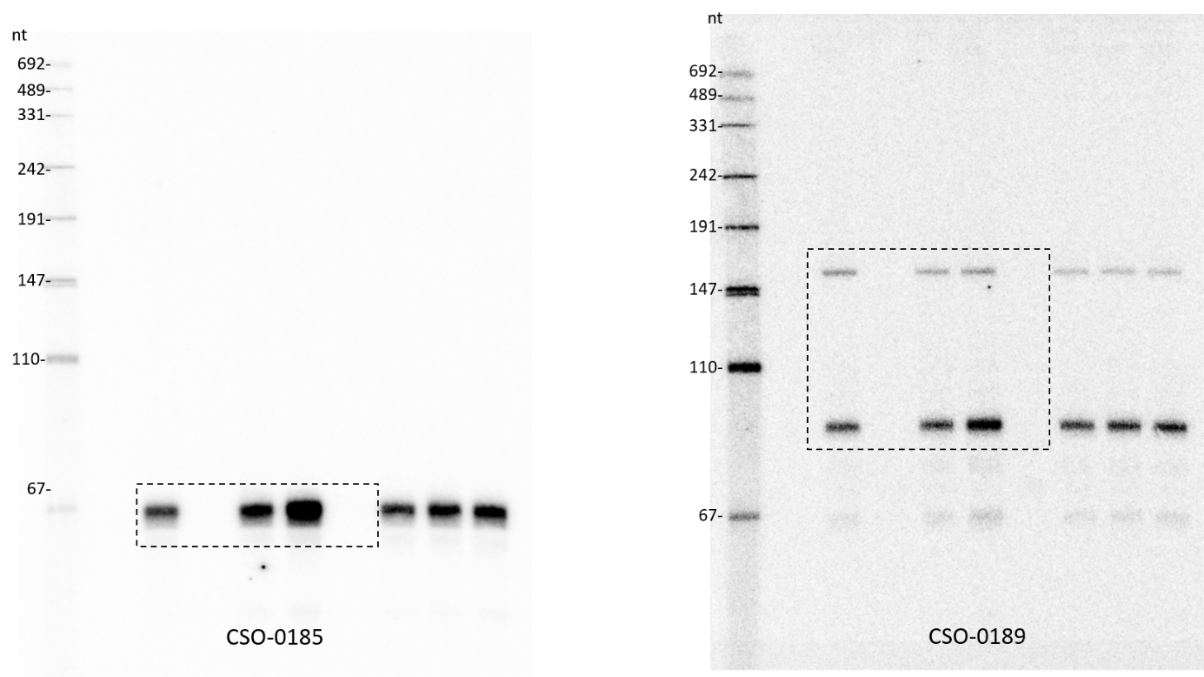
**

**
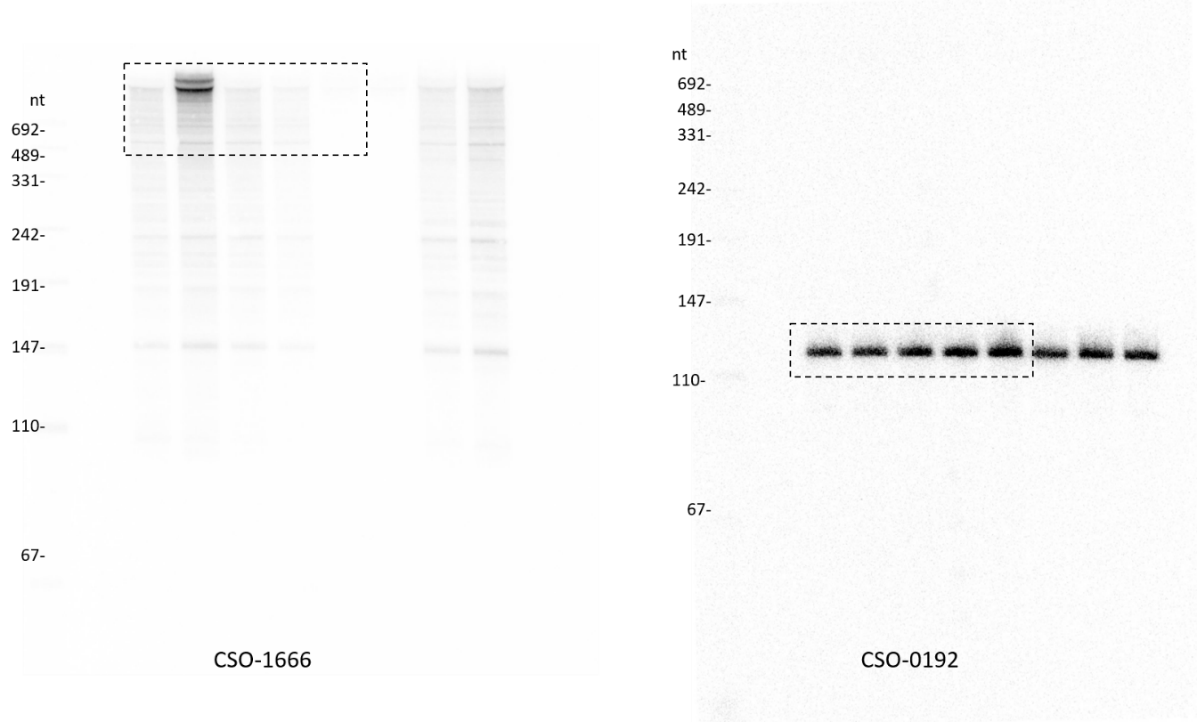
**

Northern blot quantification raw values

|  | **Intensity-Bkg [%]** | | |
| --- | --- | --- | --- |
|  | **CJnc190** | **CJnc180** | ***ptmG* mRNA** |
|  | **CSO-0185** | **CSO-0189** | **CSO-1666** |
| **WT** | 21.73679071 | 26.95934 | 8.078106255 |
| **Δ180/190** | 0.283318268 | 0.510015 | 80.18068278 |
| **C-180/190** | 27.44873468 | 22.37158 | 7.241194902 |
| **OE-180/190** | 50.23157047 | 49.70204 | 3.303095878 |
| **Δ180/190 Δ*ptmG*** | 0.299585864 | 0.457017 | 1.196920184 |

**Panel C**

**RNA sequences used for IntaRNA input**

**>CJnc180_C.jejuni_NCTC11168**

agcUUaaagagaUUUUccaaacUcUaaaagagagUUaggcUacgaaUaaaaagggggagggaaaUagccUaacccaaacgagaUcUUa

**>CJnc190_C.jejuni_NCTC11168**

AGAGAUCUUUUAAGAUCUCGUUUGGGUUAGGCUAUUUCCCUCCCCCUUUUUAUUCGUAGCCUAACUCUCU

**>Cj1324_C.jejuni_NCTC11168**

AUUUUUAUUAAAUUGAAGGGGUGGGGAAUGAUUUAUUGUGAUCACUGCGUGAUGCCAAAUACUAGACCUGGUAUUAAUUUUACAAAAGAUAAAGAAGGUAAAAAUAUCUGUUCAGCUUGCAUCAAUCAUAAAAAUAAAGAAAAUAUUGAUUAUAAAGCAAGGUUUAAAGAGCUUGAAGUCUUAUGCGAUAAAUACCGCAGAAUGAAUGGAAAAUUUGAAUAUGAUUGUGCUAUUGCCGUAAGUGGAGGUAAAGAUUCGCAUUUUCAAGUGCAUAUCAUGAAAGAAAAACUUGGAAUGAAUCCUAUUCUUUUUAGCGUUGAAGAUAAUUUCACCAUGACUGAAGCAGGCAAGAAAAAUCUUAAAAAUUUAAGUGAAACUUUUGGUUGUCAUAUCAUUAGCUUAAAGCCUGAUAUCAAAACUCAAAAAAAAGUUAUGCUAAAAACCUUUGAAAAAUAUGGAAAACCUACUUGGUUUAUUGAUAGACUAAUUUACAGCUAUCCUUUUGCUAUGGCUUUAAAAUUUAAUACACCUUUAUUGGUUUAUGGAGAAAAUGUUAGUUAUGAAUAUGGAGGUAGUGAUACCGAAGAAACUCCUAGCGCUAAAGAAAUAUUUUUAAAUGGUGUAGCUAGUGAUUUAAAUAUAAAUGAAUUUAUAGAUGAUGAAAUCAAAGAAGAAAAUUUGCAACUCUUUUUCAAUCCAAACAAAGAUAAACUCGAUAAACUUAACCCUAUCUAUCUAAGUUAUUUUGUAAAAUGGAAUUCUUAUAGCAAUUAUAUUUUUGCUAAAAGUCGUGGUUUUACUGAUCUAGAAGGCGAAUGGGAUAGAACAAUGUGUGCAGAAAAUUUUGAUCAAGUUGAUAGUAUAGGCUAUAUCCUACACGCUUGGAUGAAAUACCCUAAAUUUGGUCAUGCUUGUGCGAGUGAUUACGCAGCUCGUUUCGUGCGUUAUGGACUUUUAAGCAGAAAAGAAGCUAUUGAACUUGUGCAAAAAAGAGAUCAUAAGCUUGACAAUAAAUGCGUUGAAGAUUUUUGCAAUUUCAUAGGUAUUAGUAAAACUACAUUUUGGAAAAUAGUAGAAAAACAUUACAAUAUGGAUUUAUUUUAUAAAAAUGAUUUUGGAGAAUUUAAAUUAAAAAACAAAUUACAAUAA

**Panel D**

|  |  | **Intensity-Bkg [%]** | | | |
| --- | --- | --- | --- | --- | --- |
|  |  | **anti-FLAG (PtmG-3xFLAG)** | | | |
|  |  | **R1** | **R2** | **R4** | **R5** |
| **PtmG-3xFLAG** | **WT** | 6.79651 | 7.83947 | 6.12076 | 6.92767 |
| **PtmG-3xFLAG** | **Δ180/90** | 13.27588 | 9.67691 | 8.69753 | 8.47448 |
| **PtmG-3xFLAG** | **C-180/190** | 5.09683 | 5.70894 | 7.41613 | 7.52065 |
| **PtmG-3xFLAG** | **C-180(Proc)** | 17.65217 | 13.45725 | 9.15564 | 9.64460 |
| **PtmG-3xFLAG** | **C-190(Proc)** | 3.00541 | 5.90075 | 7.04093 | 6.43383 |
