# Supplementary figures and images for: RNase III-mediated processing of a trans-acting bacterial sRNA and its cis-encoded antagonist (part 1 of 2)
Source: eLife. 2021 Nov 29;10:e69064. doi: 10.7554/eLife.69064 (PMC8687705; doi:10.7554/eLife.69064)

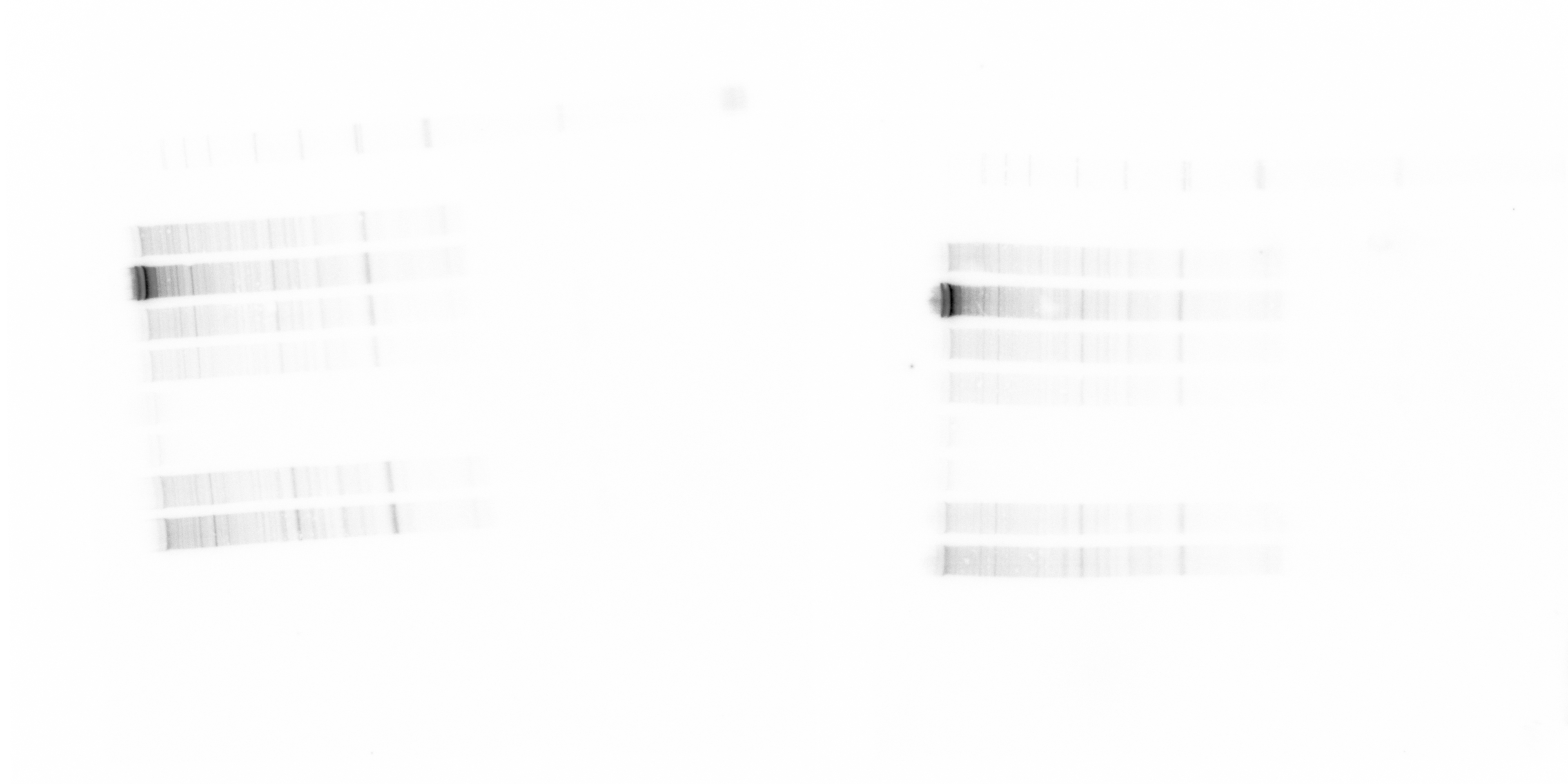

Supplement: Figure 1—source data 1. [file elife-69064-fig1-data1.zip › Source data - Figure 1/Figure 1B - 05062016_NB75-76_CSO-1666_6d-[Phosphor].tif]

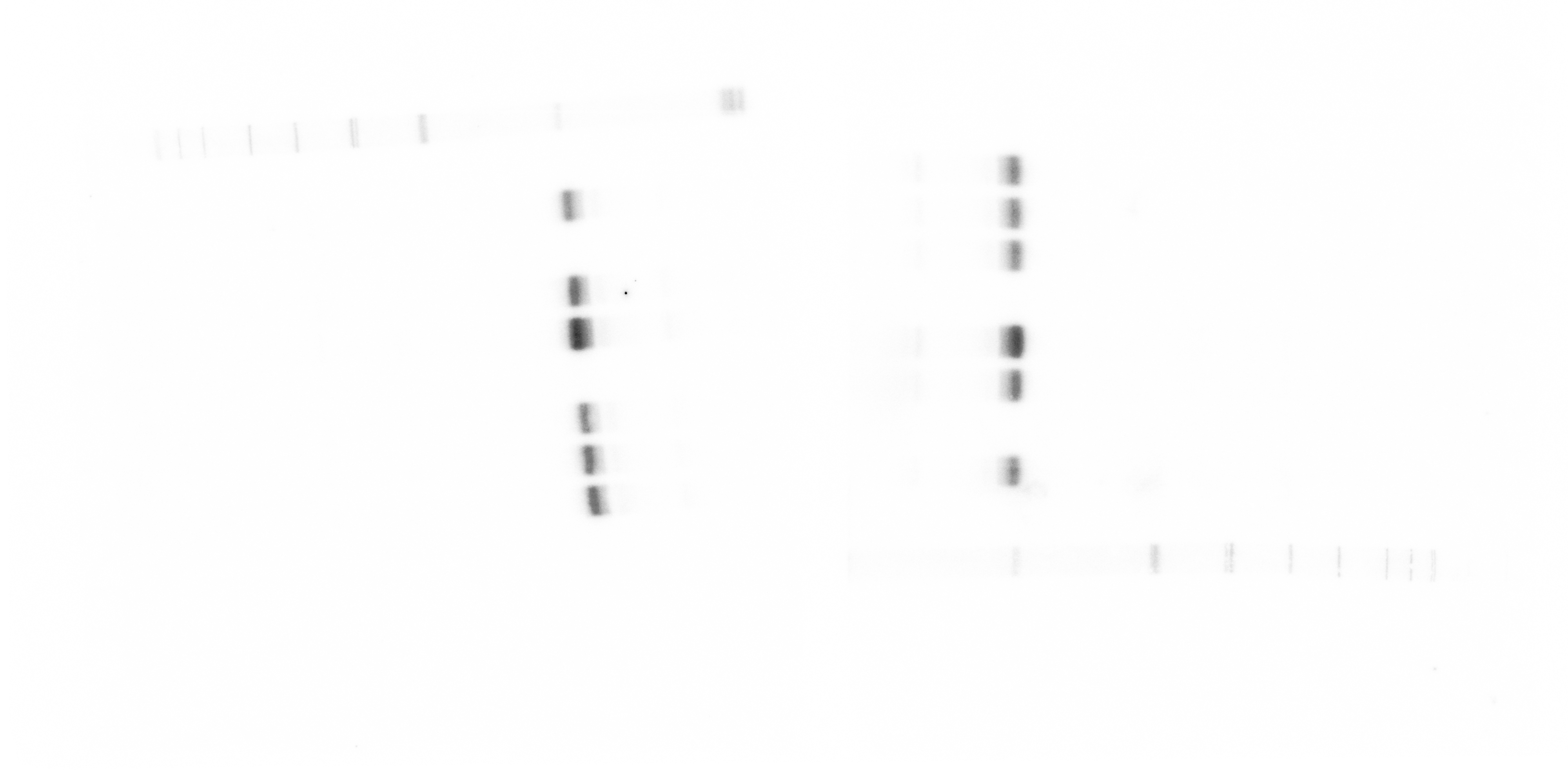

Supplement: Figure 1—source data 1. [file elife-69064-fig1-data1.zip › Source data - Figure 1/Figure 1B - 20160522_NB75_76_CSO-0185_4d-[Phosphor].tif]

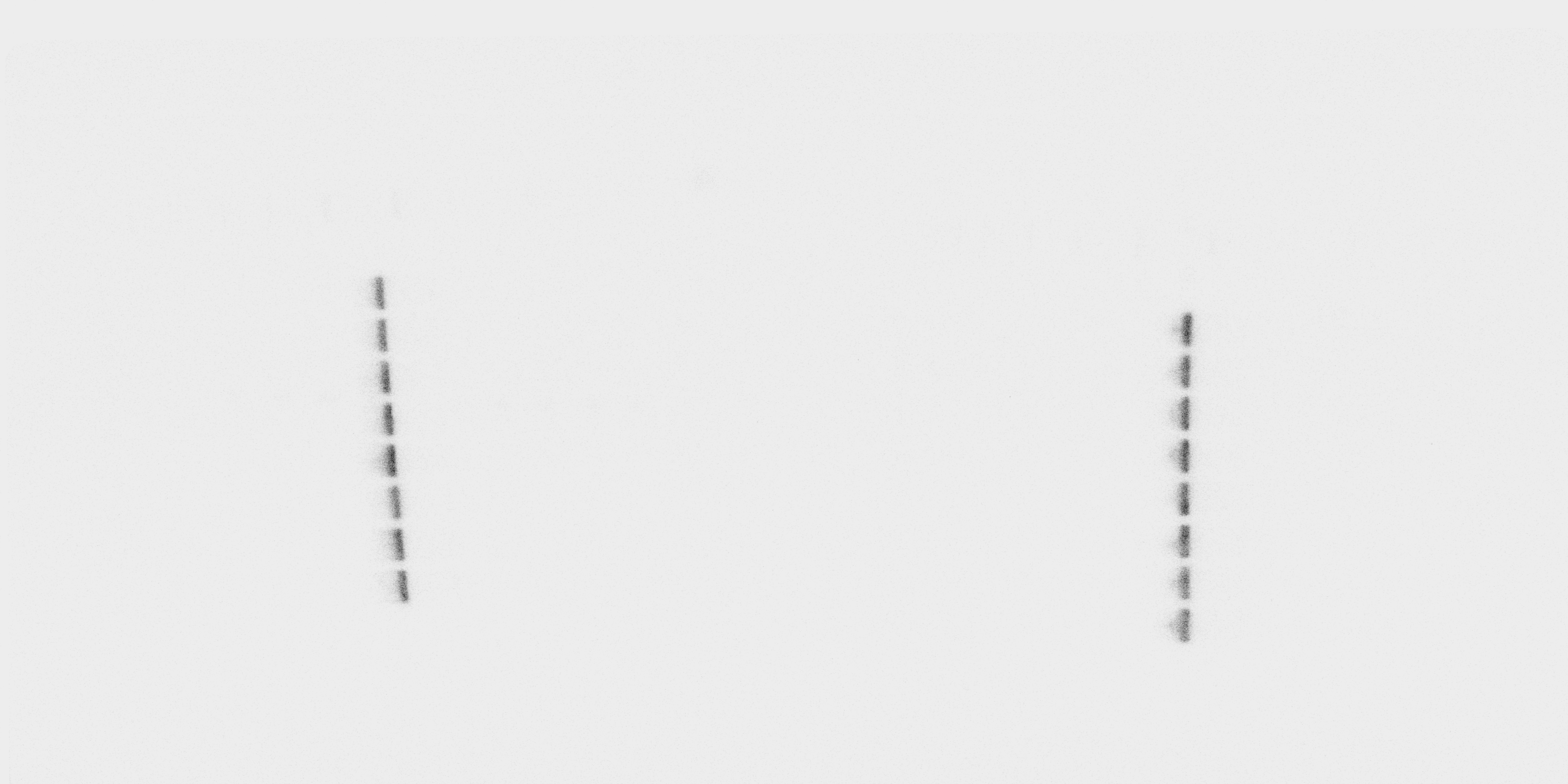

Supplement: Figure 1—source data 1. [file elife-69064-fig1-data1.zip › Source data - Figure 1/Figure 1B - 20160606_NB75_76_CSO-0192_5h-[Phosphor].tif]

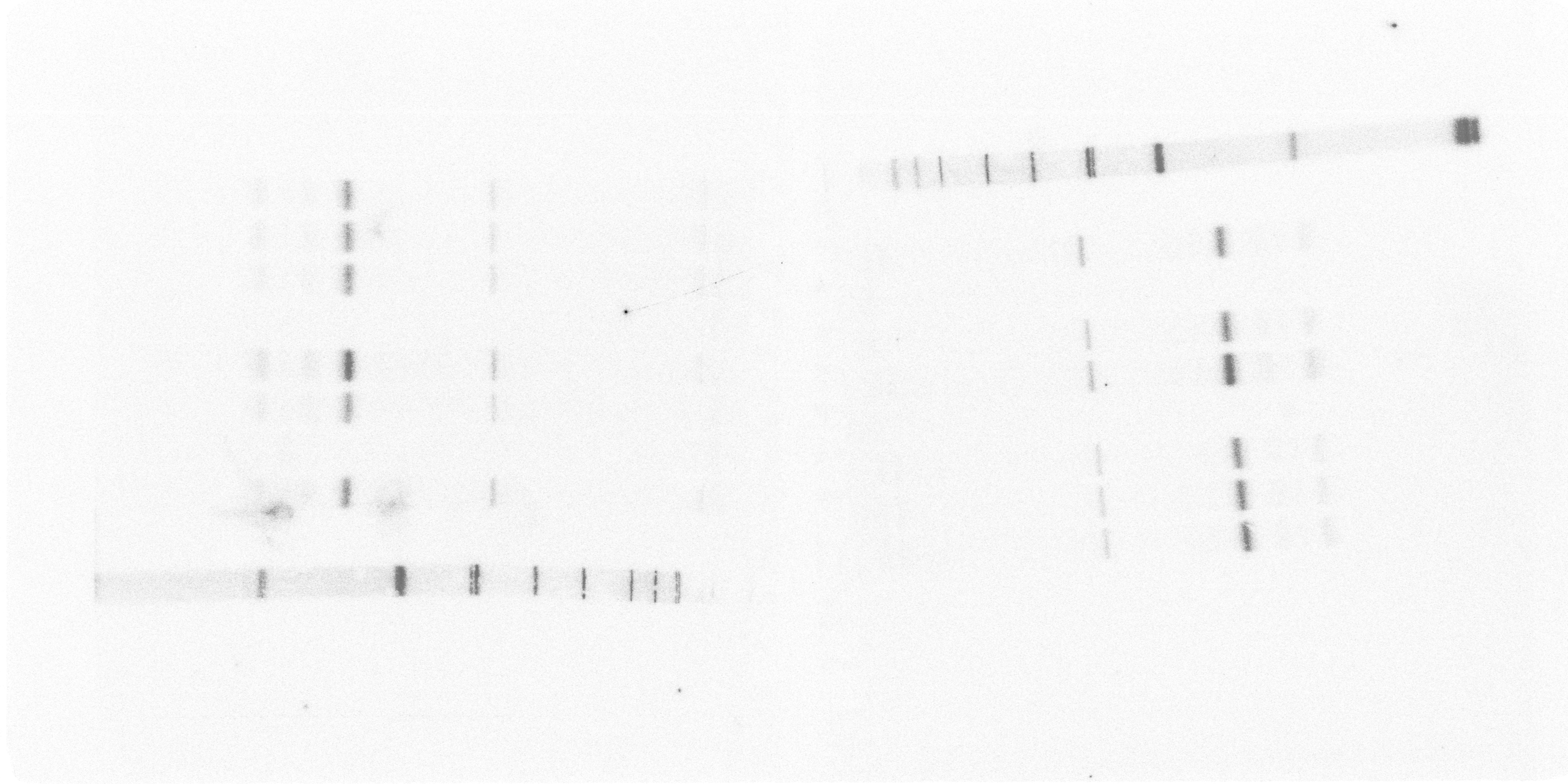

Supplement: Figure 1—source data 1. [file elife-69064-fig1-data1.zip › Source data - Figure 1/Figure 1B - NB75_76_CSO-0189_5d-[Phosphor].tif]

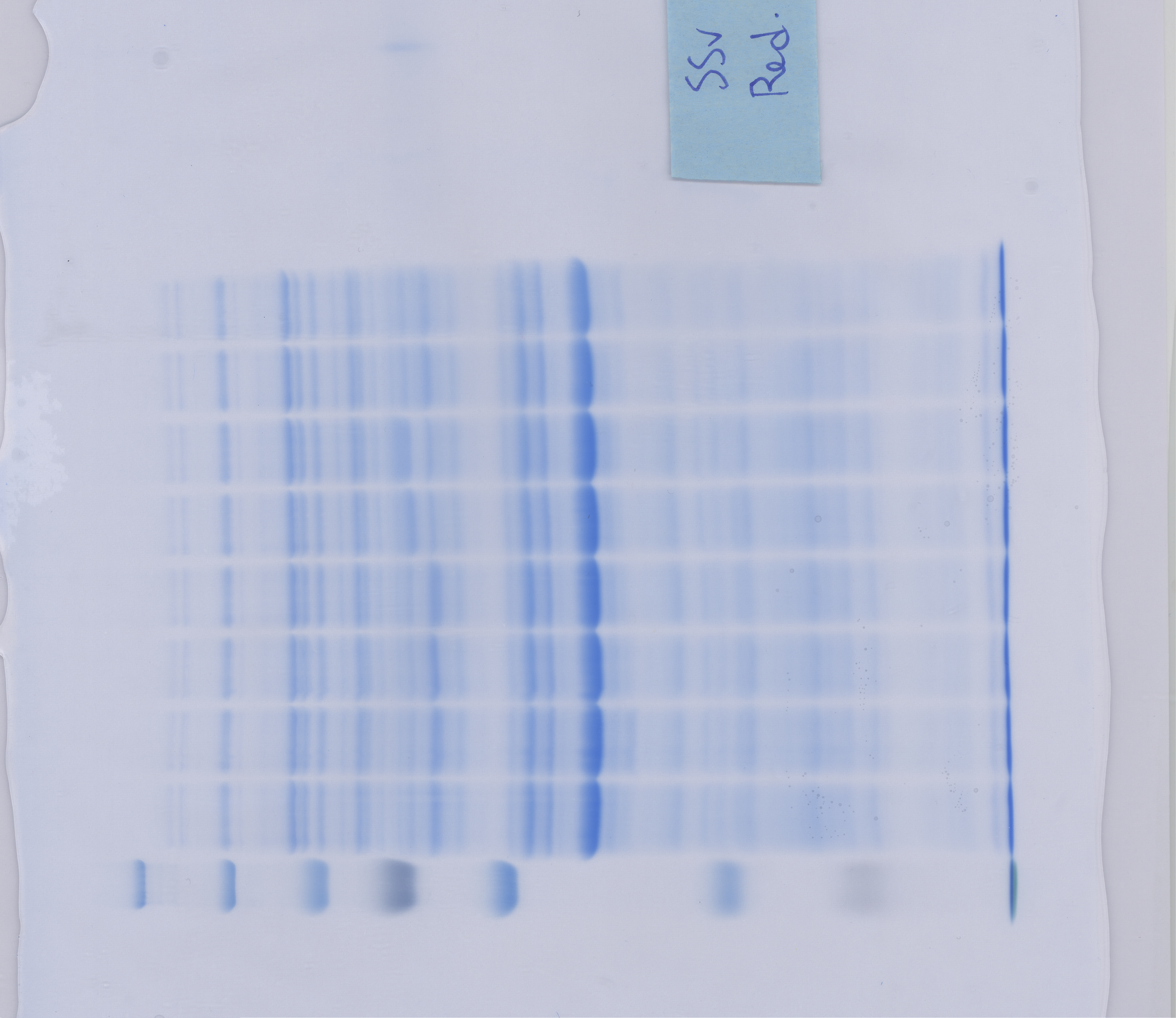

Supplement: Figure 1—source data 1. [file elife-69064-fig1-data1.zip › Source data - Figure 1/Figure 1B - SDS-PAGE.tif]

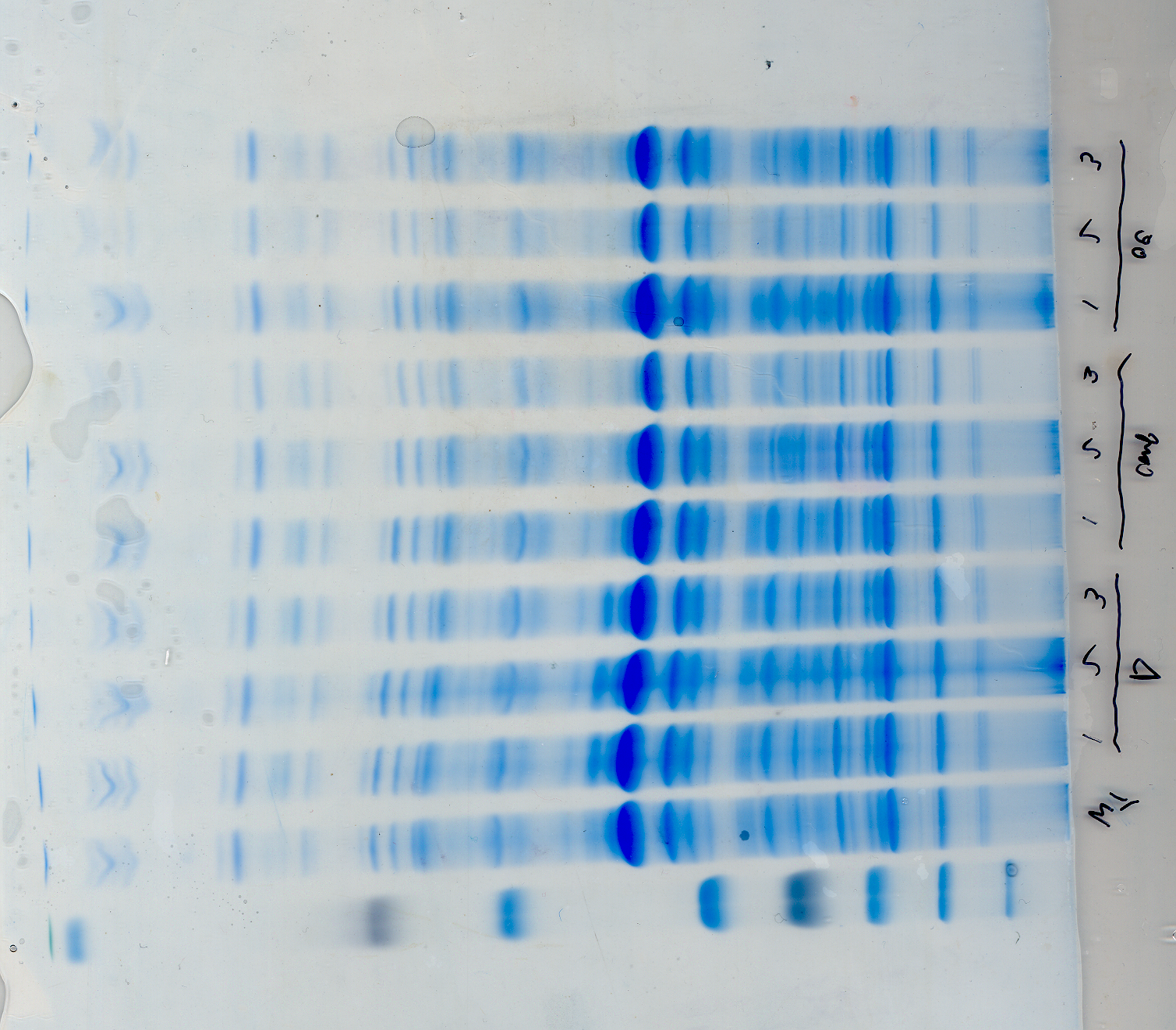

Supplement: Figure 1—figure supplement 1—source data 1. [file elife-69064-fig1-figsupp1-data1.zip › Source data - Figure 1 - figure supplement 1/Figure 1 - figure supplement 1 SDS-PAGE.tif]

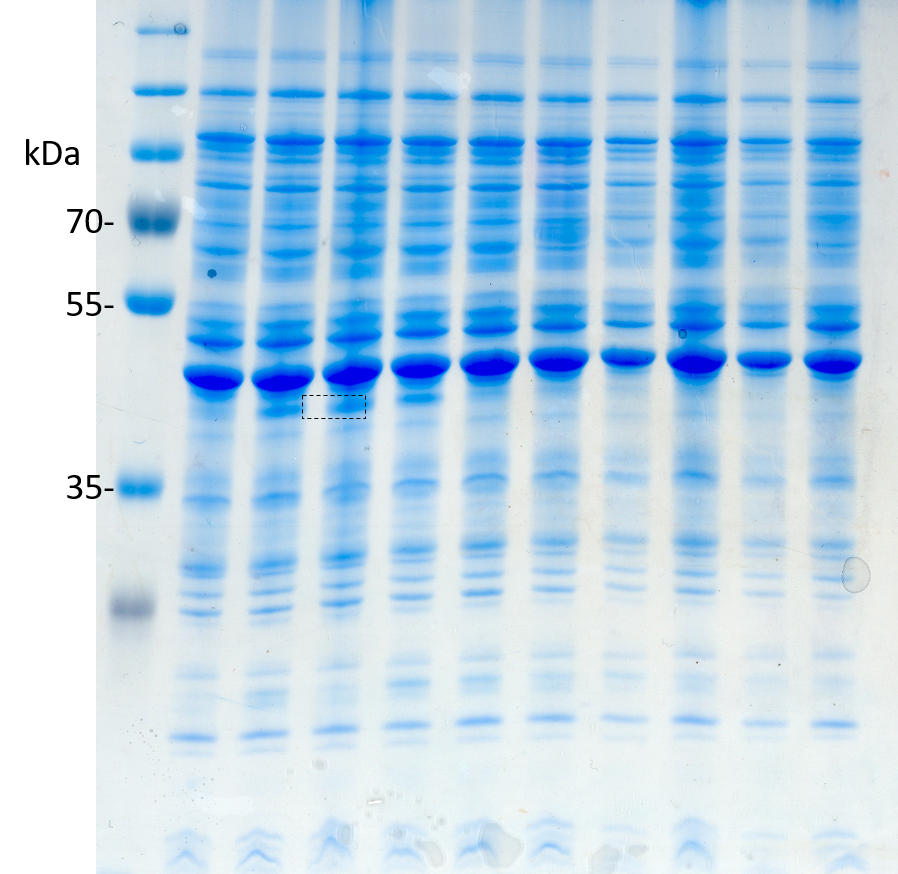

Supplement: Figure 1—figure supplement 1—source data 1. [file elife-69064-fig1-figsupp1-data1.zip › Source data - Figure 1 - figure supplement 1/Source data - Figure 1 - Figure supplement 1.docx]

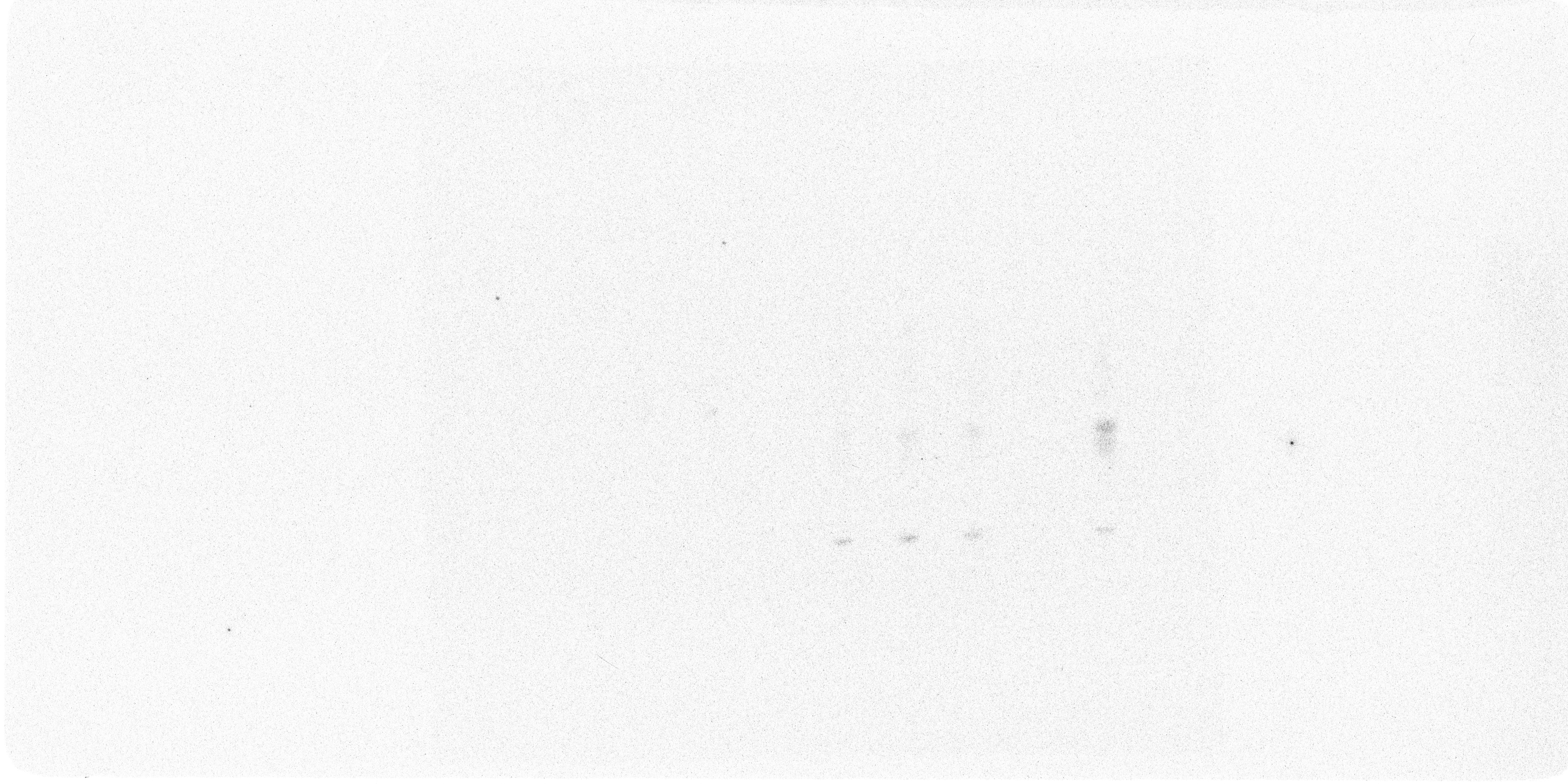

Supplement: Figure 1—figure supplement 2—source data 1. [file elife-69064-fig1-figsupp2-data1.zip › Source data - Figure 1 - figure supplement 2/Fig 1 - supp 2A - 24.9.2018_NB118_CSO-0189_7d-[Phosphor].tif]

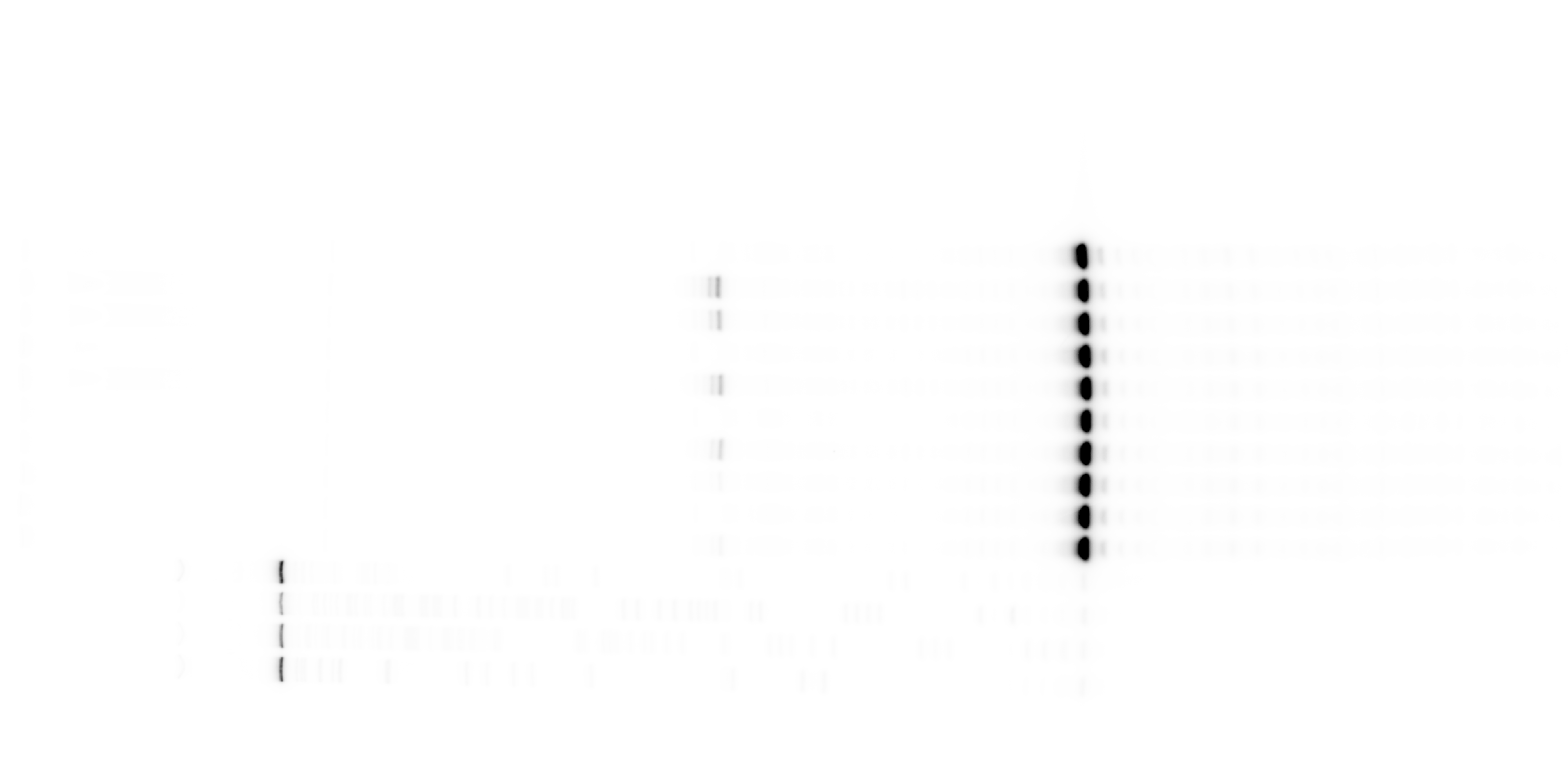

Supplement: Figure 1—figure supplement 2—source data 1. [file elife-69064-fig1-figsupp2-data1.zip › Source data - Figure 1 - figure supplement 2/Fig 1 - supp 2A - 29.10.2017_primext_CSO-0188_3d-[Phosphor].tif]

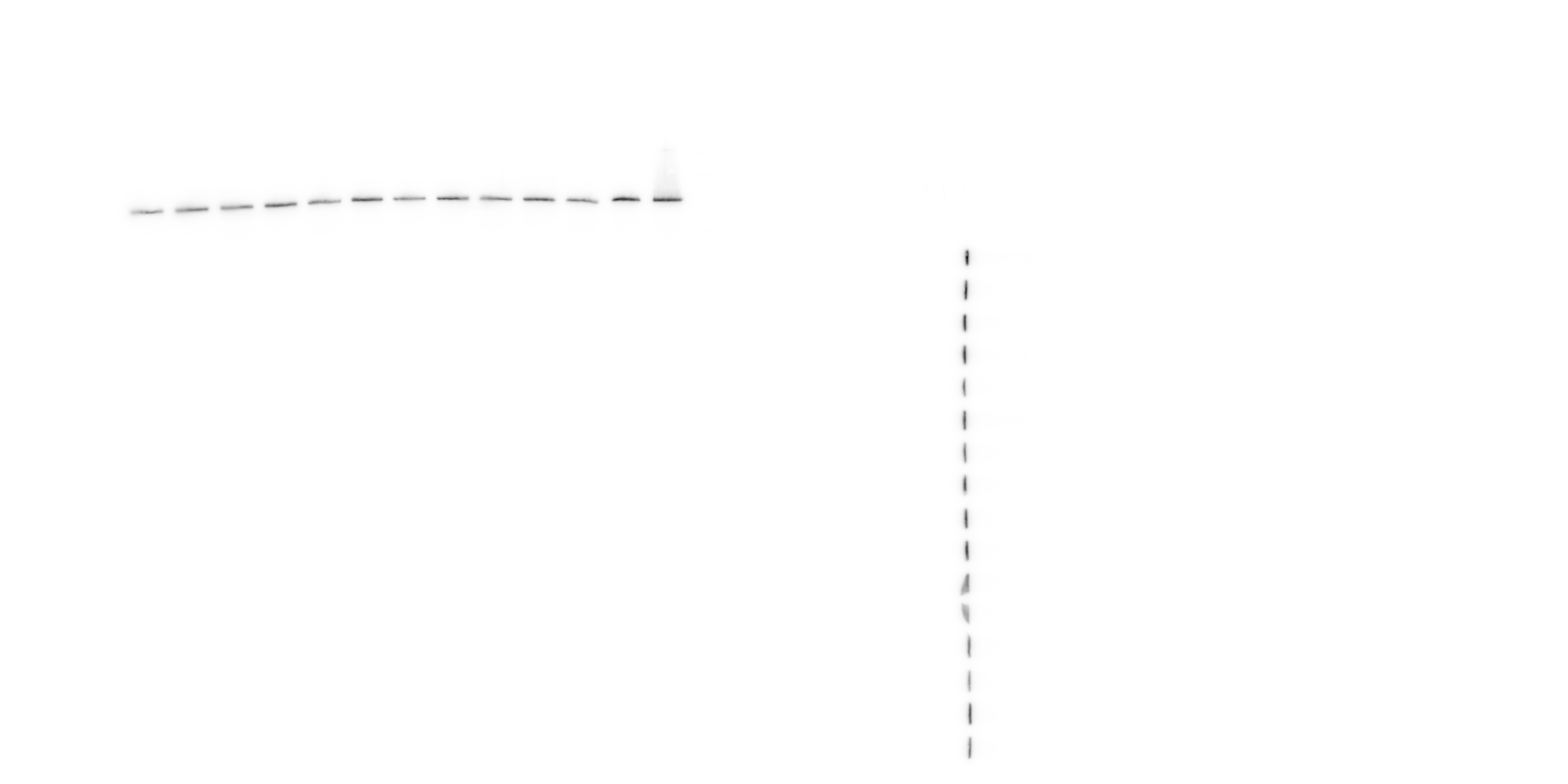

Supplement: Figure 1—figure supplement 2—source data 1. [file elife-69064-fig1-figsupp2-data1.zip › Source data - Figure 1 - figure supplement 2/Fig 1 - supp 2A - 5.11.17.NB117_118_CSO-0497_ON-[Phosphor].tif]

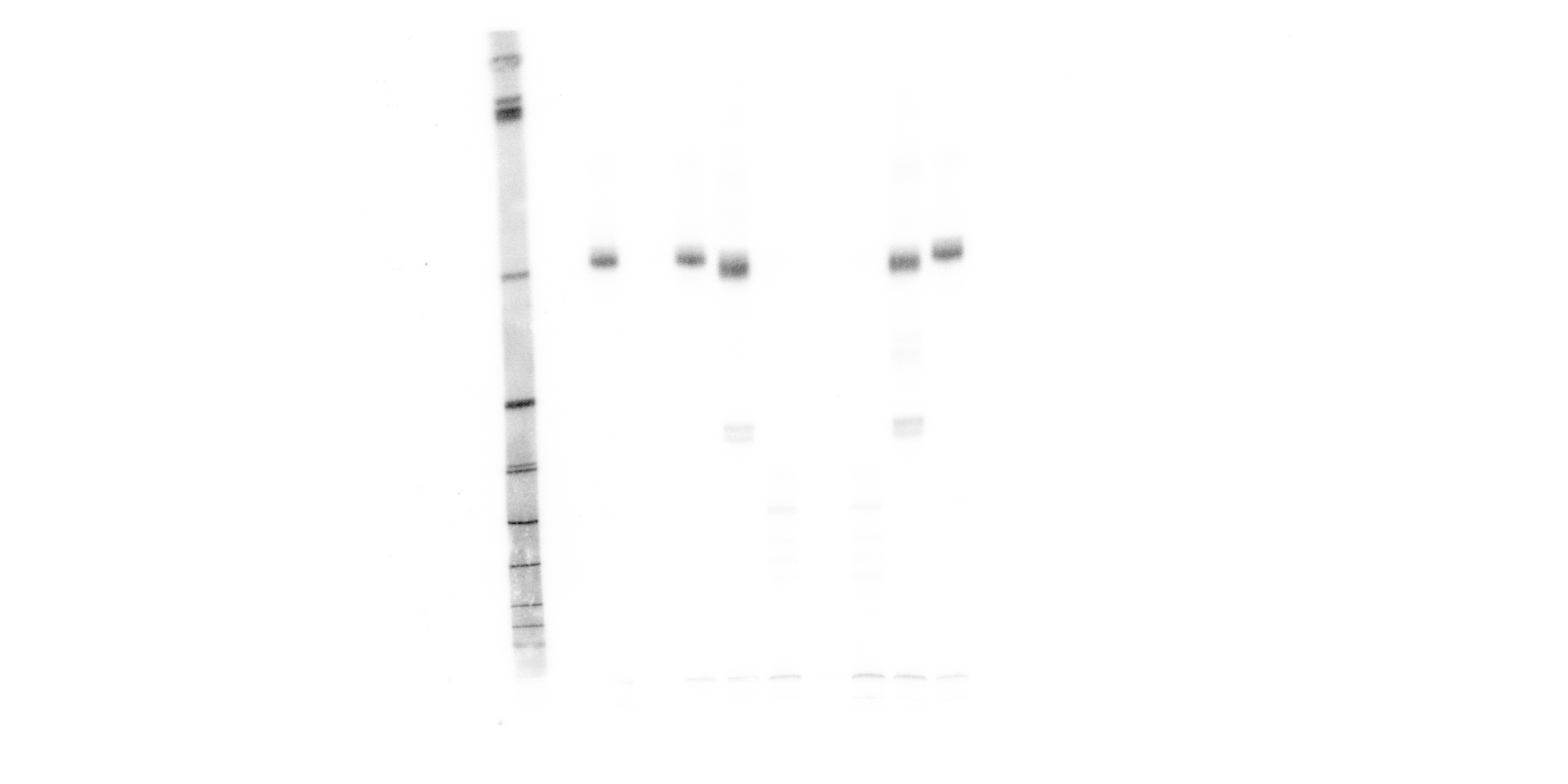

Supplement: Figure 1—figure supplement 2—source data 1. [file elife-69064-fig1-figsupp2-data1.zip › Source data - Figure 1 - figure supplement 2/Fig 1 - supp 2B - 2.7.2017_NB107_CSO-0185_4d-[Phosphor].tif]

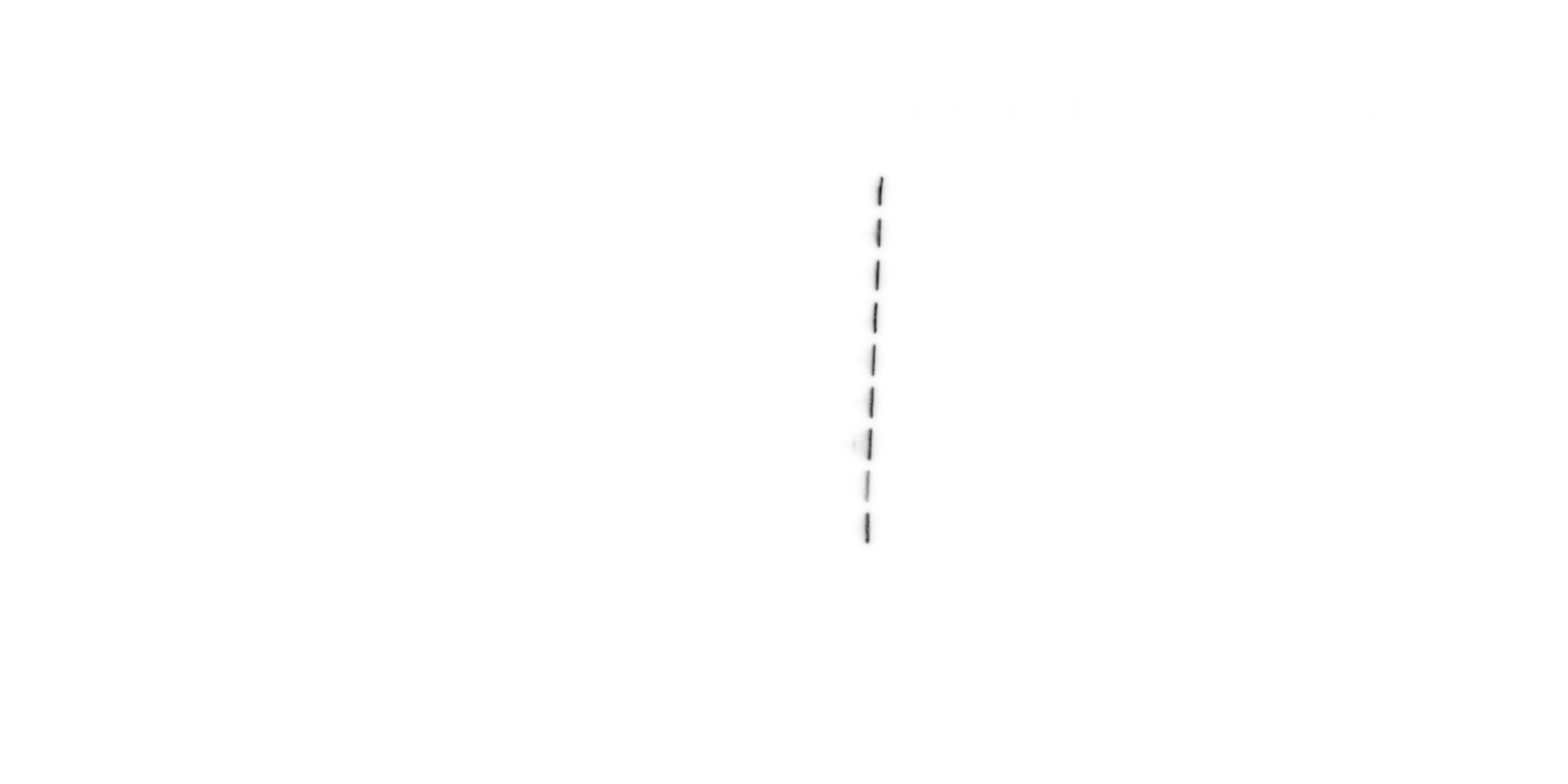

Supplement: Figure 1—figure supplement 2—source data 1. [file elife-69064-fig1-figsupp2-data1.zip › Source data - Figure 1 - figure supplement 2/Fig 1 - supp 2B - 3.7.2017_NB107_CSO-0497_5h-[Phosphor].tif]

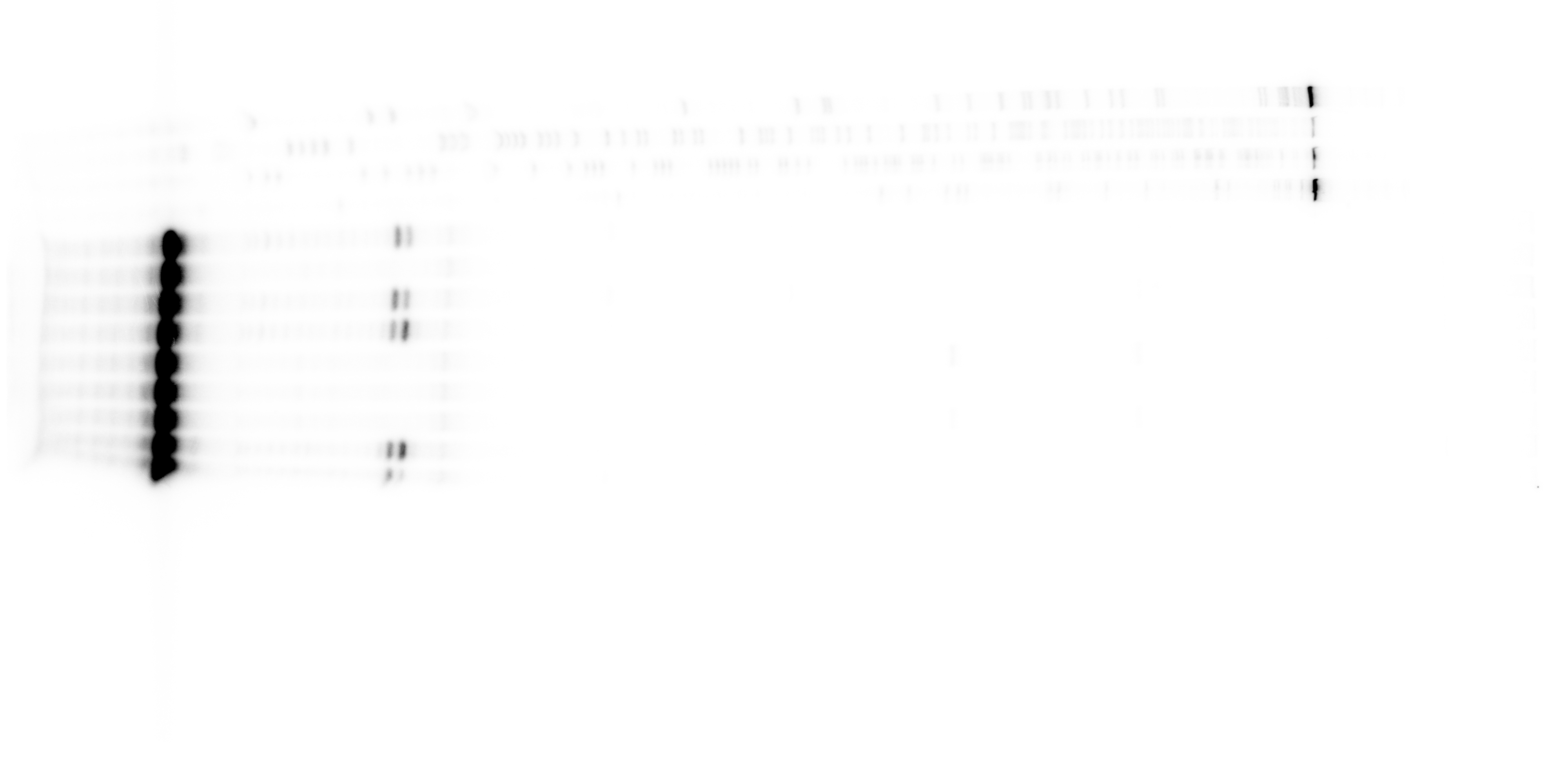

Supplement: Figure 1—figure supplement 2—source data 1. [file elife-69064-fig1-figsupp2-data1.zip › Source data - Figure 1 - figure supplement 2/Fig 1 - supp 2B - 6.7.2017_primer_ext_CSO-0185_pSSv20_3d-[Phosphor].tif]

**Source data for Figure 2 – figure supplement 2**

**Panel A**

**
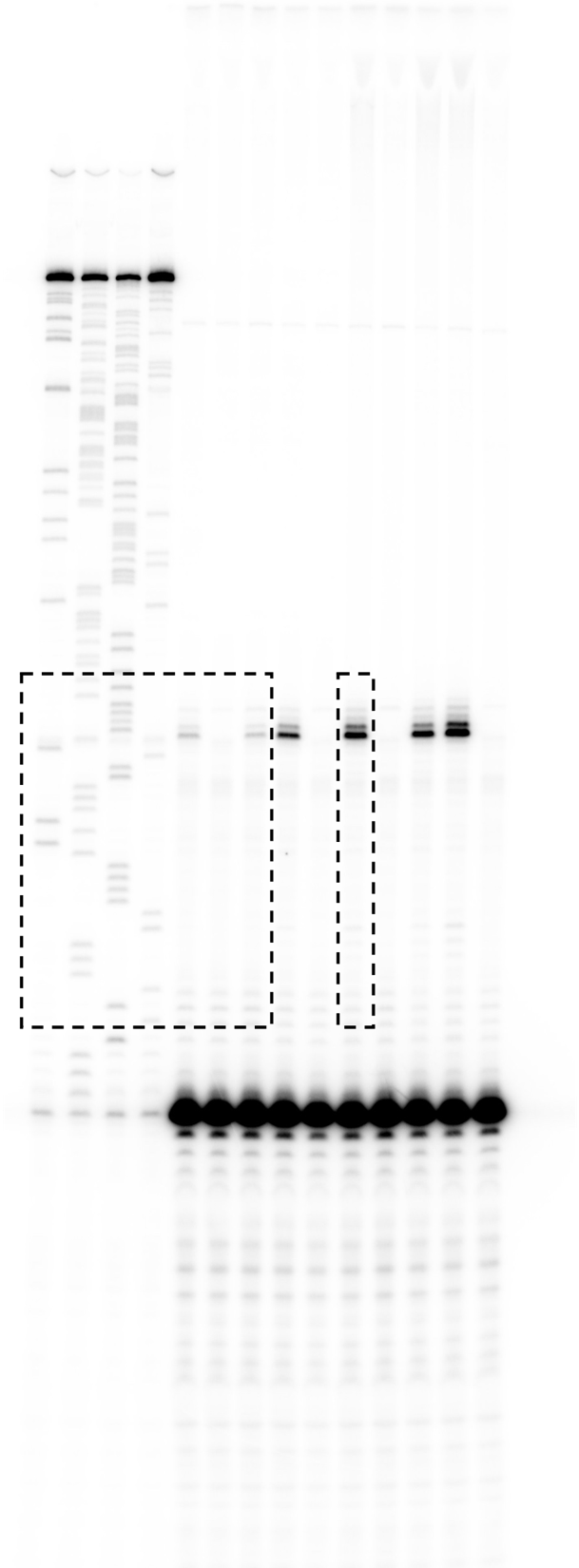
**

NB117

**
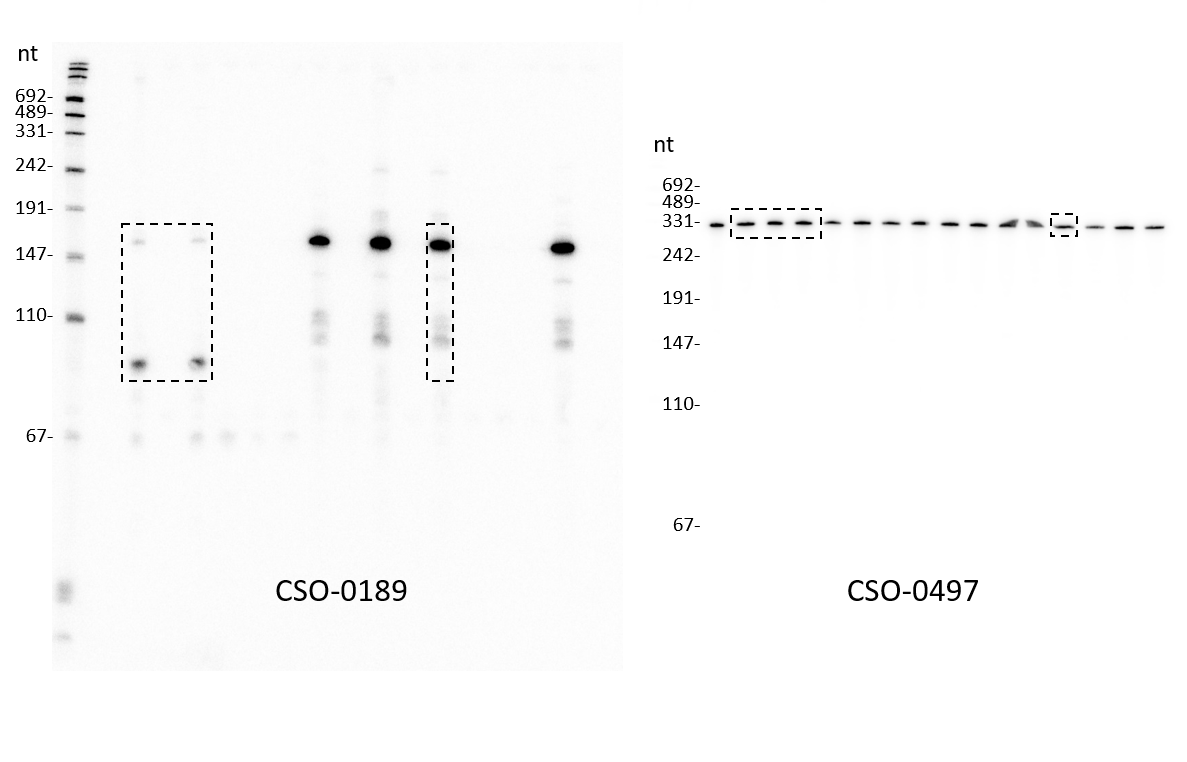
**

**Panel B**


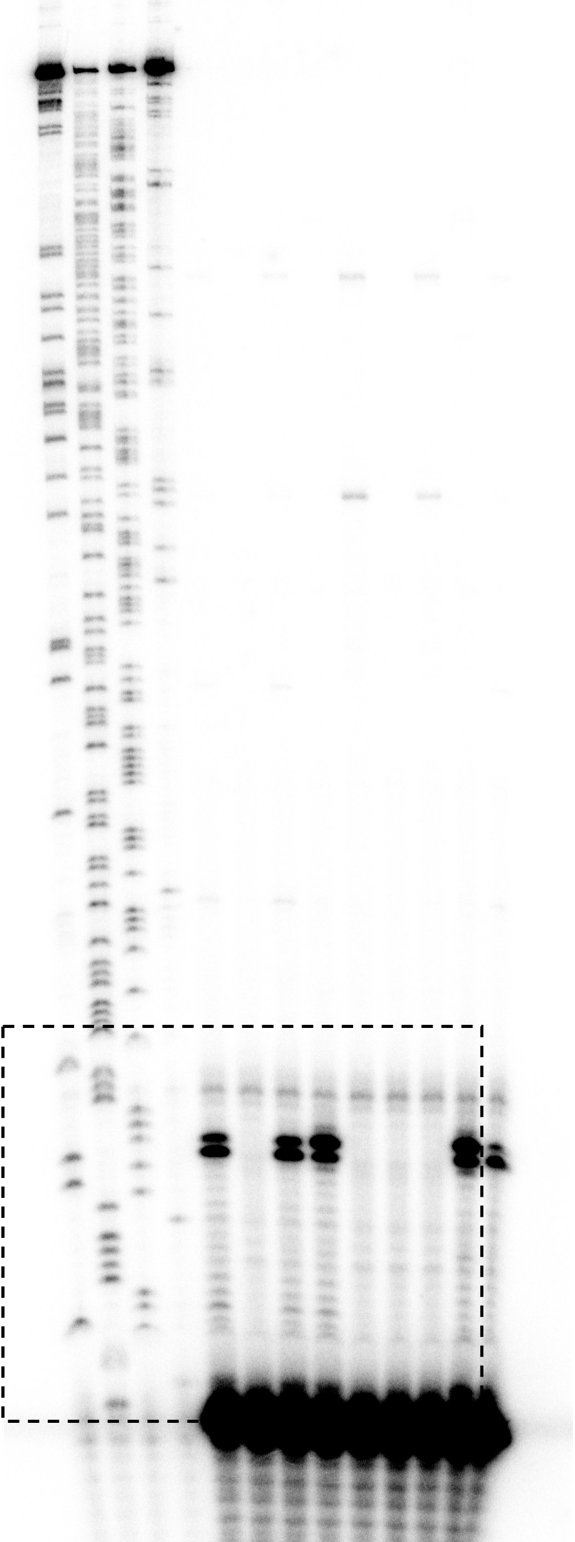


NB107

**
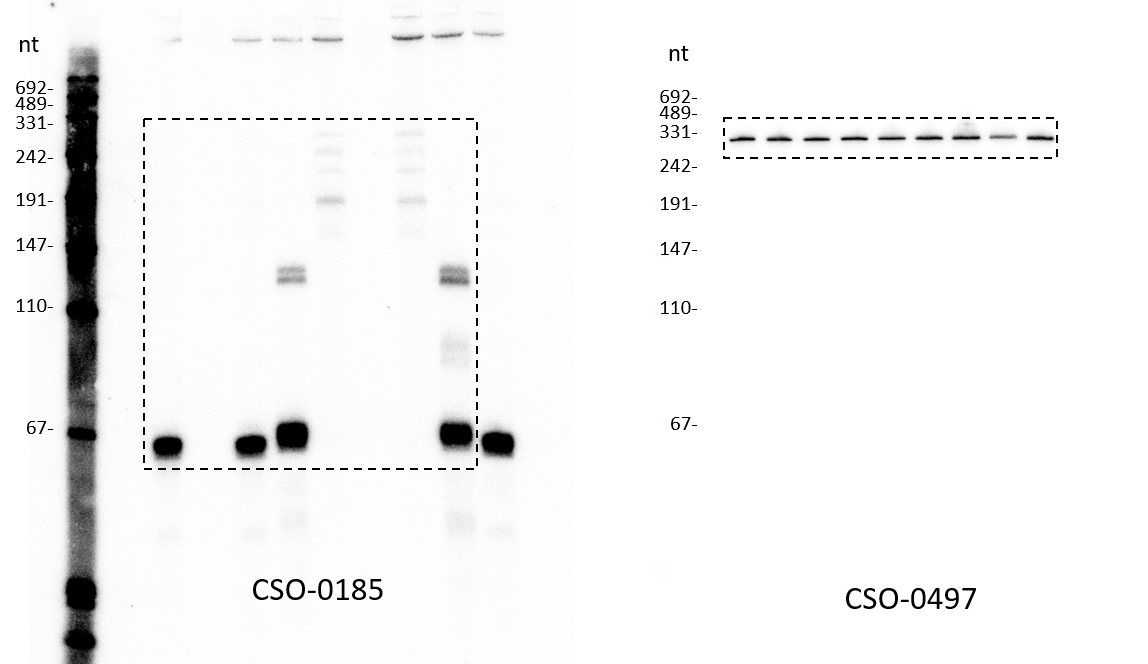
**

Supplement: Figure 1—figure supplement 2—source data 1. [file elife-69064-fig1-figsupp2-data1.zip › Source data - Figure 1 - figure supplement 2/Source data - Figure 1 - Figure supplement 2.docx]

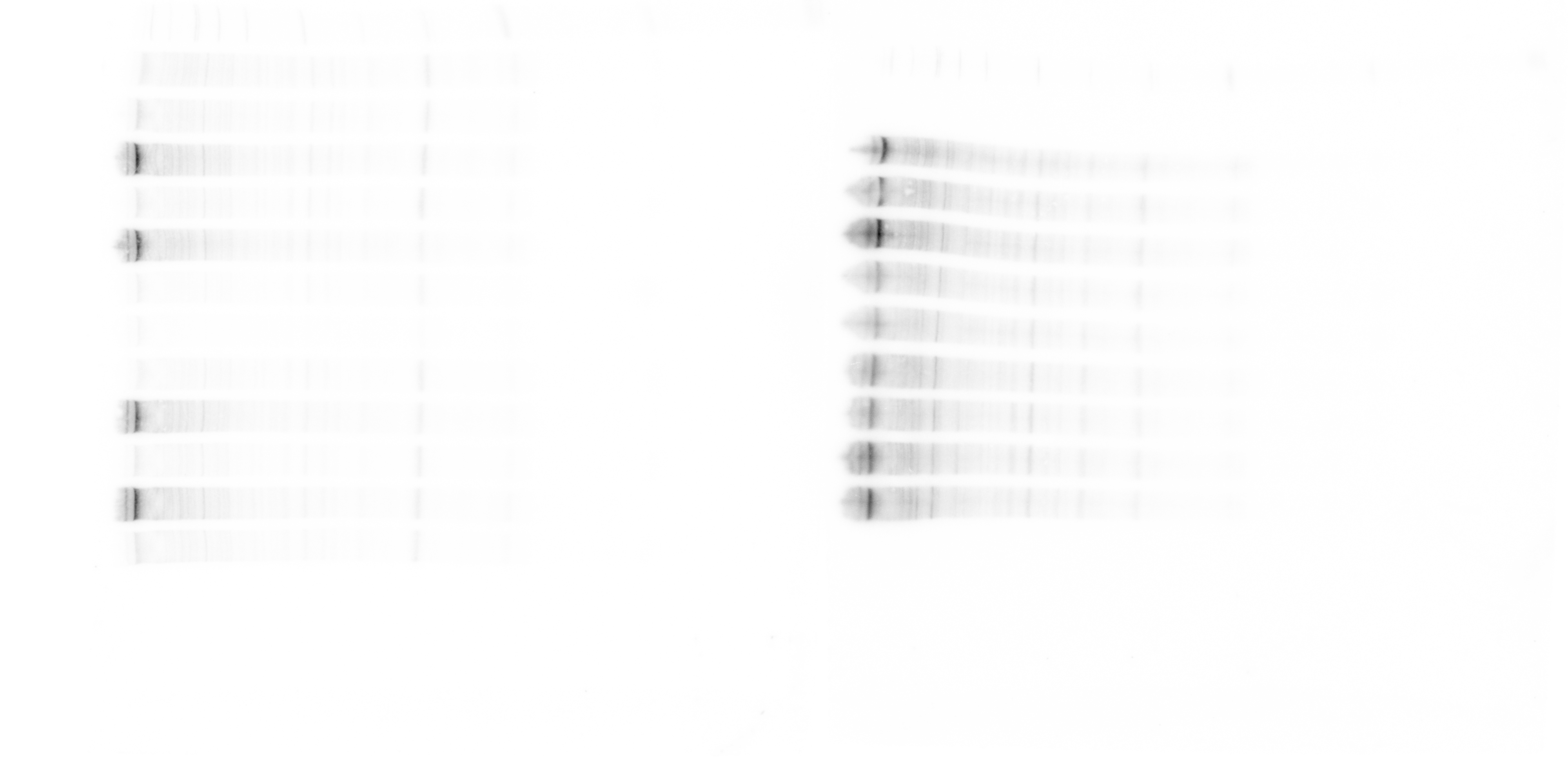

Supplement: Figure 1—figure supplement 4—source data 1. [file elife-69064-fig1-figsupp4-data1.zip › Source data - Figure 1 - figure supplement 4 - Source Data 1/Fig 1 - supp 4A - 15.3.2020_NB187_188_CSO-1666_5d-[Phosphor].tif]

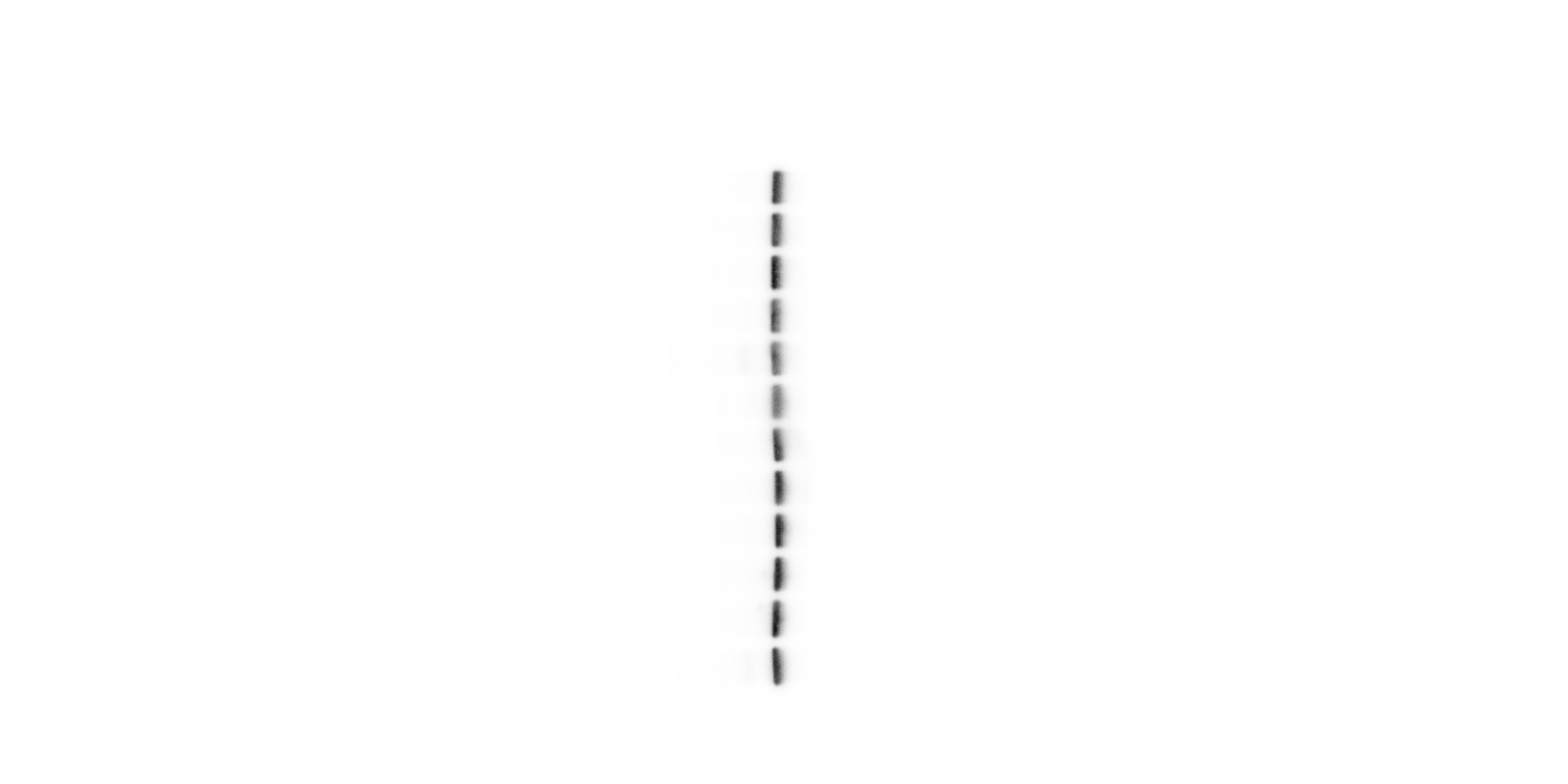

Supplement: Figure 1—figure supplement 4—source data 1. [file elife-69064-fig1-figsupp4-data1.zip › Source data - Figure 1 - figure supplement 4 - Source Data 1/Fig 1 - supp 4A - 19.3.2020_NB187_CSO-0192_2h-[Phosphor].tif]

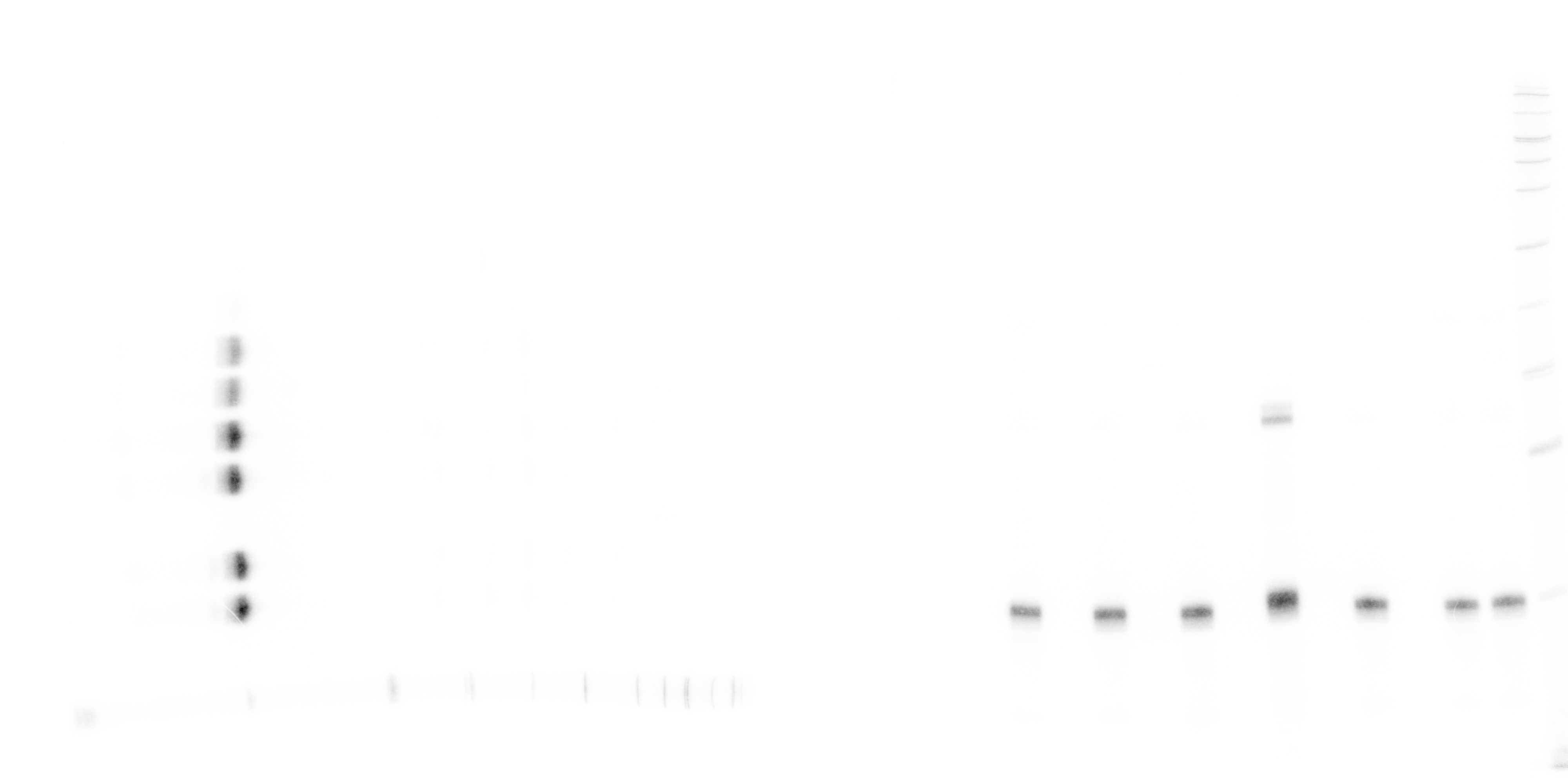

Supplement: Figure 1—figure supplement 4—source data 1. [file elife-69064-fig1-figsupp4-data1.zip › Source data - Figure 1 - figure supplement 4 - Source Data 1/Fig 1 - supp 4A - 2.3.2020_NB187_188_CSO-0185_4d-[Phosphor].tif]

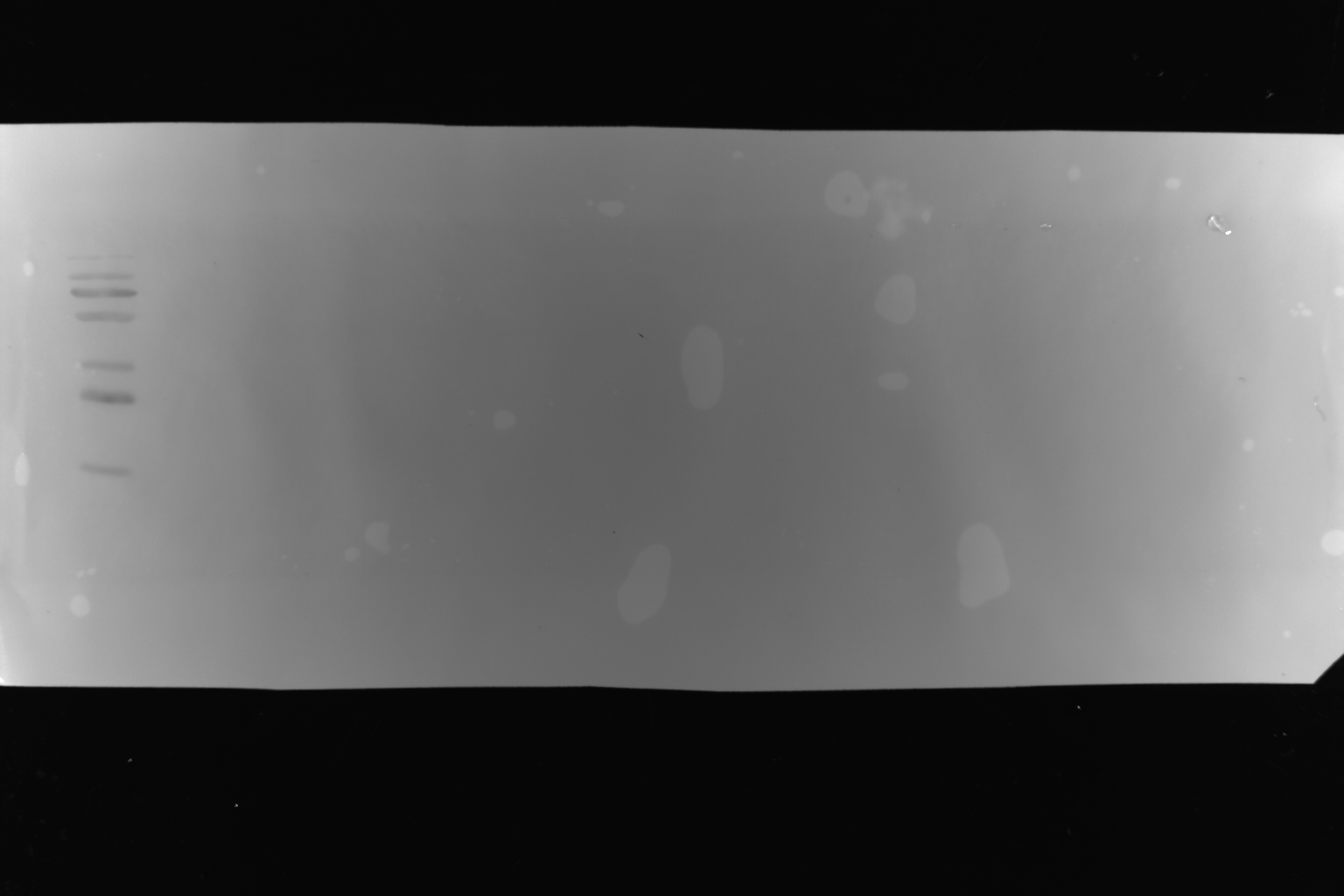

Supplement: Figure 1—figure supplement 4—source data 1. [file elife-69064-fig1-figsupp4-data1.zip › Source data - Figure 1 - figure supplement 4 - Source Data 1/Fig 1 - supp 4A - 20200218_1305 gel 1 FLAG ladder.tif]

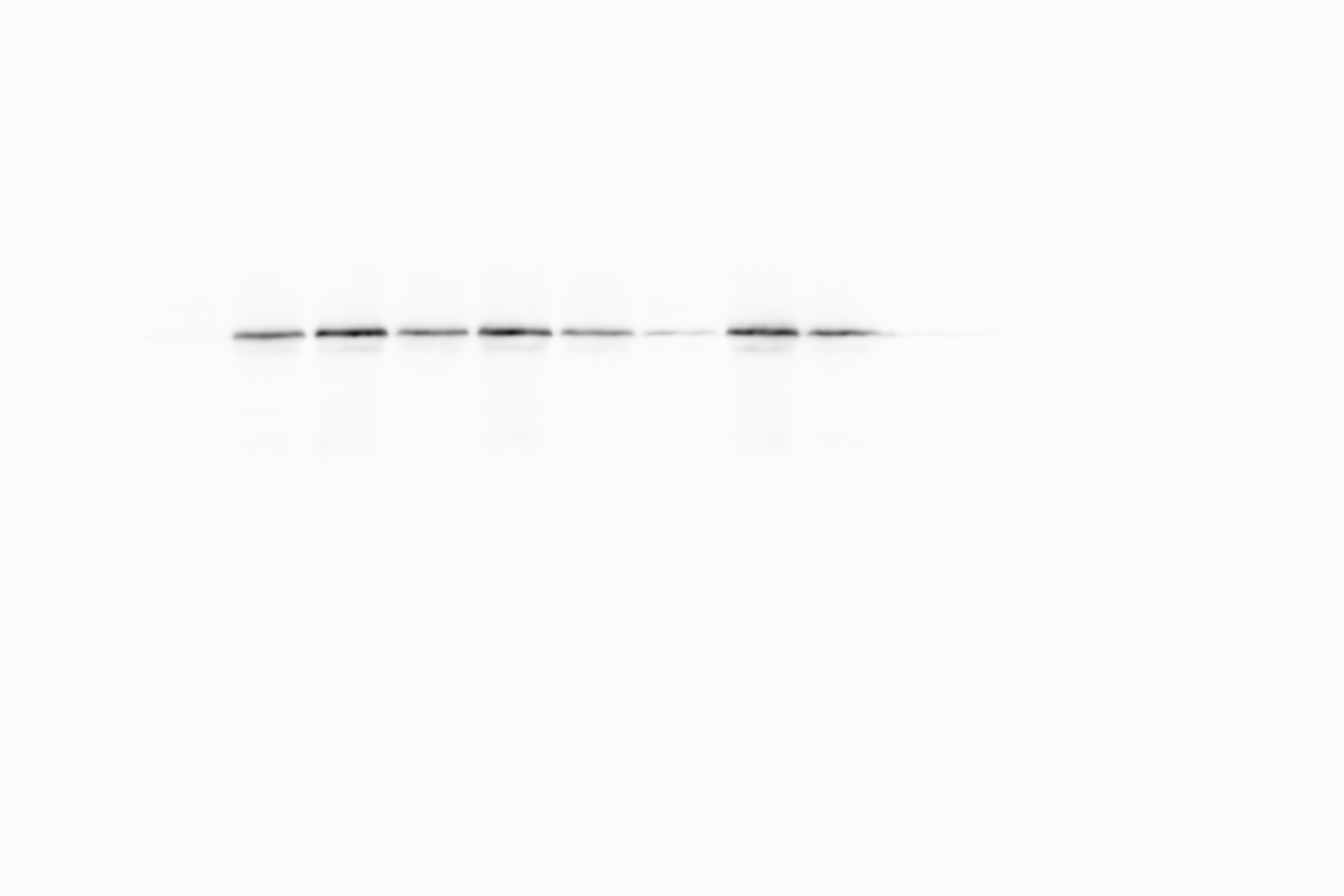

Supplement: Figure 1—figure supplement 4—source data 1. [file elife-69064-fig1-figsupp4-data1.zip › Source data - Figure 1 - figure supplement 4 - Source Data 1/Fig 1 - supp 4A - 20200218_1312 gel 1 FLAG_20.tif]

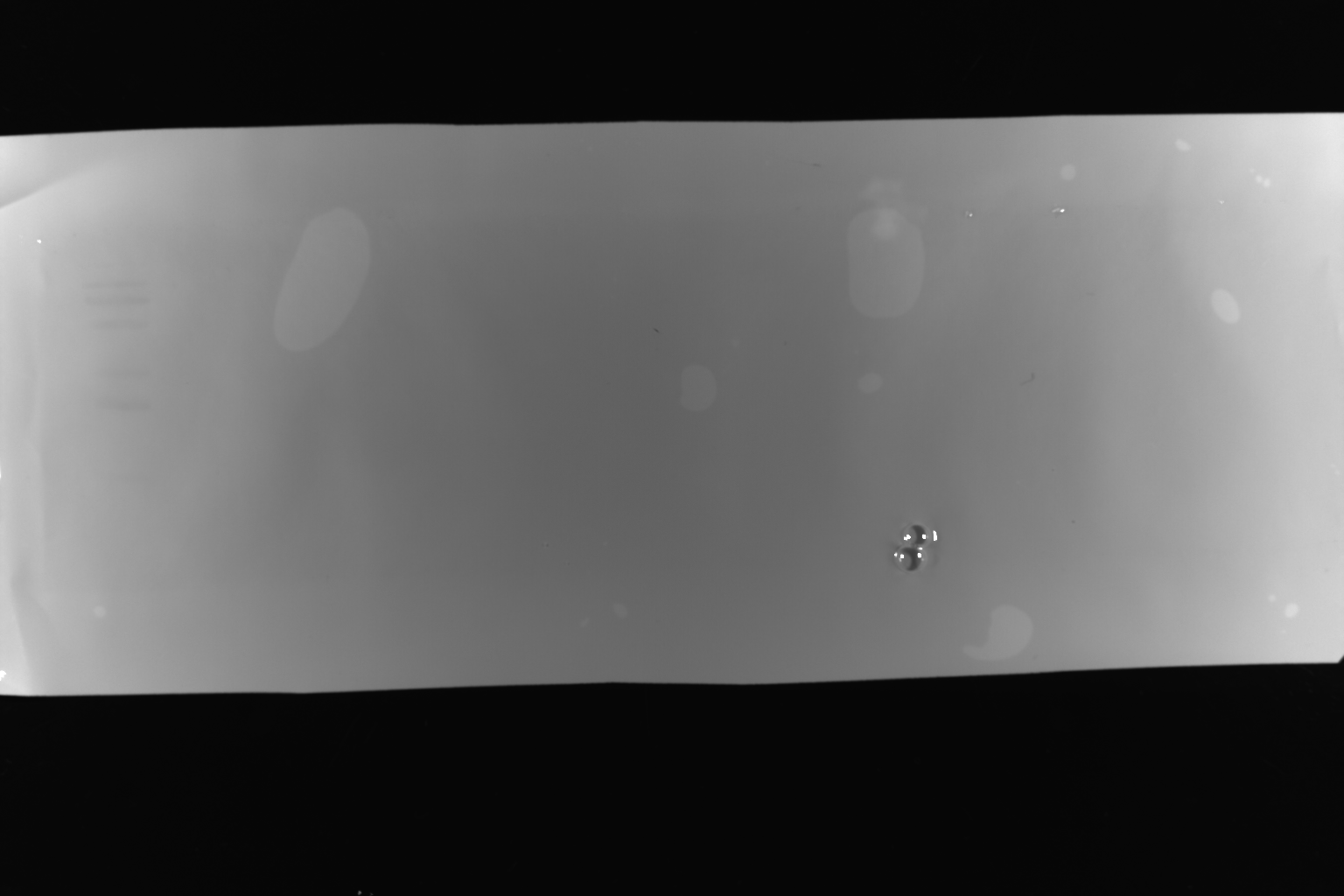

Supplement: Figure 1—figure supplement 4—source data 1. [file elife-69064-fig1-figsupp4-data1.zip › Source data - Figure 1 - figure supplement 4 - Source Data 1/Fig 1 - supp 4A - 20200220_1353 gel 1 GroEL reprobe ladder.tif]

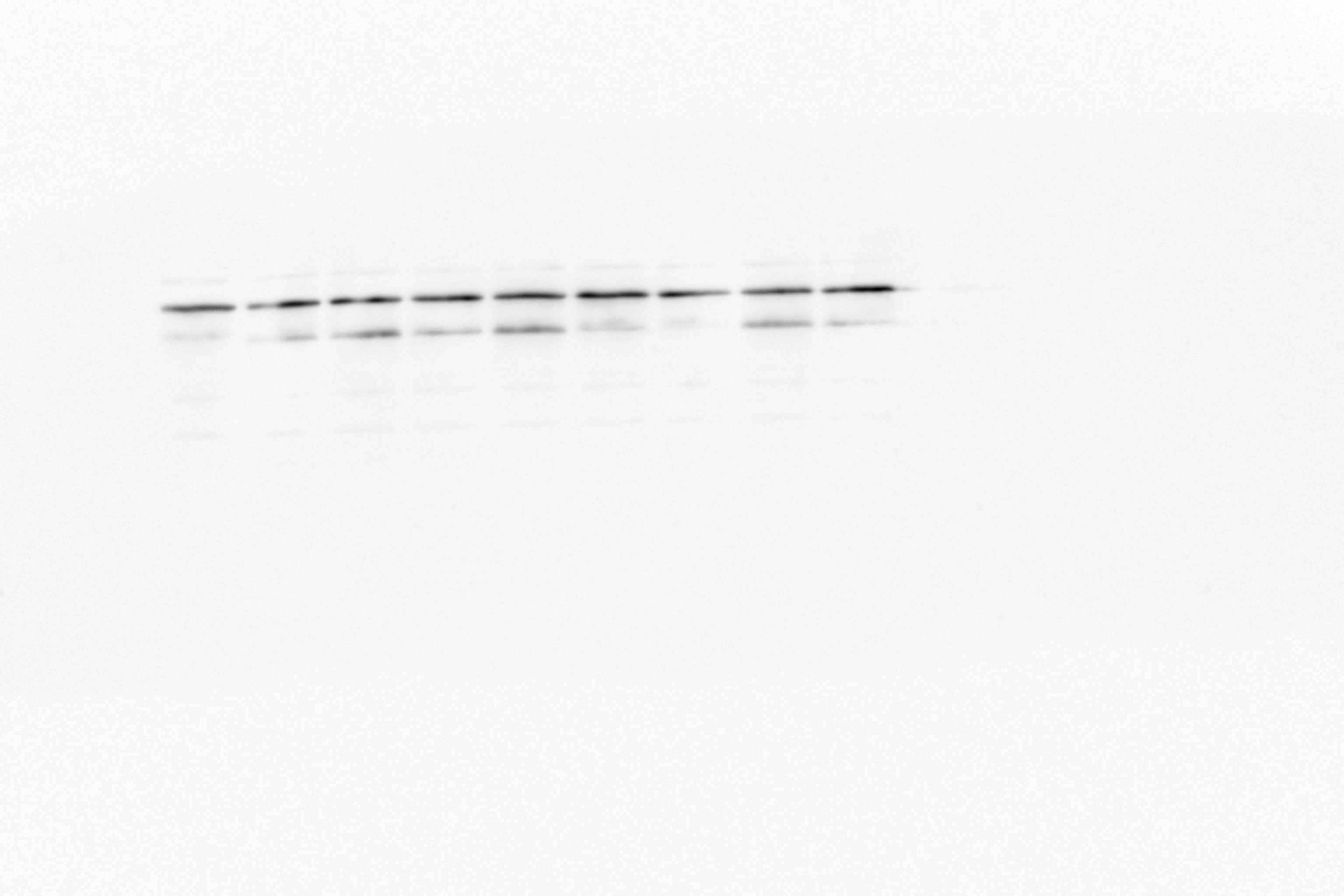

Supplement: Figure 1—figure supplement 4—source data 1. [file elife-69064-fig1-figsupp4-data1.zip › Source data - Figure 1 - figure supplement 4 - Source Data 1/Fig 1 - supp 4A - 20200220_1403 gel 1 GroEL reprobe_31.tif]

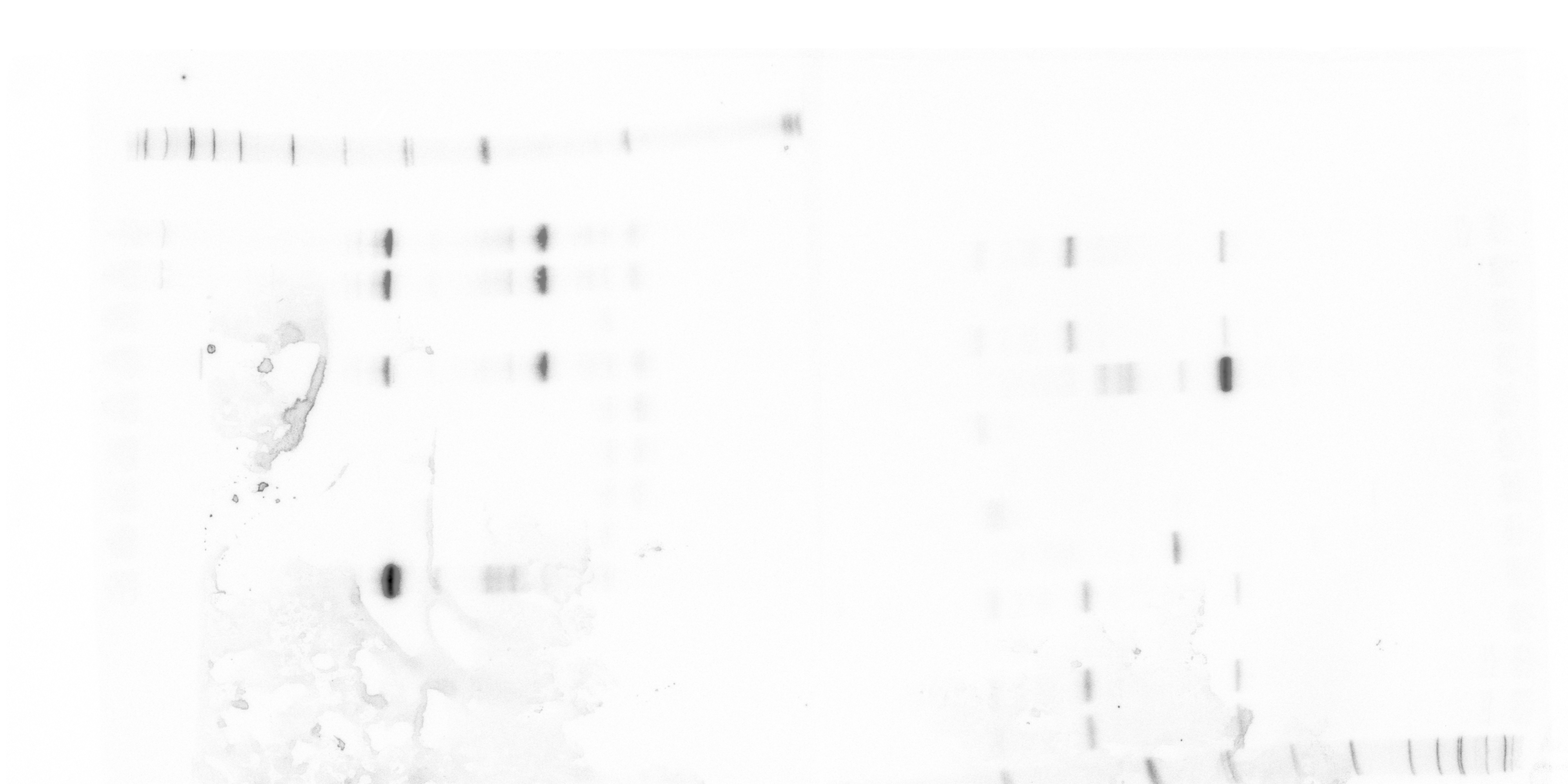

Supplement: Figure 1—figure supplement 4—source data 1. [file elife-69064-fig1-figsupp4-data1.zip › Source data - Figure 1 - figure supplement 4 - Source Data 1/Fig 1 - supp 4A - 9.3.2020_NB187_188_CSO-0189_6d-[Phosphor].tif]

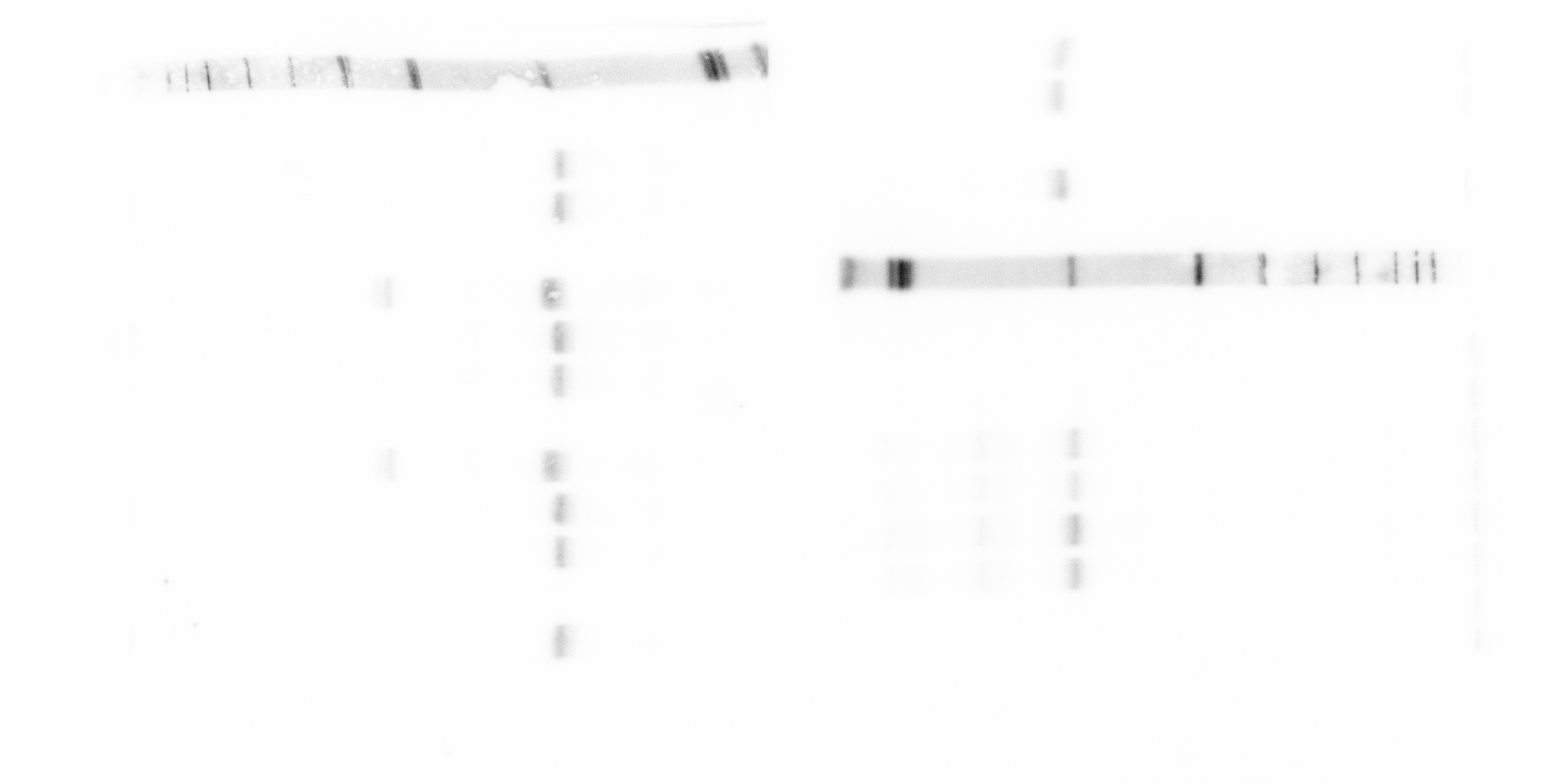

Supplement: Figure 1—figure supplement 4—source data 2. [file elife-69064-fig1-figsupp4-data2.zip › Source data - Figure 1 - figure supplement 4 - Source Data 2/Fig 1 - supp 4B - 2016061_NB77_78_CSO-0185_3d-[Phosphor].tif]

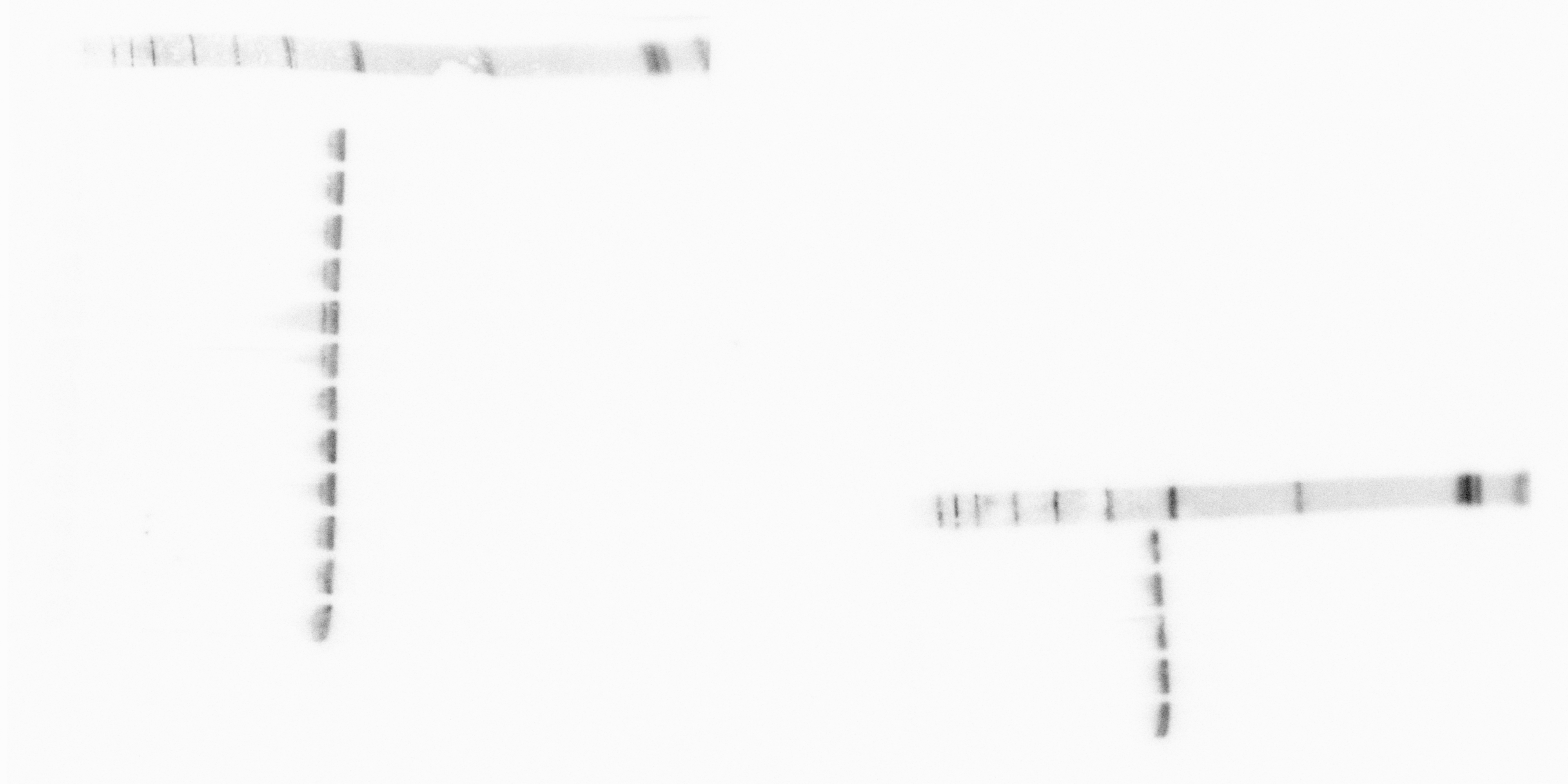

Supplement: Figure 1—figure supplement 4—source data 2. [file elife-69064-fig1-figsupp4-data2.zip › Source data - Figure 1 - figure supplement 4 - Source Data 2/Fig 1 - supp 4B - 20160626_NB77_78_CSO-0192_ON-[Phosphor].tif]

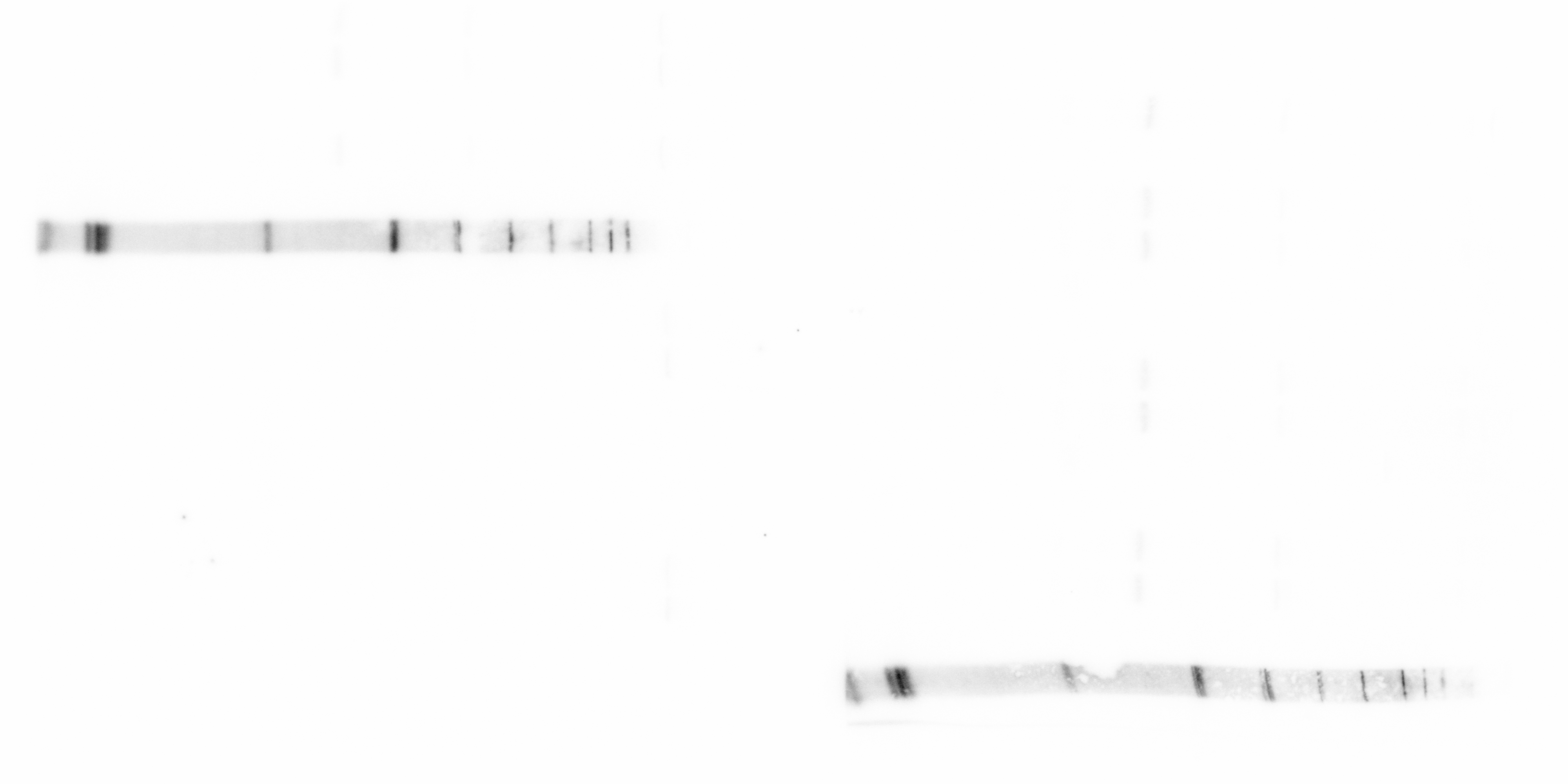

Supplement: Figure 1—figure supplement 4—source data 2. [file elife-69064-fig1-figsupp4-data2.zip › Source data - Figure 1 - figure supplement 4 - Source Data 2/Fig 1 - supp 4B - 24.06.2016_NB77_78_CSO-0189_5d-[Phosphor].tif]

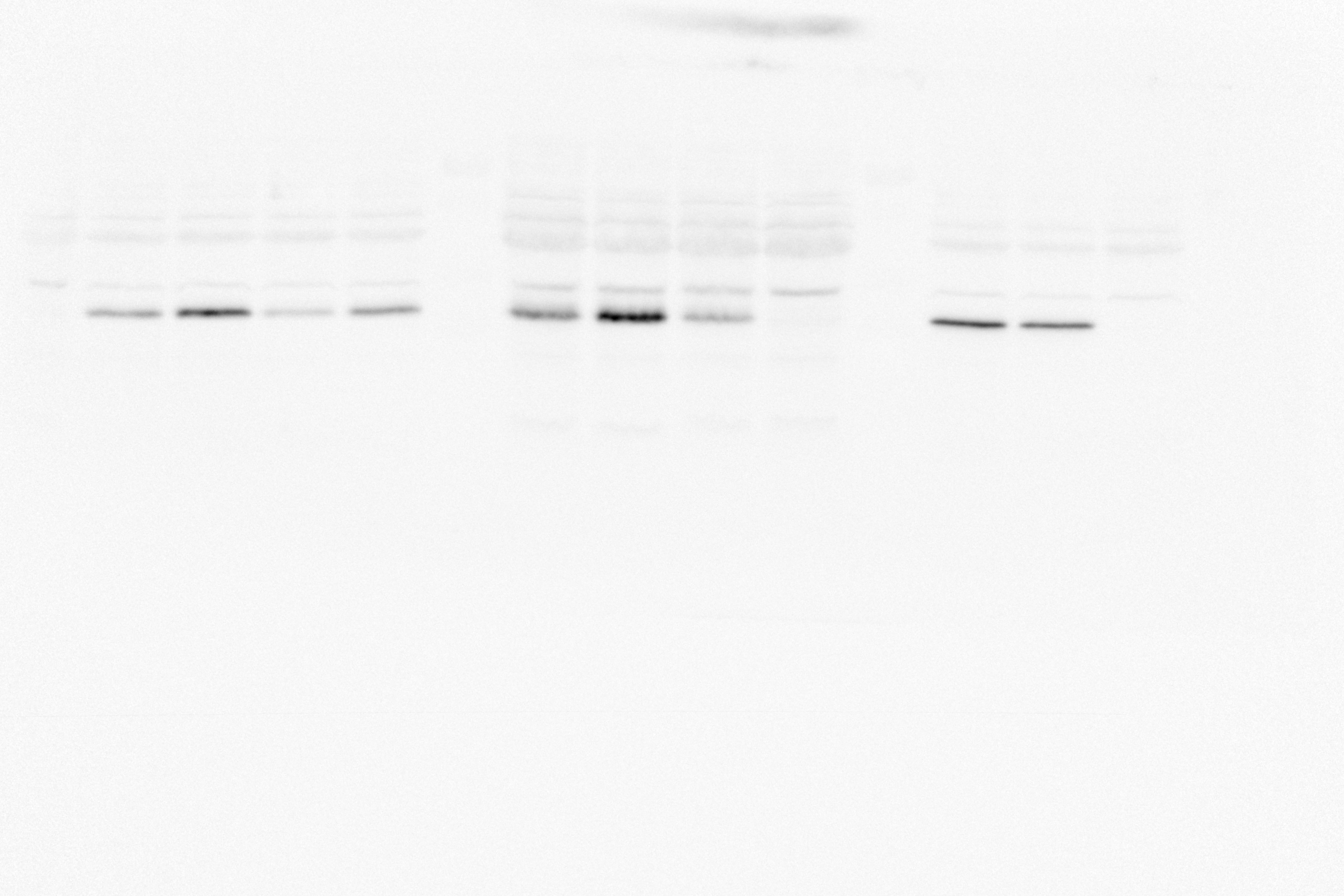

Supplement: Figure 1—figure supplement 4—source data 2. [file elife-69064-fig1-figsupp4-data2.zip › Source data - Figure 1 - figure supplement 4 - Source Data 2/Fig 1 - supp 4B - blot 1 ptmG GFP_13.tif]

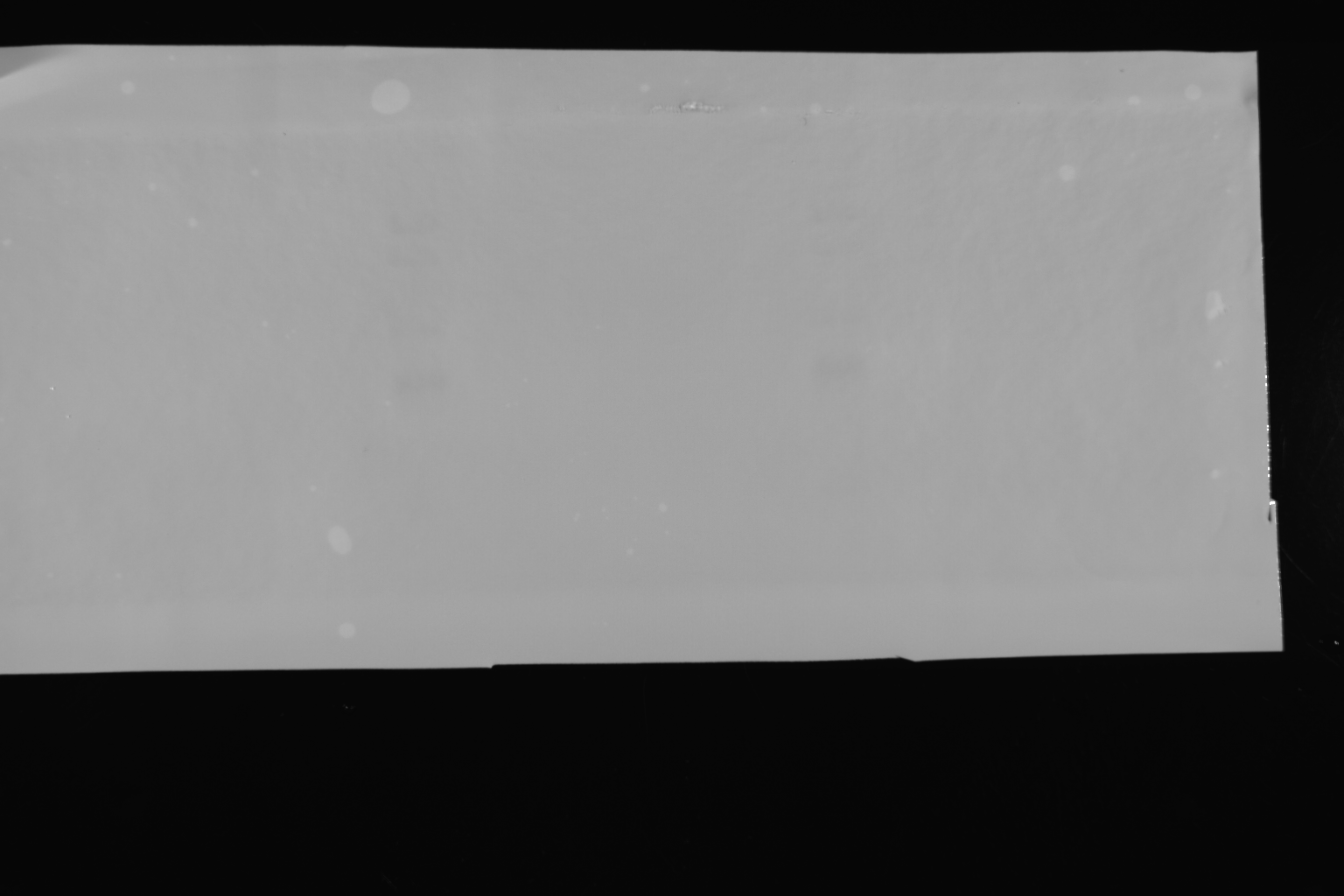

Supplement: Figure 1—figure supplement 4—source data 2. [file elife-69064-fig1-figsupp4-data2.zip › Source data - Figure 1 - figure supplement 4 - Source Data 2/Fig 1 - supp 4B - blot 1 ptmG GroEL ladder.tif]

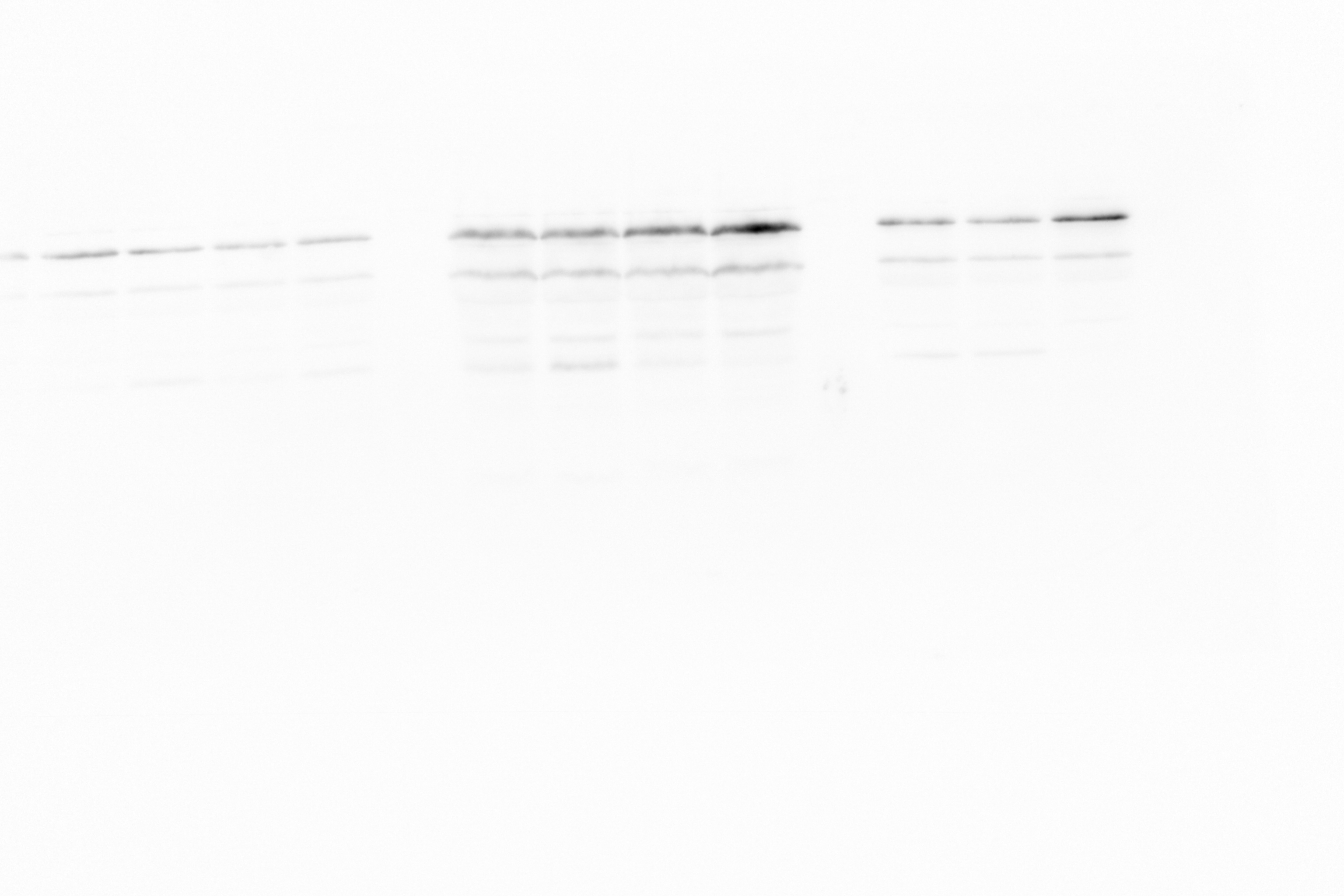

Supplement: Figure 1—figure supplement 4—source data 2. [file elife-69064-fig1-figsupp4-data2.zip › Source data - Figure 1 - figure supplement 4 - Source Data 2/Fig 1 - supp 4B - blot 1 ptmG GroEL_9.tif]

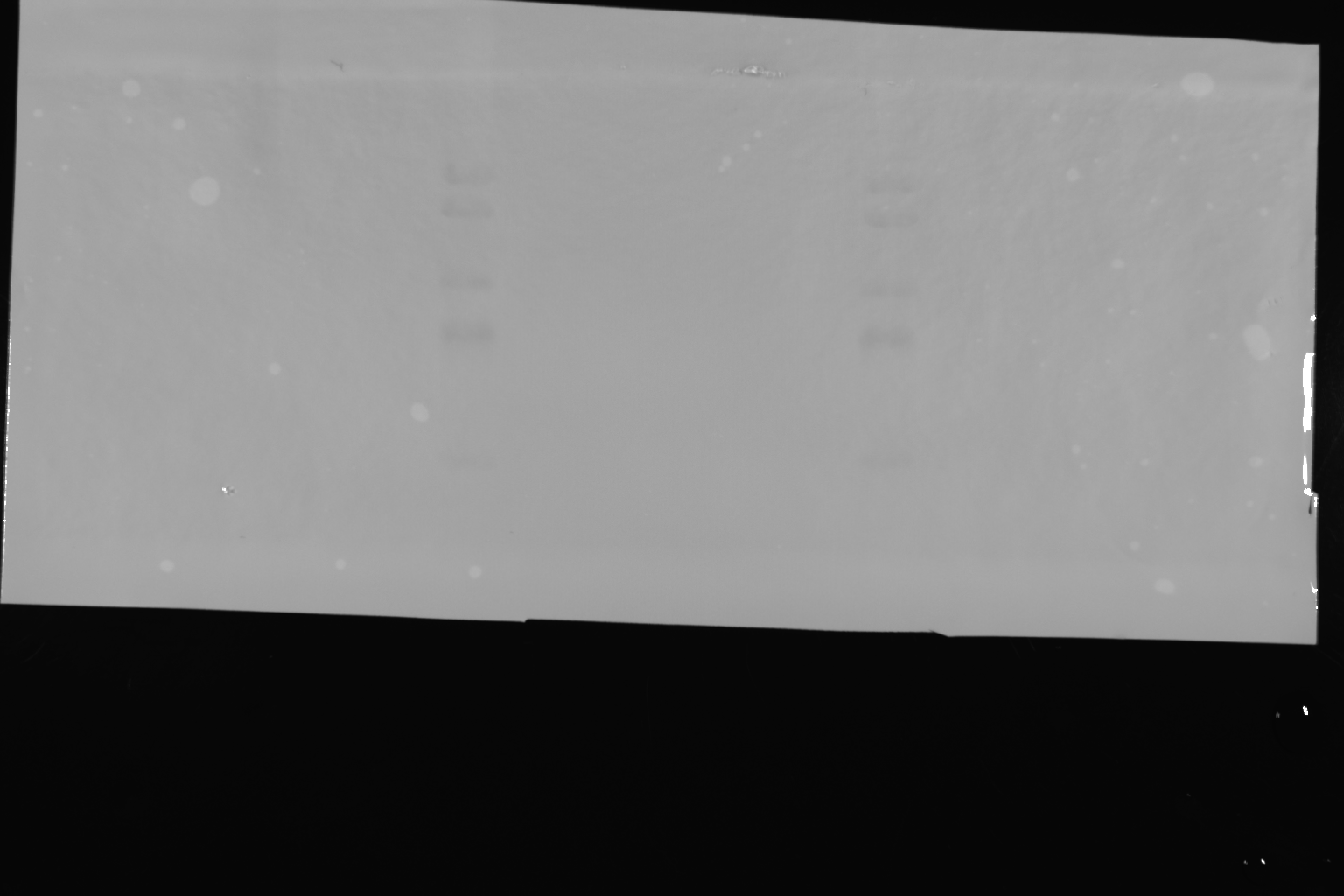

Supplement: Figure 1—figure supplement 4—source data 2. [file elife-69064-fig1-figsupp4-data2.zip › Source data - Figure 1 - figure supplement 4 - Source Data 2/Fig 1 - supp 4B - ptmG blot ladder.tif]

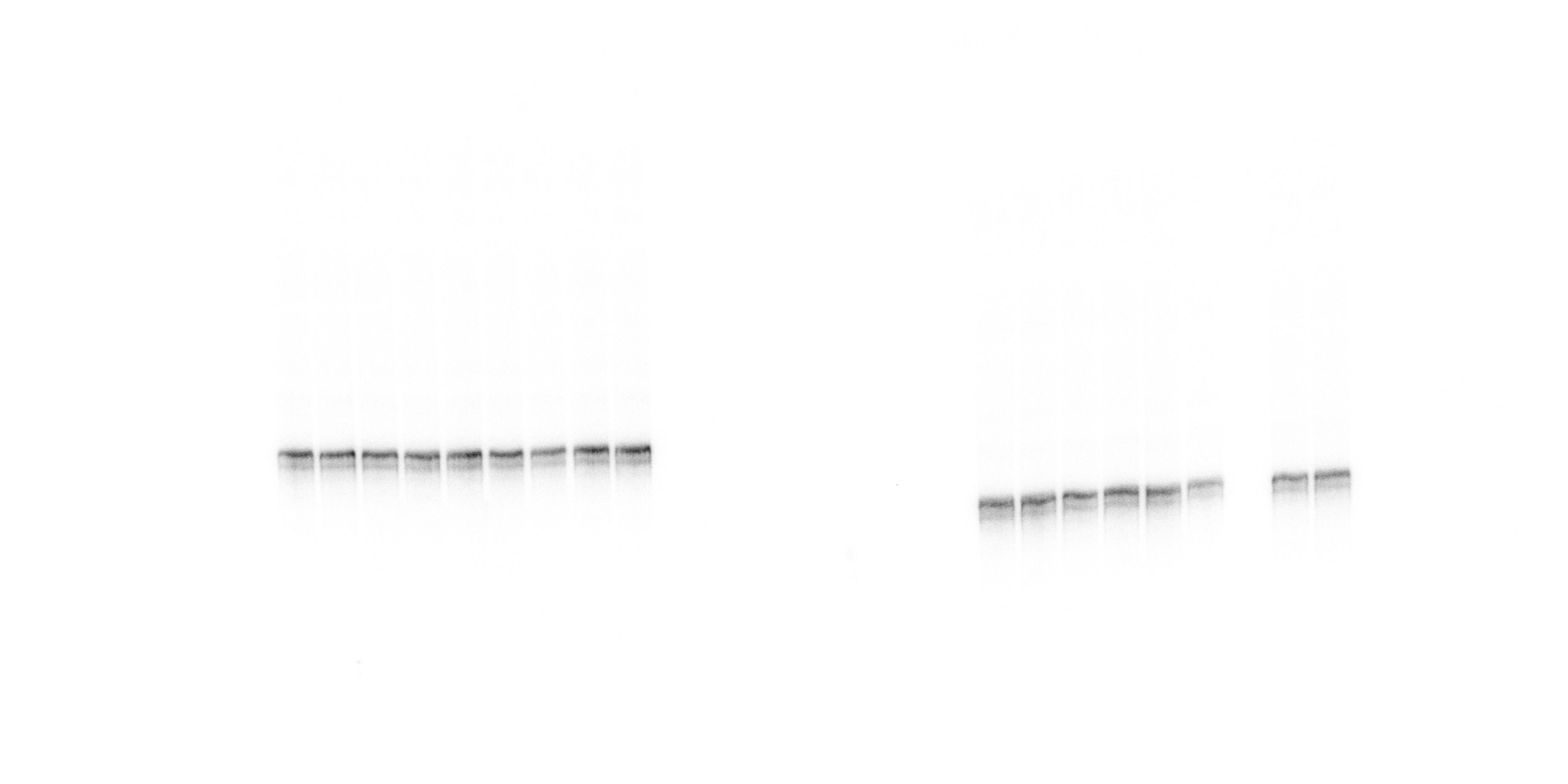

Supplement: Figure 2—source data 1. [file elife-69064-fig2-data1.zip › Source data - Figure 2/EMSA 1.tif]

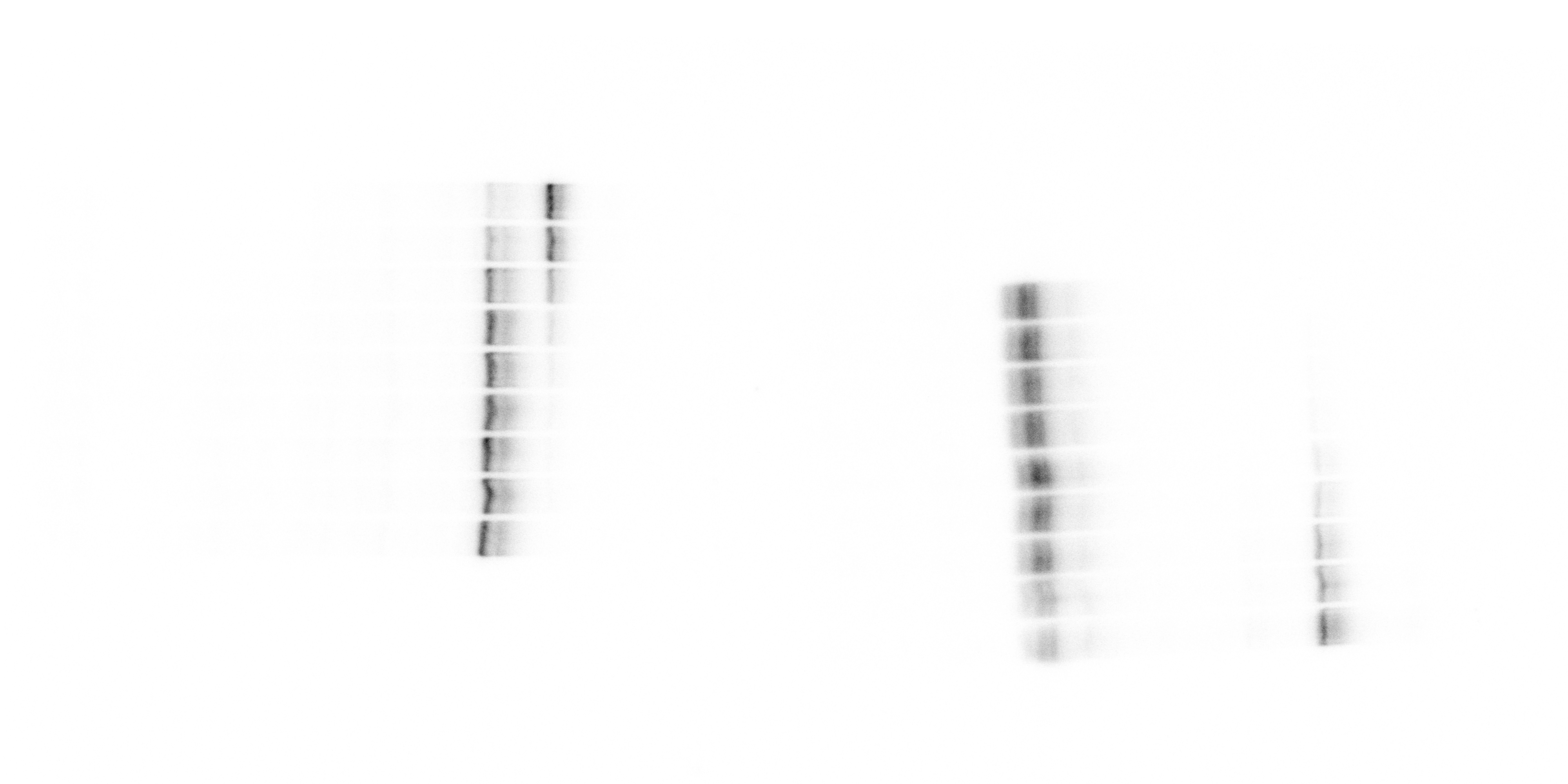

Supplement: Figure 2—source data 1. [file elife-69064-fig2-data1.zip › Source data - Figure 2/EMSA 2.tif]

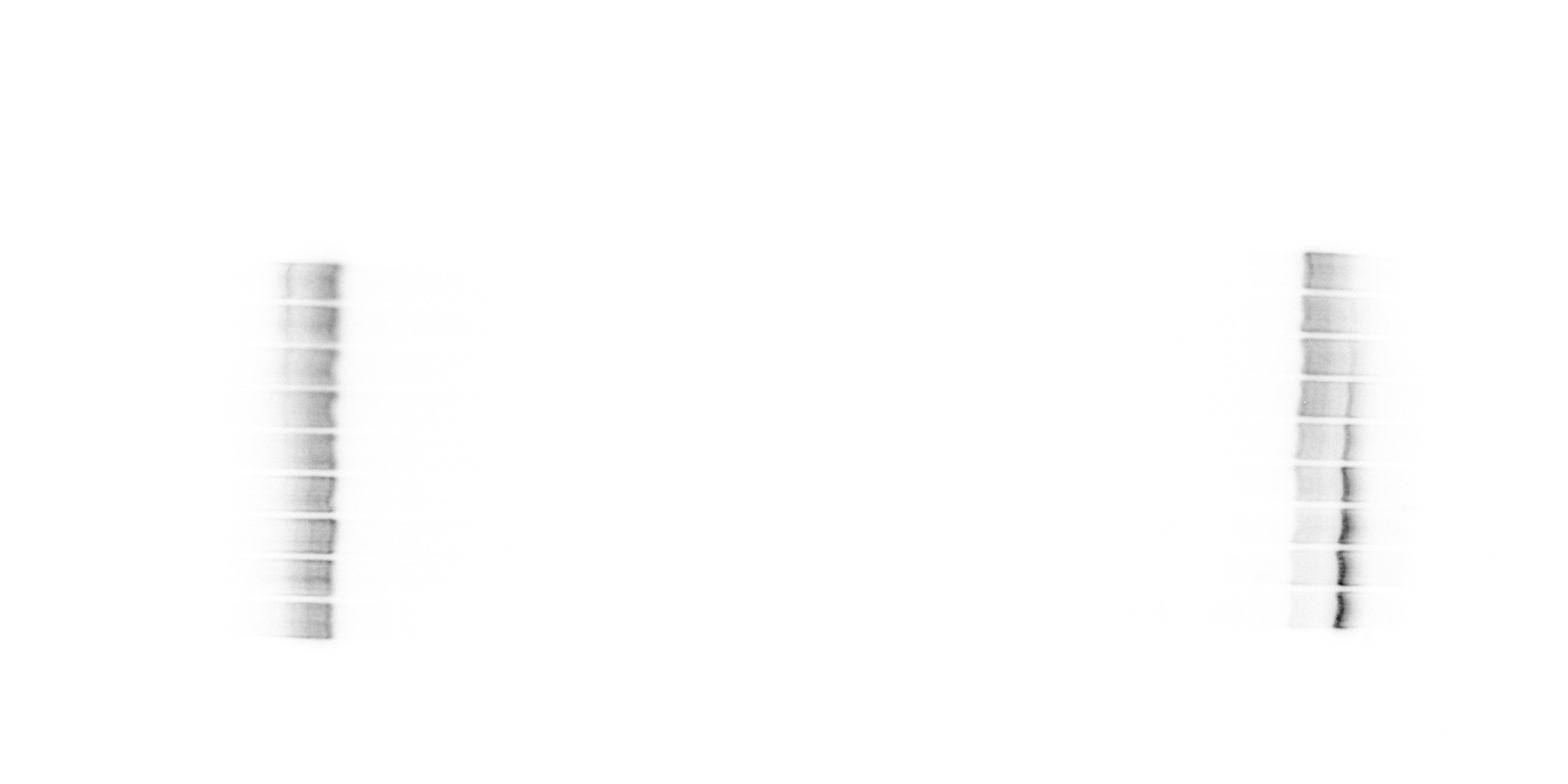

Supplement: Figure 2—source data 1. [file elife-69064-fig2-data1.zip › Source data - Figure 2/EMSA 3.tif]

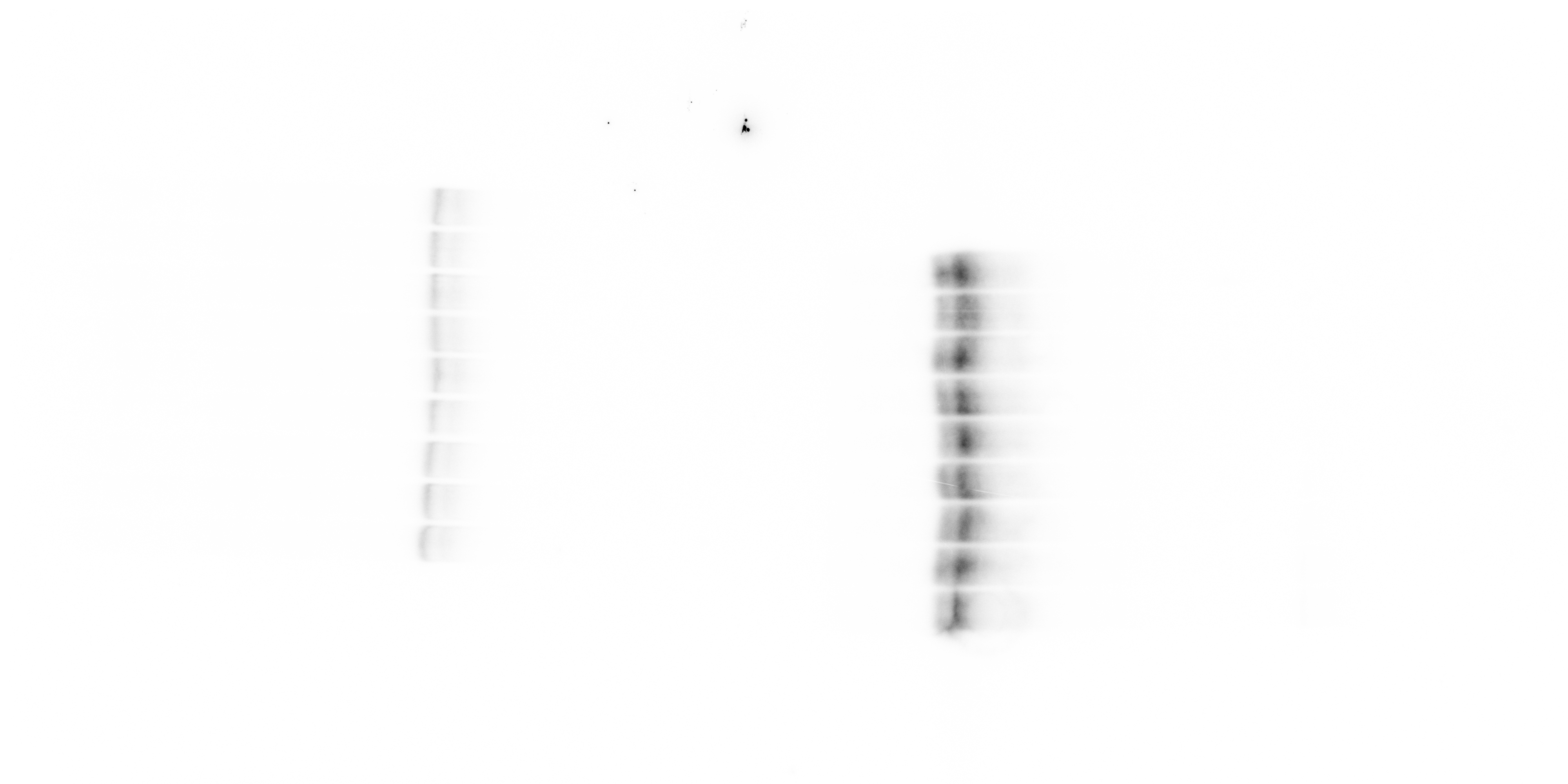

Supplement: Figure 2—source data 1. [file elife-69064-fig2-data1.zip › Source data - Figure 2/EMSA_4.tif]

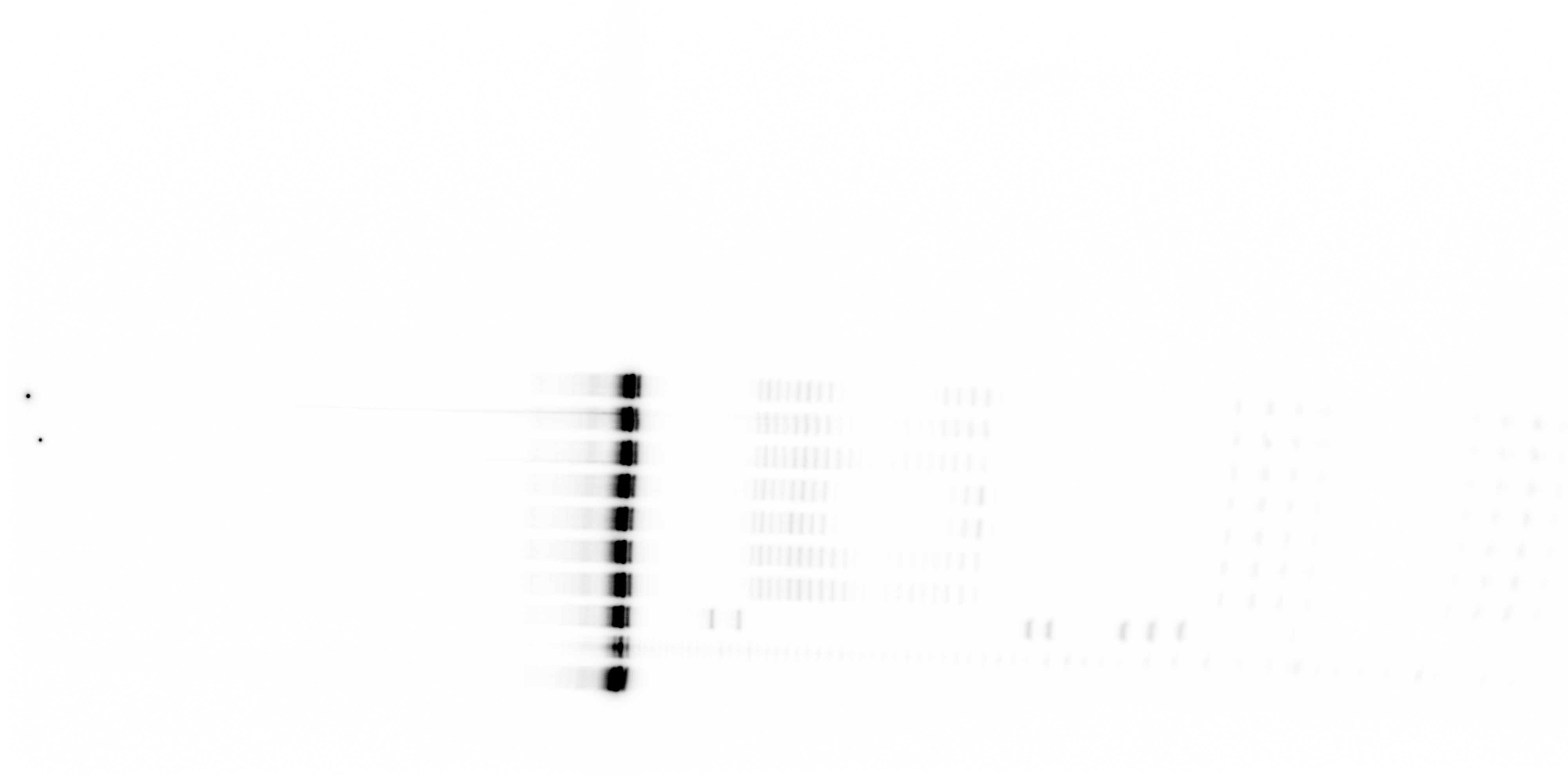

Supplement: Figure 2—source data 1. [file elife-69064-fig2-data1.zip › Source data - Figure 2/Figure 1C - inline scan 21.8.16 gel 1-[Phosphor].tif]

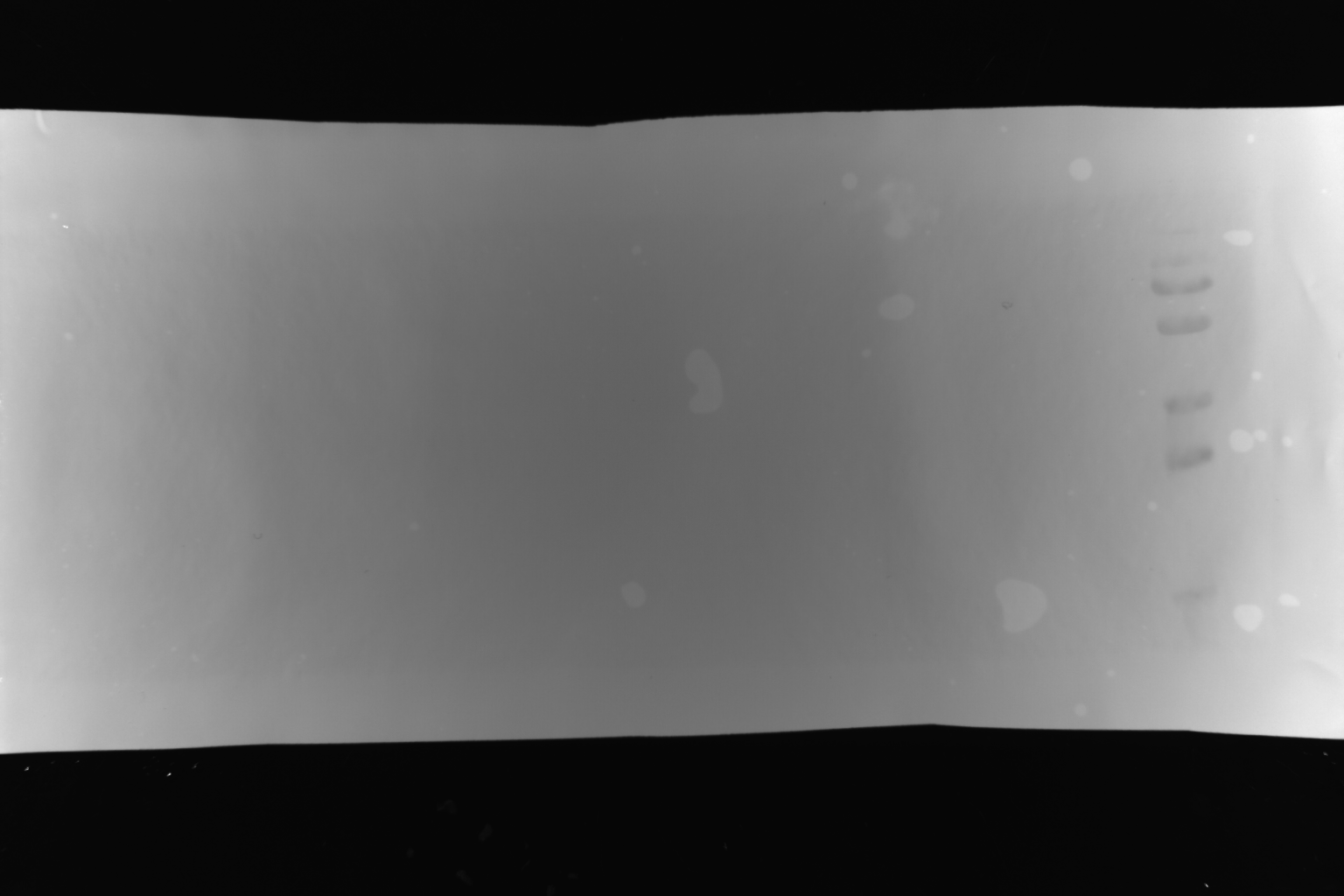

Supplement: Figure 2—source data 1. [file elife-69064-fig2-data1.zip › Source data - Figure 2/Figure 1D - GPF blot ladder.tif]

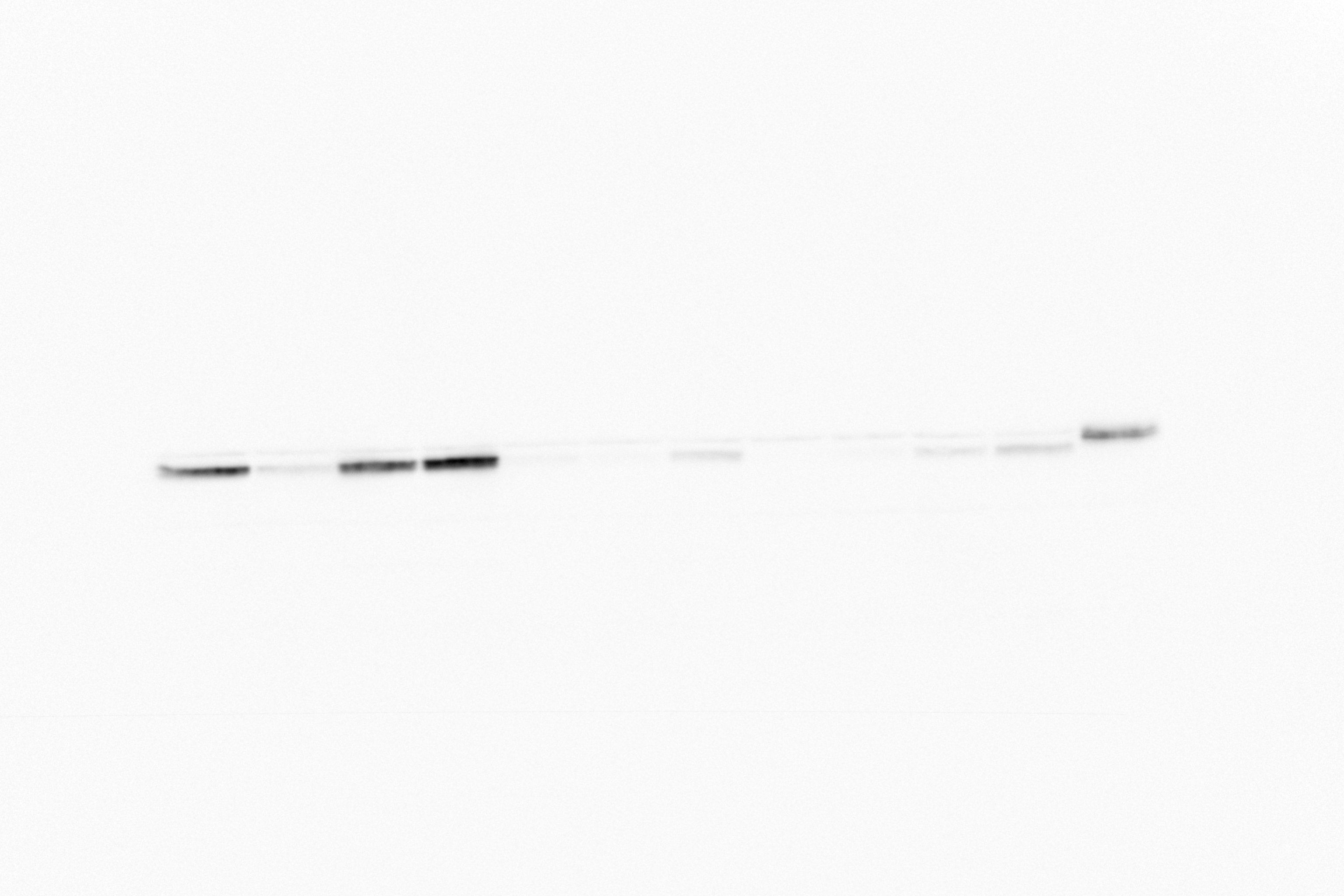

Supplement: Figure 2—source data 1. [file elife-69064-fig2-data1.zip › Source data - Figure 2/Figure 1D - GPF blot.tif]

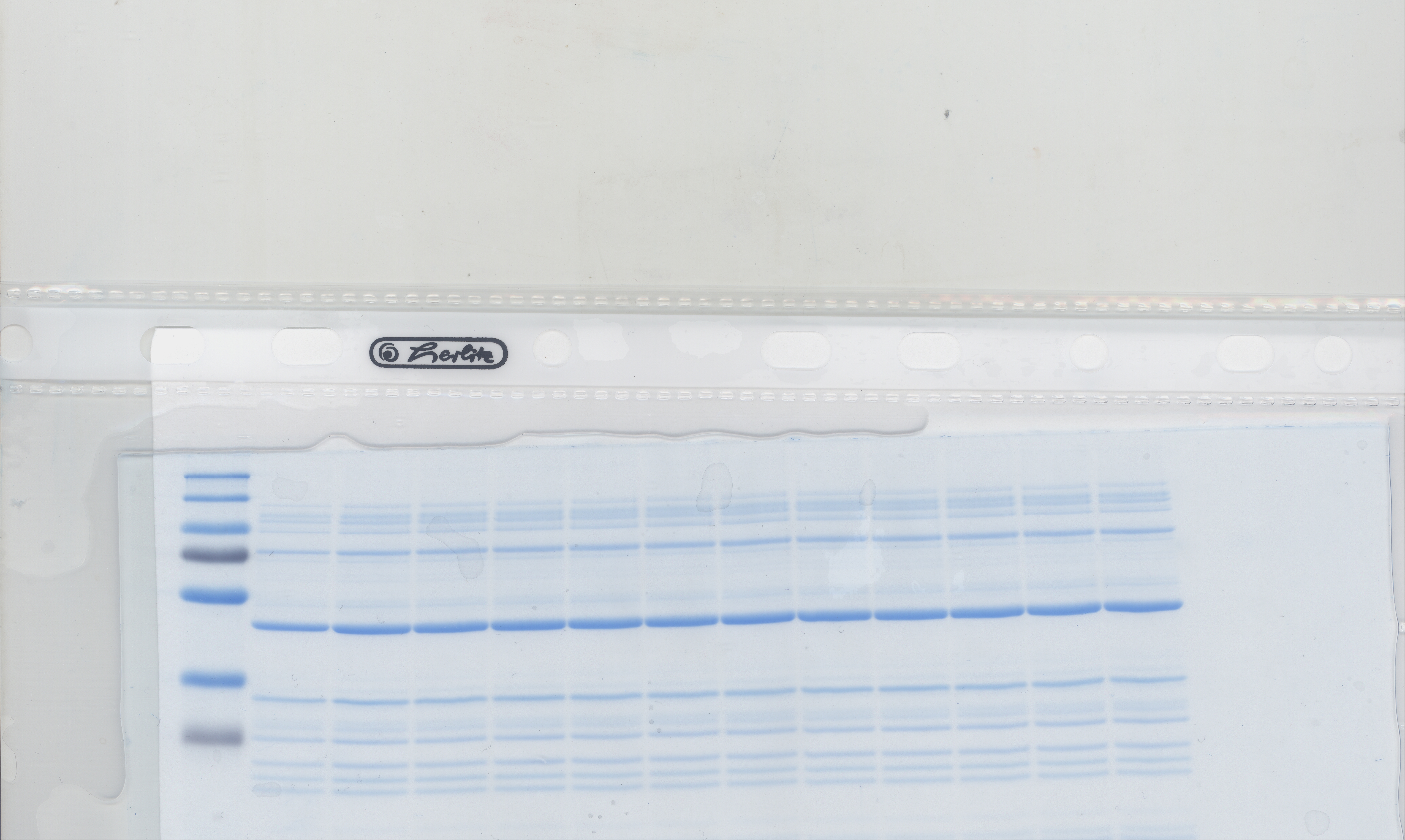

Supplement: Figure 2—source data 1. [file elife-69064-fig2-data1.zip › Source data - Figure 2/Figure 1D - SDS-PAGE.jpg]

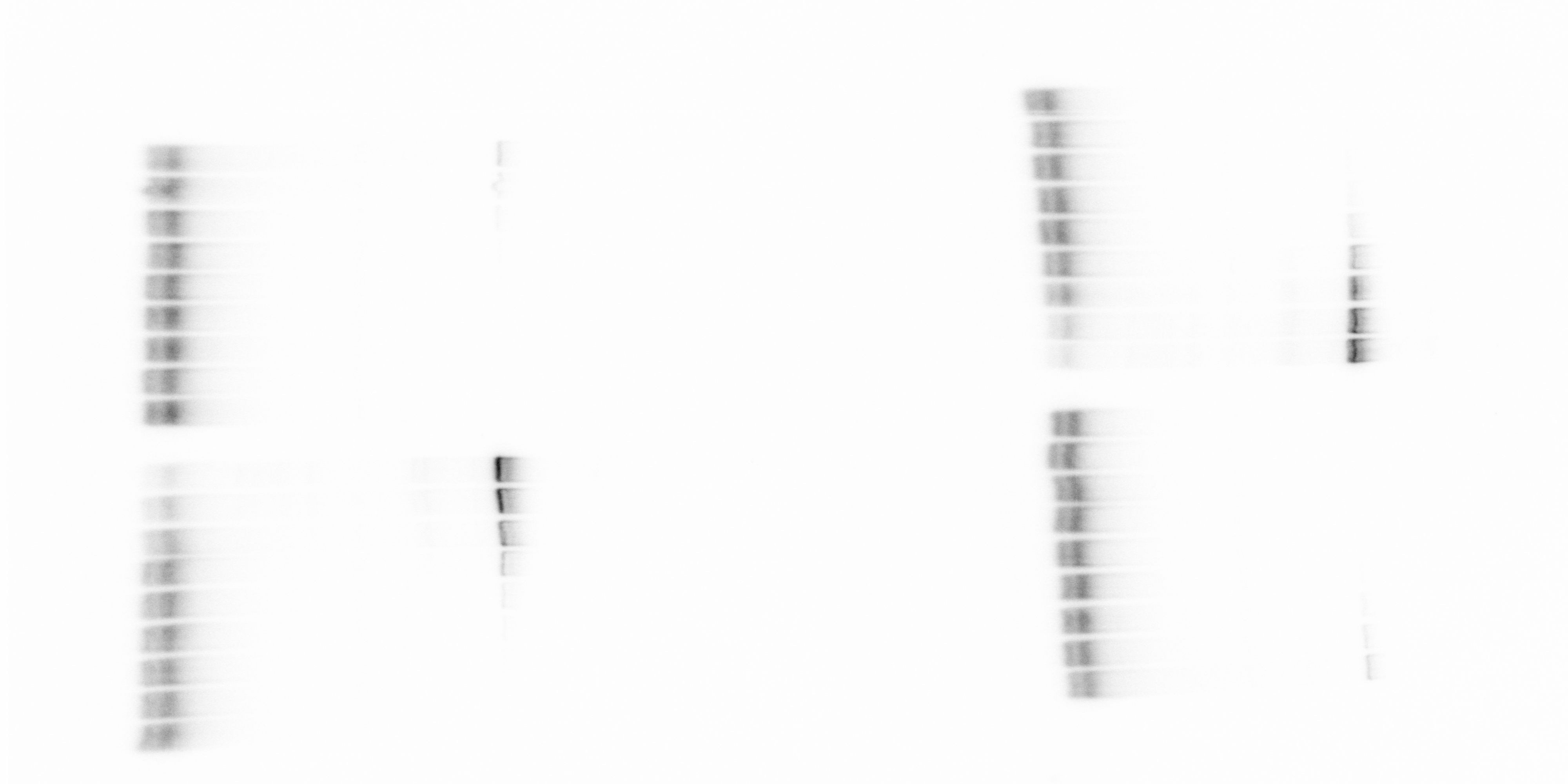

Supplement: Figure 2—figure supplement 1—source data 1. [file elife-69064-fig2-figsupp1-data1.zip › Source data - Figure 2 - figure supplement 1/Fig 2 - supp 1A - 20160621_EMSA_CJnc190-hot_3d-[Phosphor].tif]

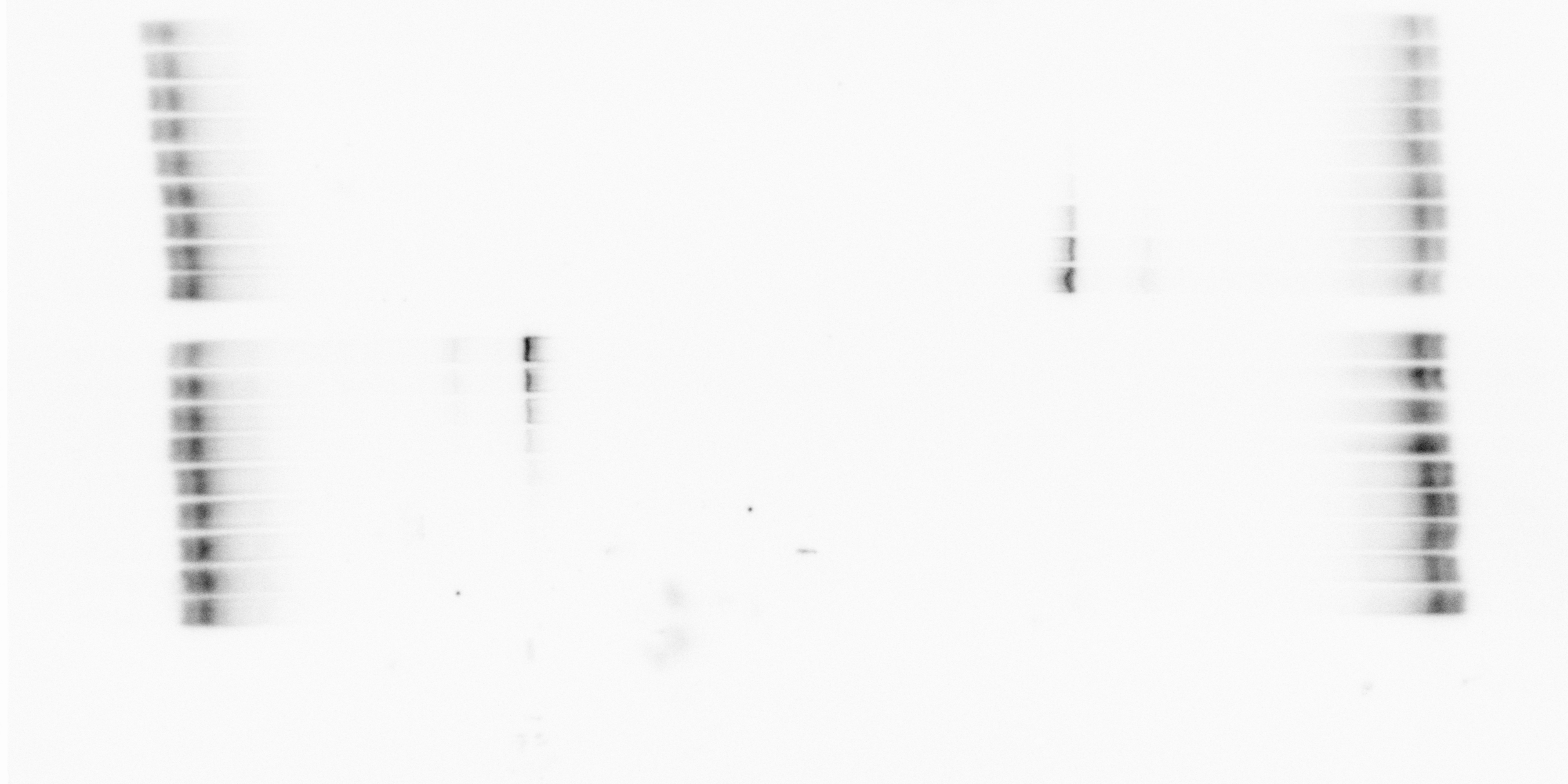

Supplement: Figure 2—figure supplement 1—source data 1. [file elife-69064-fig2-figsupp1-data1.zip › Source data - Figure 2 - figure supplement 1/Fig 2 - supp 1A - 23.7.2016_EMSA_190WT_vs_ptmGWT_M1_7d-[Phosphor].tif]

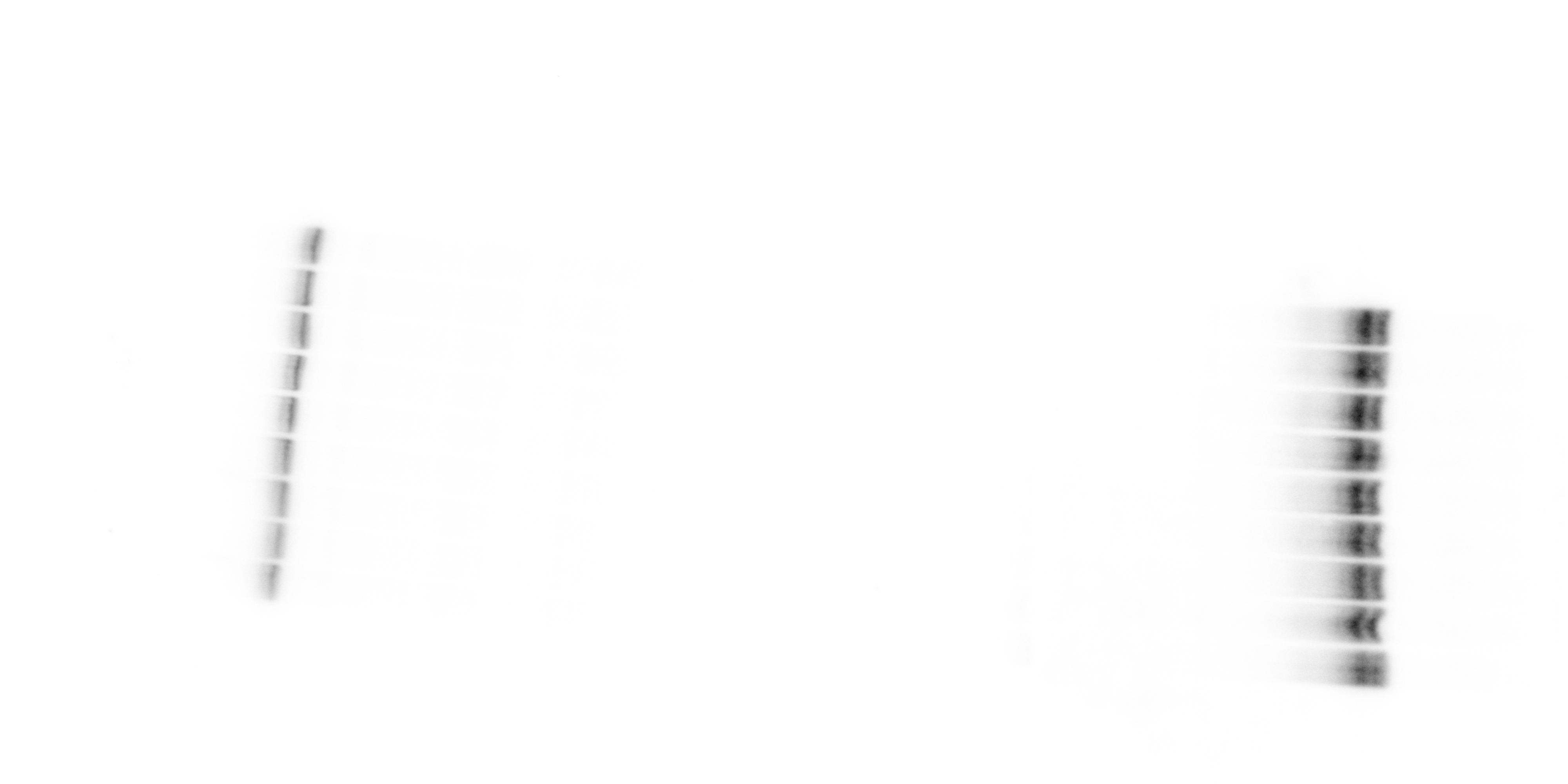

Supplement: Figure 2—figure supplement 1—source data 1. [file elife-69064-fig2-figsupp1-data1.zip › Source data - Figure 2 - figure supplement 1/Fig 2 - supp 1A - EMSA2_131117190WTh+ptmGM1_ptmGM1h+190WT_4d-[Phosphor].tif]

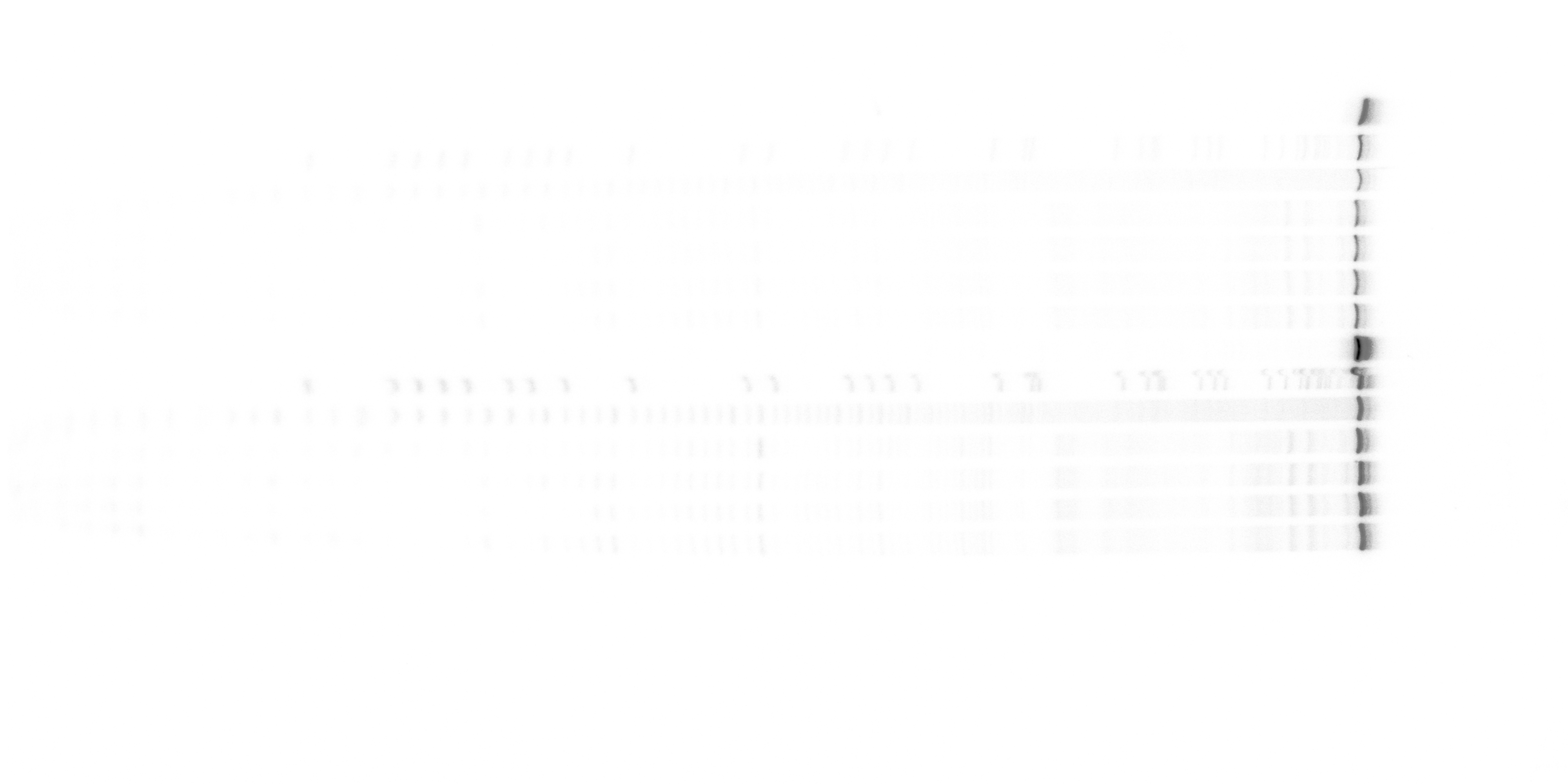

Supplement: Figure 2—figure supplement 1—source data 1. [file elife-69064-fig2-figsupp1-data1.zip › Source data - Figure 2 - figure supplement 1/Fig 2 - supp 1B - 30.5.2020_ptmG_2d-[Phosphor].tif]

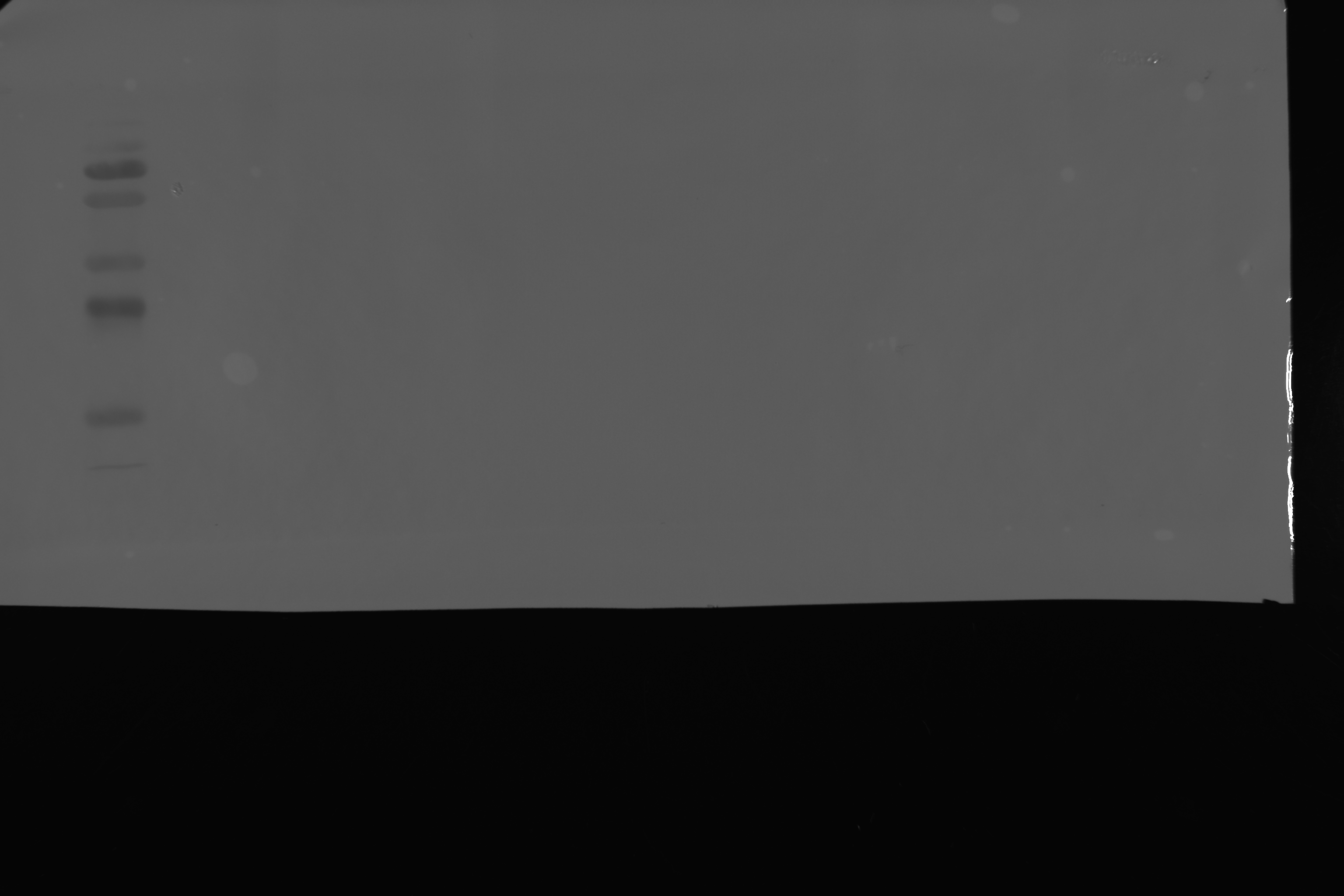

Supplement: Figure 2—figure supplement 1—source data 1. [file elife-69064-fig2-figsupp1-data1.zip › Source data - Figure 2 - figure supplement 1/Fig 2 - supp 1C - 20160414_1145_ladder.tif]

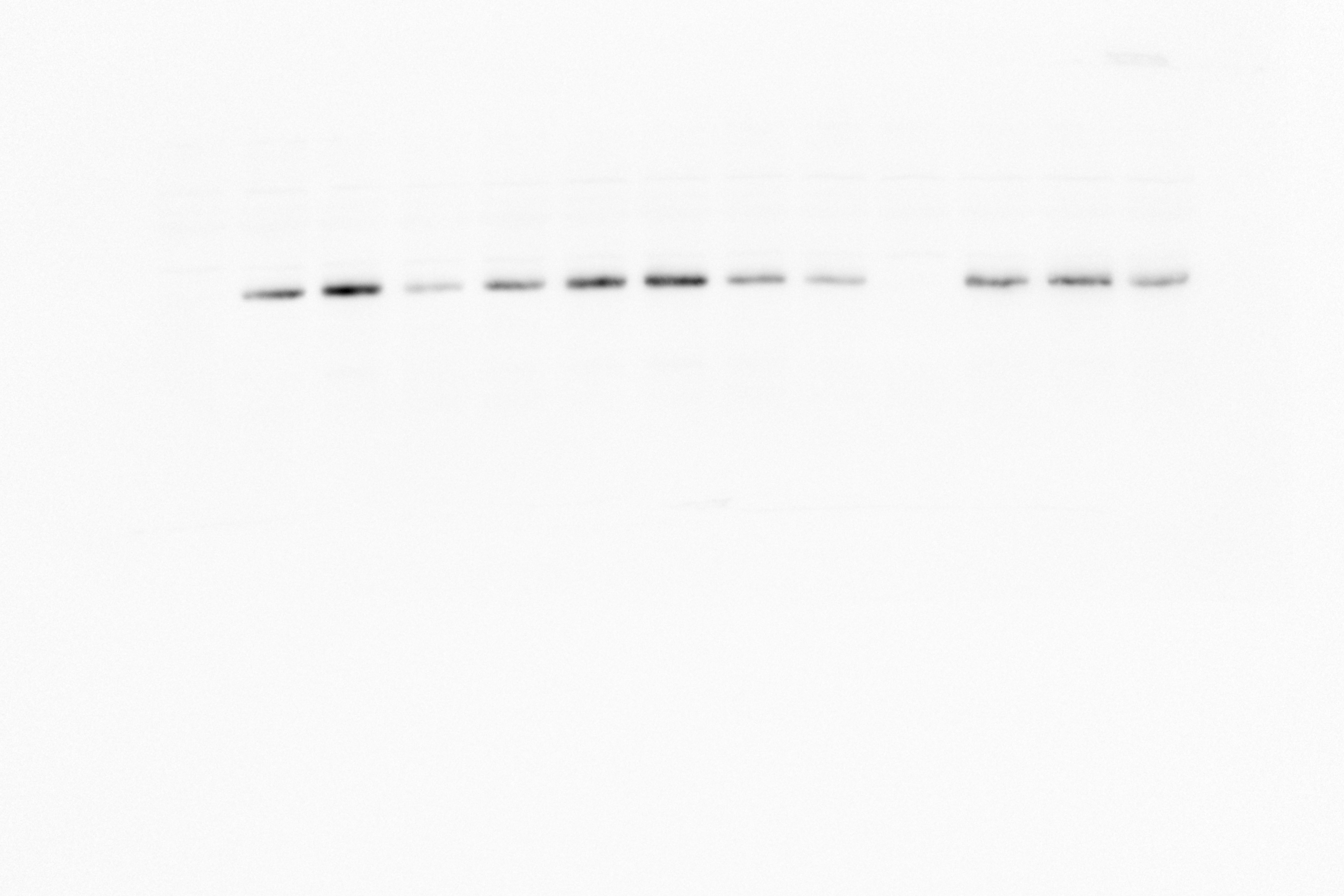

Supplement: Figure 2—figure supplement 1—source data 1. [file elife-69064-fig2-figsupp1-data1.zip › Source data - Figure 2 - figure supplement 1/Fig 2 - supp 1C - 20160414_1152_GFP_8.tif]

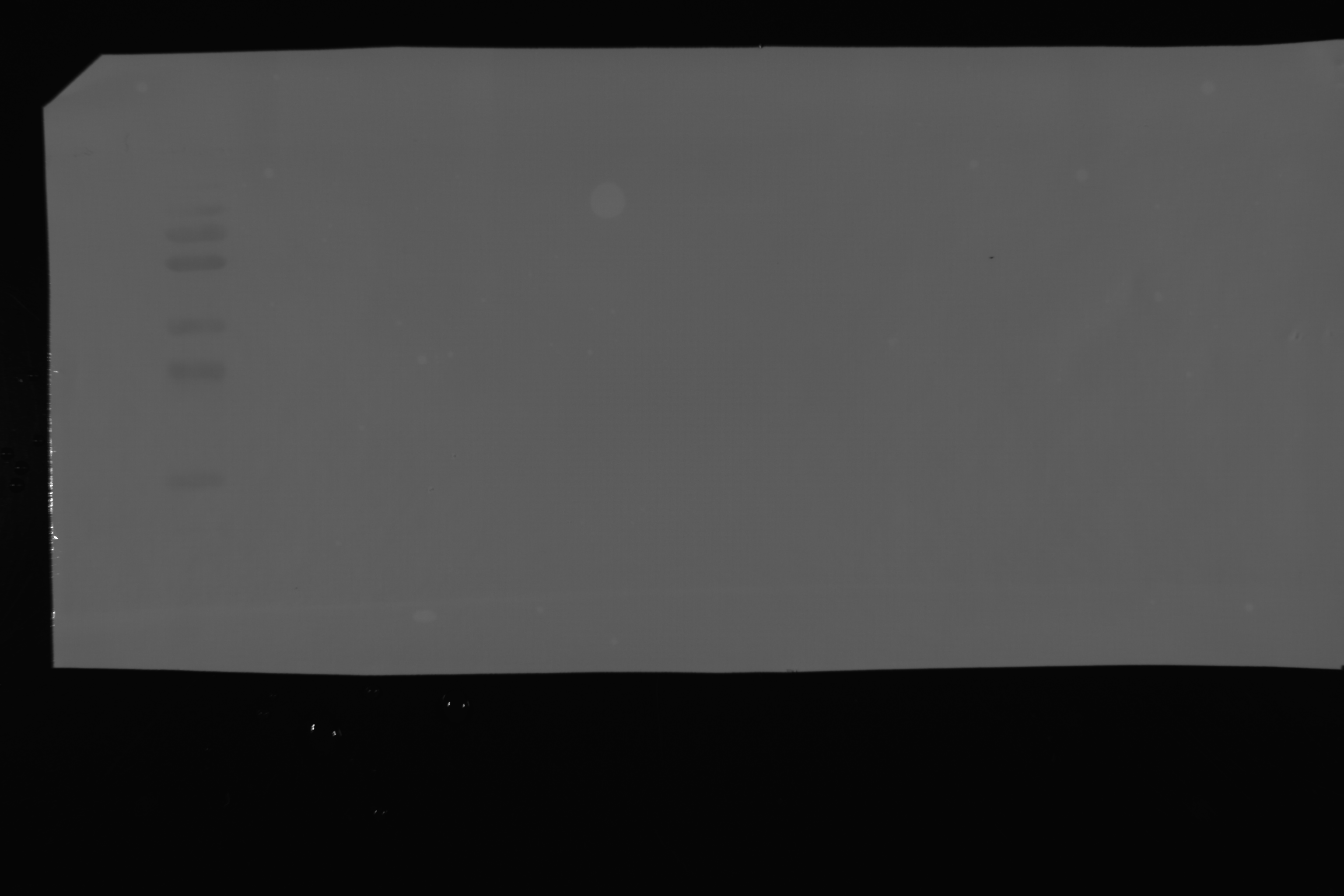

Supplement: Figure 2—figure supplement 1—source data 1. [file elife-69064-fig2-figsupp1-data1.zip › Source data - Figure 2 - figure supplement 1/Fig 2 - supp 1C - 20160415_1139_ladder.tif]

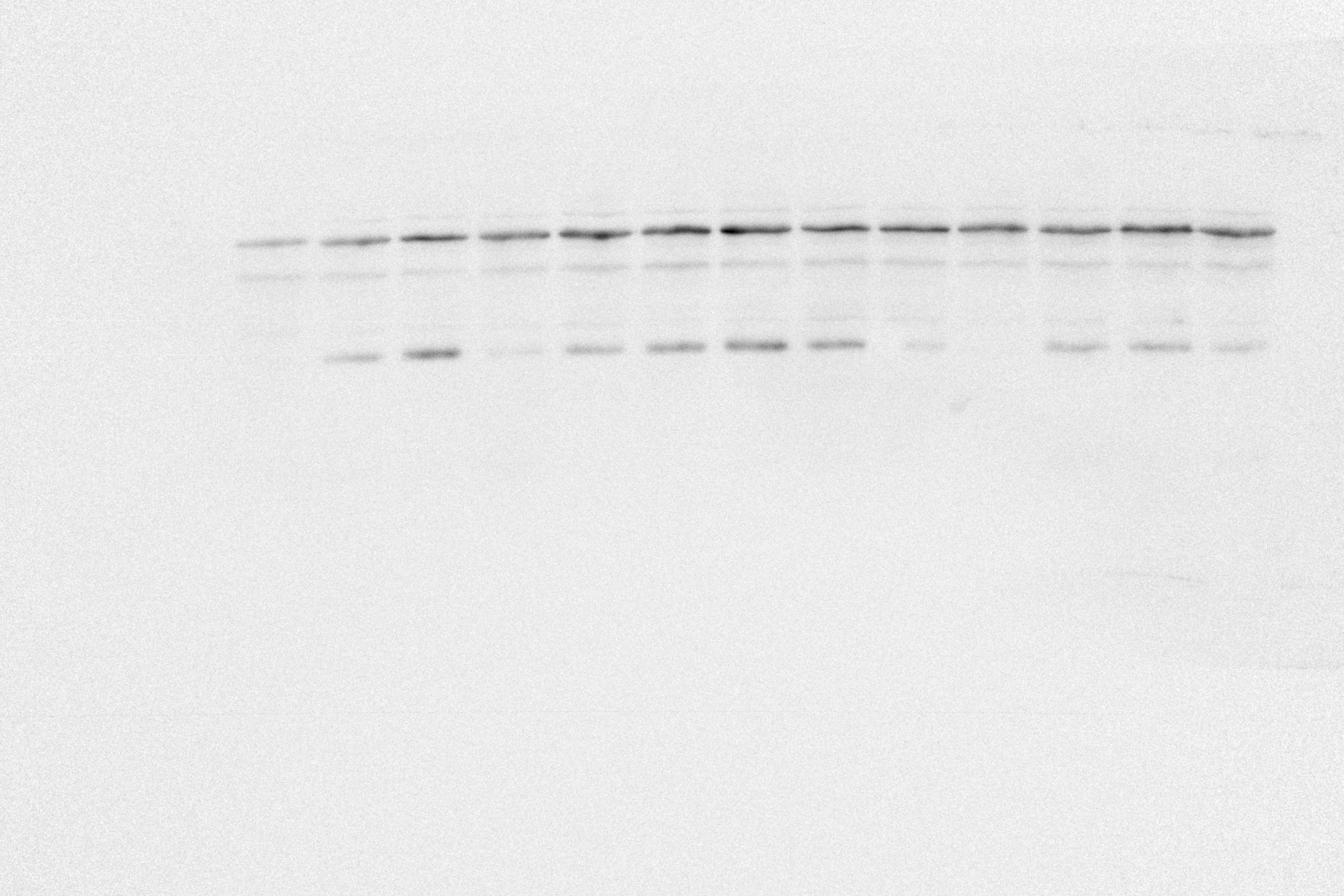

Supplement: Figure 2—figure supplement 1—source data 1. [file elife-69064-fig2-figsupp1-data1.zip › Source data - Figure 2 - figure supplement 1/Fig 2 - supp 1C - 20160415_1149_GroEL_15.tif]

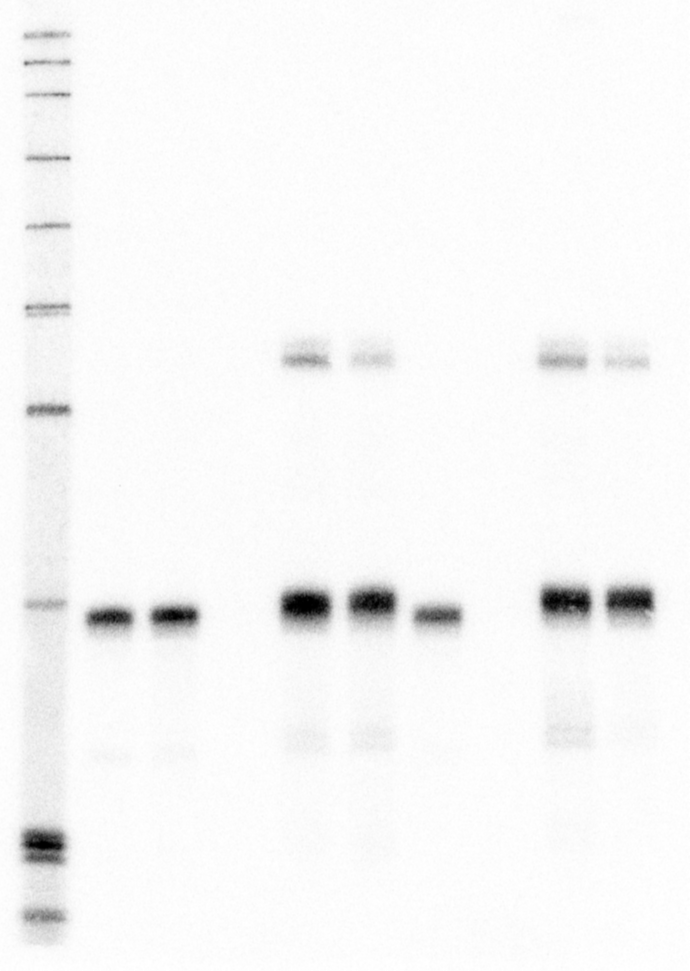

Supplement: Figure 2—figure supplement 1—source data 1. [file elife-69064-fig2-figsupp1-data1.zip › Source data - Figure 2 - figure supplement 1/Fig 2 - supp 1C - NB71_CSO-0185.tif]

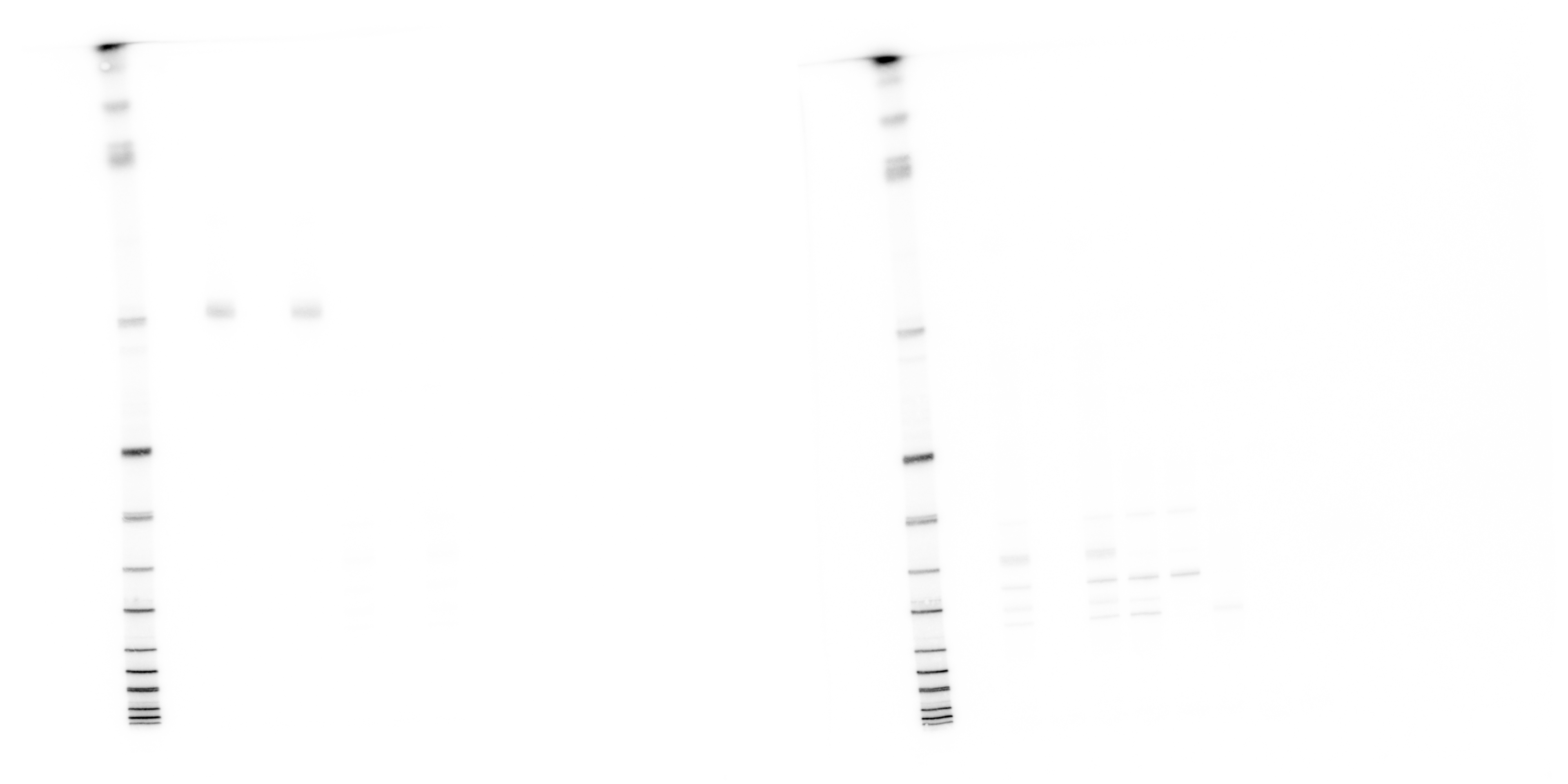

Supplement: Figure 3—source data 1. [file elife-69064-fig3-data1.zip › Source data - Figure 3/Figure 3A - 20171103_NB119_120_CSO-0185_5d-[Phosphor].tif]

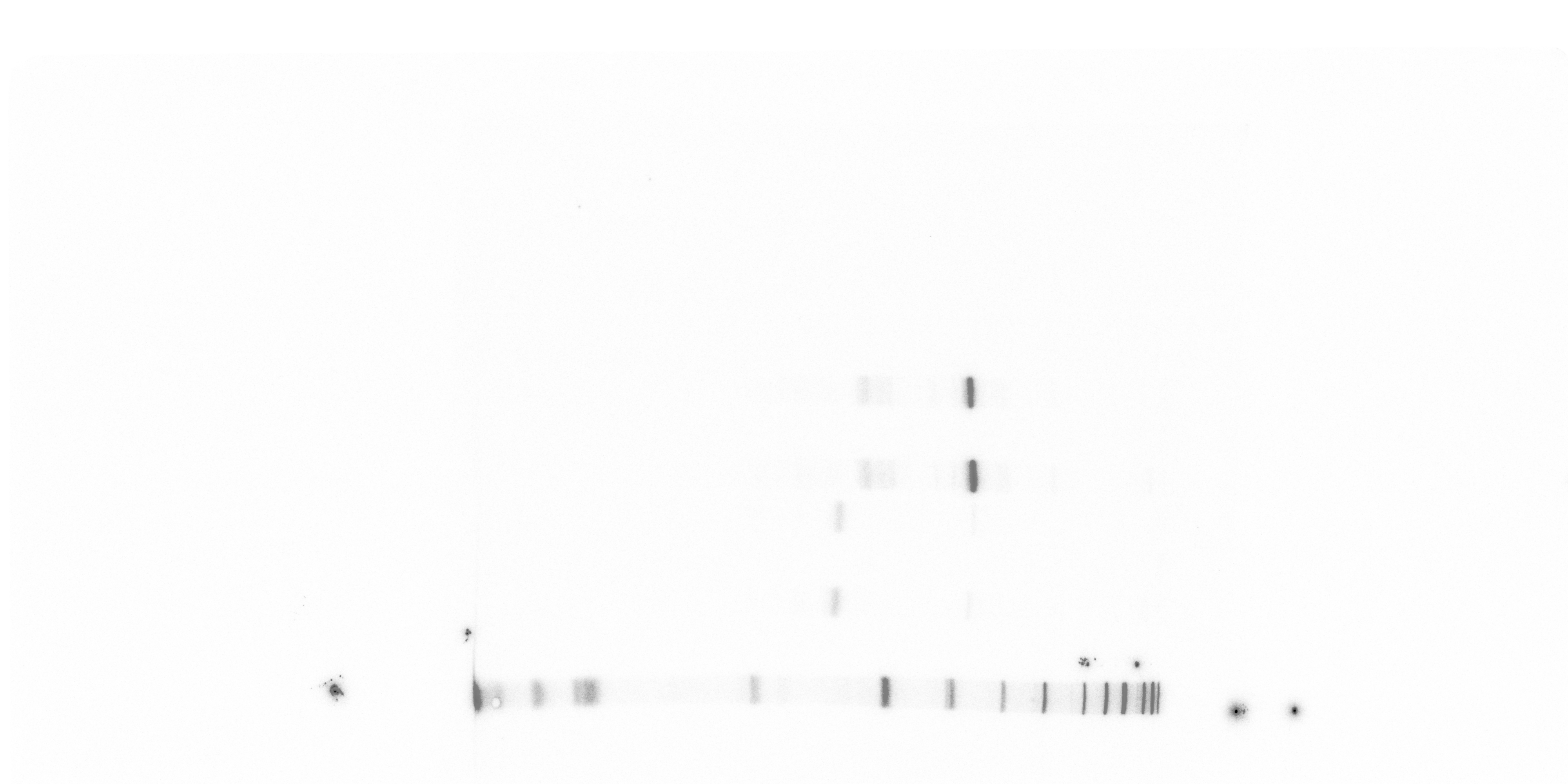

Supplement: Figure 3—source data 1. [file elife-69064-fig3-data1.zip › Source data - Figure 3/Figure 3A - 20171226_NB119_CSO-0189_7d-[Phosphor].tif]

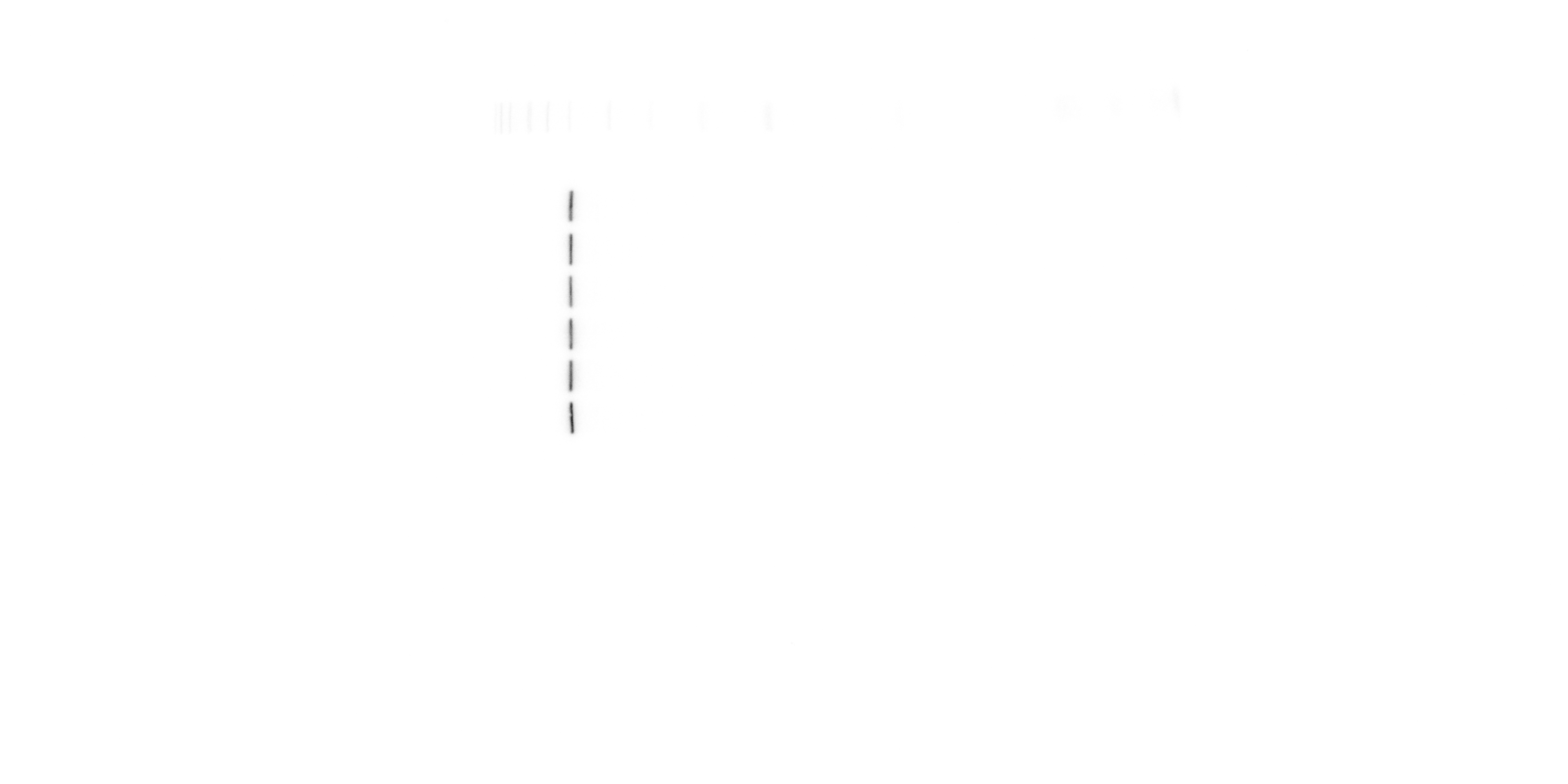

Supplement: Figure 3—source data 1. [file elife-69064-fig3-data1.zip › Source data - Figure 3/Figure 3A - 7.1.2018_NB119_CSO-0497_1d-[Phosphor].tif]

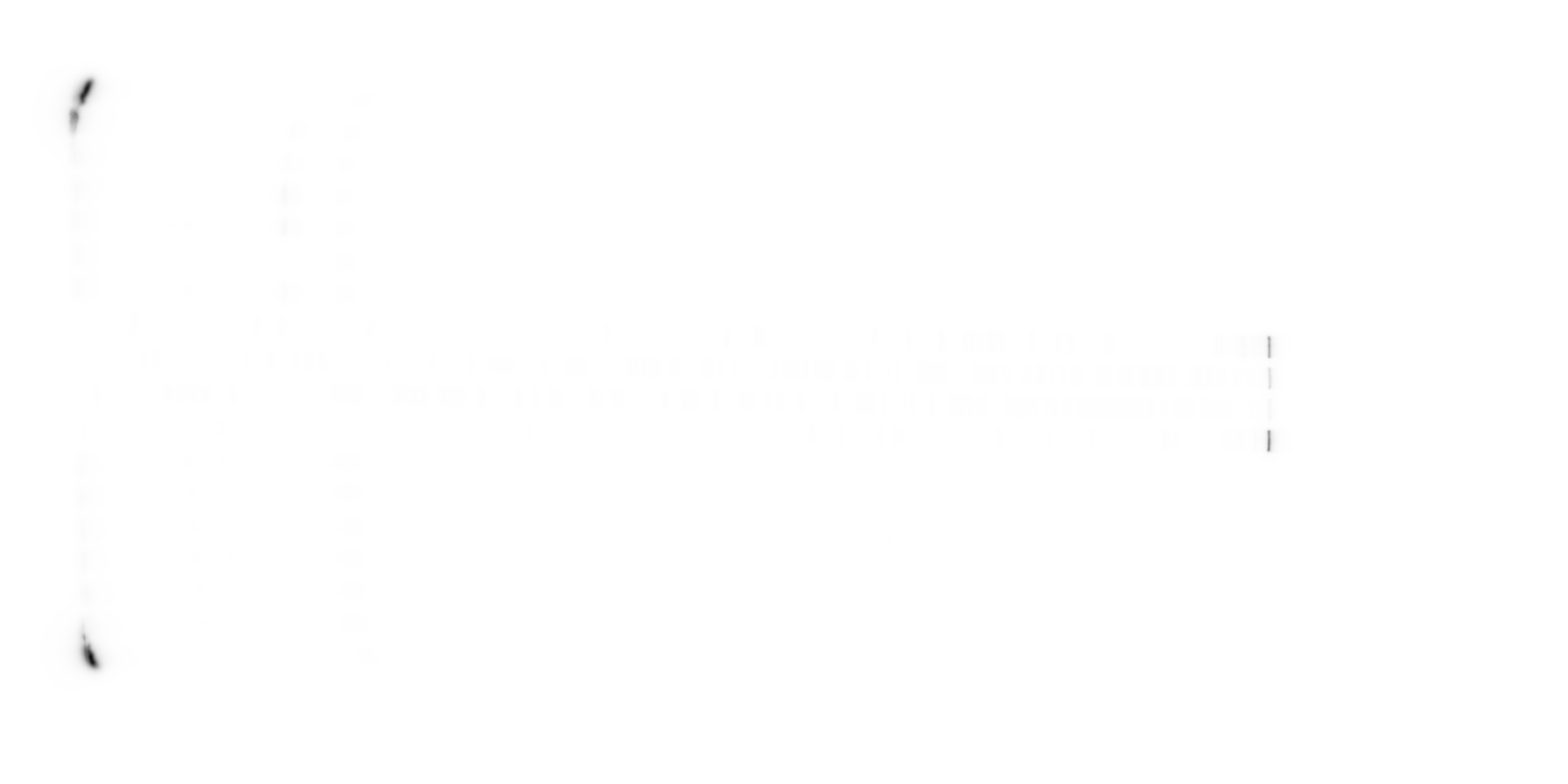

Supplement: Figure 3—source data 1. [file elife-69064-fig3-data1.zip › Source data - Figure 3/Figure 3C - 24.10.2017_primext_CSO-0185_gel2_1d-[Phosphor].tif]

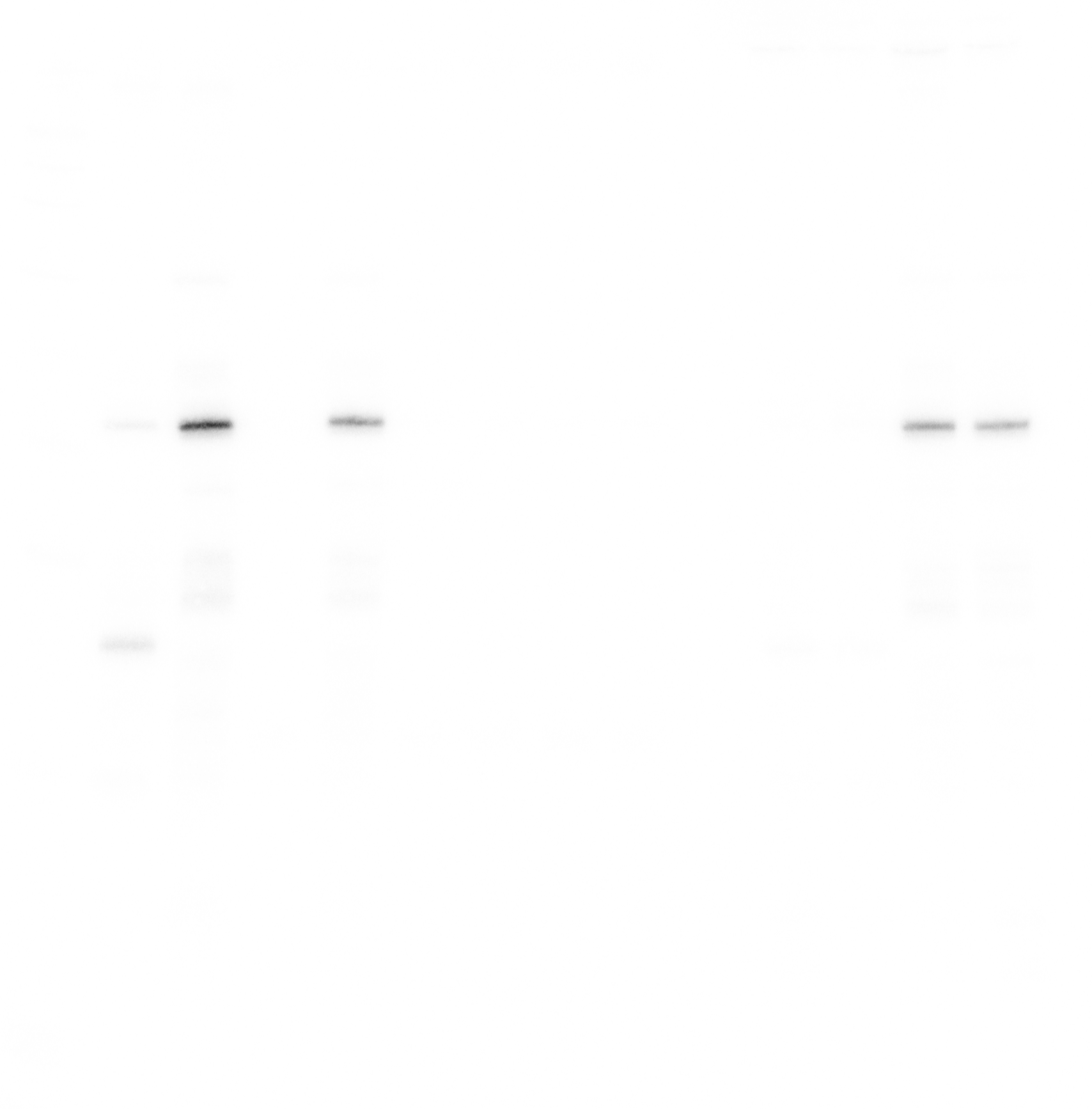

Supplement: Figure 3—source data 1. [file elife-69064-fig3-data1.zip › Source data - Figure 3/Figure 3D - 22012018_NB124_125_CSO-0189_6d-[Phosphor].tif]

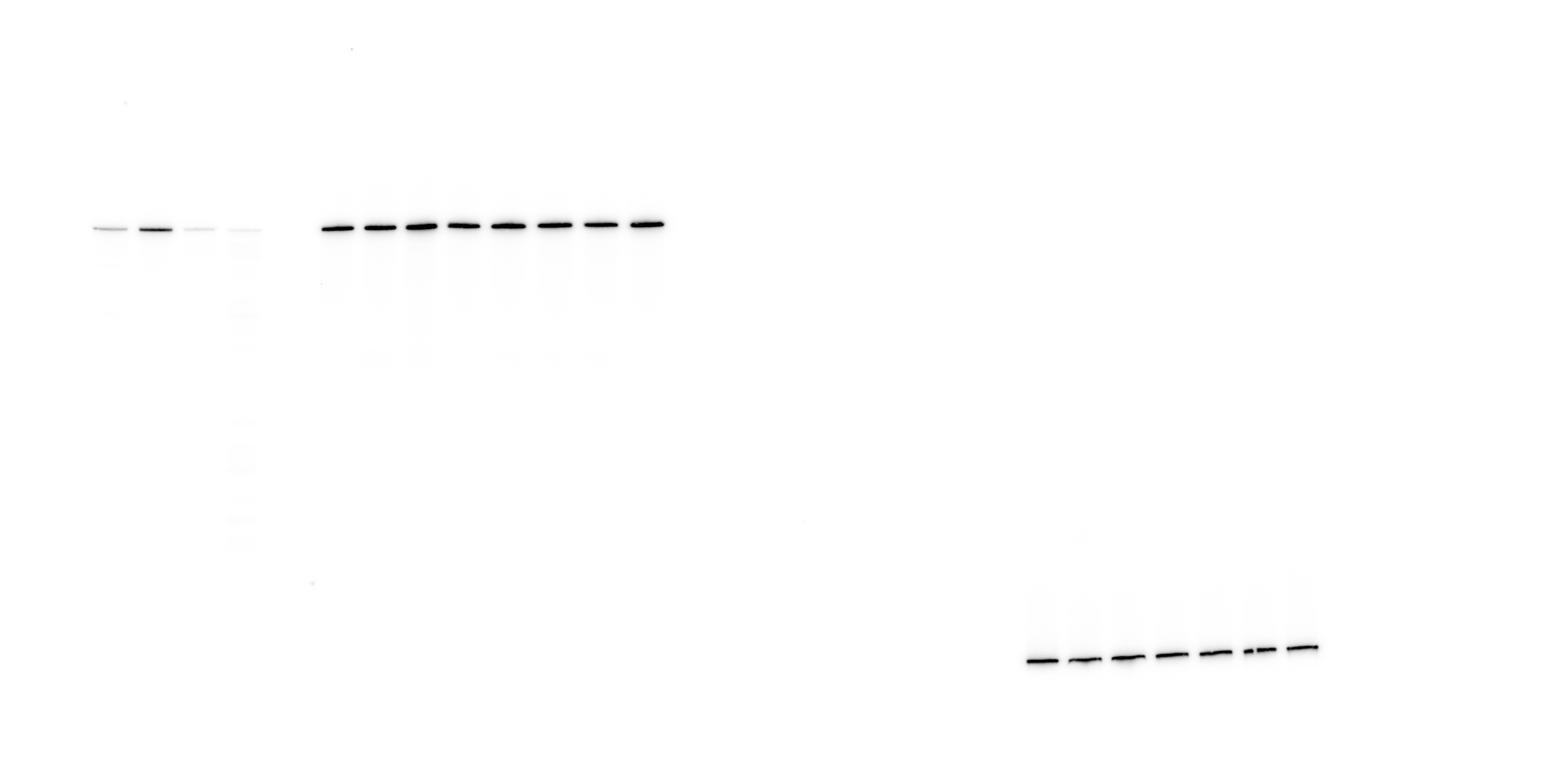

Supplement: Figure 3—source data 1. [file elife-69064-fig3-data1.zip › Source data - Figure 3/Figure 3D - 24.1.2018_NB124_125_CSO-0497_1d-[Phosphor].tif]

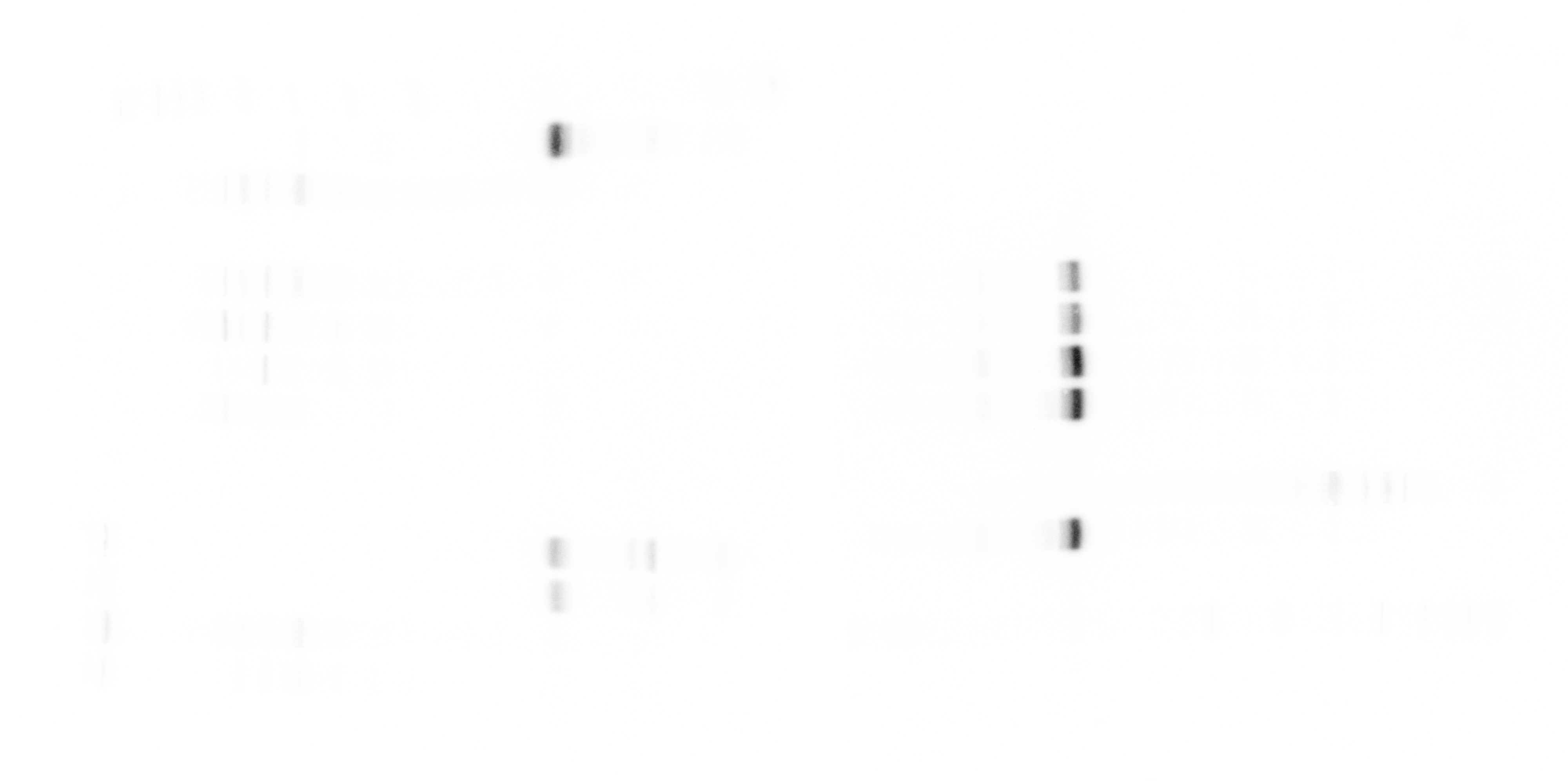

Supplement: Figure 3—source data 1. [file elife-69064-fig3-data1.zip › Source data - Figure 3/Figure 3D - 5.1.2018_NB124_125_CSO-0185_4d-[Phosphor].tif]

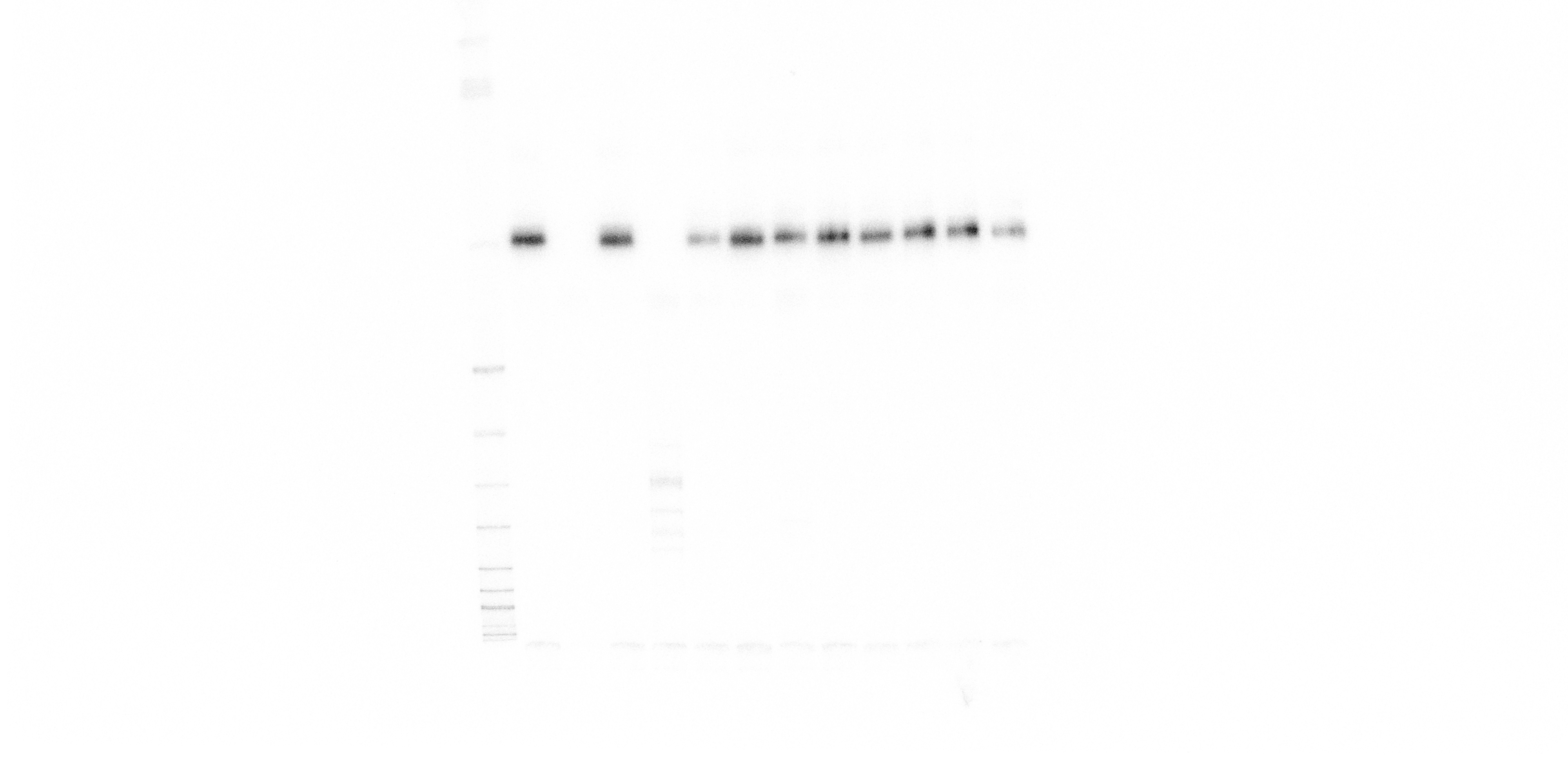

Supplement: Figure 3—figure supplement 1—source data 1. [file elife-69064-fig3-figsupp1-data1.zip › Source data - Figure 3 - figure supplement 1 - Source Data 1/Fig 3 - supp 1A - 11.1.2018_BA-NB88_CSO-0185_5d[Phosphor].tif]

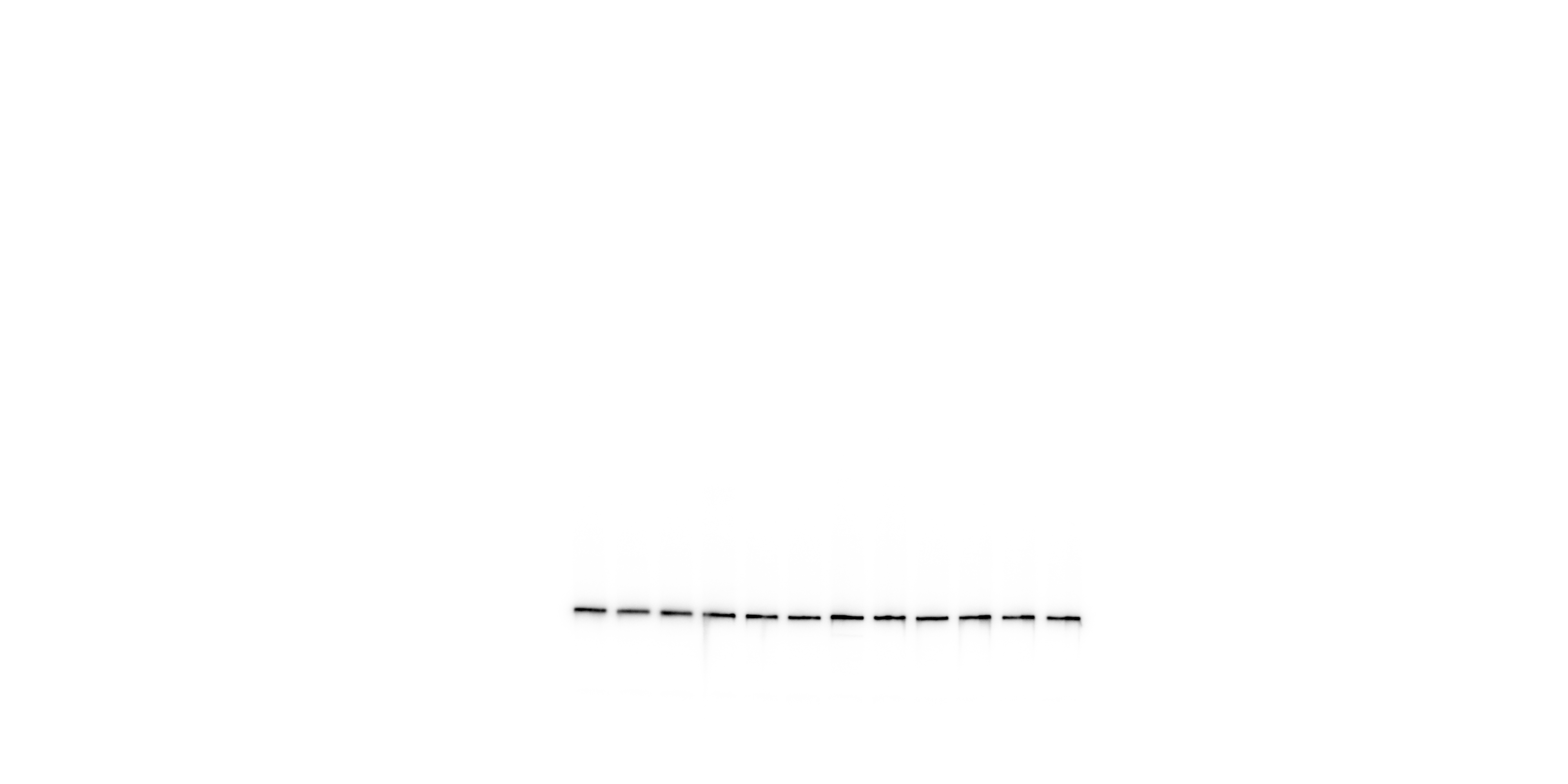

Supplement: Figure 3—figure supplement 1—source data 1. [file elife-69064-fig3-figsupp1-data1.zip › Source data - Figure 3 - figure supplement 1 - Source Data 1/Fig 3 - supp 1A - 19.2.2018_BANB88_CSO-0497_8h-[Phosphor].tif]

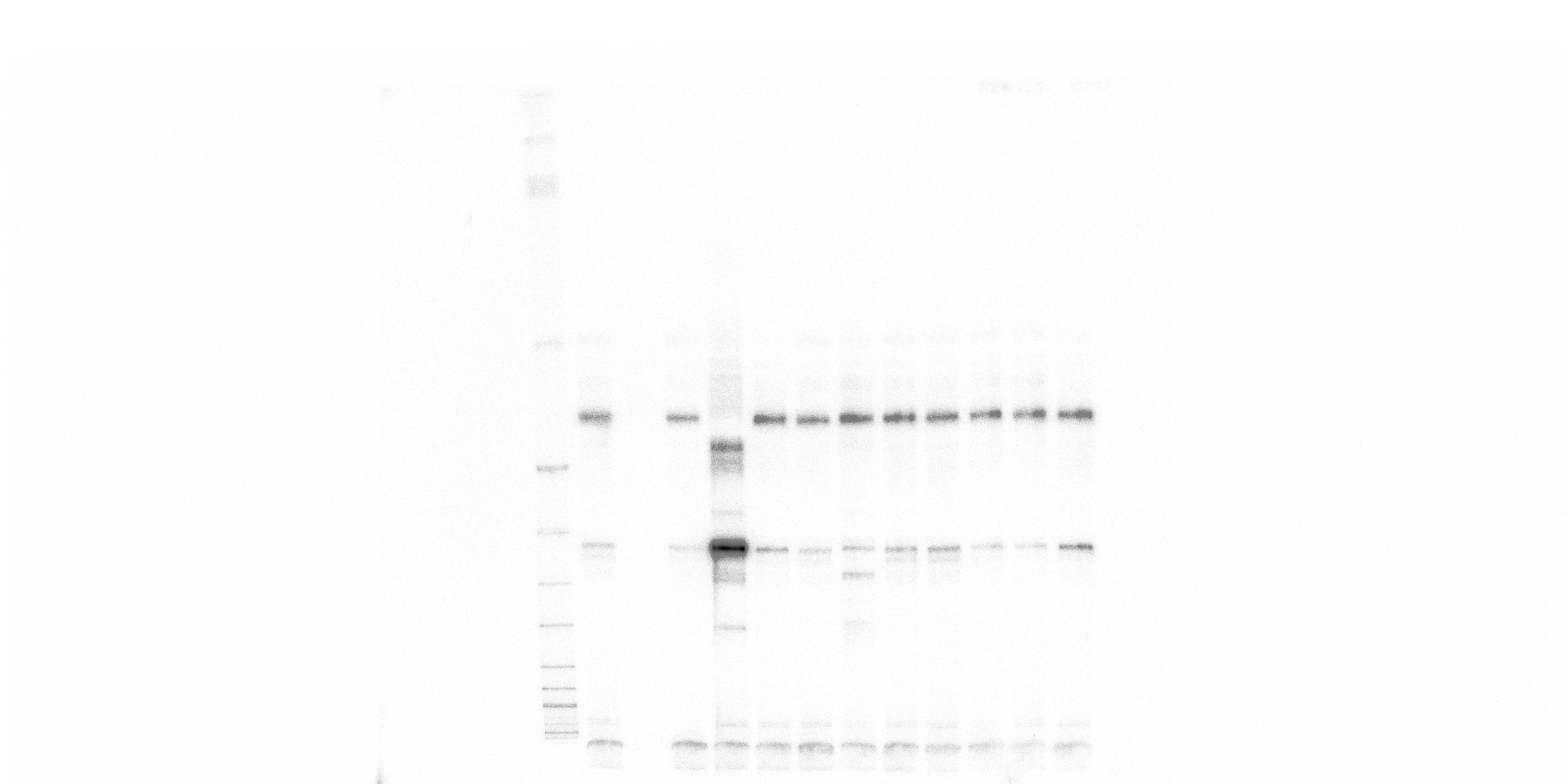

Supplement: Figure 3—figure supplement 1—source data 1. [file elife-69064-fig3-figsupp1-data1.zip › Source data - Figure 3 - figure supplement 1 - Source Data 1/Fig 3 - supp 1A - 20170117_NBBA88_CSO-0189_5d-[Phosphor].tif]

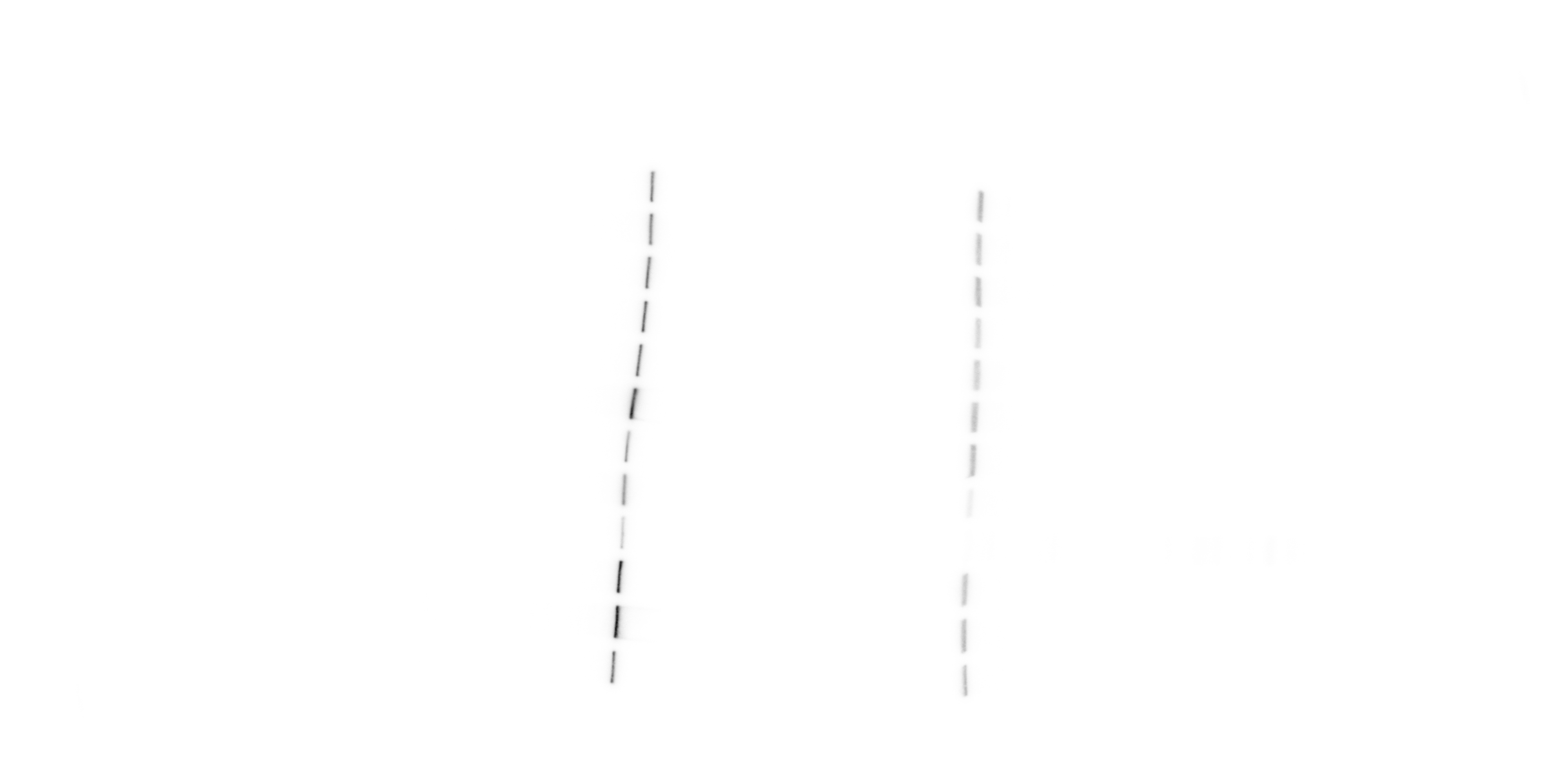

Supplement: Figure 3—figure supplement 1—source data 1. [file elife-69064-fig3-figsupp1-data1.zip › Source data - Figure 3 - figure supplement 1 - Source Data 1/Fig 3 - supp 1C - 12.2.2021_NB198_199_CSO-0497_1d-[Phosphor].tif]

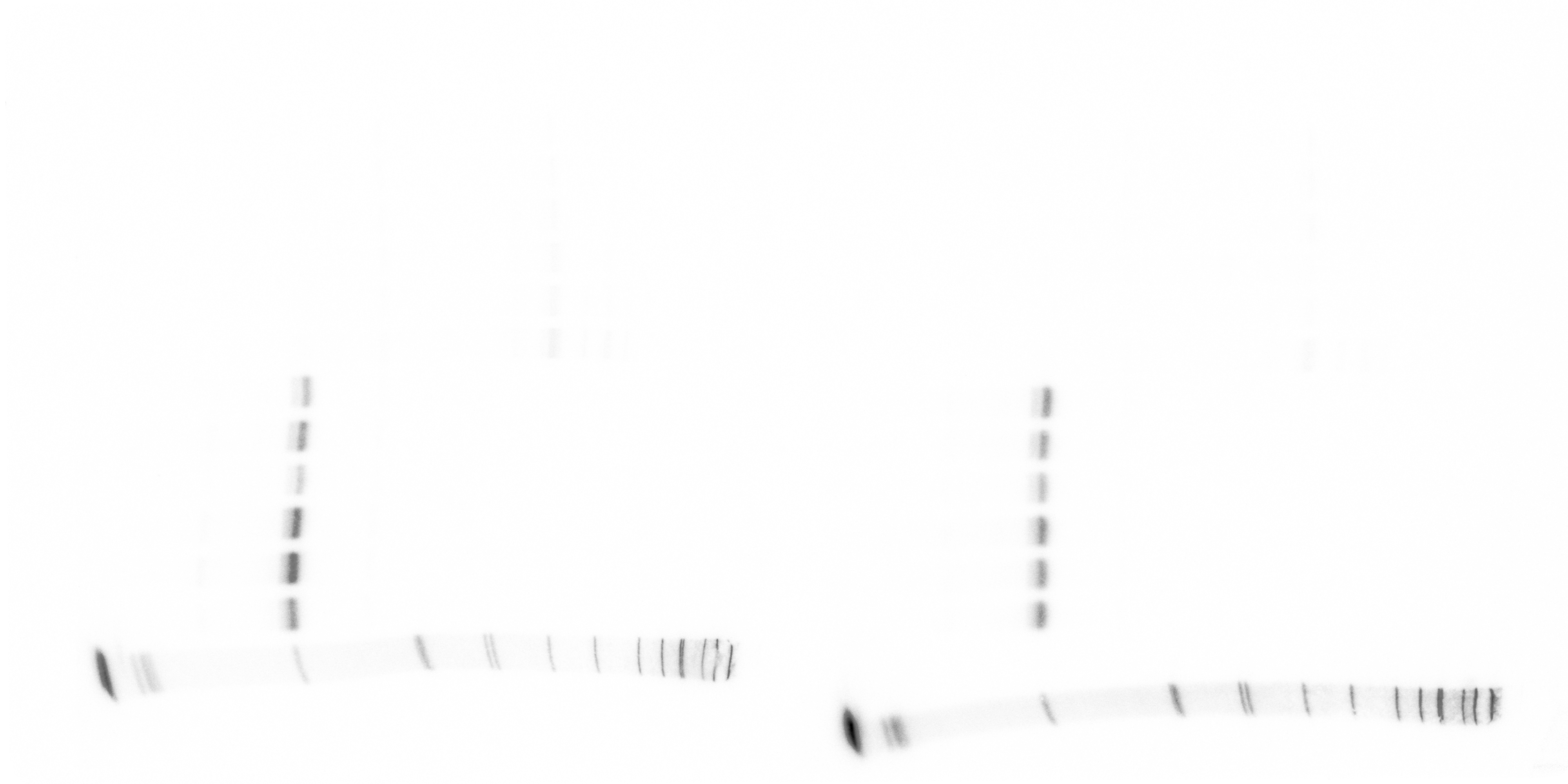

Supplement: Figure 3—figure supplement 1—source data 1. [file elife-69064-fig3-figsupp1-data1.zip › Source data - Figure 3 - figure supplement 1 - Source Data 1/Fig 3 - supp 1C - 22.1.2021_NB198_199_CSO-0185_14d-[Phosphor].tif]

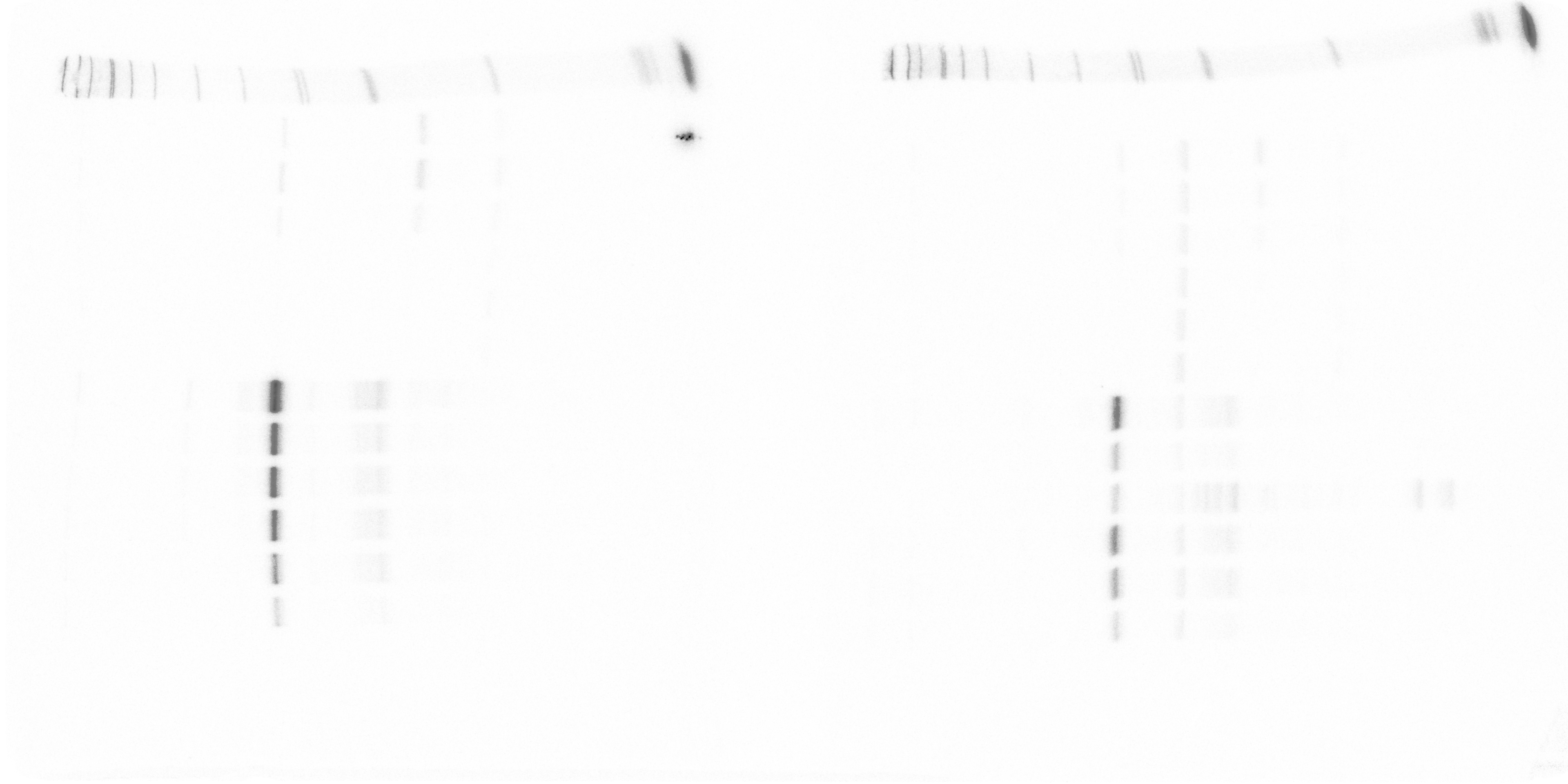

Supplement: Figure 3—figure supplement 1—source data 1. [file elife-69064-fig3-figsupp1-data1.zip › Source data - Figure 3 - figure supplement 1 - Source Data 1/Fig 3 - supp 1C - 5.1.2021_NB198_199_CSO-0189_9d-[Phosphor].tif]

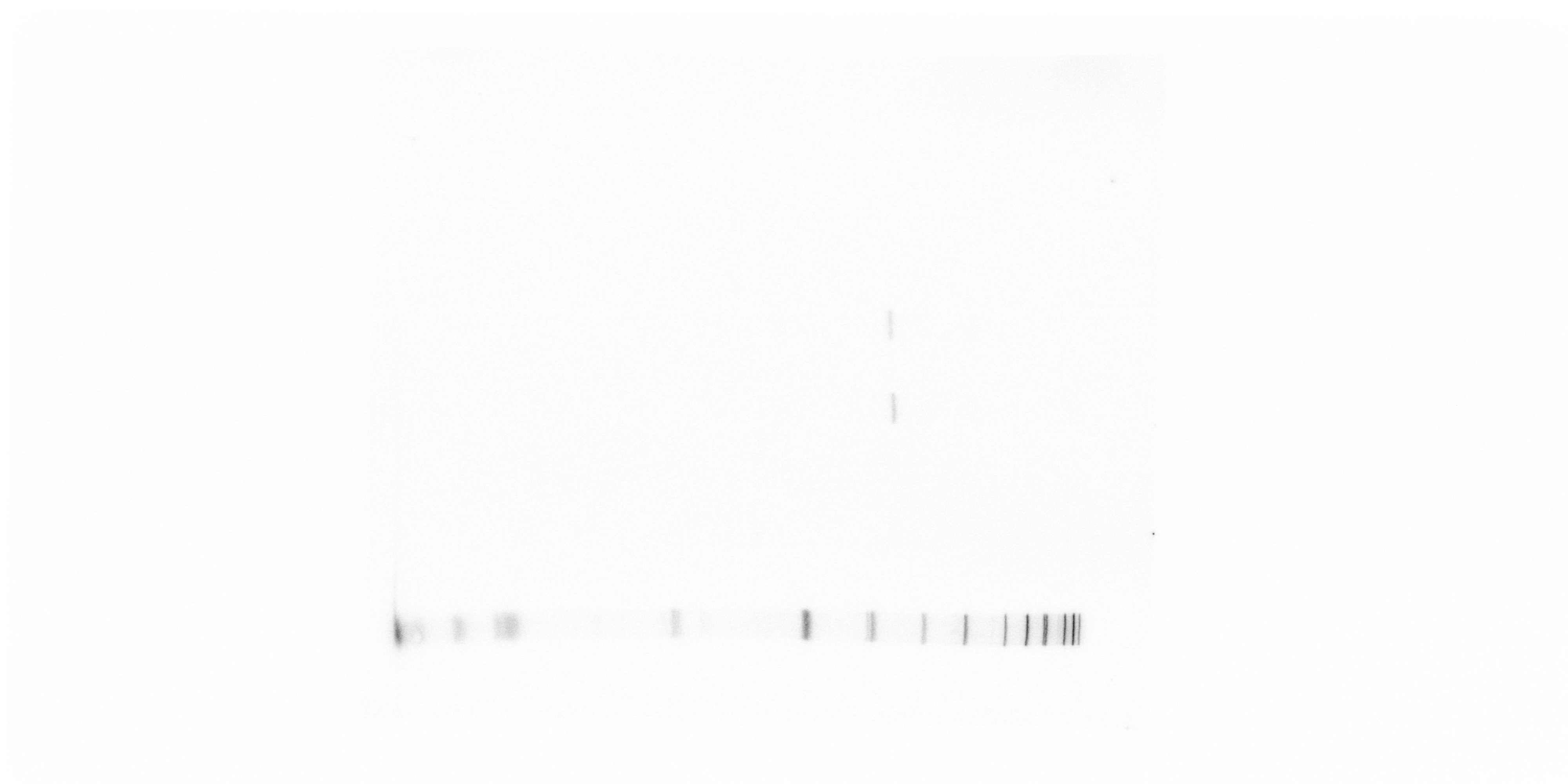

Supplement: Figure 3—figure supplement 2—source data 1. [file elife-69064-fig3-figsupp2-data1.zip › Source data - Figure 3 - figure supplement 2/Fig 3 - supp 2B - 5.1.2018_NB119_CSO-3095_9d-[Phosphor].tif]

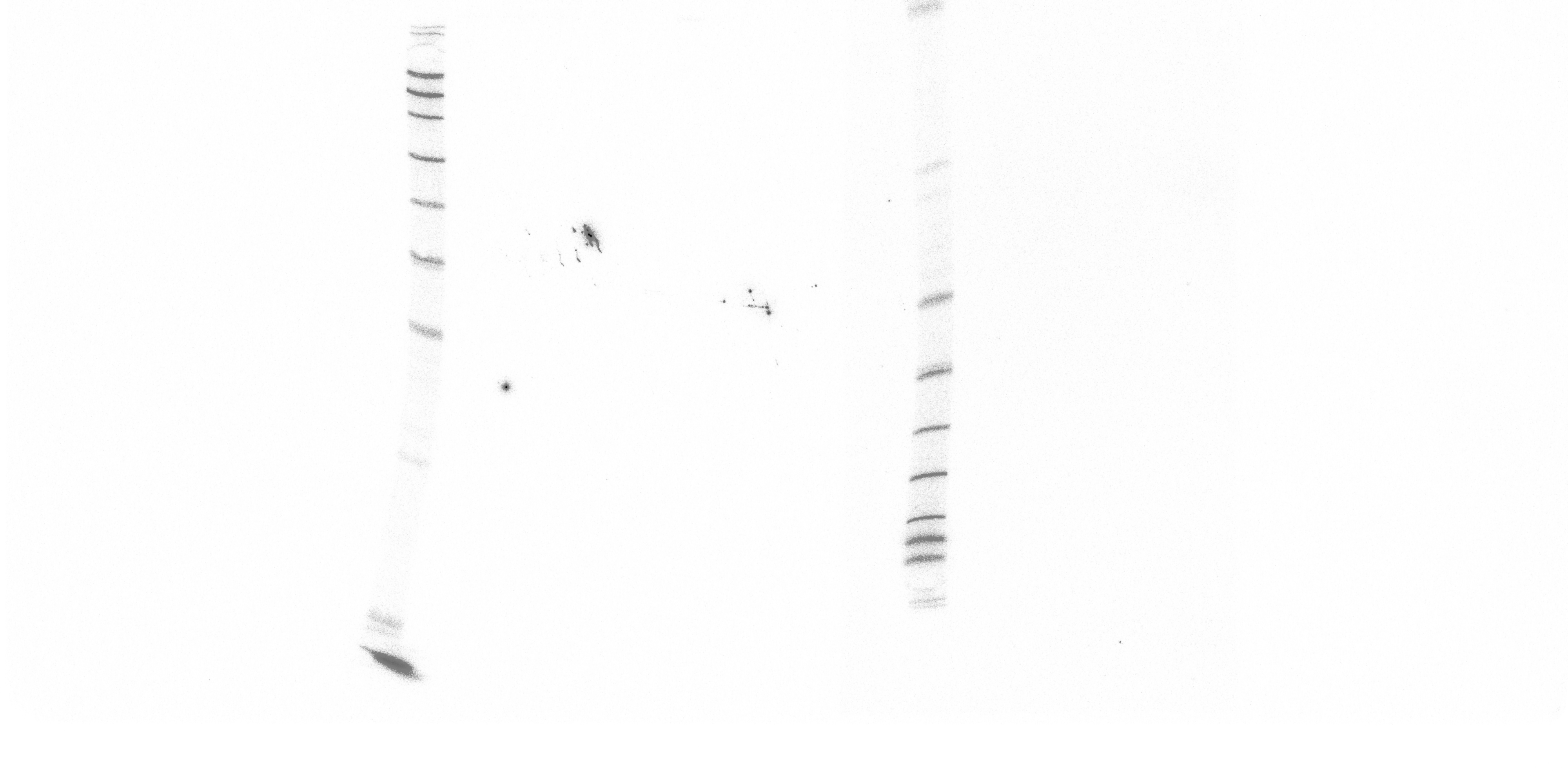

Supplement: Figure 3—figure supplement 2—source data 1. [file elife-69064-fig3-figsupp2-data1.zip › Source data - Figure 3 - figure supplement 2/Fig 3 - supp 2C - 1.3.2021_NB207_208_CSO-5476_1970_3d-[Phosphor].tif]

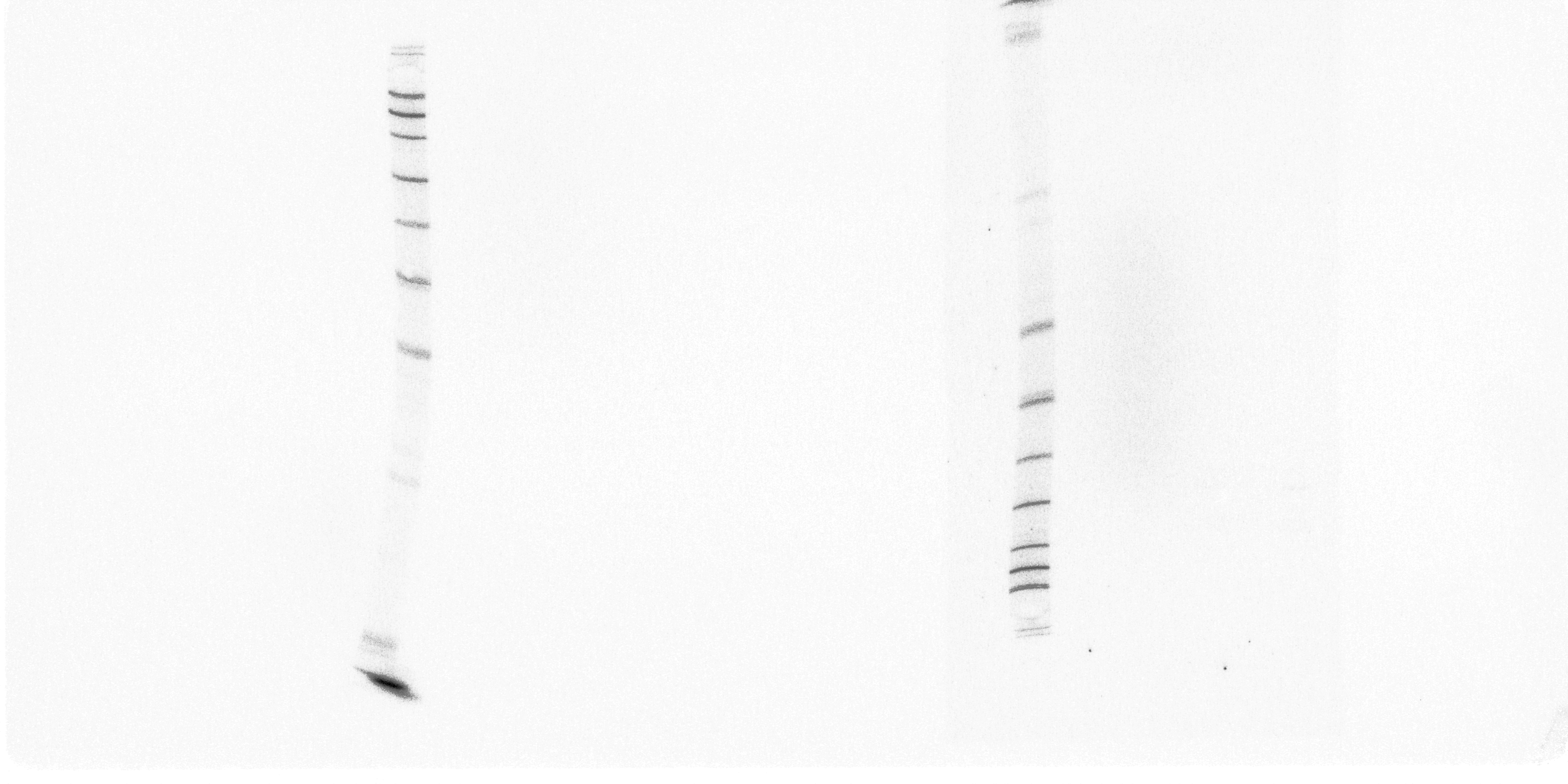

Supplement: Figure 3—figure supplement 2—source data 1. [file elife-69064-fig3-figsupp2-data1.zip › Source data - Figure 3 - figure supplement 2/Fig 3 - supp 2C - 10.3.2021_NB207_5476_NB208_1967-[Phosphor].tif]

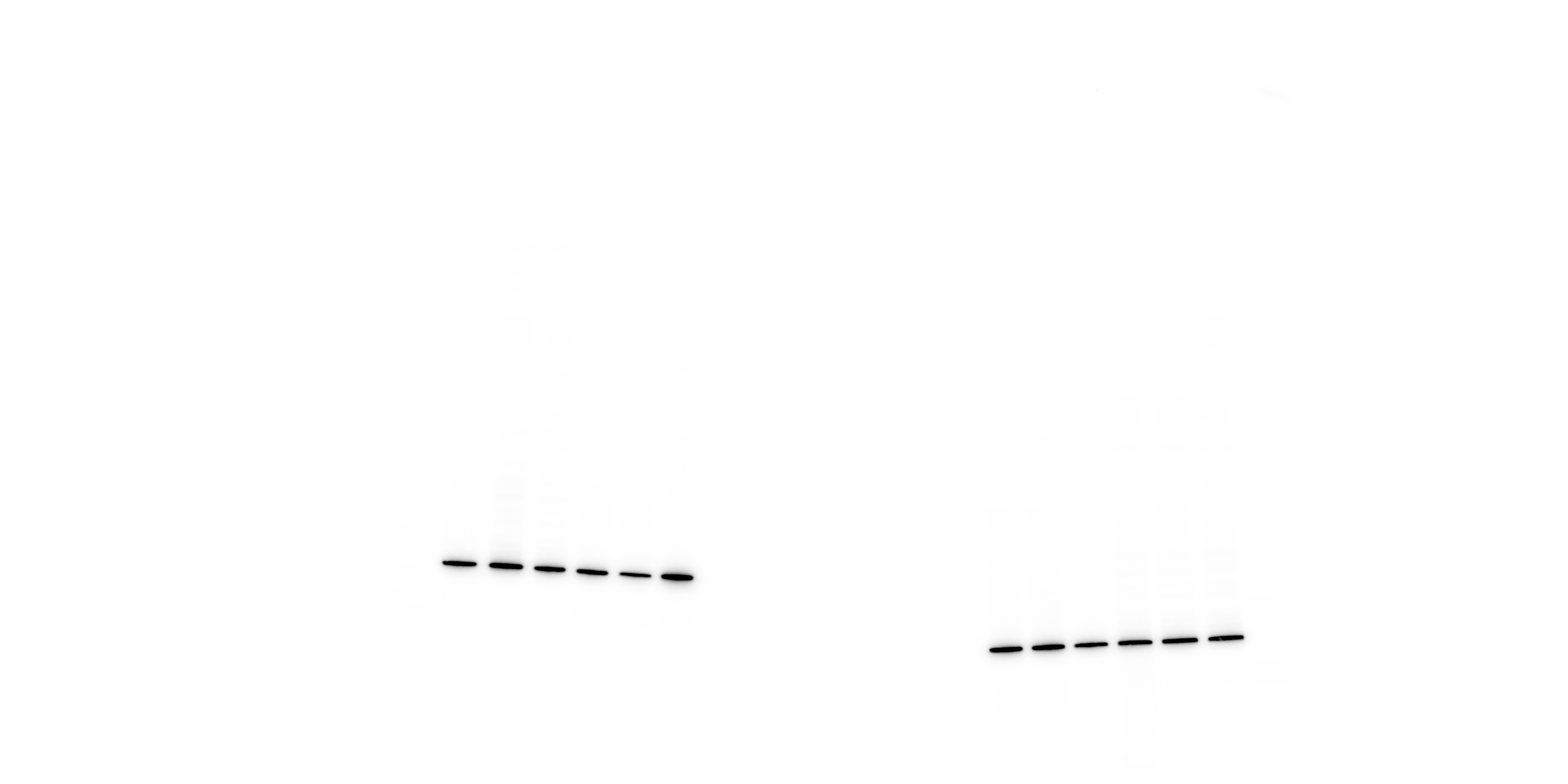

Supplement: Figure 3—figure supplement 2—source data 1. [file elife-69064-fig3-figsupp2-data1.zip › Source data - Figure 3 - figure supplement 2/Fig 3 - supp 2C - 12.3.2021_NB207_208_CSO-0497_1d-[Phosphor].tif]

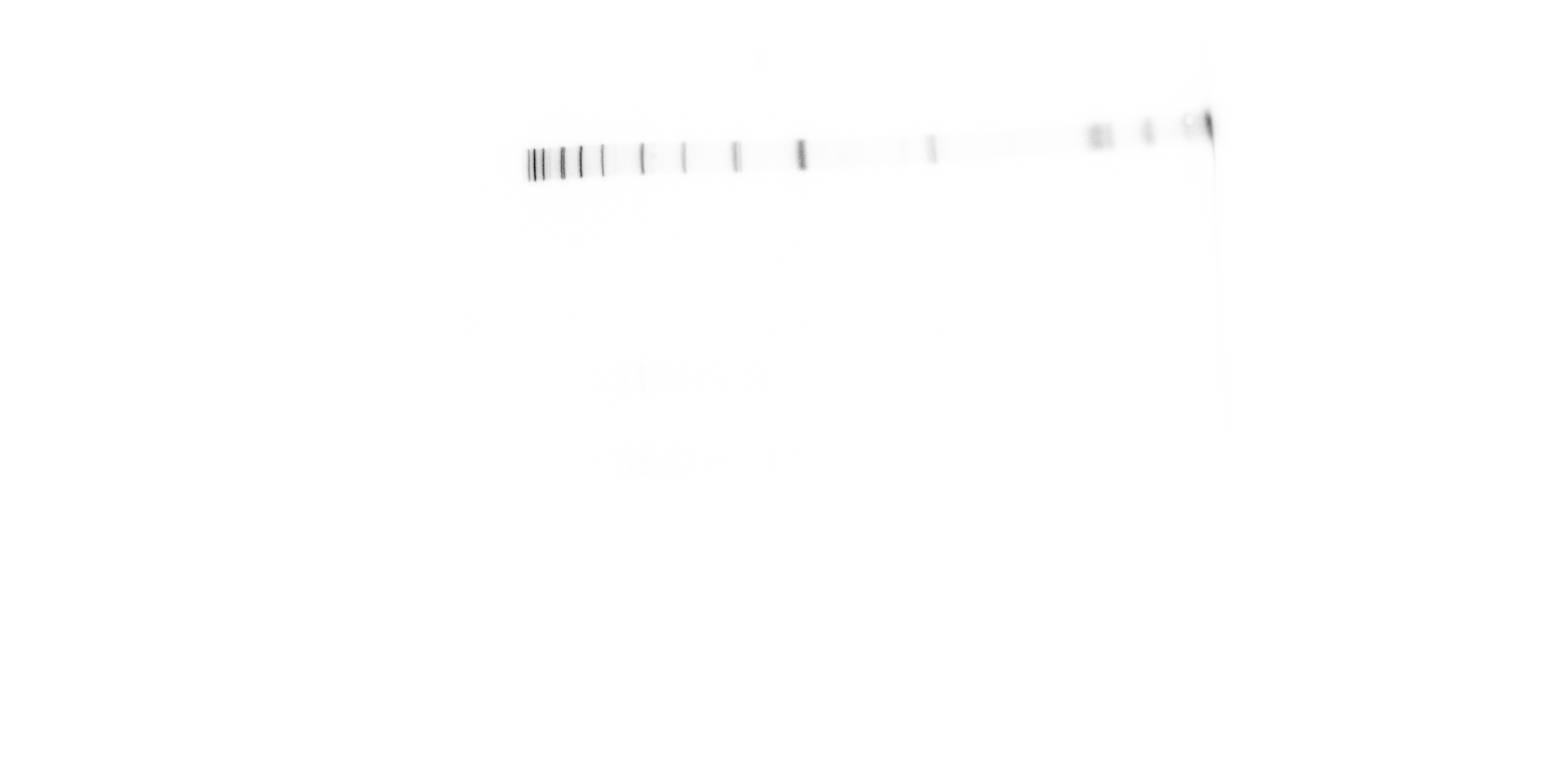

Supplement: Figure 3—figure supplement 2—source data 1. [file elife-69064-fig3-figsupp2-data1.zip › Source data - Figure 3 - figure supplement 2/Fig 3 - supp 2C - 2017111_SSvNB119_CSO_0184_6d-[Phosphor].tif]

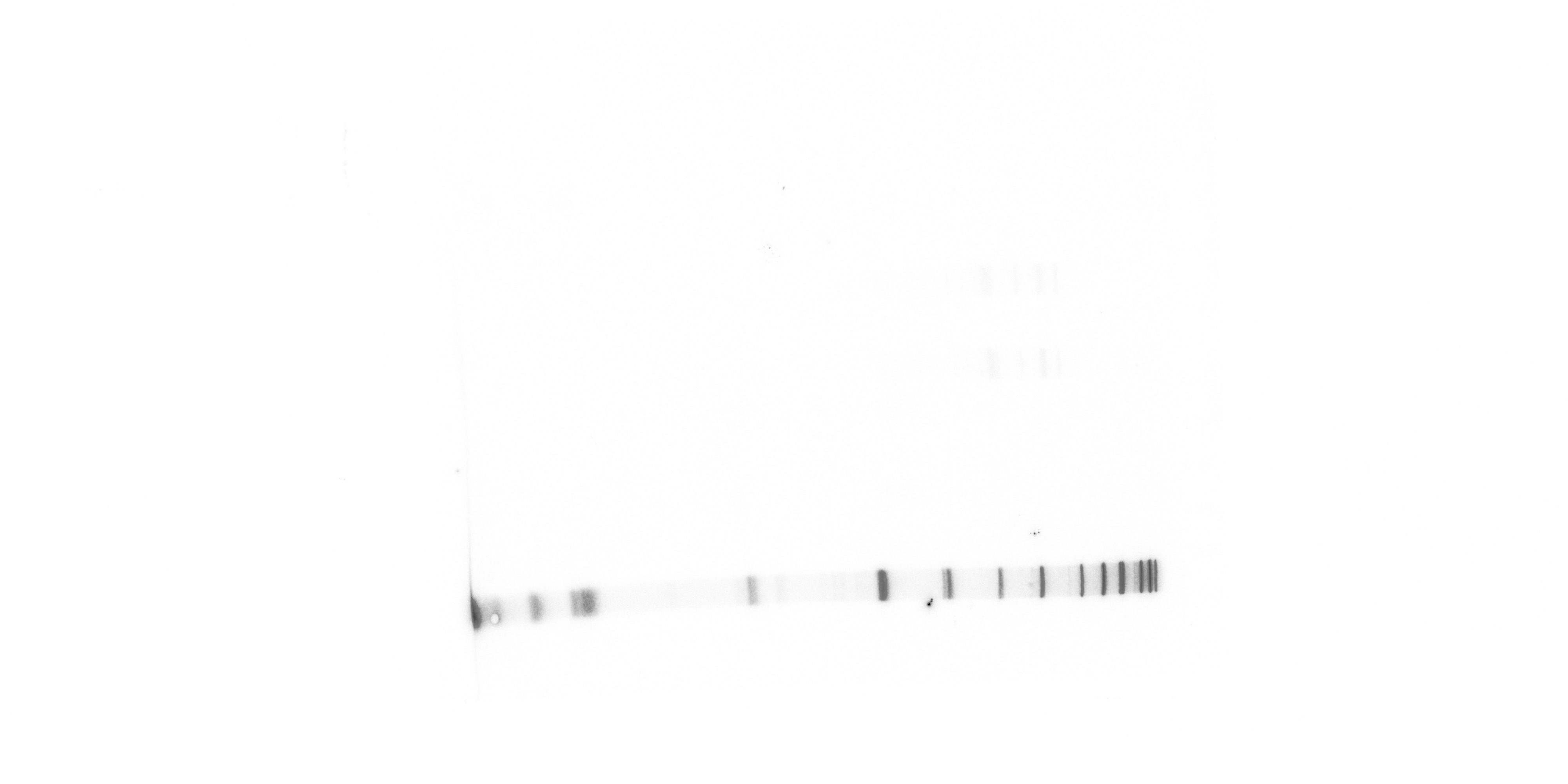

Supplement: Figure 3—figure supplement 2—source data 1. [file elife-69064-fig3-figsupp2-data1.zip › Source data - Figure 3 - figure supplement 2/Fig 3 - supp 2C - 22.11.2017_NB119_CSO-2159_5d-[Phosphor].tif]

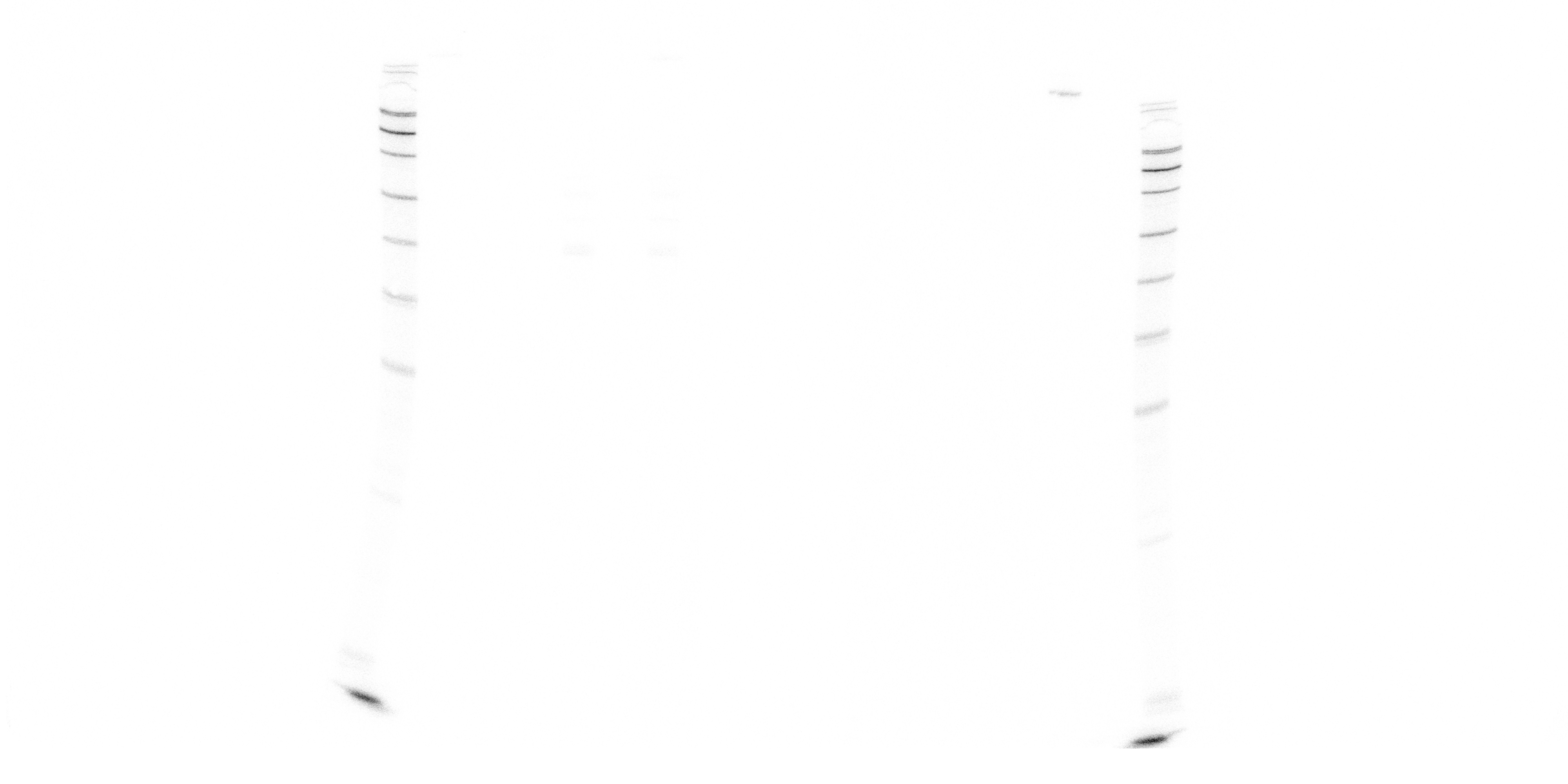

Supplement: Figure 3—figure supplement 2—source data 1. [file elife-69064-fig3-figsupp2-data1.zip › Source data - Figure 3 - figure supplement 2/Fig 3 - supp 2C - 25.2.2021_NB20_208_CSO-5436_5437_4d-[Phosphor].tif]

**Source data for Figure 3 – Figure supplement 2**

**Panel B**

NB119


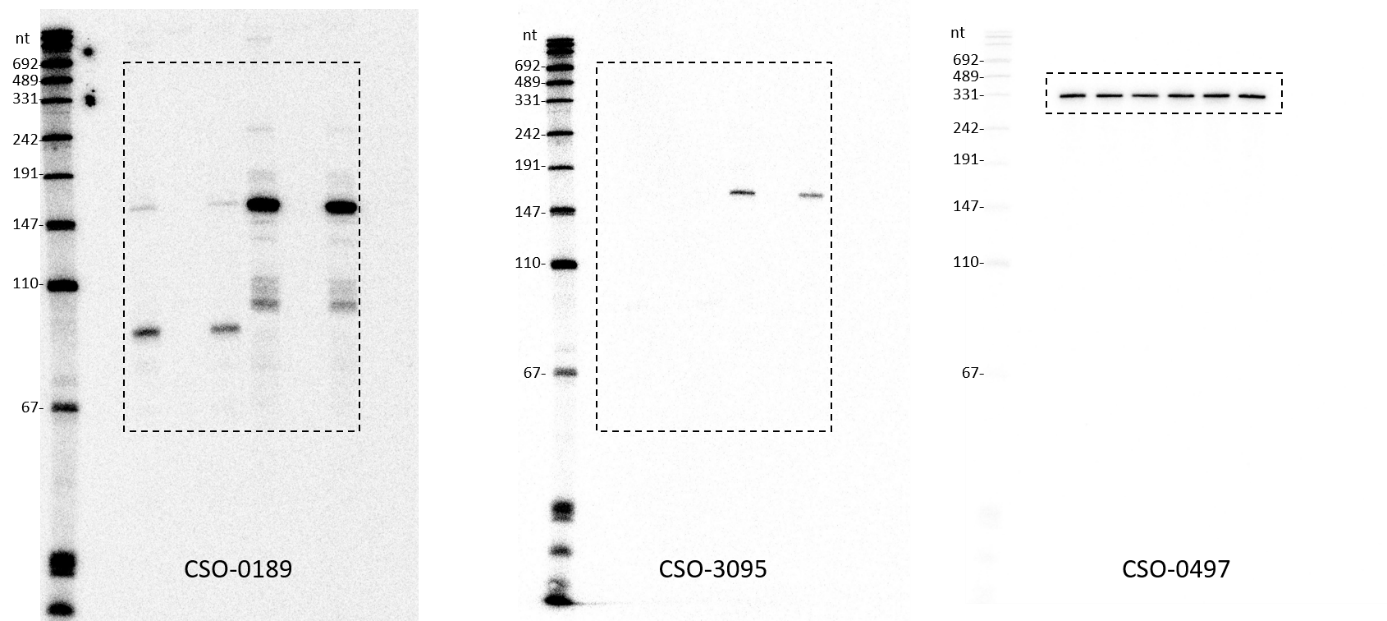


**Panel C**

NB207

**
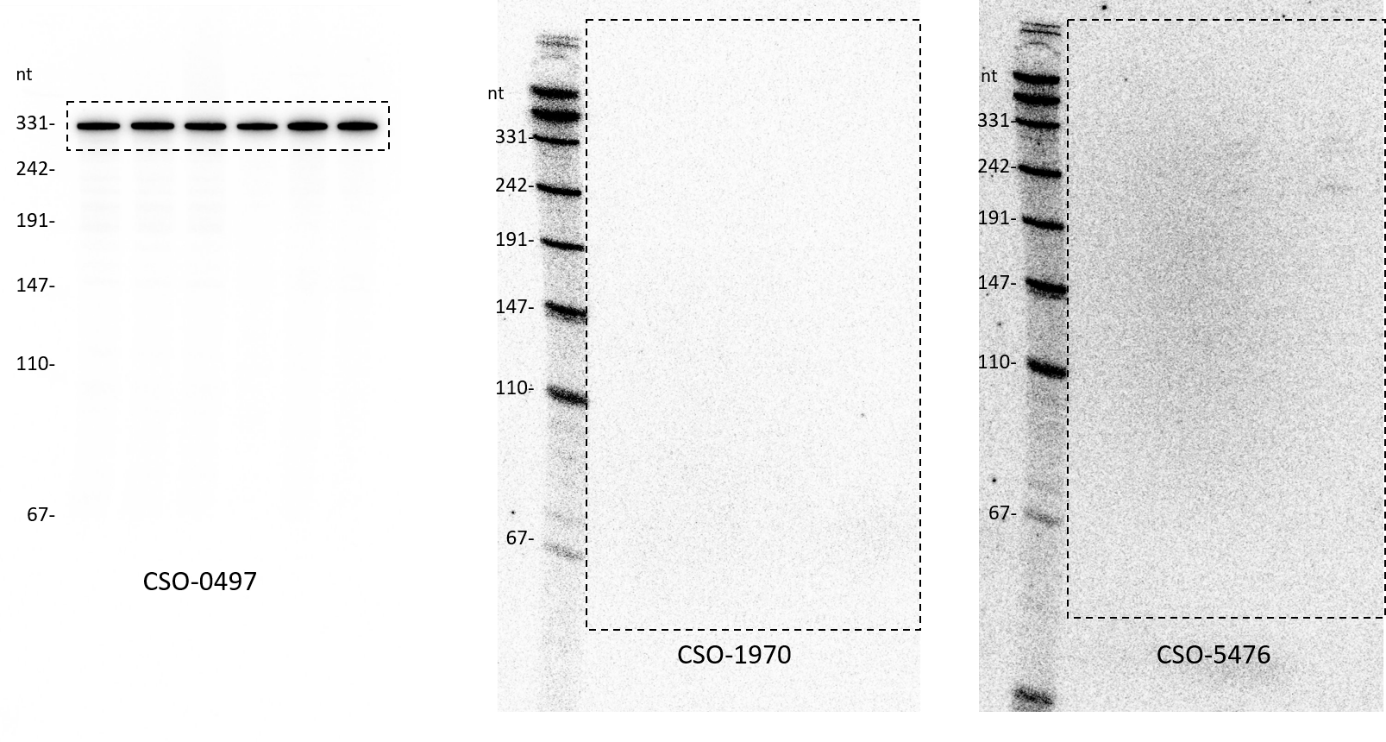
**

**NB208**

**
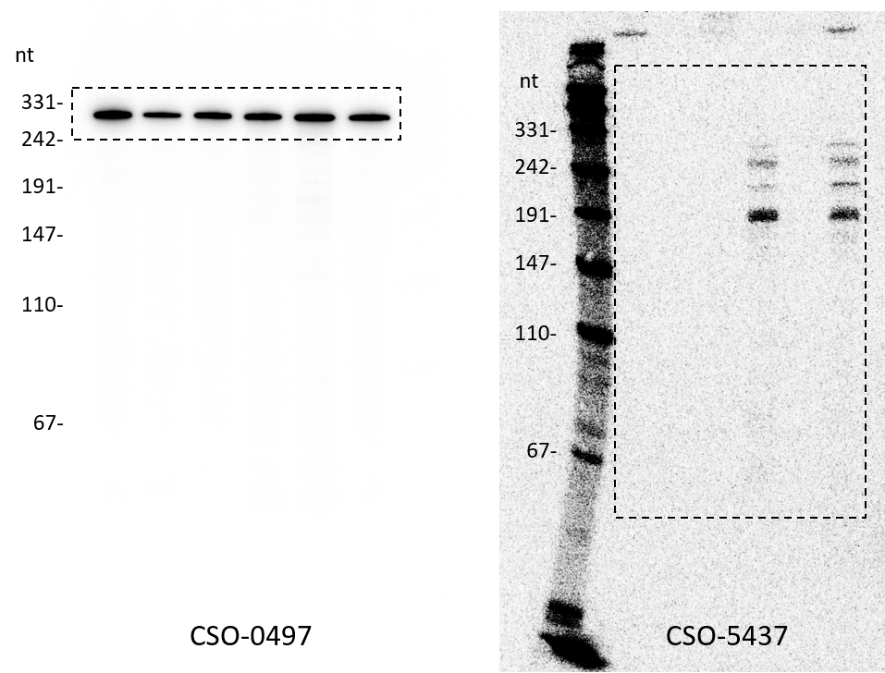
**

NB119

***
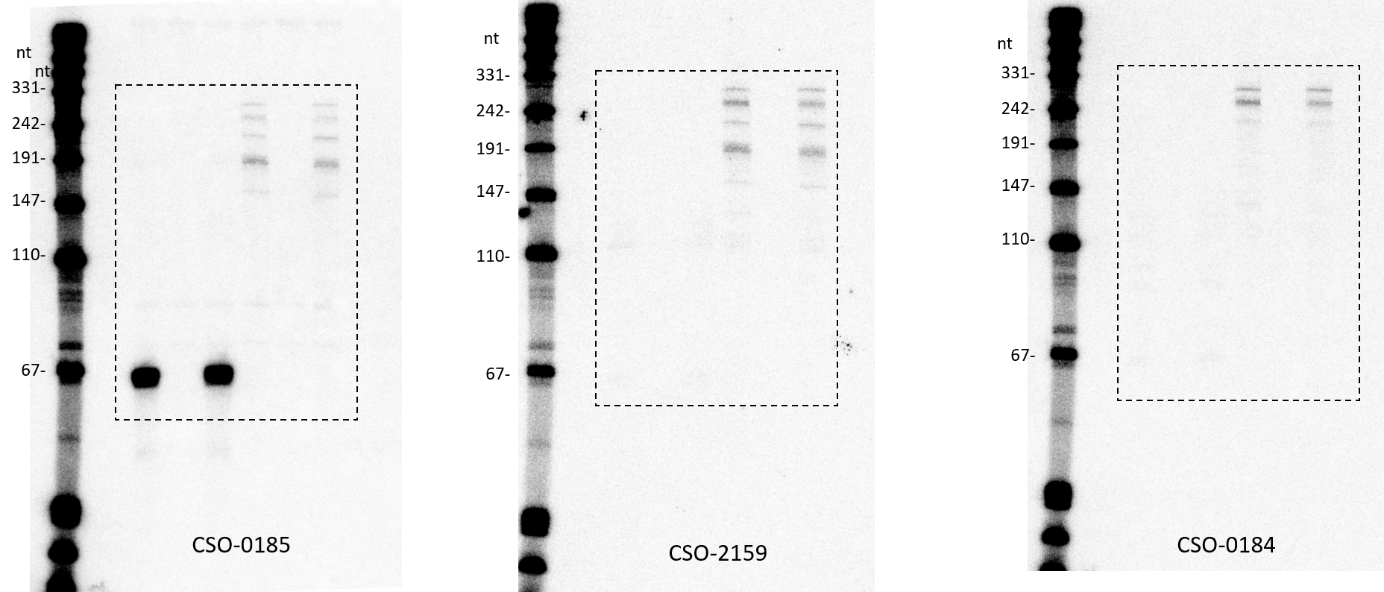
***

Supplement: Figure 3—figure supplement 2—source data 1. [file elife-69064-fig3-figsupp2-data1.zip › Source data - Figure 3 - figure supplement 2/Source data - Figure 3 - Figure supplement 2.docx]

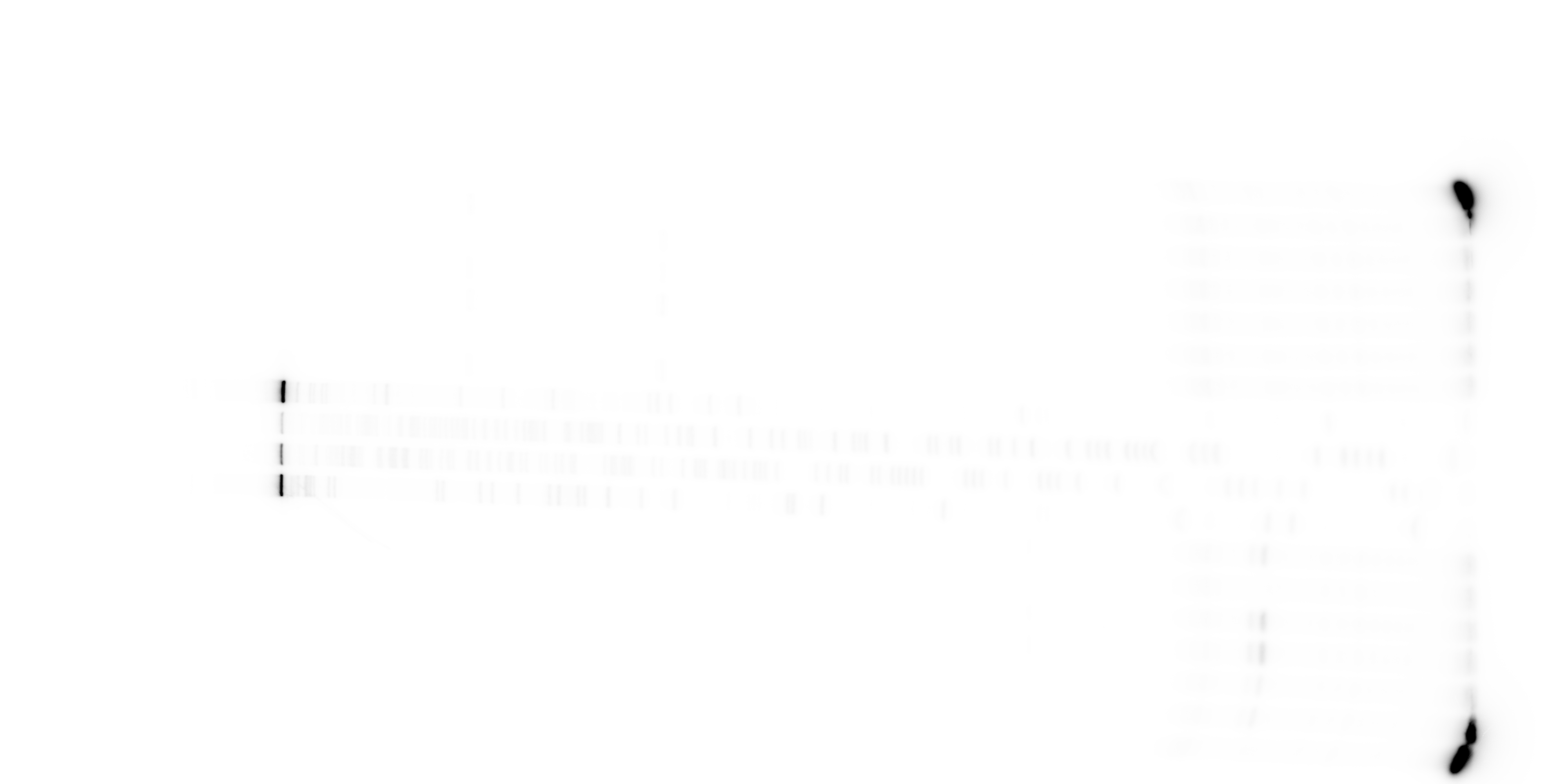

Supplement: Figure 3—figure supplement 3—source data 1. [file elife-69064-fig3-figsupp3-data1.zip › Source data - Figure 3 - figure supplement 3/Fig 3 - supp 3AB - 30.10.2017_primext_CSO-0185_R1_gel2_6d-[Phosphor].tif]

**Source data Figure 3 – figure supplement 3**

**Panels A & B**


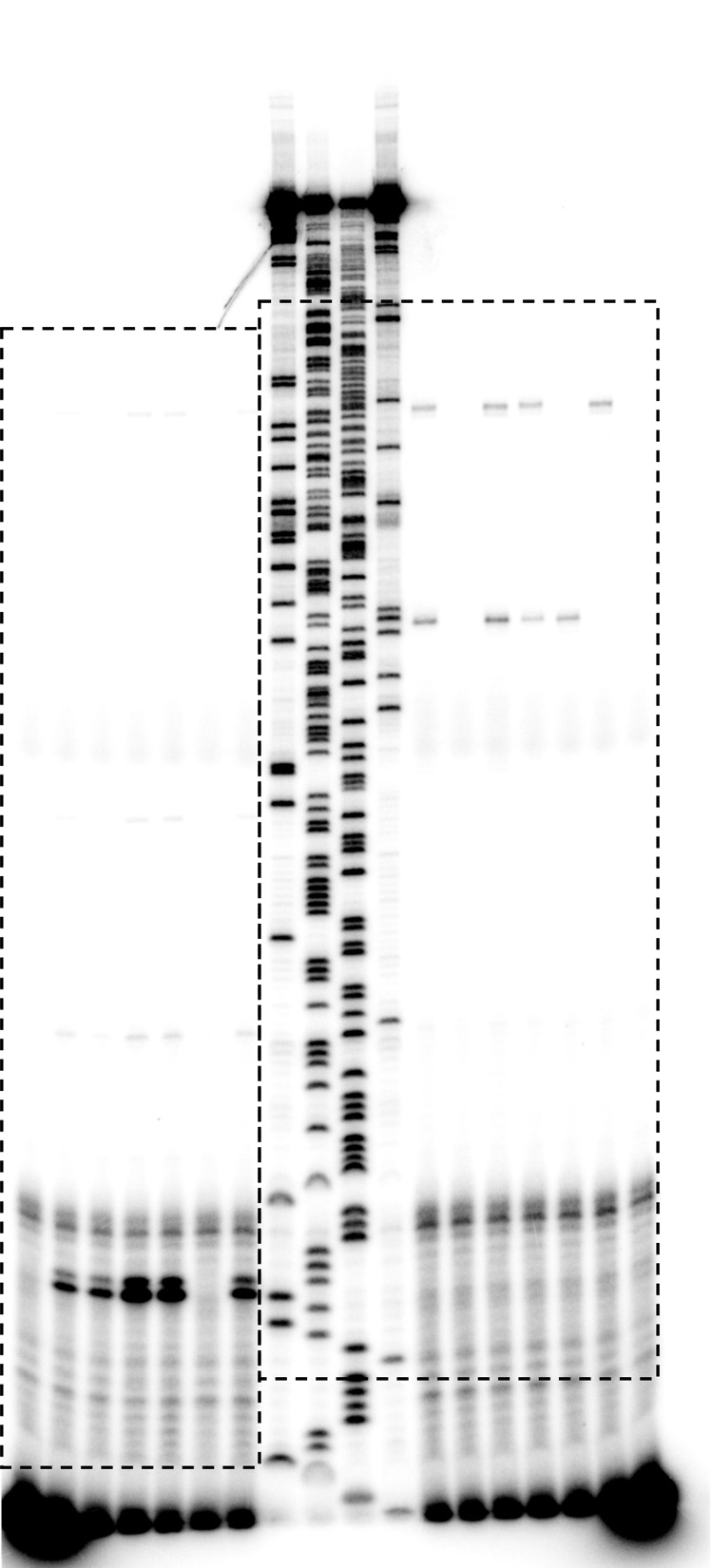

Supplement: Figure 3—figure supplement 3—source data 1. [file elife-69064-fig3-figsupp3-data1.zip › Source data - Figure 3 - figure supplement 3/Source data - Figure 3 - Figure supplement 3.docx]

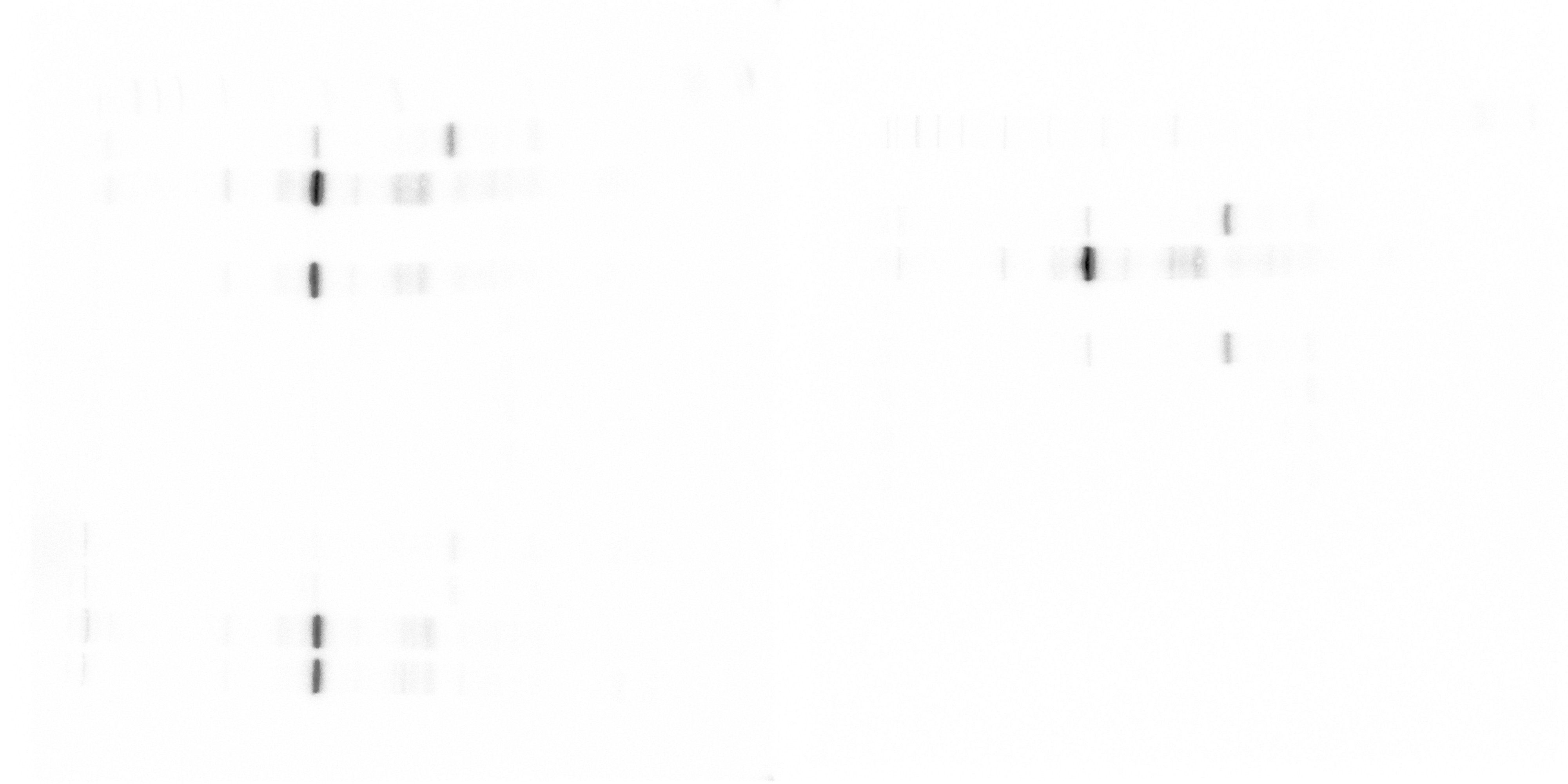

Supplement: Figure 4—source data 1. [file elife-69064-fig4-data1.zip › Source data - Figure 4/Figure 4A - 22012018_NB124_125_CSO-0189_6d-[Phosphor].tif]

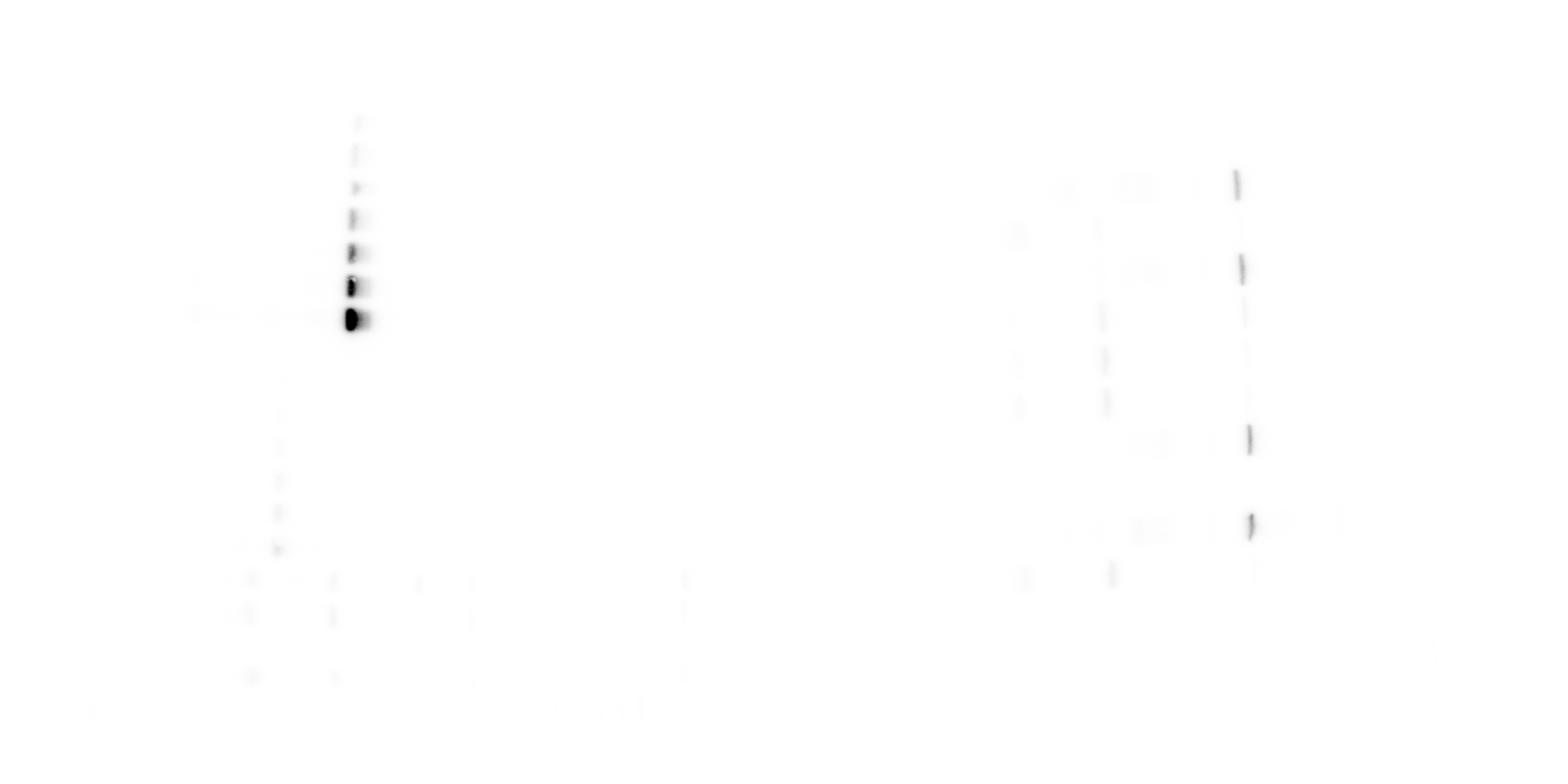

Supplement: Figure 4—source data 1. [file elife-69064-fig4-data1.zip › Source data - Figure 4/Figure 4B - 15.1.2018_NB126_127_CSO-0189_4d-[Phosphor].tif]

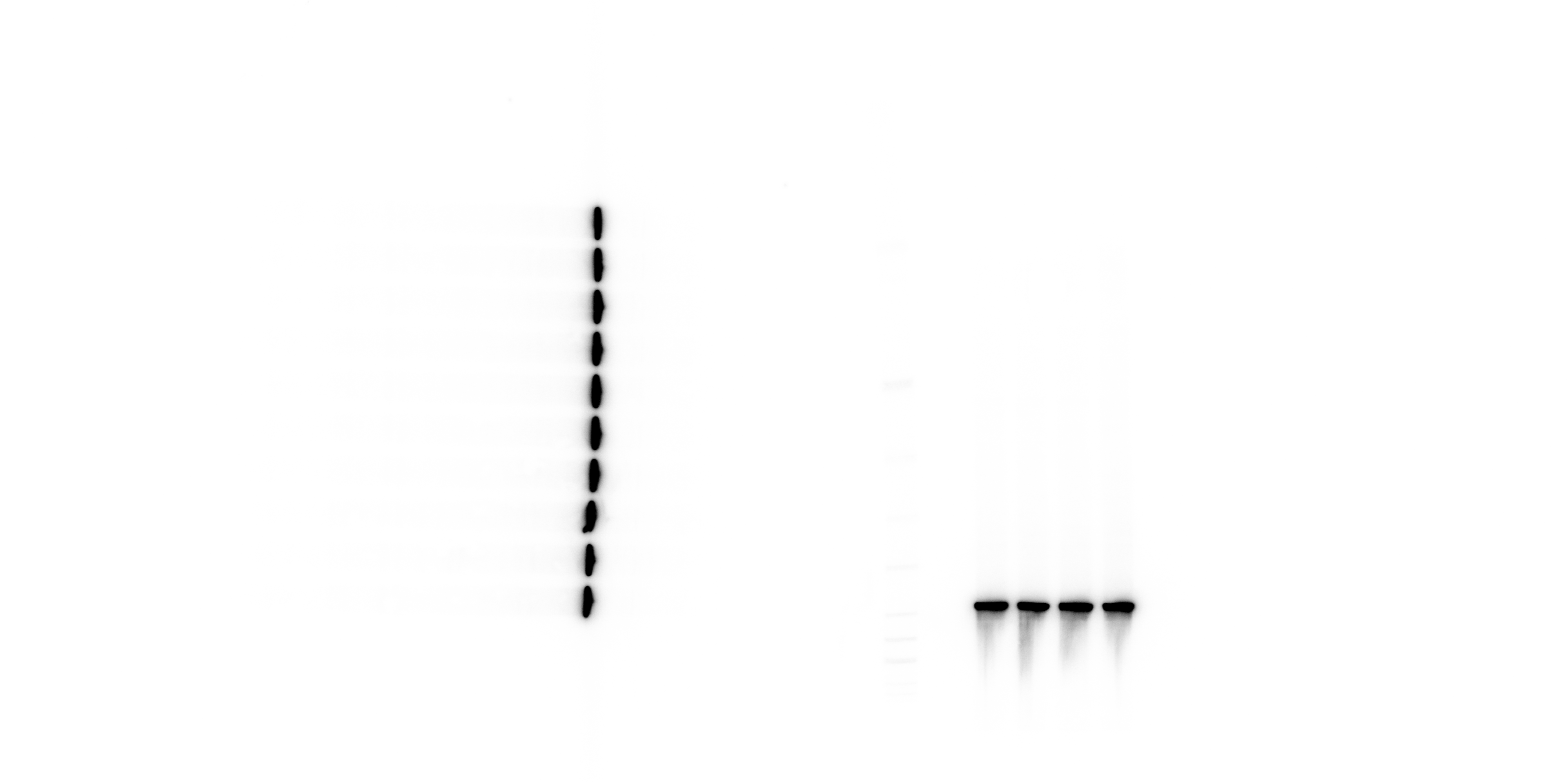

Supplement: Figure 4—source data 1. [file elife-69064-fig4-data1.zip › Source data - Figure 4/Figure 4B - 20180129_NB126_128_CSO-0497_2d-[Phosphor].tif]

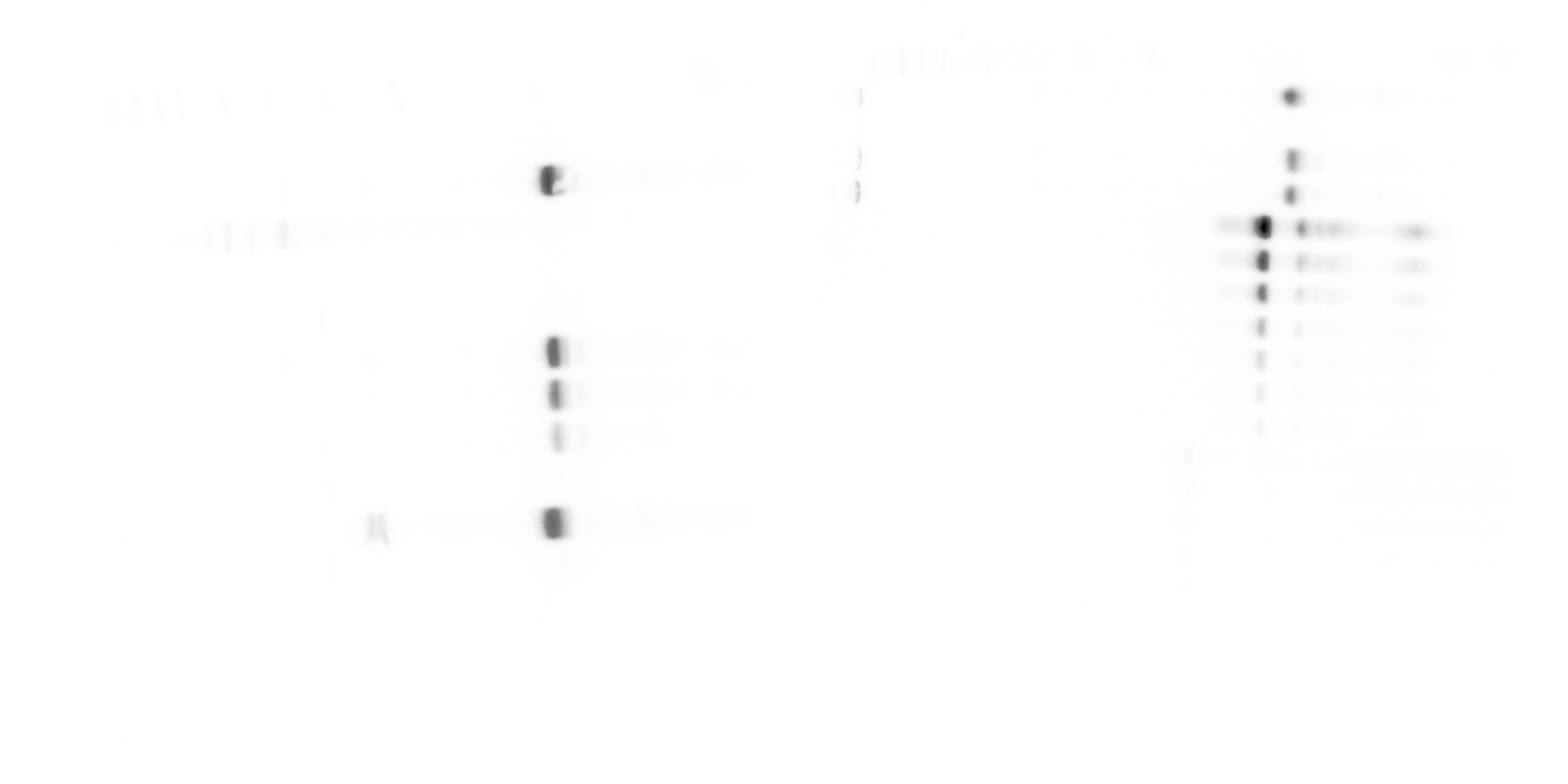

Supplement: Figure 4—source data 1. [file elife-69064-fig4-data1.zip › Source data - Figure 4/Figure 4B - 22012018_NB126_127_CSO-0185_6d-[Phosphor].tif]

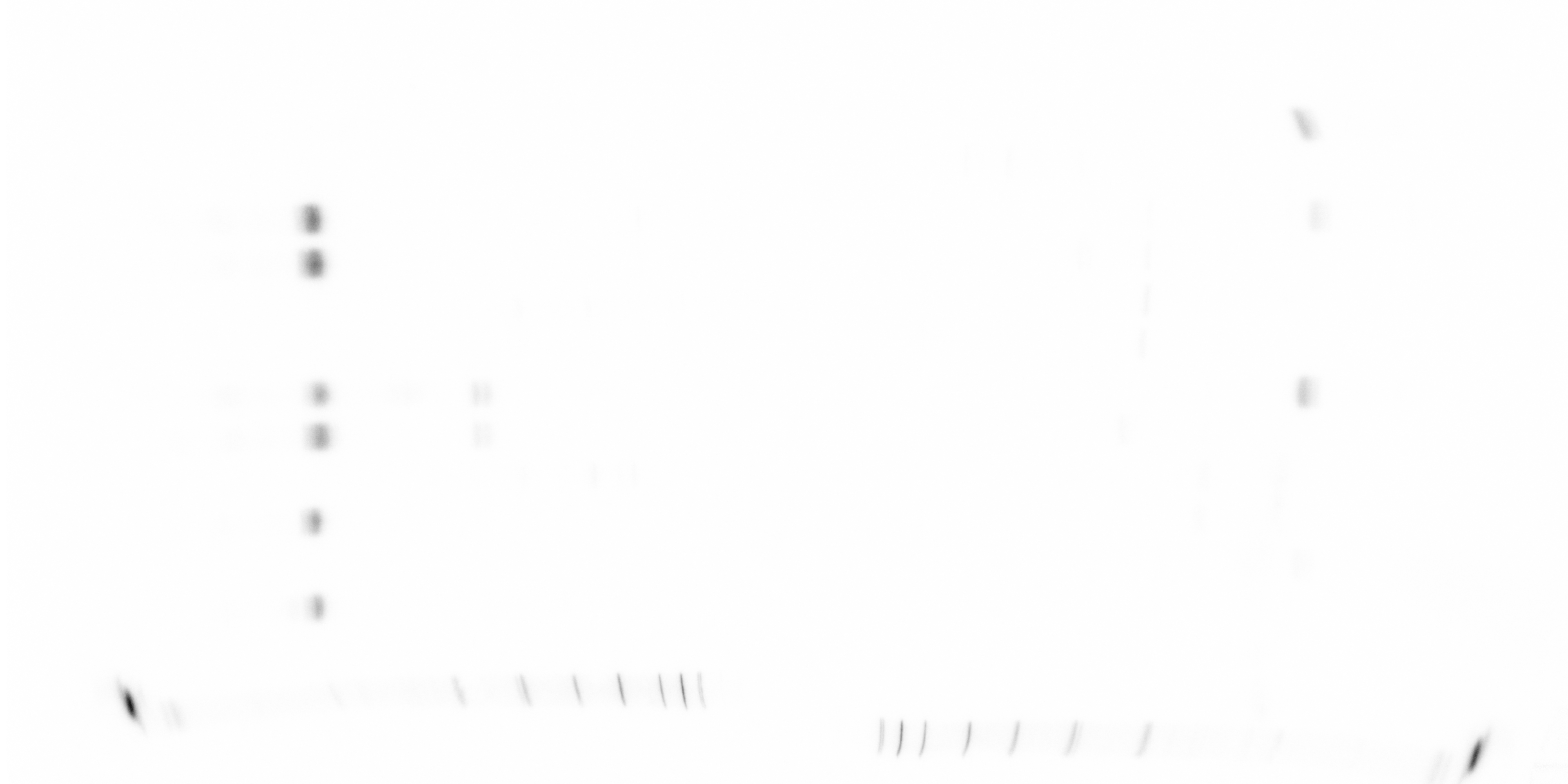

Supplement: Figure 5—source data 1. [file elife-69064-fig5-data1.zip › Source data - Figure 5/Figure 5B - 25.2.2021_NB205_206_CSO-0185_6d-[Phosphor].tif]

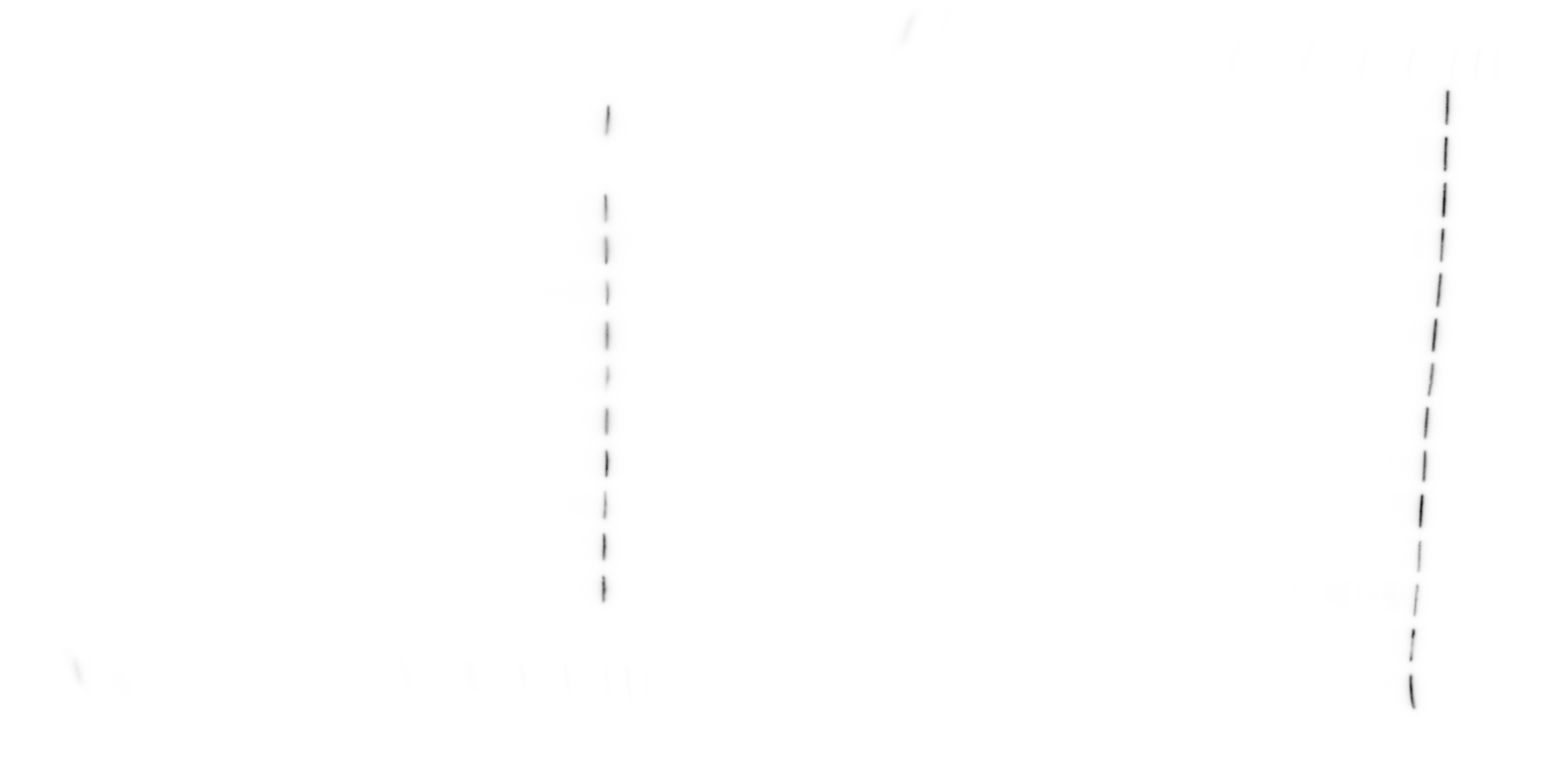

Supplement: Figure 5—source data 1. [file elife-69064-fig5-data1.zip › Source data - Figure 5/Figure 5B - 8.2.2021_NB205_206_CSO-0497_3d-[Phosphor].tif]

**Source data for Figure 5**

**Panel B**

NB205


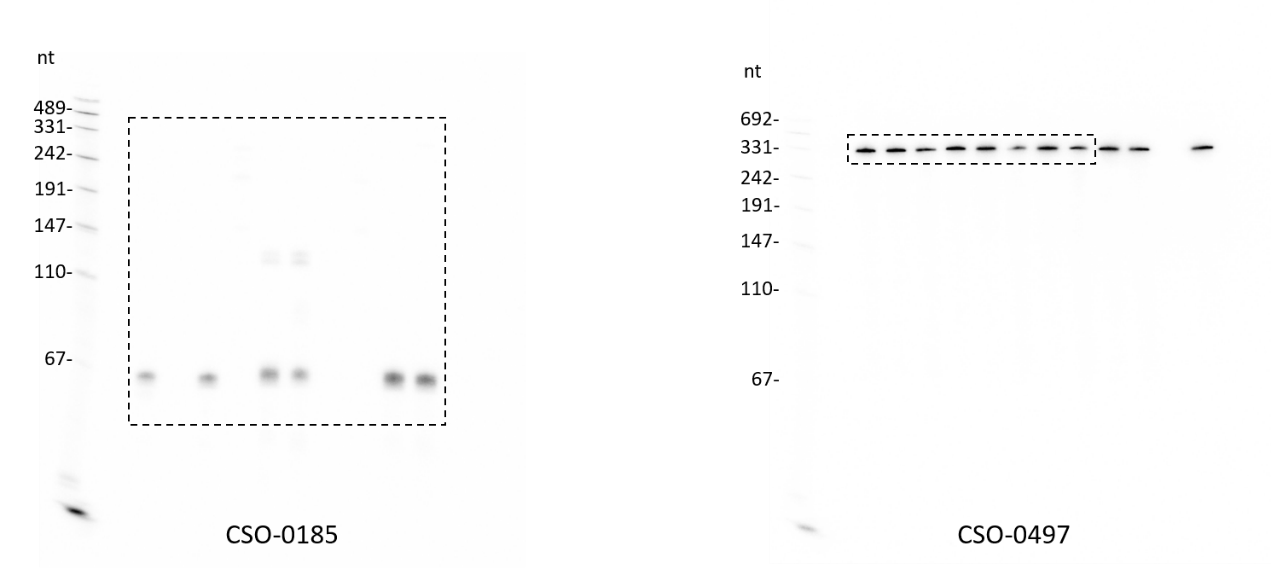

Supplement: Figure 5—source data 1. [file elife-69064-fig5-data1.zip › Source data - Figure 5/Source data - Figure 5.docx]

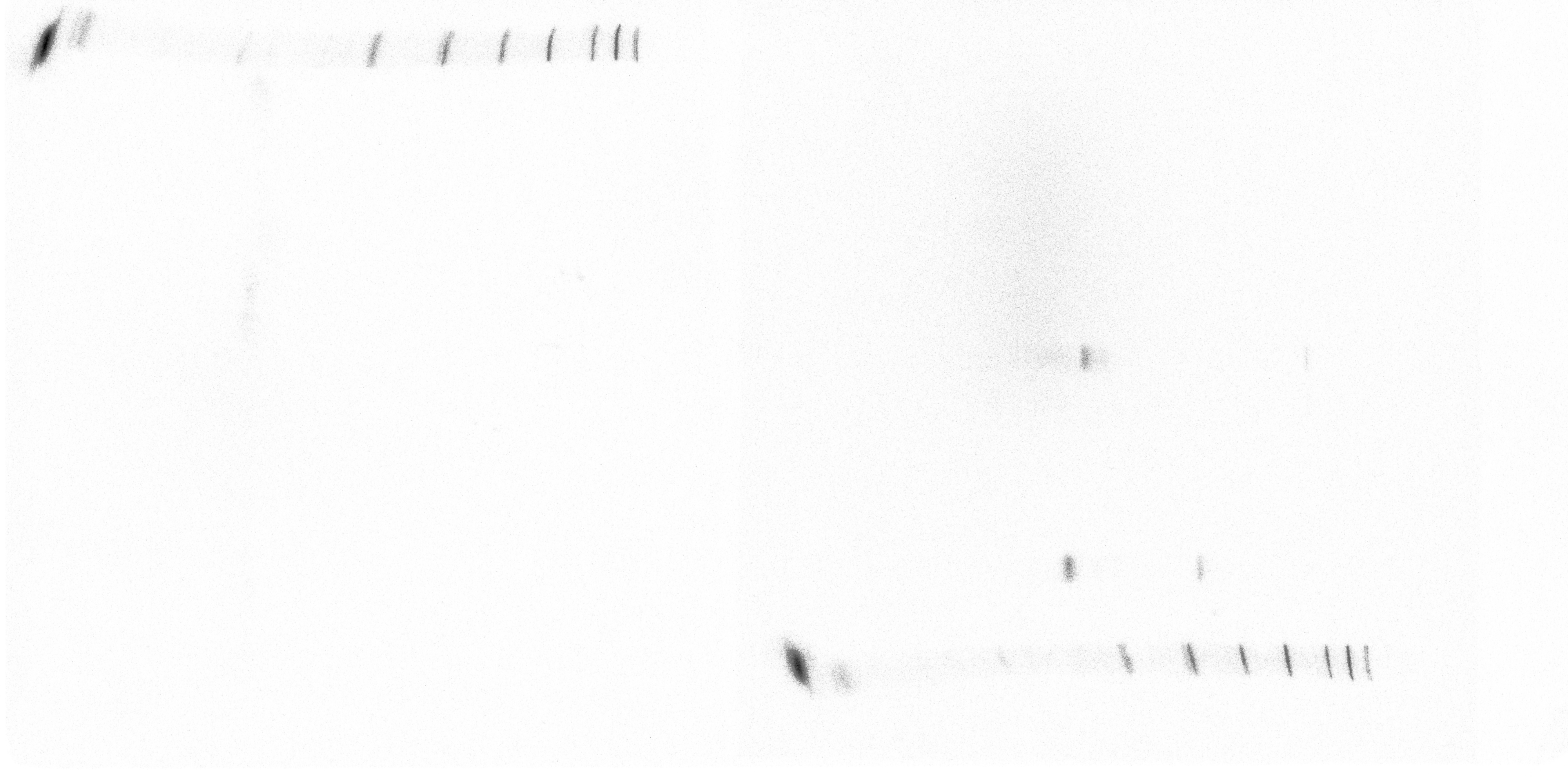

Supplement: Figure 5—figure supplement 2—source data 1. [file elife-69064-fig5-figsupp2-data1.zip › Source data - Figure 5 - figure supplement 2/Fig 5 - supp 2B - 1.3.2021_NB205_206_CSO-0189_3d-[Phosphor].tif]

**Source data for Figure 5 – figure supplement 2**

**Panel B**


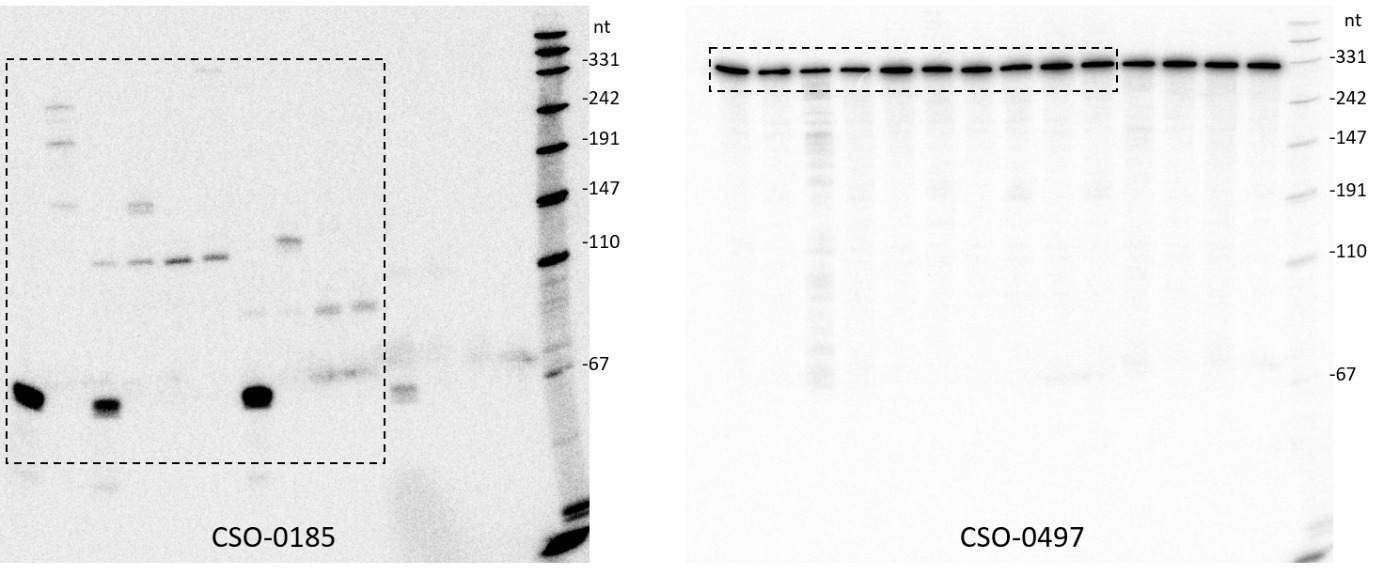

Supplement: Figure 5—figure supplement 2—source data 1. [file elife-69064-fig5-figsupp2-data1.zip › Source data - Figure 5 - figure supplement 2/Source data - Figure 5 - figure supplement 2.docx]

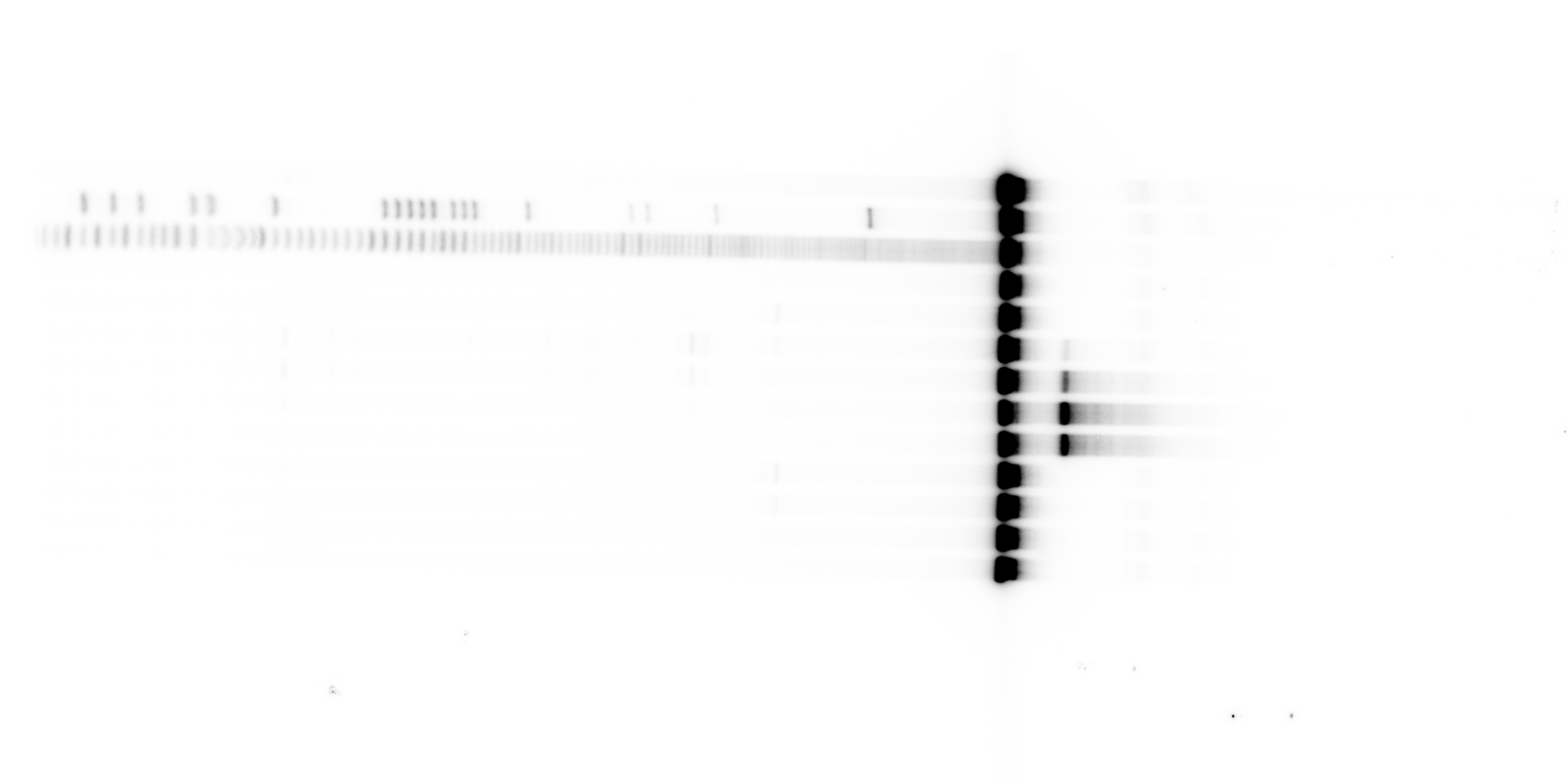

Supplement: Figure 6—source data 1. [file elife-69064-fig6-data1.zip › Source data - Figure 6/Figure 6B - 17.1.2018_cleavage1_5d-[Phosphor].tif]

**Source data for Figure 6**

**Panel B**


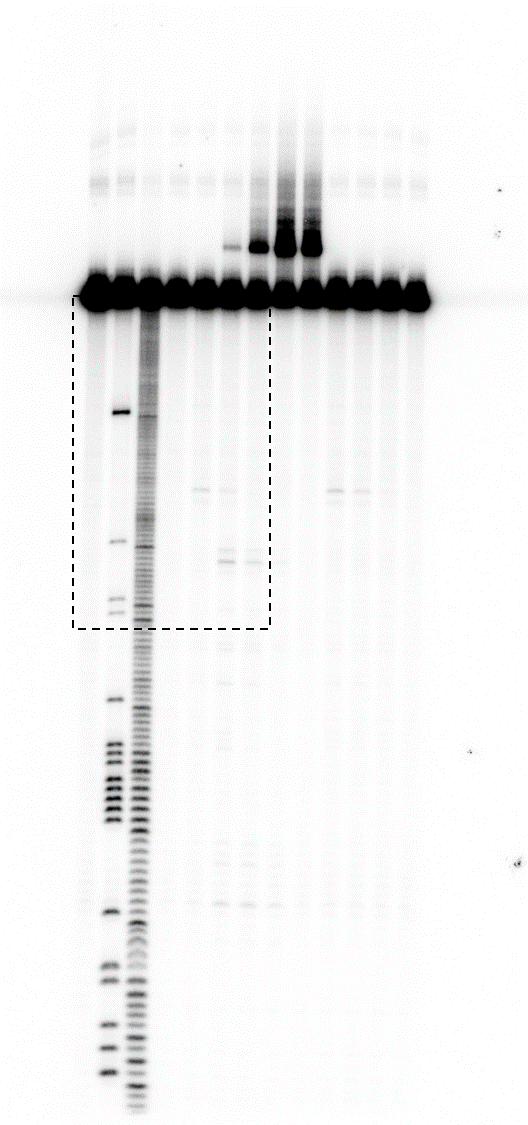

Supplement: Figure 6—source data 1. [file elife-69064-fig6-data1.zip › Source data - Figure 6/Source data - Figure 6.docx]

**Source data for Figure 6 – figure supplement 1**

**Panel B**


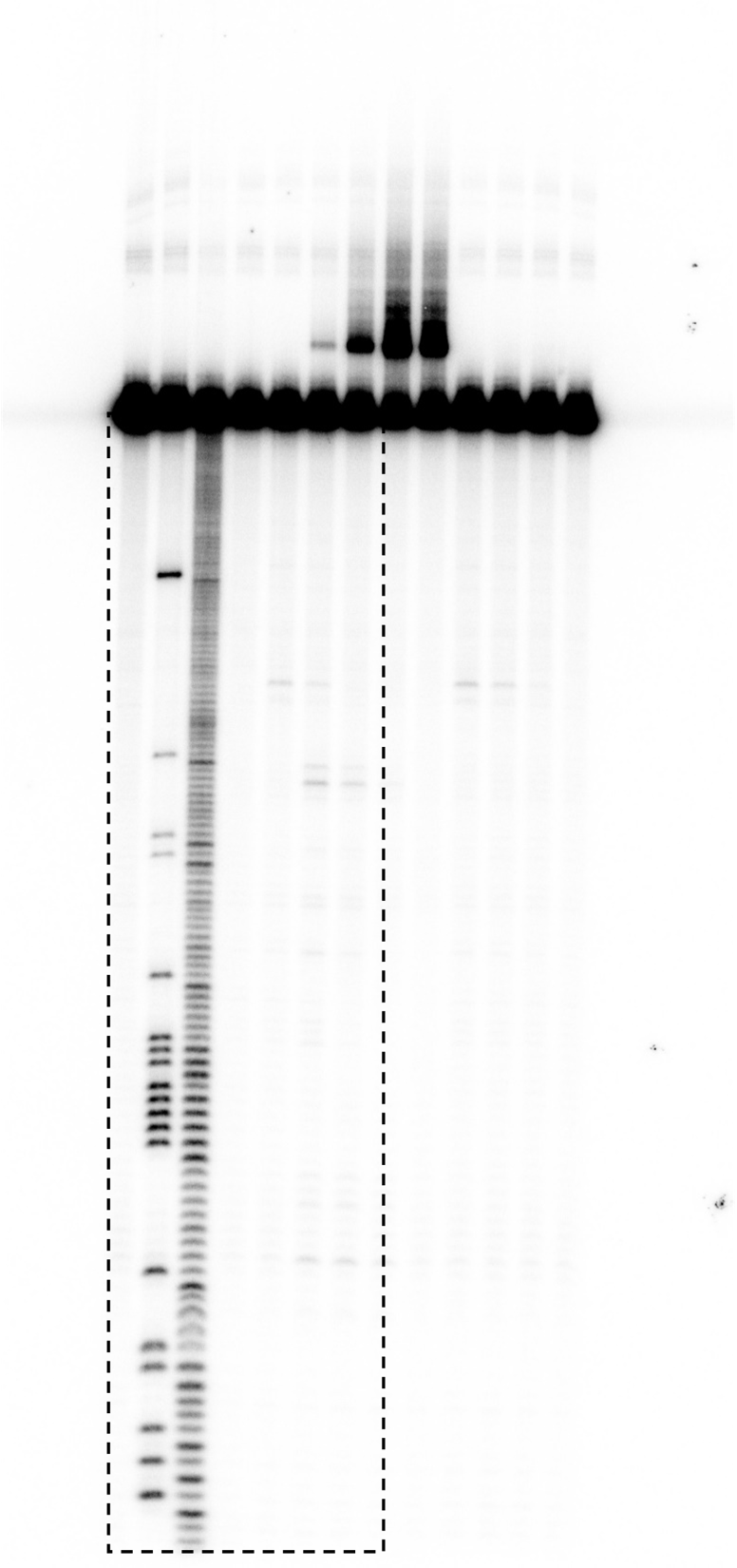

Supplement: Figure 6—figure supplement 1—source data 1. [file elife-69064-fig6-figsupp1-data1.zip › Source data - Figure 6 - figure supplement 1/Source data - Figure 6 - figure supplement 1.docx]

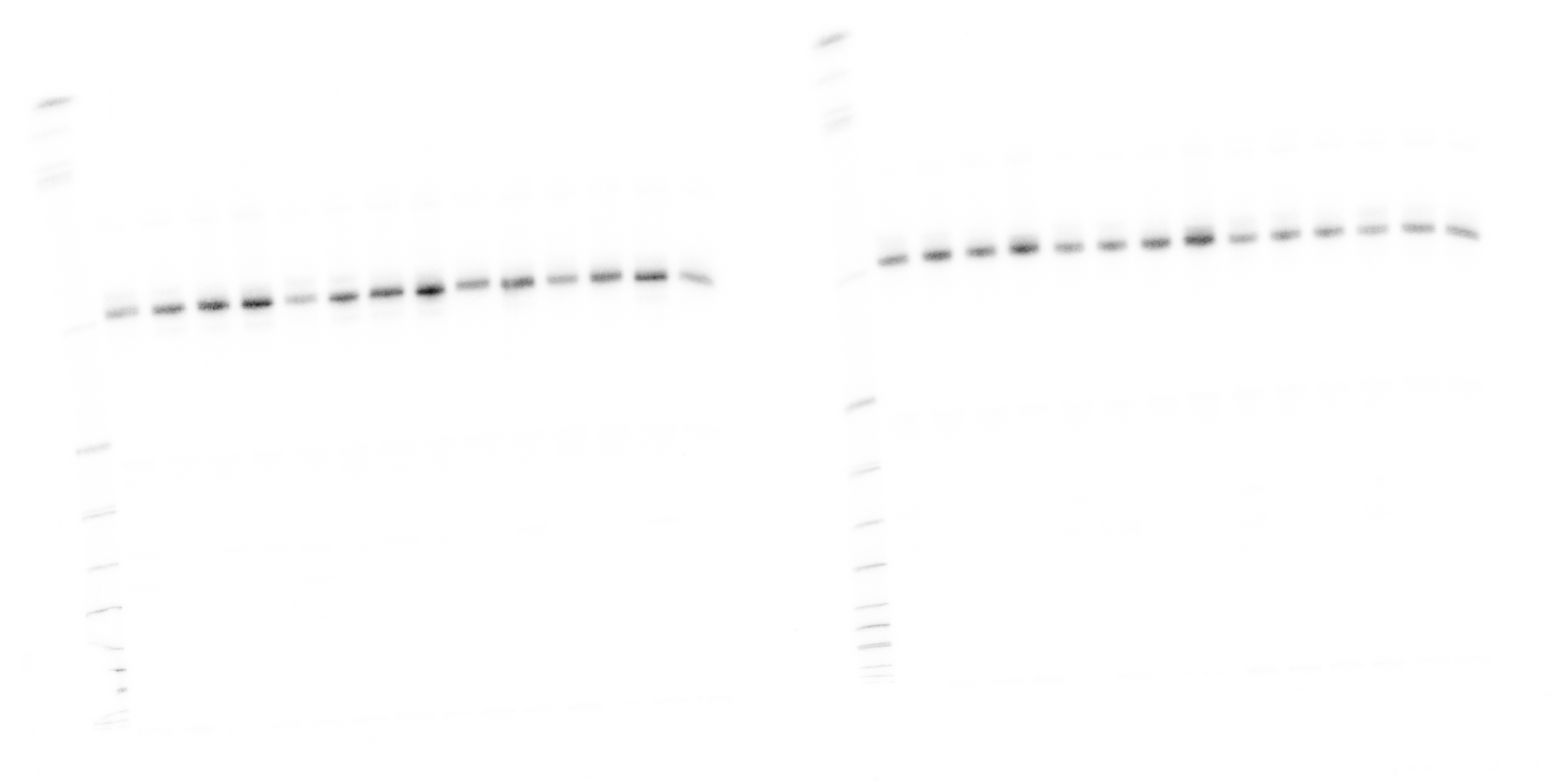

Supplement: Figure 7—source data 1. [file elife-69064-fig7-data1.zip › Source data - Figure 7/Figure 7C - 20180708_NB132_133_CSO-0185_4d-[Phosphor].tif]

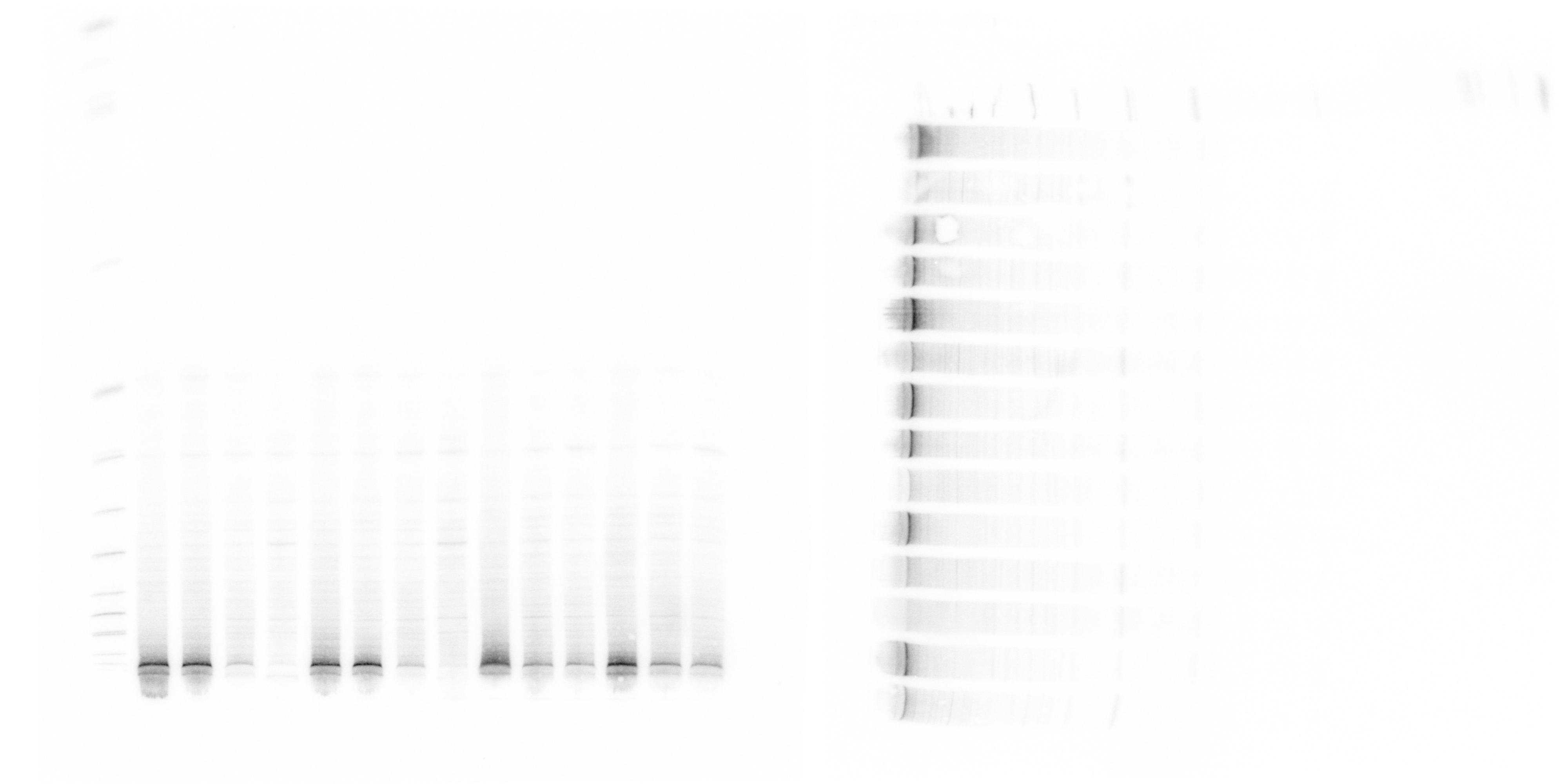

Supplement: Figure 7—source data 1. [file elife-69064-fig7-data1.zip › Source data - Figure 7/Figure 7C - 20180713_NB132_133_CSO-1666_4d-[Phosphor].tif]

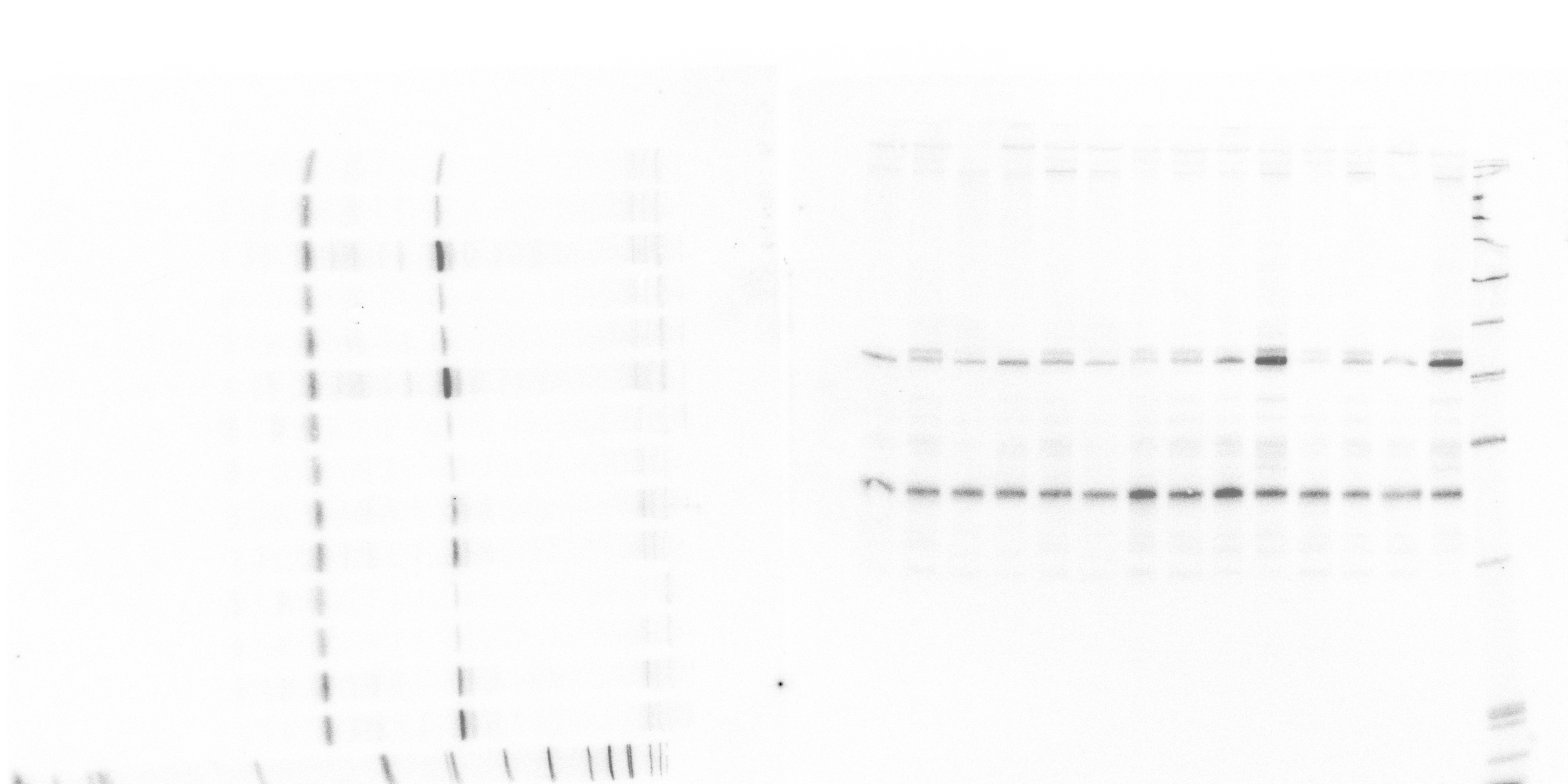

Supplement: Figure 7—source data 1. [file elife-69064-fig7-data1.zip › Source data - Figure 7/Figure 7C - 20180720_NB132_133_CSO-0189_4d-[Phosphor].tif]

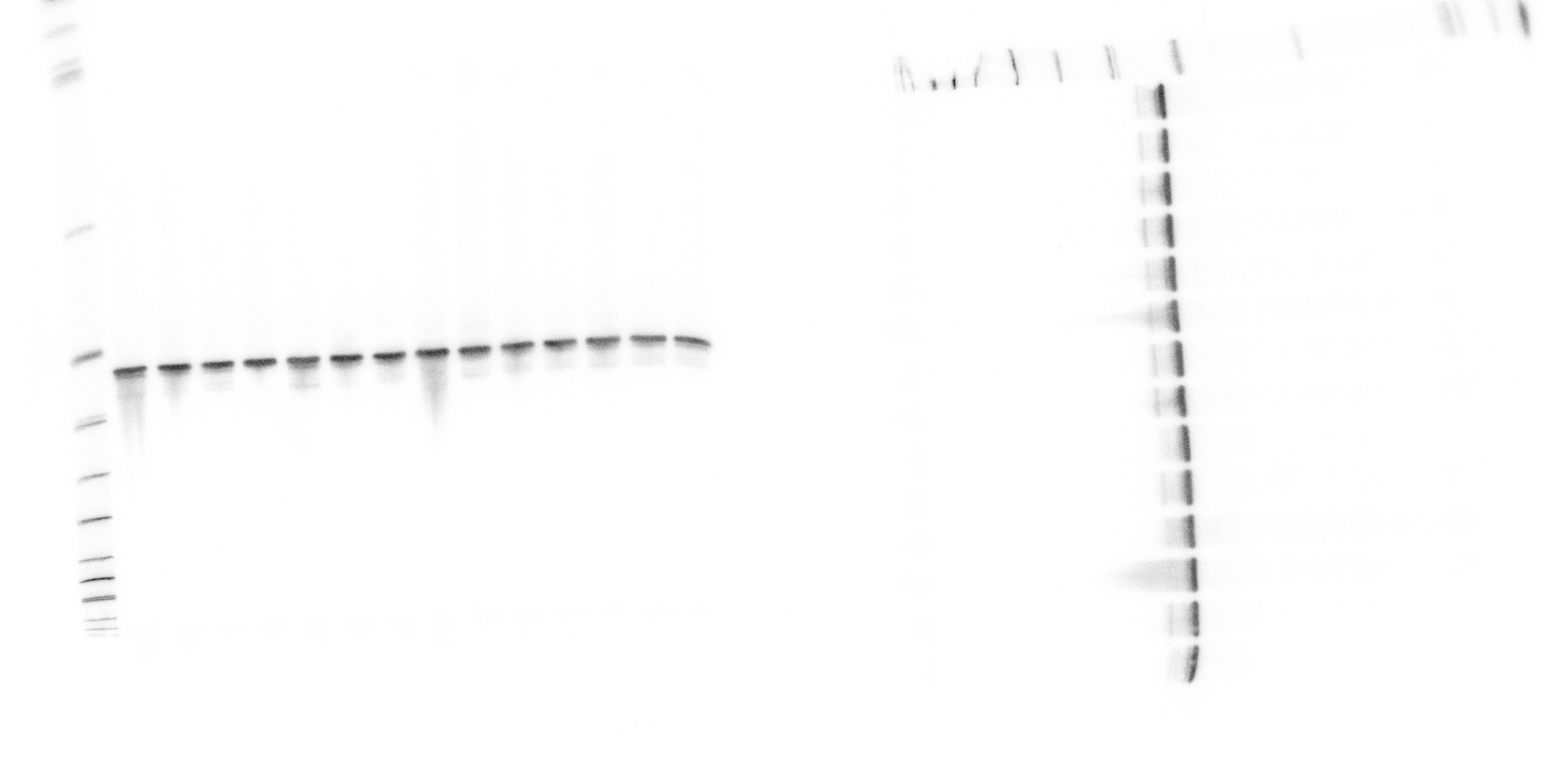

Supplement: Figure 7—source data 1. [file elife-69064-fig7-data1.zip › Source data - Figure 7/Figure 7C - 30.6.2018_NB132_133_CSO-0192_1d-[Phosphor].tif]

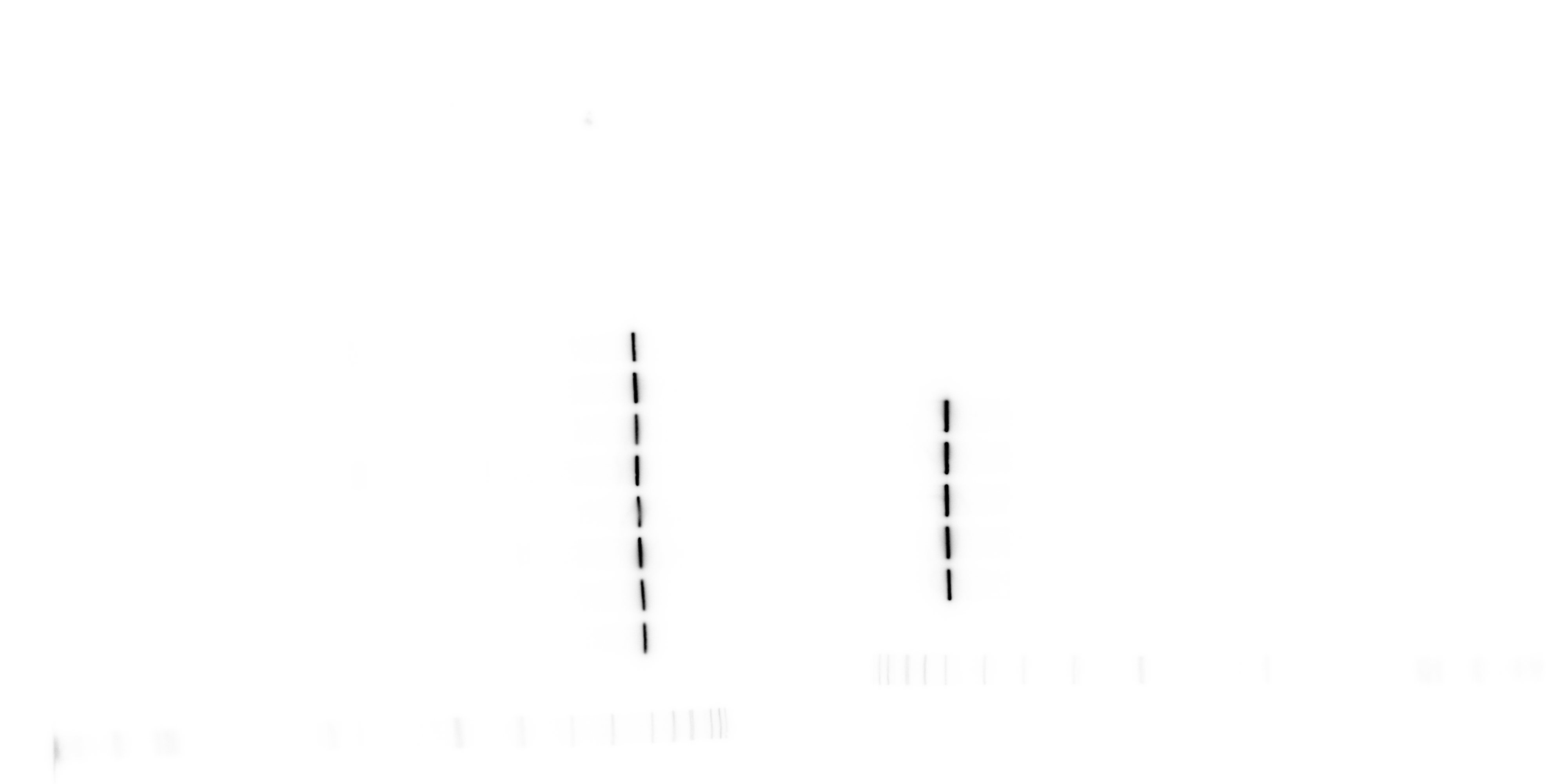

Supplement: Figure 7—figure supplement 1—source data 1. [file elife-69064-fig7-figsupp1-data1.zip › Source data - Figure 7 - figure supplement 1/Fig 7 - supp 1A - 11112017_NB120_122_CSO-0497_1d-[Phosphor].tif]

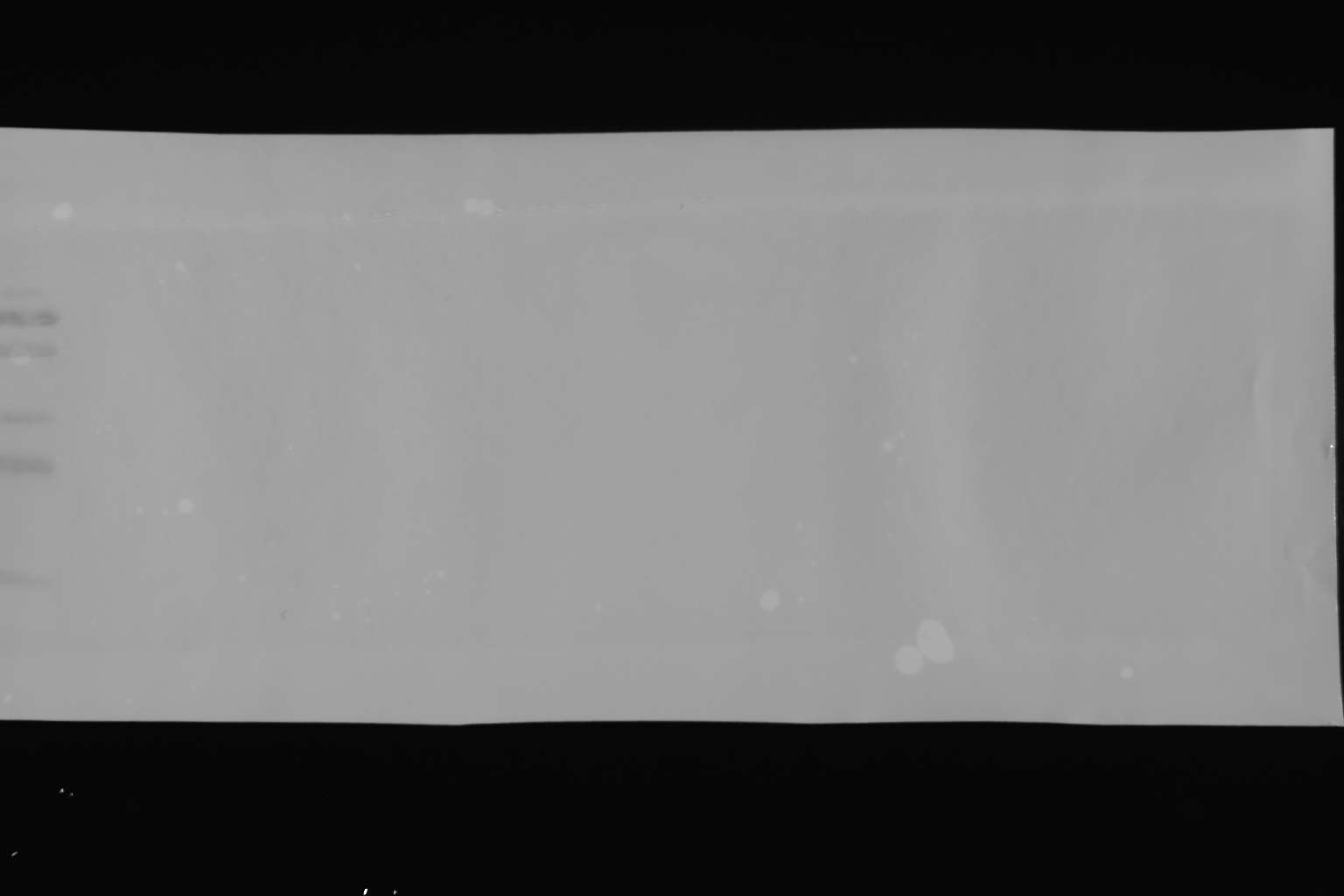

Supplement: Figure 7—figure supplement 1—source data 1. [file elife-69064-fig7-figsupp1-data1.zip › Source data - Figure 7 - figure supplement 1/Fig 7 - supp 1A - 20171022_1653_FLAG_ladder.gel]

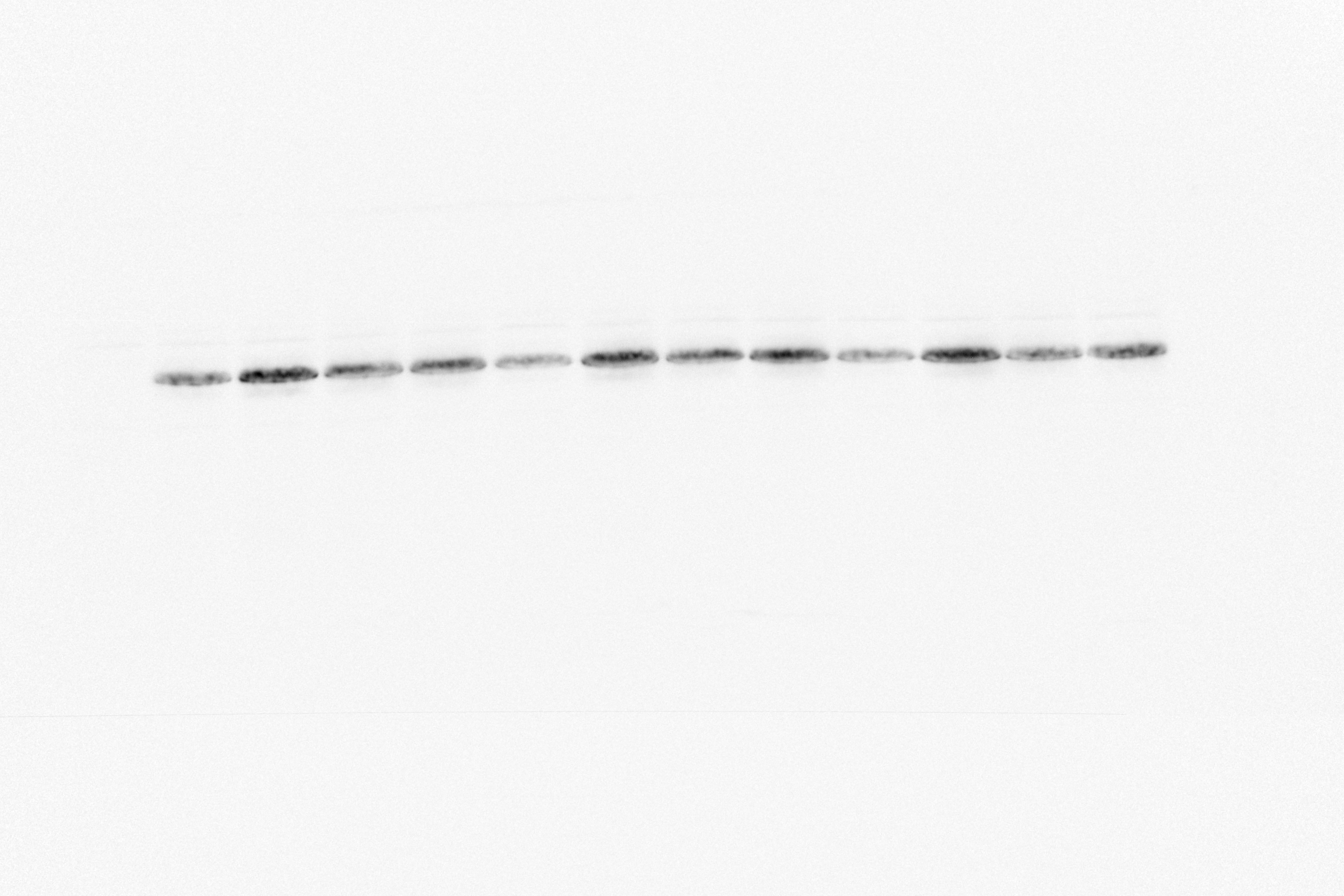

Supplement: Figure 7—figure supplement 1—source data 1. [file elife-69064-fig7-figsupp1-data1.zip › Source data - Figure 7 - figure supplement 1/Fig 7 - supp 1A - 20171022_1659_blot2_FLAG_17.gel]

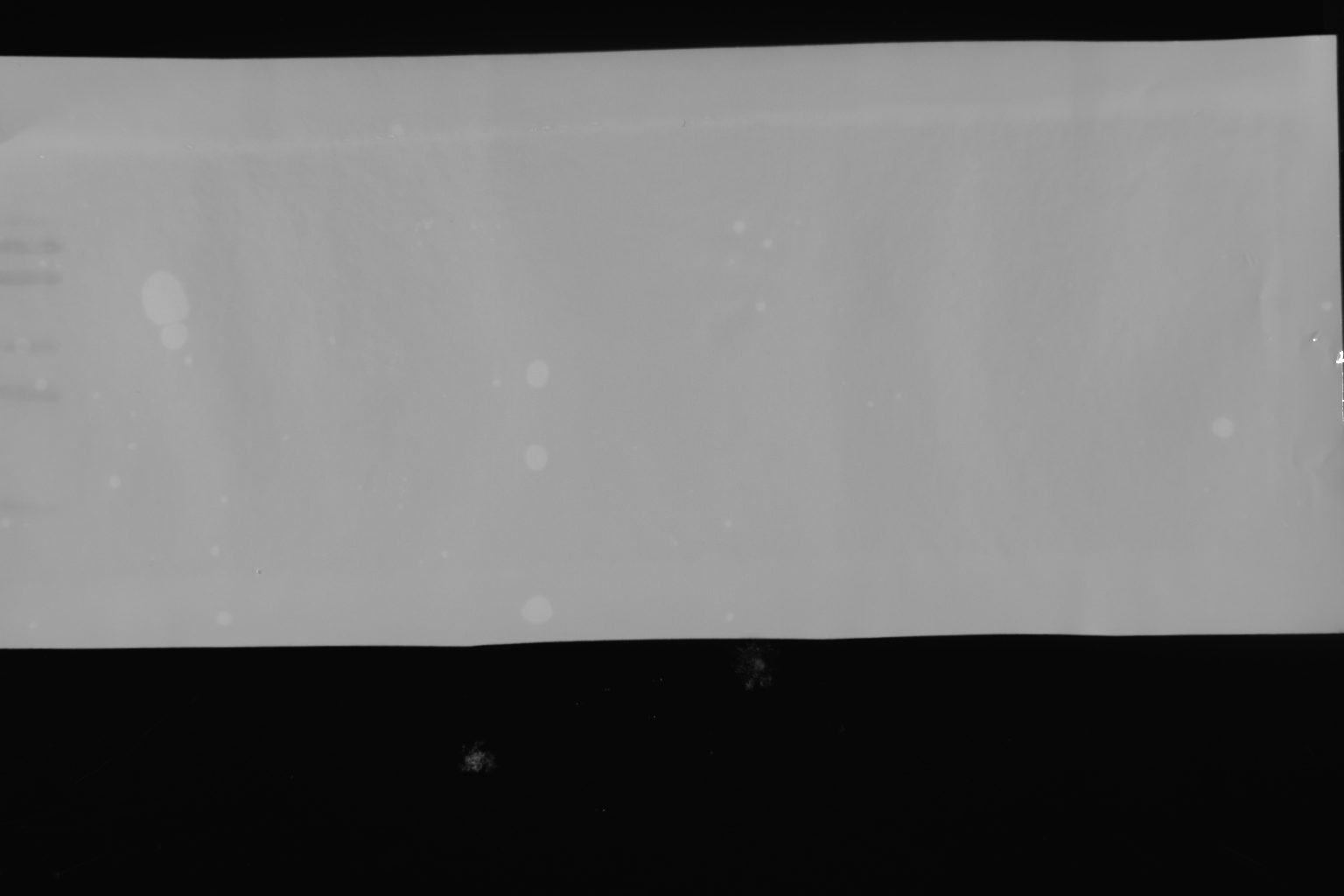

Supplement: Figure 7—figure supplement 1—source data 1. [file elife-69064-fig7-figsupp1-data1.zip › Source data - Figure 7 - figure supplement 1/Fig 7 - supp 1A - 20171023_1542_blot2_GroEL_ladder.gel]

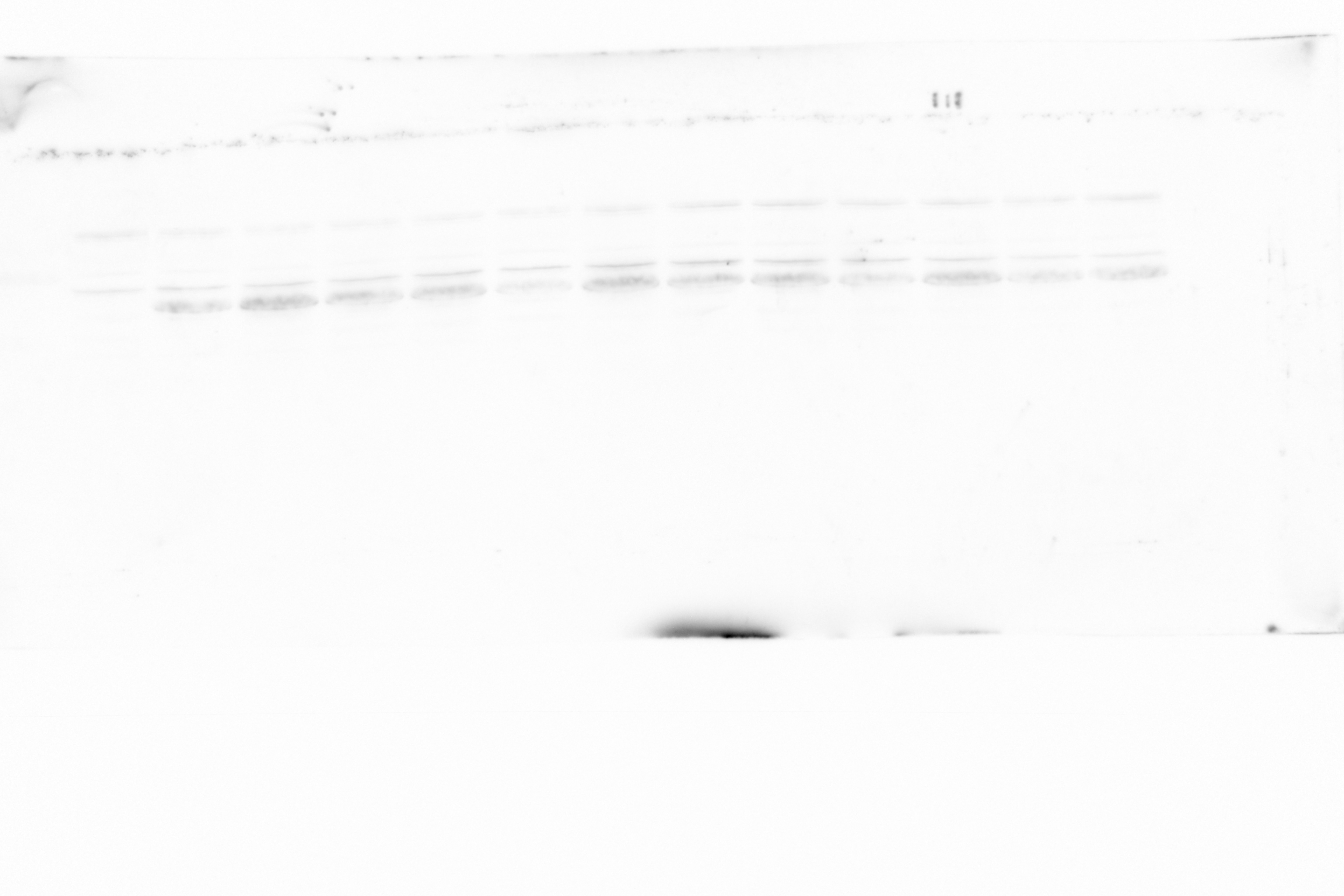

Supplement: Figure 7—figure supplement 1—source data 1. [file elife-69064-fig7-figsupp1-data1.zip › Source data - Figure 7 - figure supplement 1/Fig 7 - supp 1A - 20171023_1548_blot2_GroEL_7.gel]

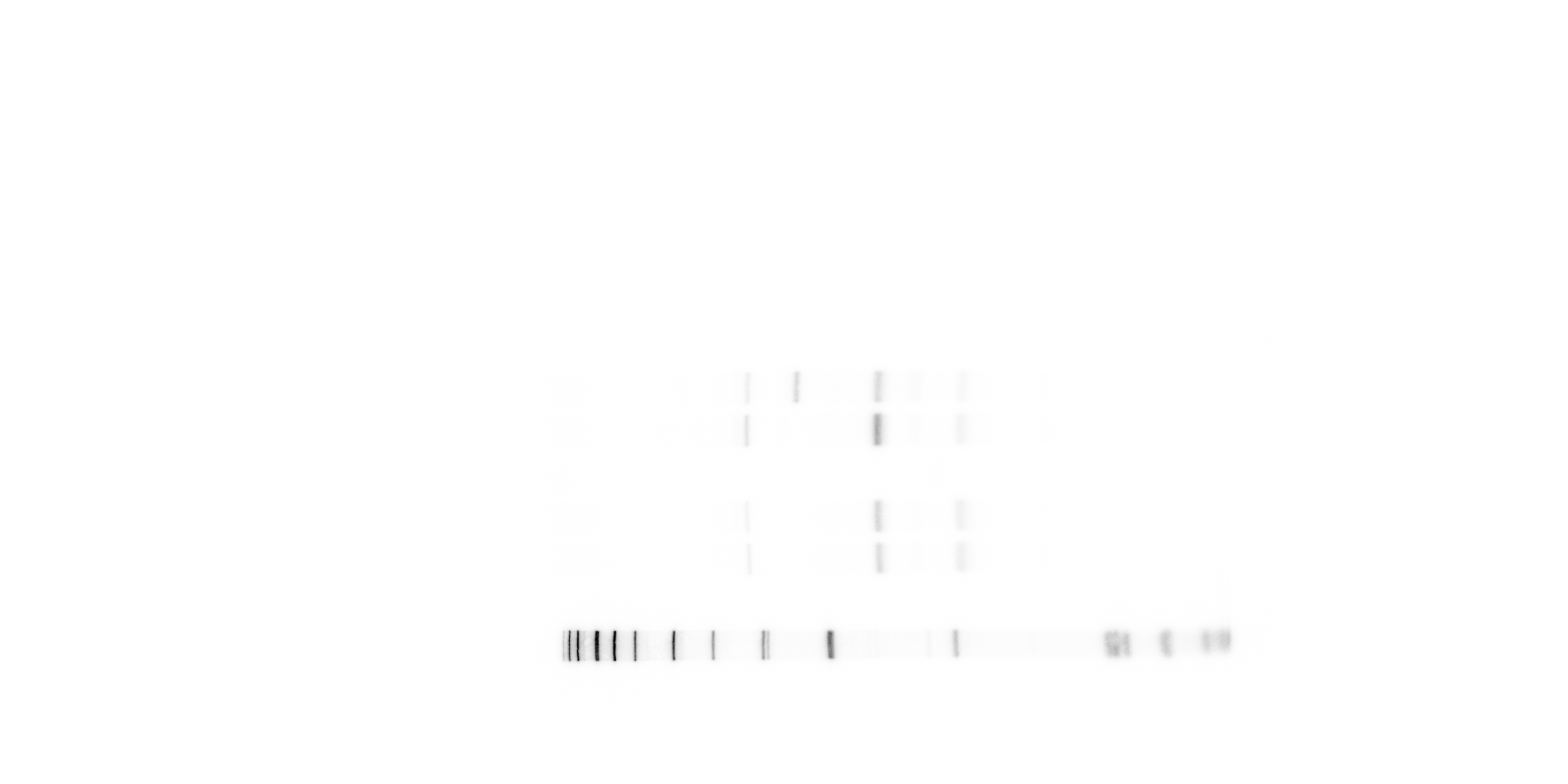

Supplement: Figure 7—figure supplement 1—source data 1. [file elife-69064-fig7-figsupp1-data1.zip › Source data - Figure 7 - figure supplement 1/Fig 7 - supp 1A - 20171103_NB122_CSO-0189_5d-[Phosphor].tif]

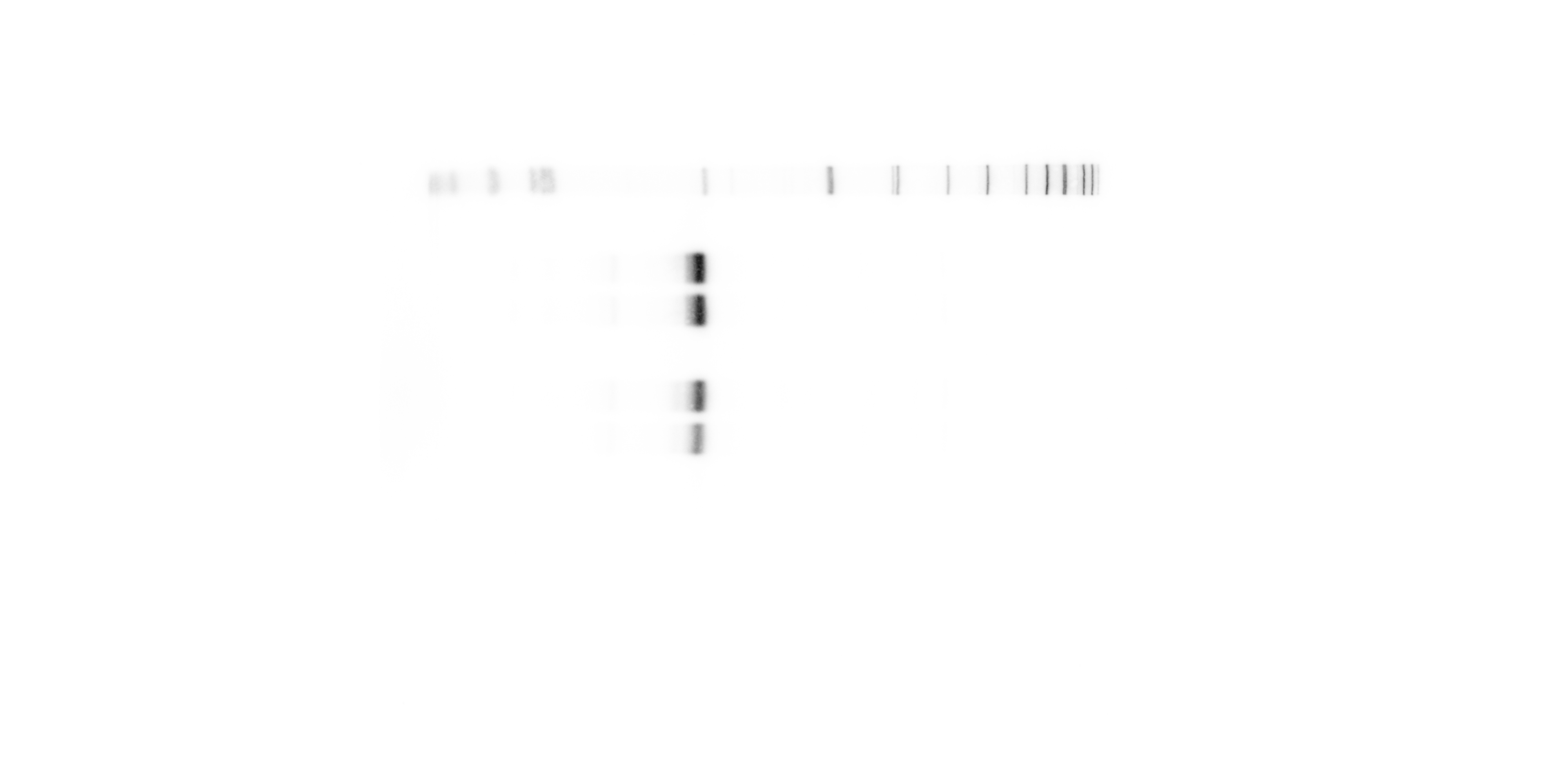

Supplement: Figure 7—figure supplement 1—source data 1. [file elife-69064-fig7-figsupp1-data1.zip › Source data - Figure 7 - figure supplement 1/Fig 7 - supp 1A - 20171110_NB122_CSO-0185_6d-[Phosphor].tif]

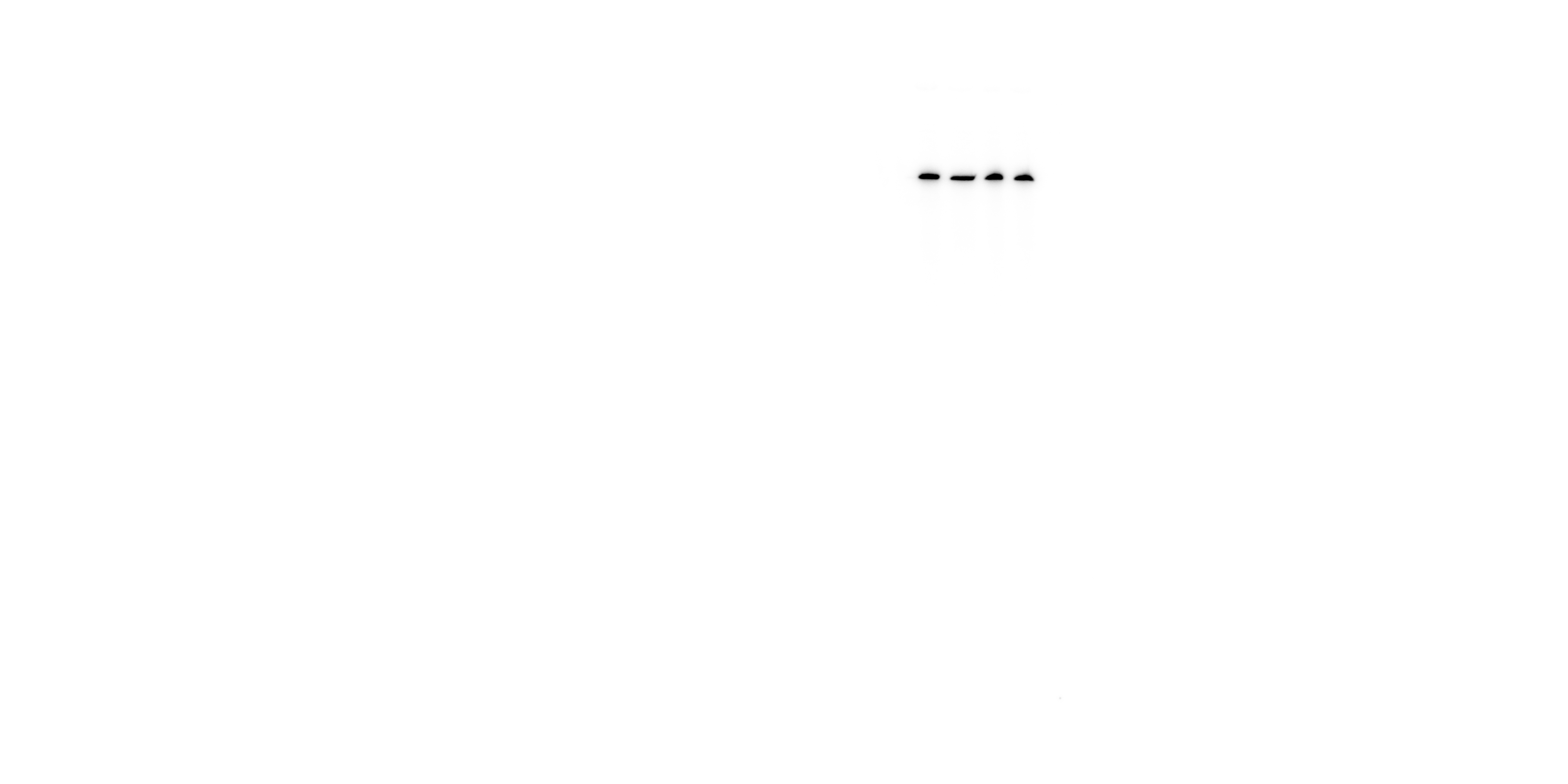

Supplement: Figure 7—figure supplement 1—source data 1. [file elife-69064-fig7-figsupp1-data1.zip › Source data - Figure 7 - figure supplement 1/Fig 7 - supp 1B - 24.1.2018_NB127_CSO-0497_1d-[Phosphor].tif]

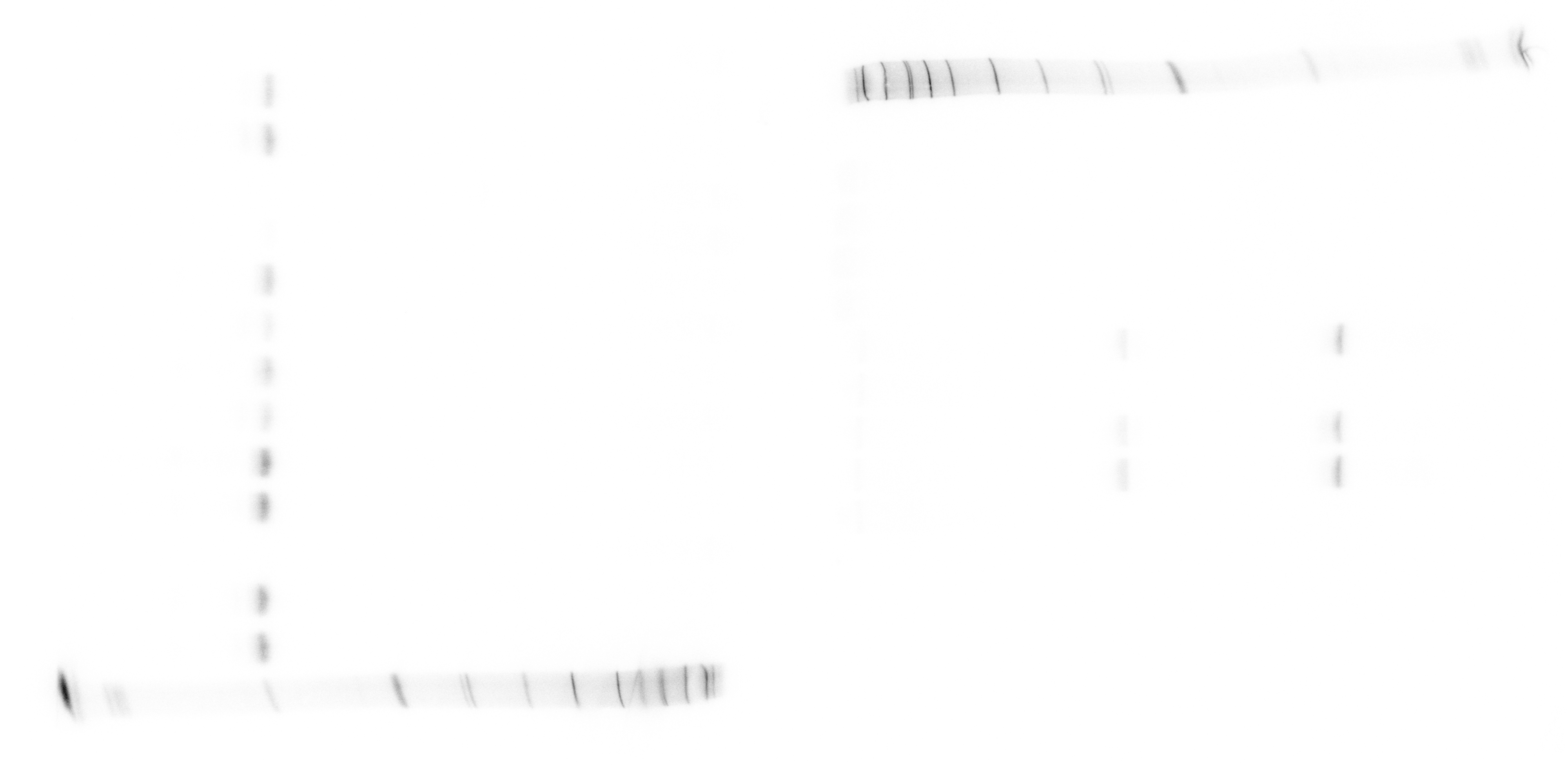

Supplement: Figure 7—figure supplement 1—source data 1. [file elife-69064-fig7-figsupp1-data1.zip › Source data - Figure 7 - figure supplement 1/Fig 7 - supp 1D - 19.7.2021_NB213_214_CSO-0185_214_3855_3d-[Phosphor].tif]

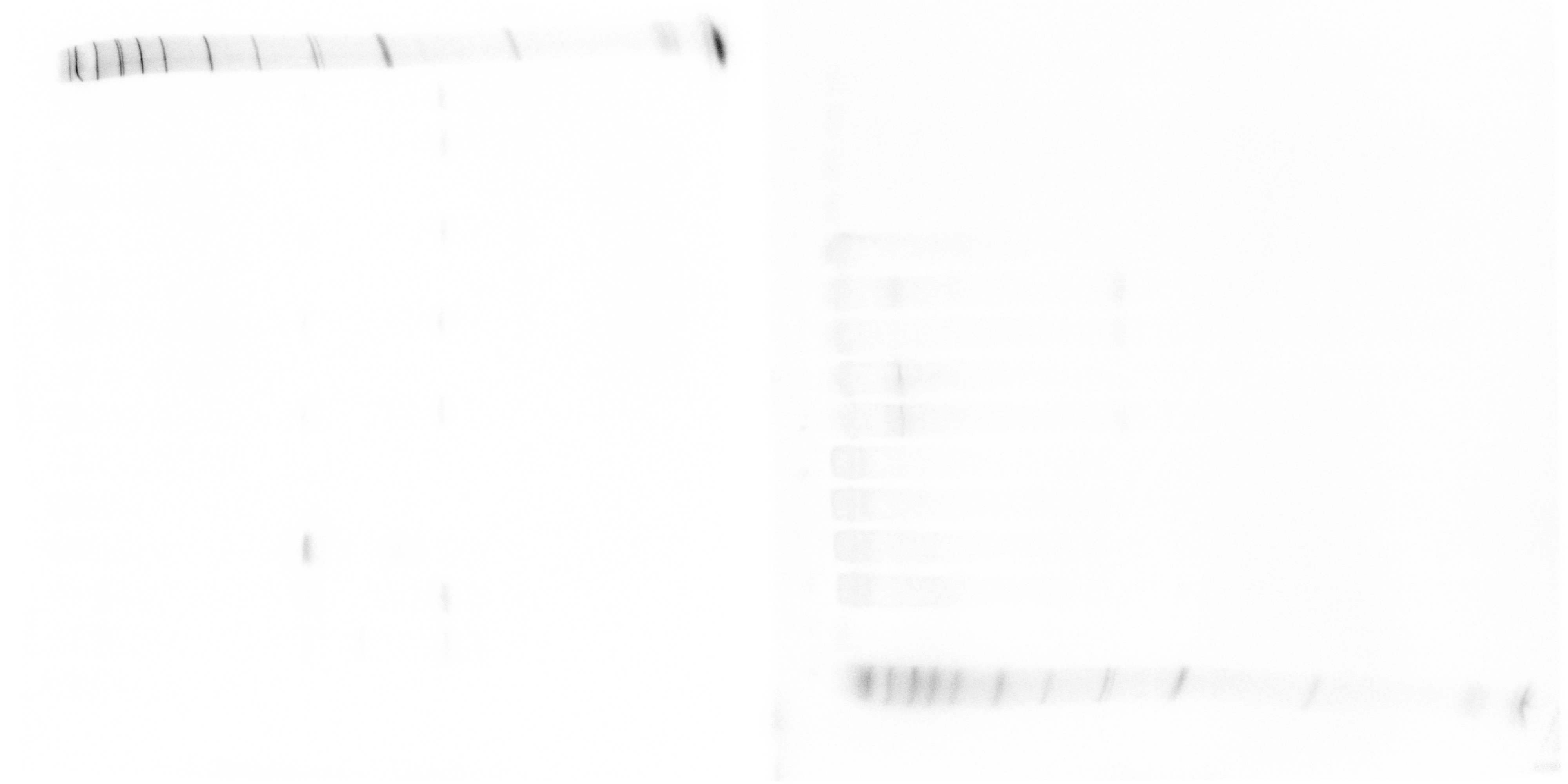

Supplement: Figure 7—figure supplement 1—source data 1. [file elife-69064-fig7-figsupp1-data1.zip › Source data - Figure 7 - figure supplement 1/Fig 7 - supp 1D - 24.7.2021_NB213_214_5635_0189_3d-[Phosphor].tif]

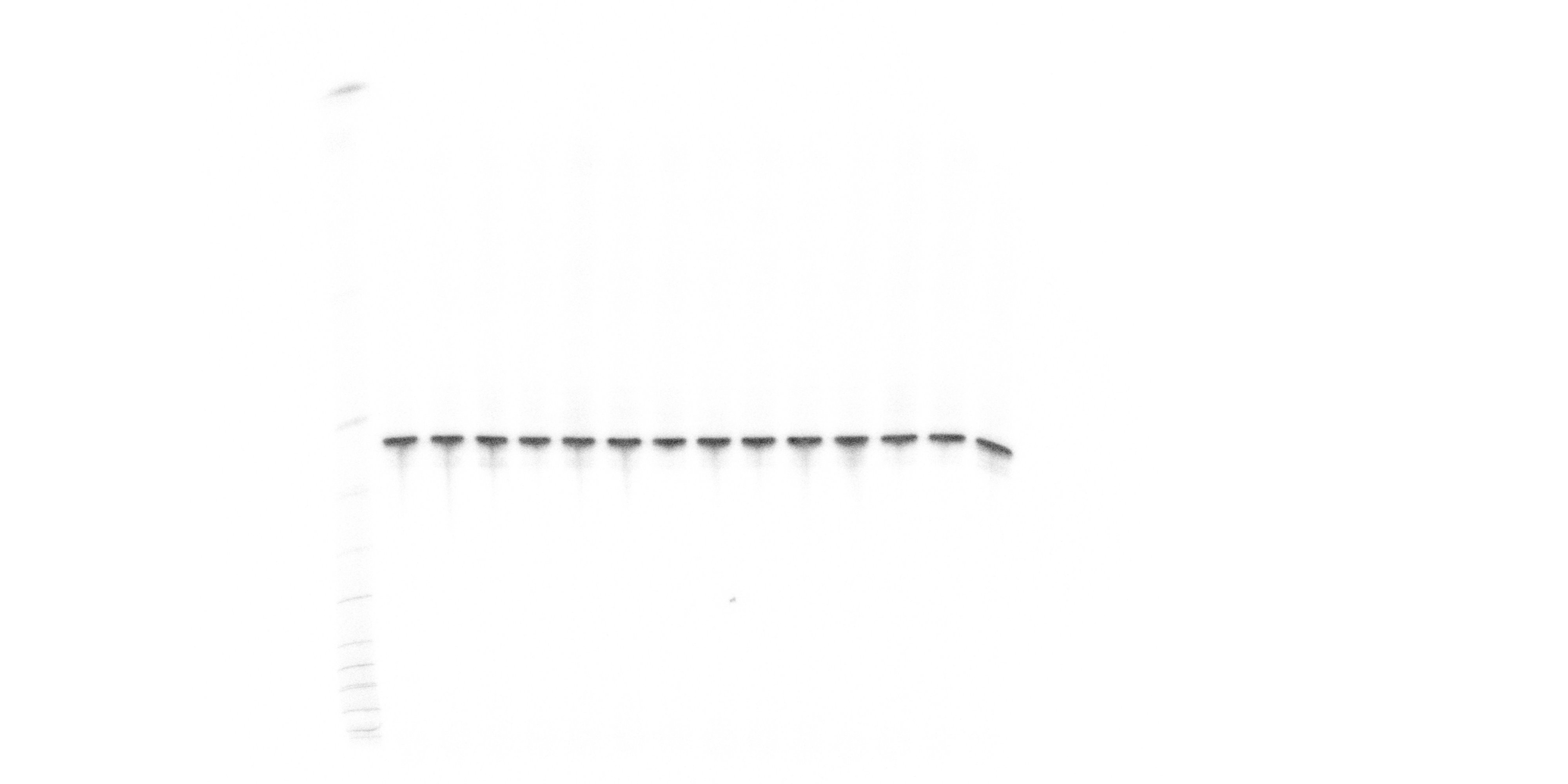

Supplement: Figure 7—figure supplement 1—source data 1. [file elife-69064-fig7-figsupp1-data1.zip › Source data - Figure 7 - figure supplement 1/Fig 7 - supp 1D - 25.7.2021_NB213_CSO-0192_3h-[Phosphor].tif]

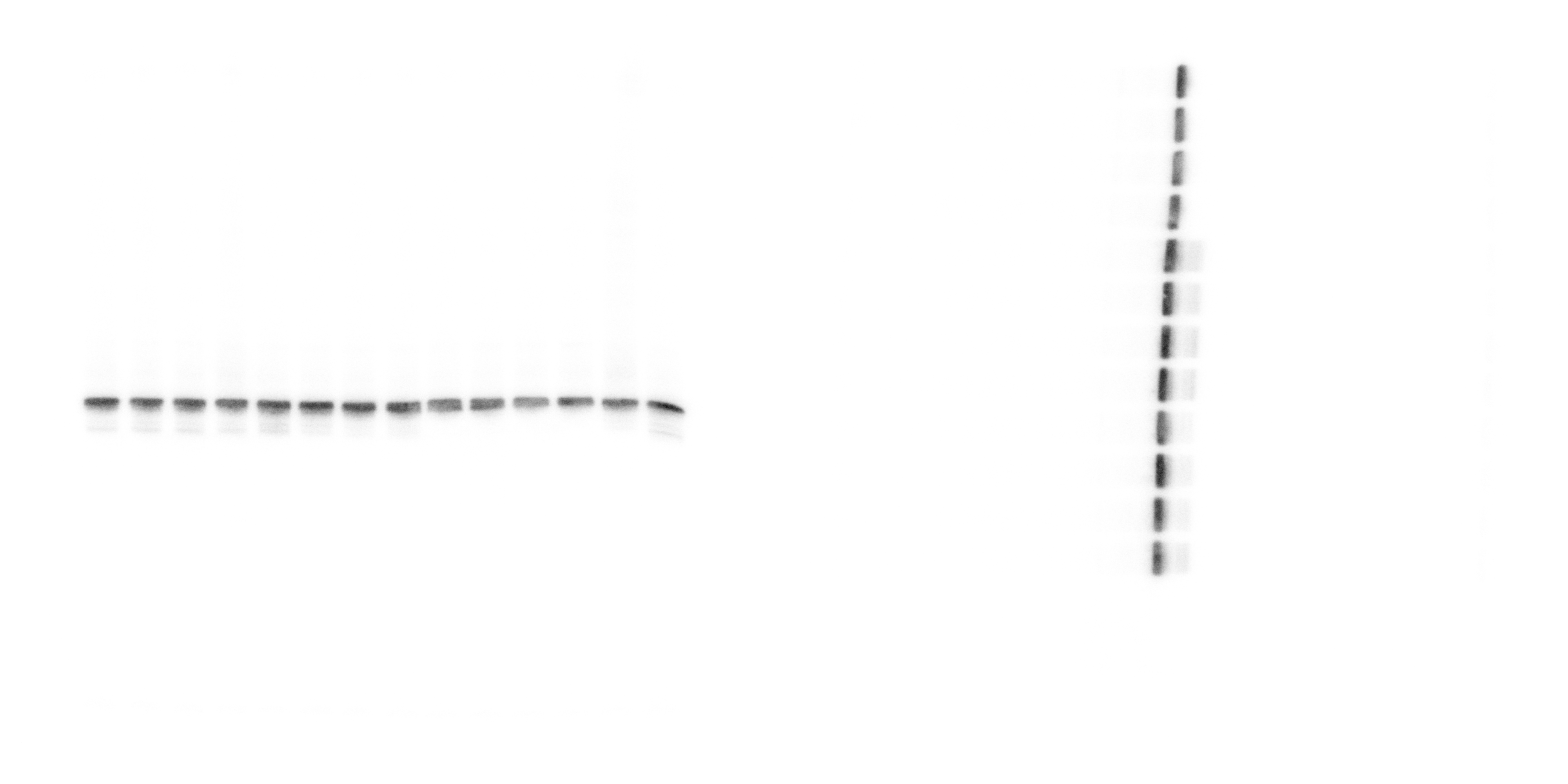

Supplement: Figure 7—figure supplement 2—source data 1. [file elife-69064-fig7-figsupp2-data1.zip › Source data - Figure 7 - figure supplement 2/Fig 7 - supp 2 - 08.11.18_NB145_146_CSO-0192_1h-[Phosphor].tif]

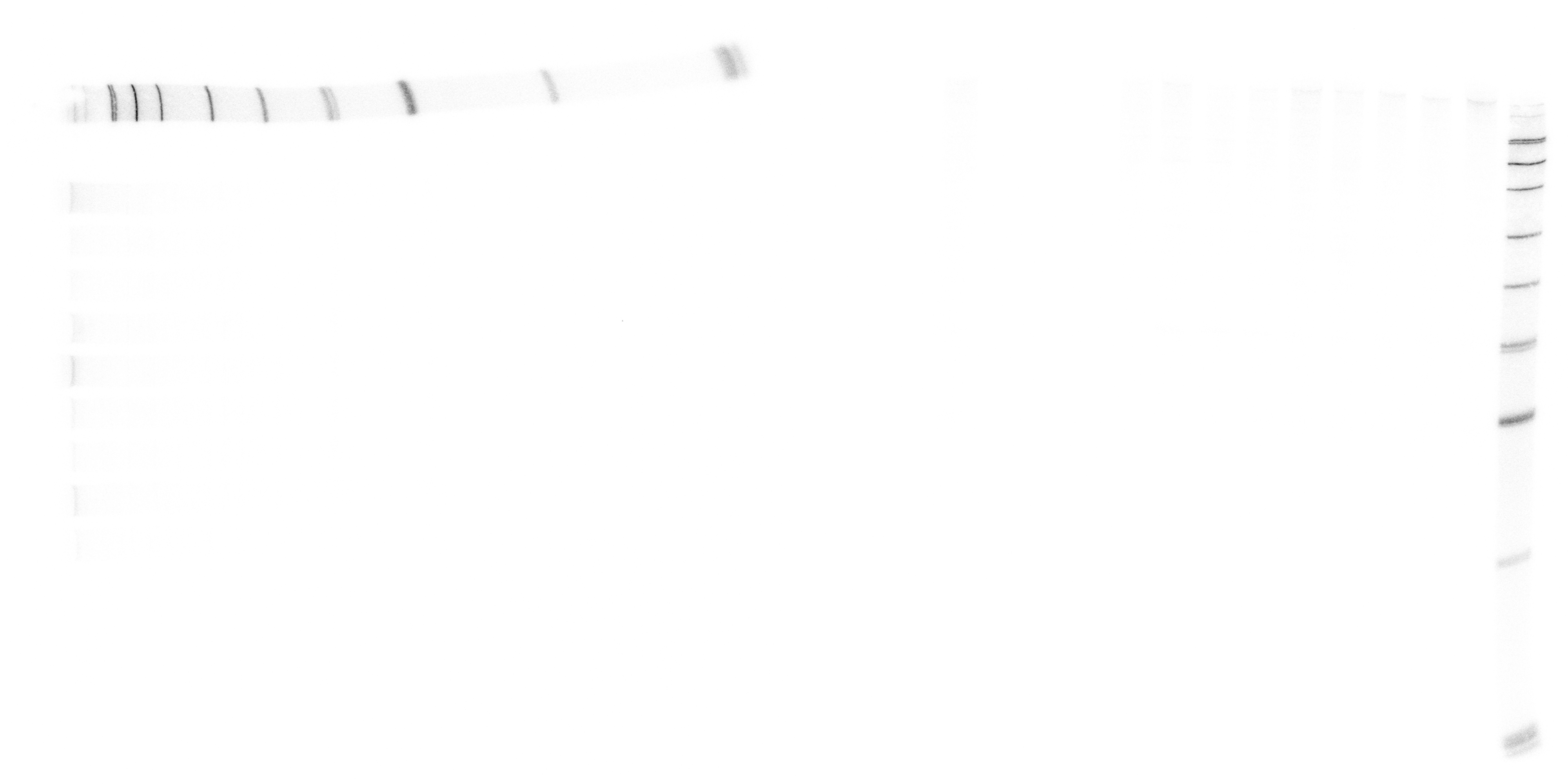

Supplement: Figure 7—figure supplement 2—source data 1. [file elife-69064-fig7-figsupp2-data1.zip › Source data - Figure 7 - figure supplement 2/Fig 7 - supp 2 - 20181012_SSv_NB145_146_CSO-1666_3d-[Phosphor].tif]

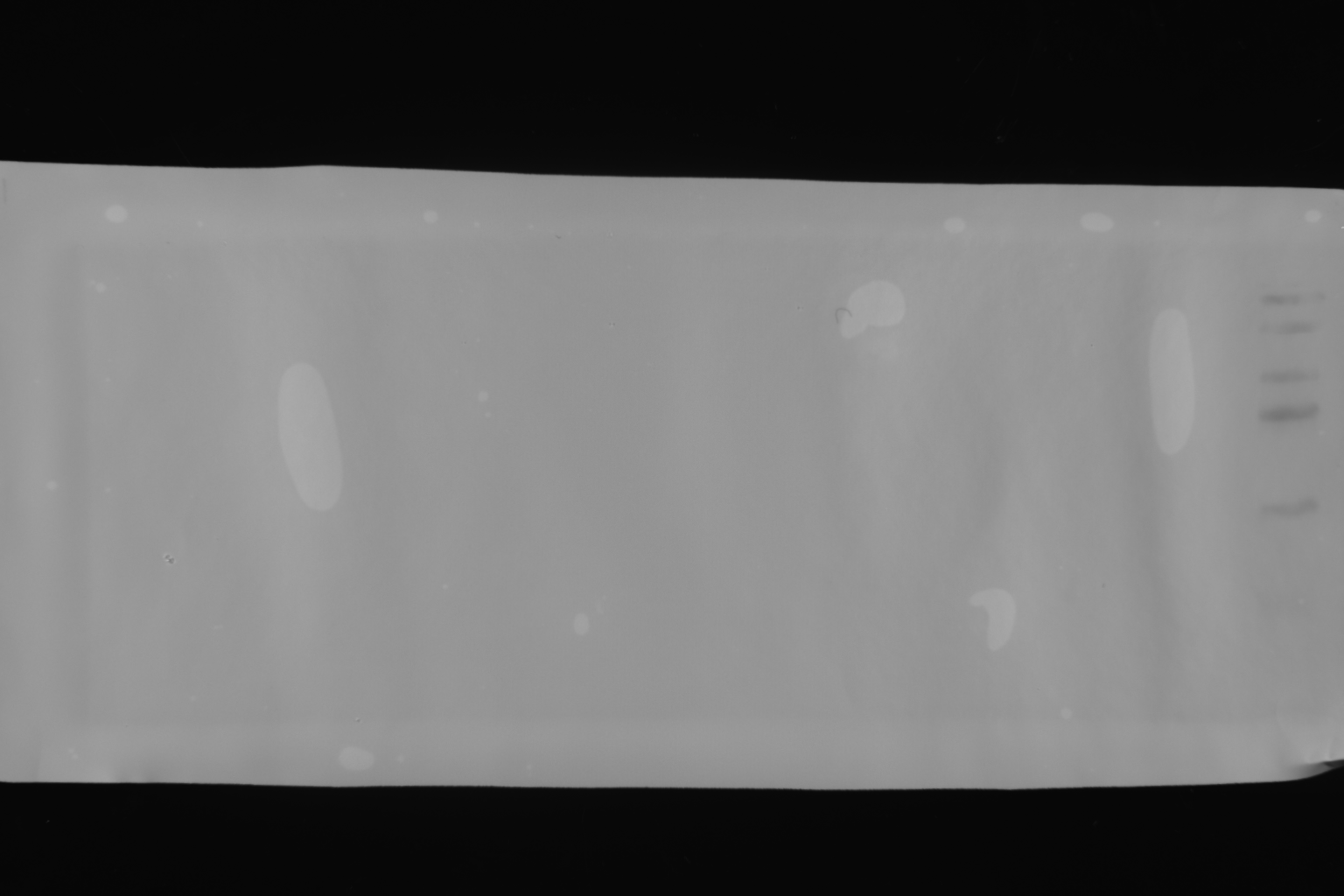

Supplement: Figure 7—figure supplement 2—source data 1. [file elife-69064-fig7-figsupp2-data1.zip › Source data - Figure 7 - figure supplement 2/Fig 7 - supp 2 - 20181014_1655_R1_FLAG_ladder.tif]

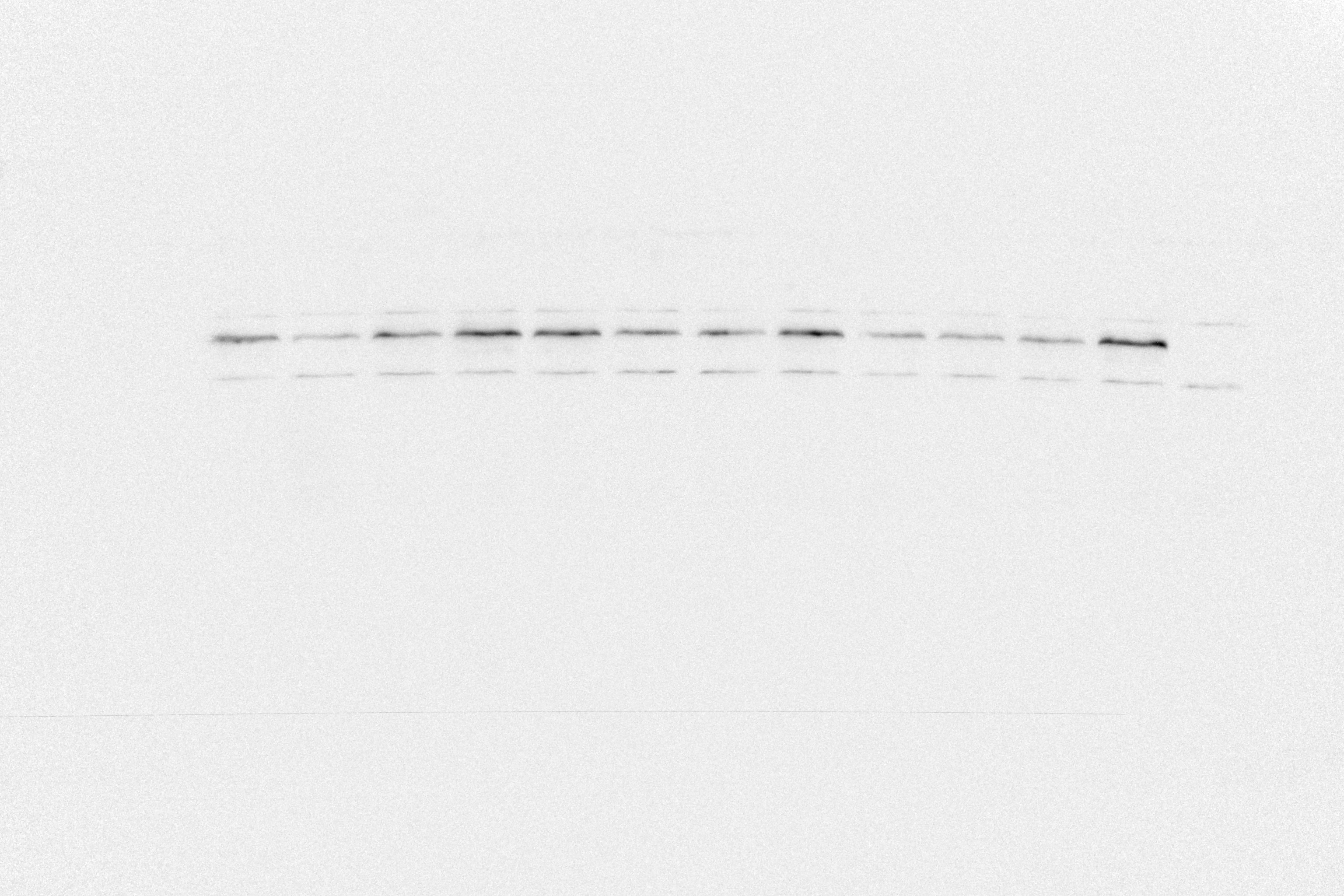

Supplement: Figure 7—figure supplement 2—source data 1. [file elife-69064-fig7-figsupp2-data1.zip › Source data - Figure 7 - figure supplement 2/Fig 7 - supp 2 - 20181014_1702_R1_FLAG_19.tif]
