# Supplementary material for: RNase III-mediated processing of a trans-acting bacterial sRNA and its cis-encoded antagonist
Source: eLife. 2021 Nov 29;10:e69064. doi: 10.7554/eLife.69064 (PMC8687705; doi:10.7554/eLife.69064)

**Source data for Figure 1 - figure supplement 4**

**Panel A**


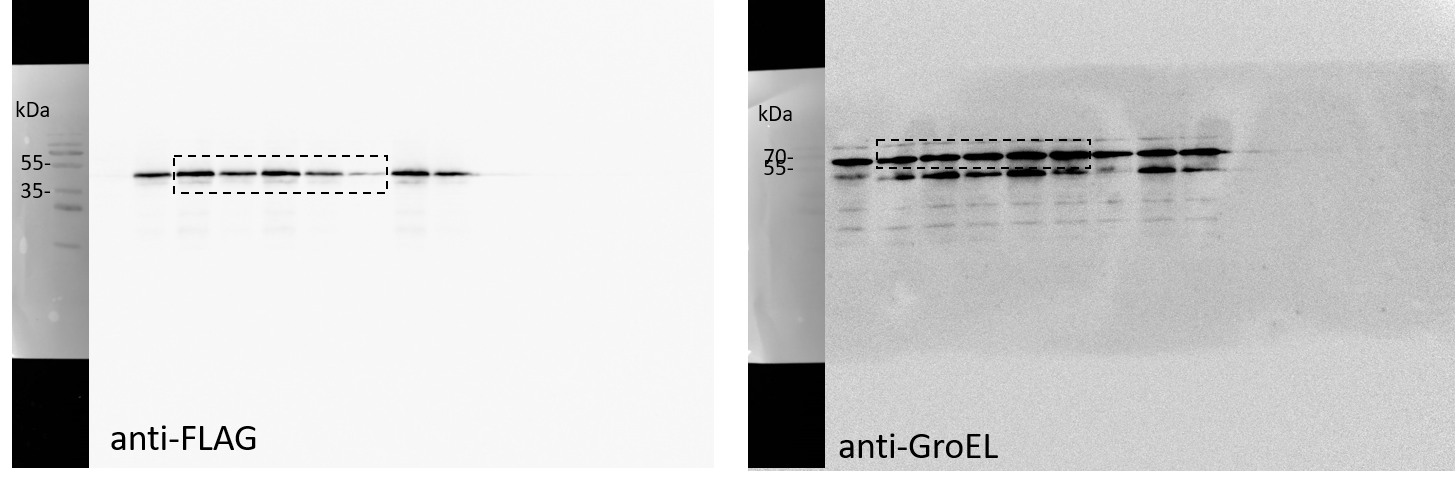


Western blot quantification raw values

|  |  | **PtmG-3xFLAG** |
| --- | --- | --- |
|  |  | **anti-FLAG** |
|  |  | **Intensity-Bkg [%]** |
| PtmG-3xFLAG |  | 12.49139885 |
| PtmG-3xFLAG | Δ180/190 | 18.80379343 |
| PtmG-3xFLAG | C-180/190 | 11.36703739 |
| PtmG-3xFLAG | C-180(Proc) | 17.61512838 |
| PtmG-3xFLAG | C-190(Proc) | 8.945460647 |

NB187


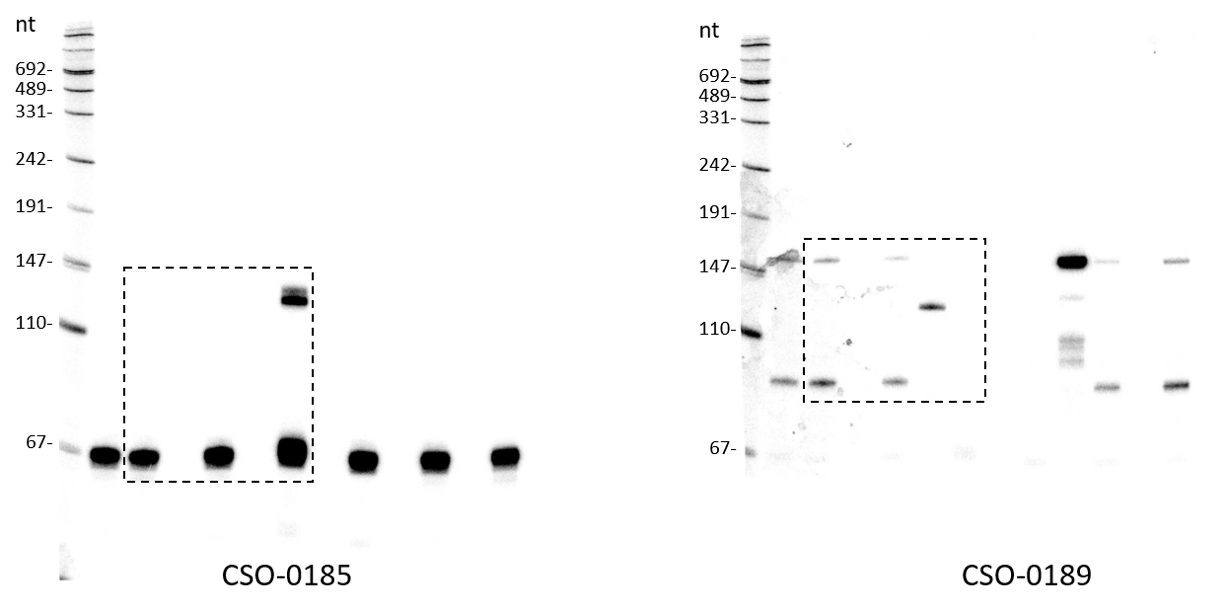


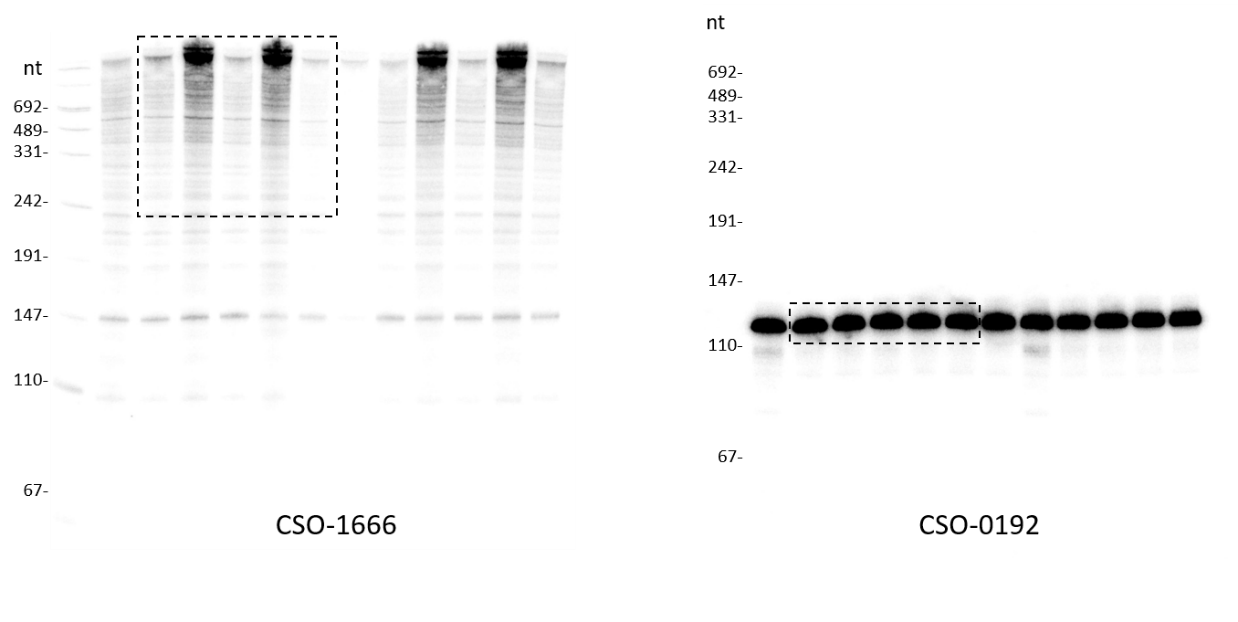


**Panel B**


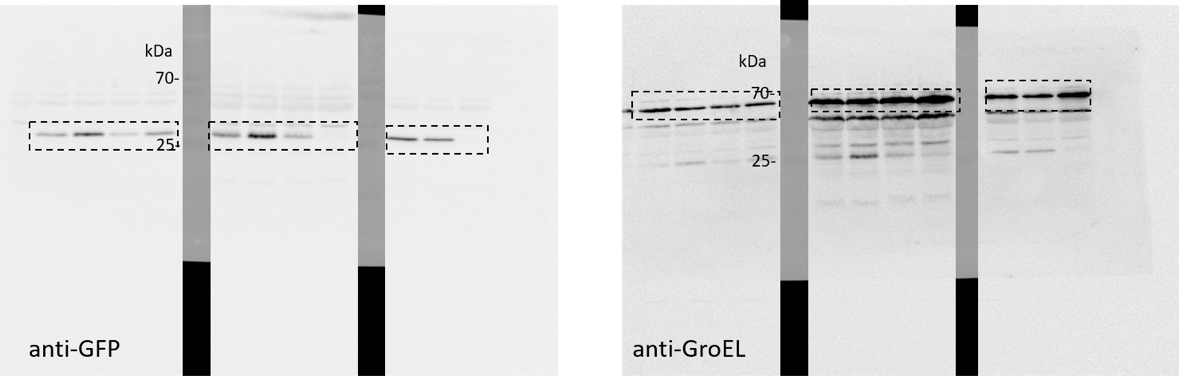


Western blot quantification raw values

|  |  |  | **PtmG-GFP** |
| --- | --- | --- | --- |
|  |  |  | **anti-GFP** |
| Promoter | UTR |  | **Intensity-Bkg [%]** |
| PptmG | *ptmG* |  | 6.656977374 |
|  |  | Δ180/90 | 14.49013607 |
|  |  | C-190(Proc) | 3.375073672 |
|  |  | Δ*fliA* | 7.413540937 |
| P*flaA* | *ptmG* |  | 12.20581208 |
|  |  | Δ180/90 | 23.35009876 |
|  |  | C-190(Proc) | 7.442297505 |
|  |  | Δ*fliA* | 1.467606121 |
| PflaA | *flaA* |  | 13.47294336 |
|  |  | Δ180/90 | 9.872619068 |
|  |  | Δ*fliA* | 0.252895058 |

NB77


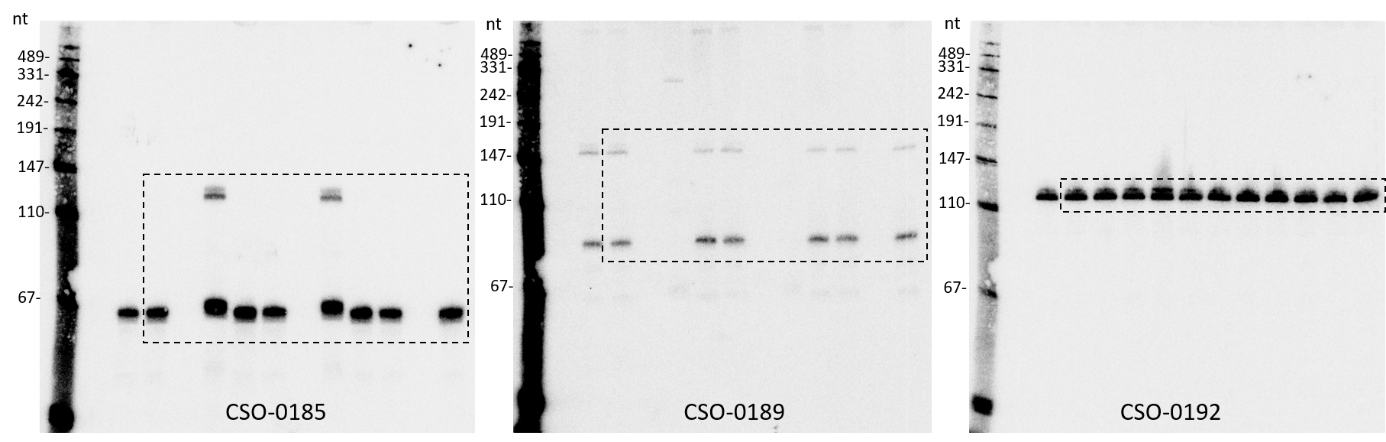

Supplement: Figure 1—figure supplement 4—source data 1. [file elife-69064-fig1-figsupp4-data1.zip › Source data - Figure 1 - figure supplement 4 - Source Data 1/Source data - Figure 1 - Figure supplement 4.docx]
