# Supplementary material for: RNase III-mediated processing of a trans-acting bacterial sRNA and its cis-encoded antagonist
Source: eLife. 2021 Nov 29;10:e69064. doi: 10.7554/eLife.69064 (PMC8687705; doi:10.7554/eLife.69064)
Supplement: Figure 2—source data 1. [file elife-69064-fig2-data1.zip › Source data - Figure 2/Source data - Figure 2.docx]

**Source data for Figure 2**

**Panel B**

**
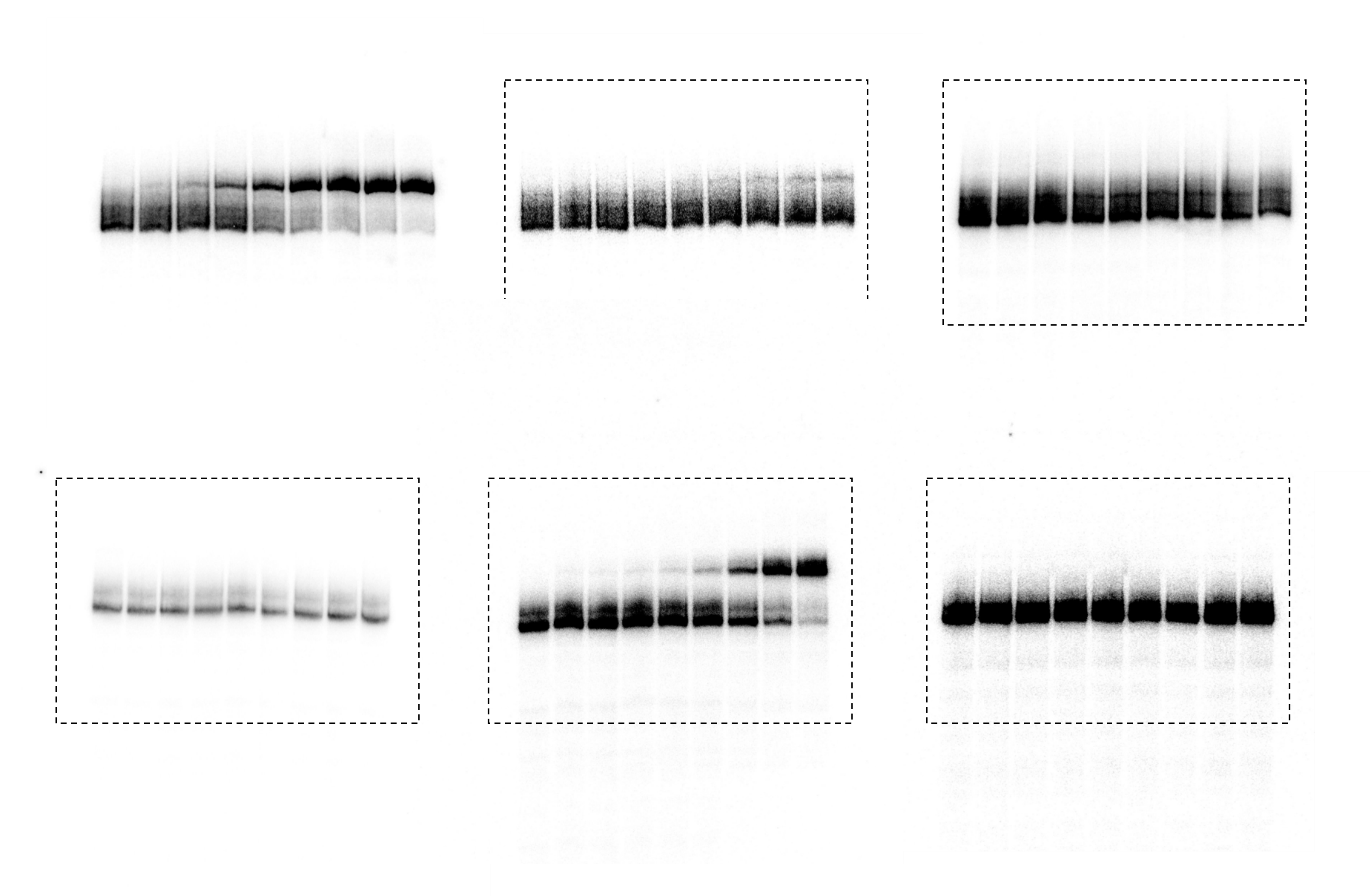
**

**Panel C**


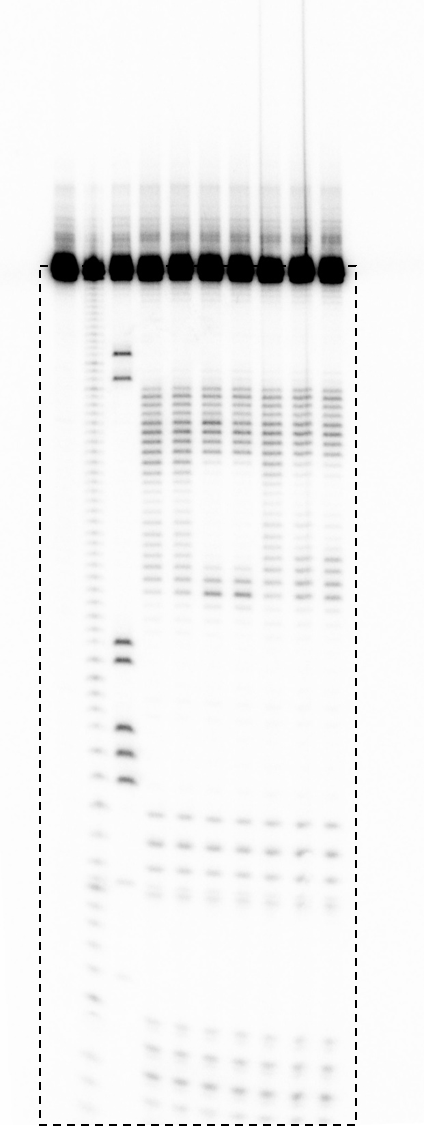


**Panel D**

**
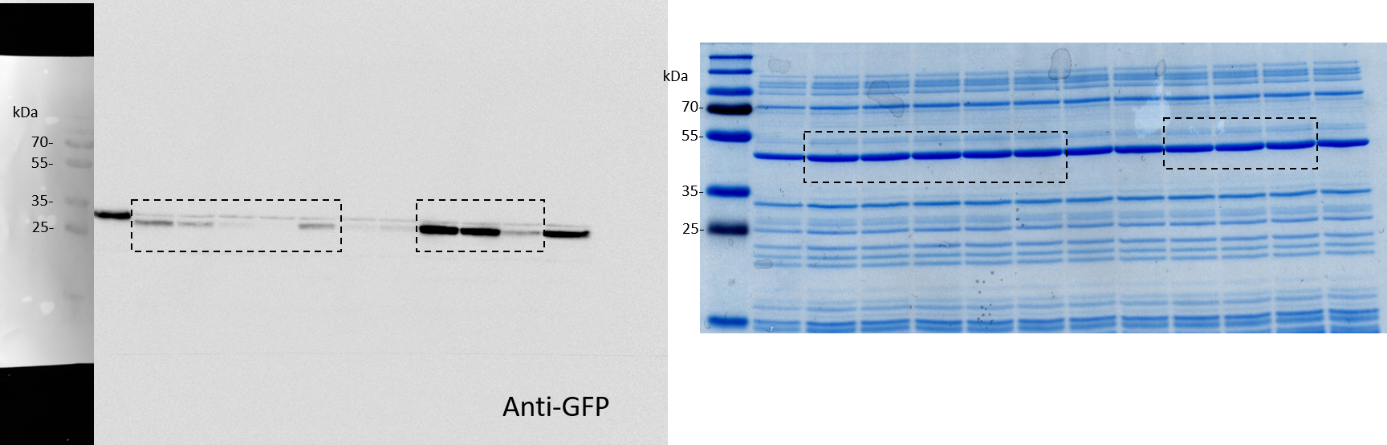
**

Western blot raw values

|  |  | **anti-GFP** |
| --- | --- | --- |
| **template** | **sRNA** | **Intensity-Bkg [%]** |
| *ptmG*-GFP WT | - | 4.849070944 |
| *ptmG*-GFP WT | CJnc190 WT 2 pmol | 2.196417654 |
| *ptmG*-GFP WT | CJnc190 WT 20 pmol | 0.532153696 |
| *ptmG*-GFP WT | CJnc190 WT 100 pmol | 0.14246316 |
| *ptmG*-GFP WT | CJnc190 M1 100 pmol | 3.019941652 |
| *ptmG*-GFP M1' | - | 25.41724063 |
| *ptmG*-GFP M1' | CJnc190 M1 100 pmol | 3.826230249 |
| *ptmG*-GFP M1' | CJnc190 WT 100 pmol | 22.73592013 |

**Panel E**

Western blot raw values

|  |  |  | **anti-GFP** | | |
| --- | --- | --- | --- | --- | --- |
|  |  |  | **Intensity-Bkg [%]** | | |
|  |  |  | **R1** | **R2** | **R3** |
| WT | - | *ptmG*-GFP WT | 9.7400 | 11.6100 | 6.7700 |
| Δ180/190 | - | *ptmG*-GFP WT | 23.6800 | 21.7100 | 14.5900 |
| WT | - | *ptmG*-GFP M1' | 15.3900 | 17.5000 | 14.7300 |
| Δ180/190 | - | *ptmG*-GFP M1' | 20.0600 | 20.4900 | 19.0400 |
| Δ180/190 | *rdxA*-CJnc190(Proc) WT | *ptmG*-GFP WT | 5.5200 | 4.0800 | 1.3400 |
| Δ180/190 | *rdxA*-CJnc190(Proc) M1 | *ptmG*-GFP WT | 9.3300 | 10.5100 | 5.2800 |
| Δ180/190 | *rdxA*-CJnc190(Proc) WT | *ptmG*-GFP M1' | 11.8500 | 8.7600 | 10.2200 |
| Δ180/190 | *rdxA*-CJnc190(Proc) M1 | *ptmG*-GFP M1' | 4.4200 | 5.3200 | 4.5400 |
