# Supplementary material for: RNase III-mediated processing of a trans-acting bacterial sRNA and its cis-encoded antagonist
Source: eLife. 2021 Nov 29;10:e69064. doi: 10.7554/eLife.69064 (PMC8687705; doi:10.7554/eLife.69064)

**Source data for Figure 2 - figure supplement 1**

Panel A


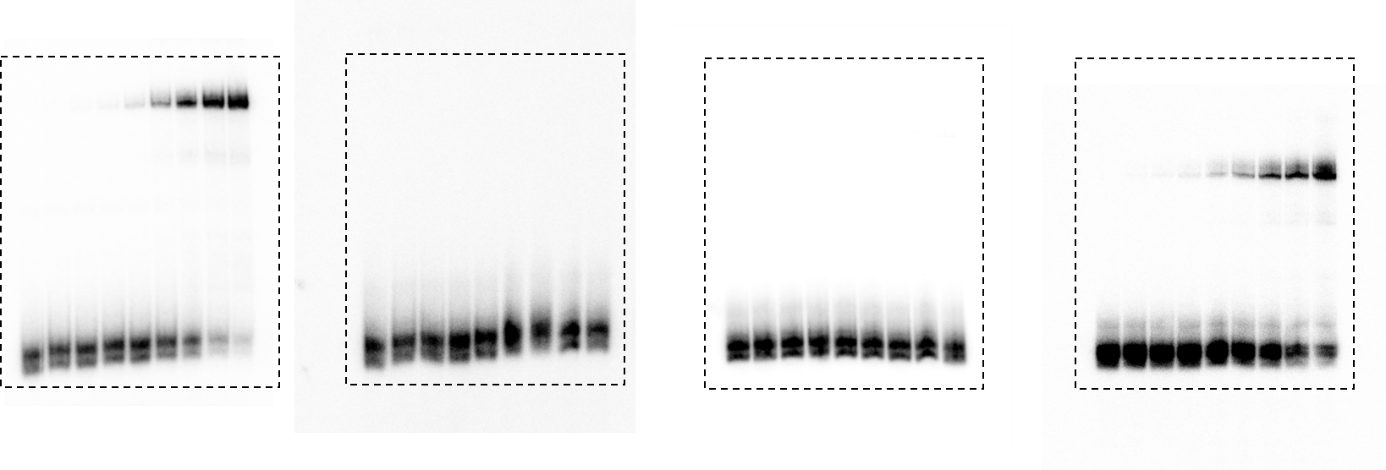


Panel B


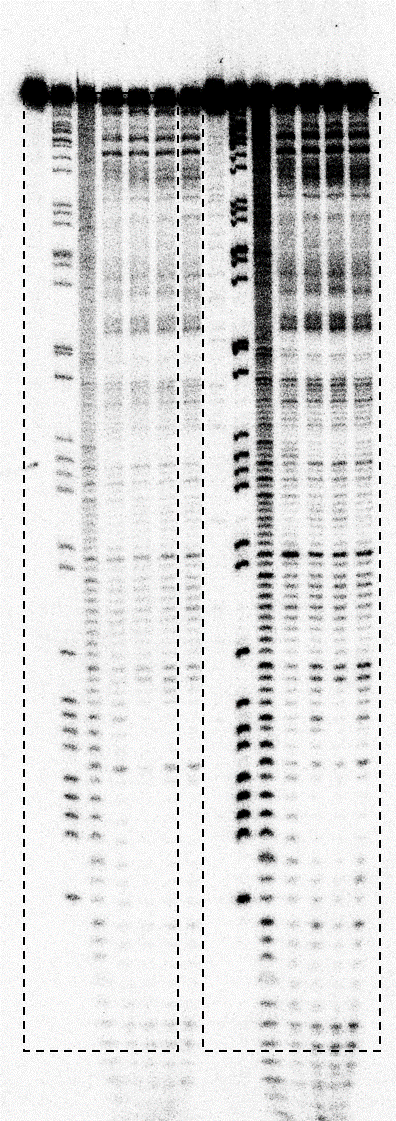


**Panel C**


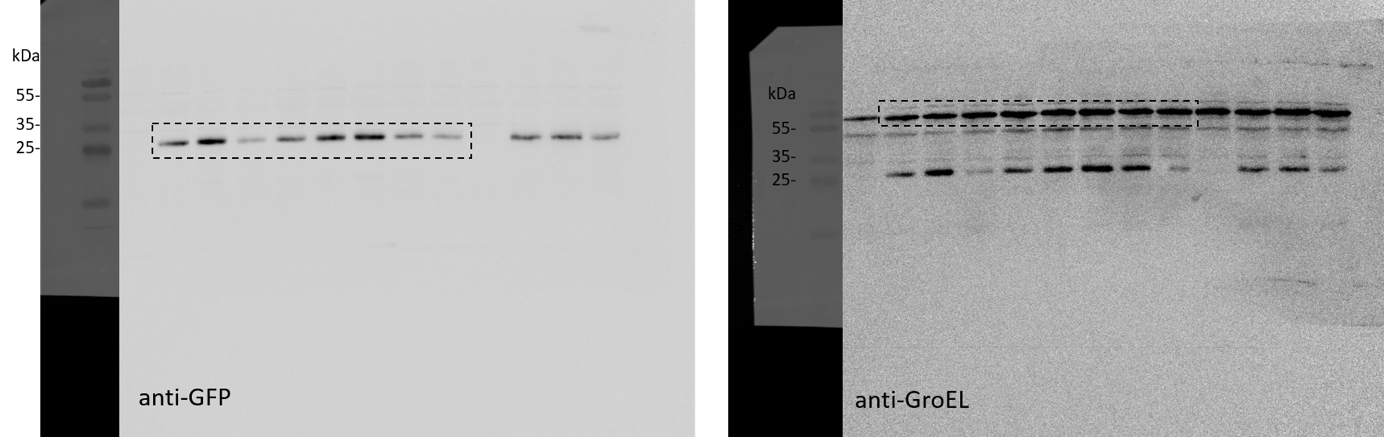


Western blot quantification raw values

|  |  | **PtmG(10th)-GFP** |
| --- | --- | --- |
|  |  | **anti-GFP** |
|  |  | **Intensity-Bkg [%]** |
| *ptmG*(10th)-GFP WT |  | 11.61263366 |
| *ptmG*(10th)-GFP WT | Δ180/190 | 21.71379316 |
| *ptmG*(10th)-GFP WT | C-180/190 WT | 4.083465287 |
| *ptmG*(10th)-GFP WT | C-180/190 M1 | 10.50785125 |
| *ptmG*(10th)-GFP M1' |  | 17.50379432 |
| *ptmG*(10th)-GFP M1' | Δ180/190 | 20.49273826 |
| *ptmG*(10th)-GFP M1' | C-180/190 WT | 8.763825775 |
| *ptmG*(10th)-GFP M1' | C-180/190 M1 | 5.321898284 |

NB71


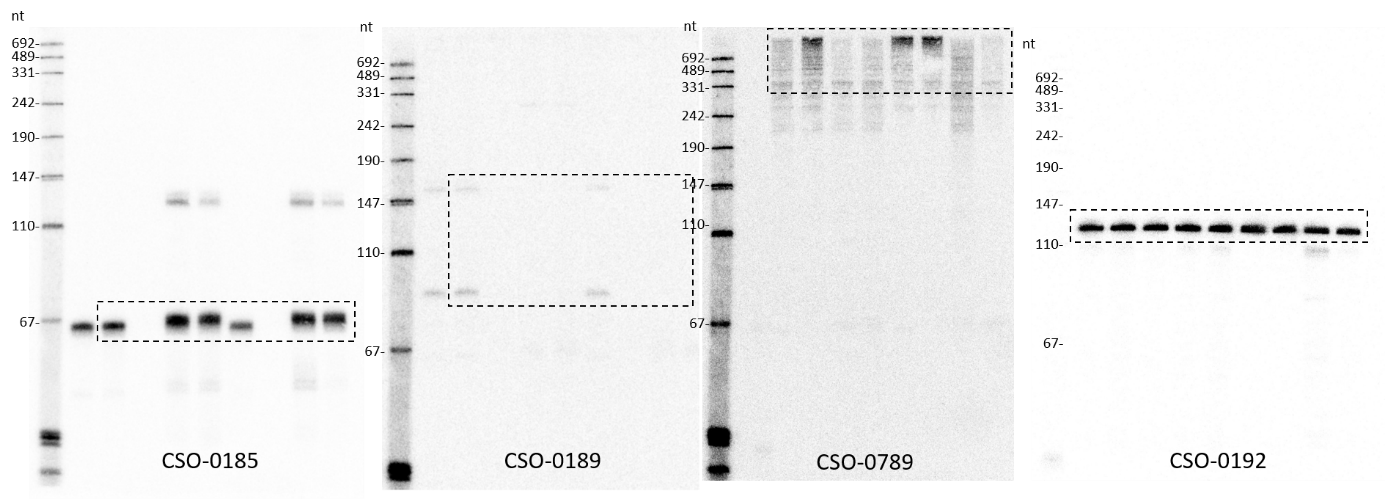

Supplement: Figure 2—figure supplement 1—source data 1. [file elife-69064-fig2-figsupp1-data1.zip › Source data - Figure 2 - figure supplement 1/Source data - Figure 2 - Figure supplement 1.docx]
