# Supplementary material for: RNase III-mediated processing of a trans-acting bacterial sRNA and its cis-encoded antagonist
Source: eLife. 2021 Nov 29;10:e69064. doi: 10.7554/eLife.69064 (PMC8687705; doi:10.7554/eLife.69064)

**Source data for Figure 3**

**Panel A**

NB119

**
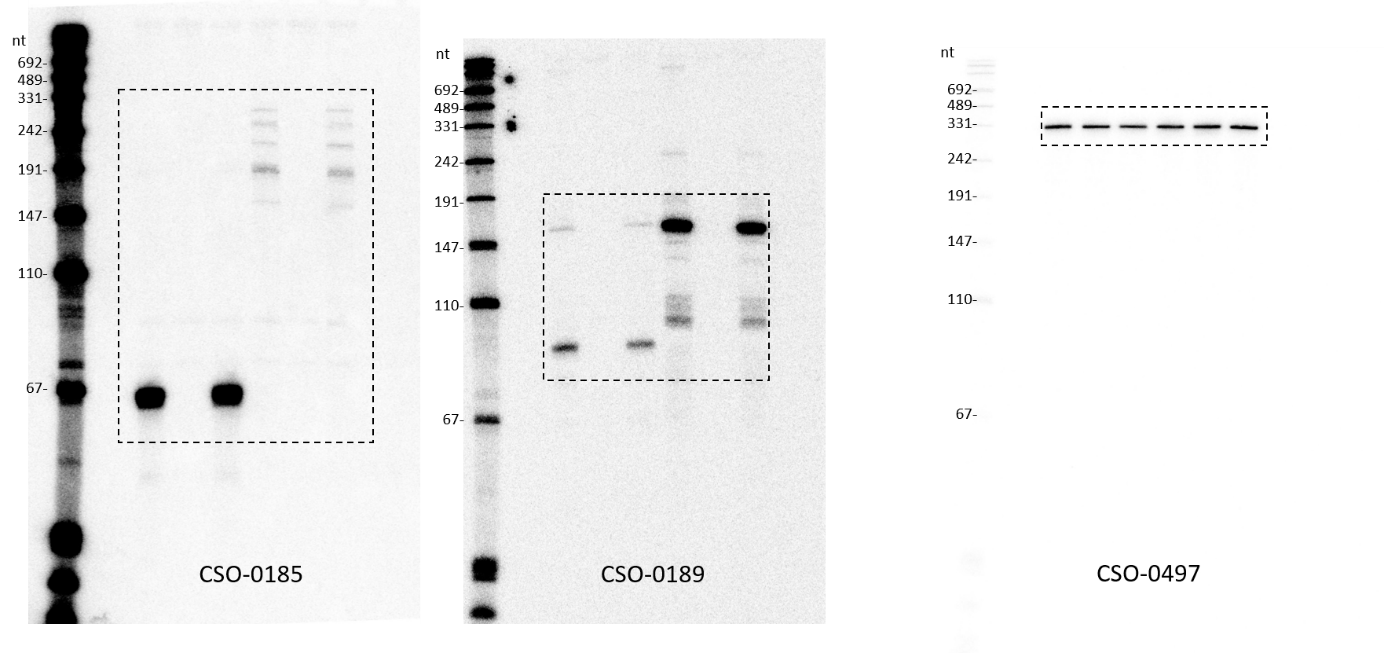
**

Northern blot quantification raw values

|  | **CJnc190 whole lane** |
| --- | --- |
|  | **CSO-0185** |
|  | **Intensity-Bkg [%]** |
| **WT** | 46.40729084 |
| **Δ180/190** | 1.080800971 |
| **C-180/190** | 42.2377058 |
| **WT** | 5.247965887 |
| **Δ180/190** | 0.131583734 |
| **C-180/190** | 4.894652769 |

**Panel B**

|  |  | **PtmG-3xFLAG** | | | | | |
| --- | --- | --- | --- | --- | --- | --- | --- |
|  |  | **anti-FLAG** | | | | | |
|  |  | **R1** | | | **R2** | | |
|  |  | **tech 1** | **tech 2** | **average** | **tech 1** | **tech 2** | **average** |
| PtmG-3xFLAG | - | 5.519941 | 8.705629 | 7.112785 | 7.003229 | 8.705629 | 7.854429 |
| PtmG-3xFLAG | Δ180/190 | 12.32333 | 15.28671 | 13.80502 | 11.05895 | 15.28671 | 13.17283 |
| PtmG-3xFLAG | C-190(Proc) | 4.243869 | 4.303177 | 4.273523 | 2.95707 | 4.303177 | 3.630124 |
| PtmG-3xFLAG | Δ*rnc* | 11.24299 | 11.47009 | 11.35654 | 7.459262 | 11.47009 | 9.464676 |
| PtmG-3xFLAG | Δ180/190 Δ*rnc* | 14.66204 | 12.48951 | 13.57577 | 11.74947 | 12.48951 | 12.11949 |
| PtmG-3xFLAG | C-190(Proc) Δrnc | 3.524842 | 3.336289 | 3.430566 | 4.180616 | 3.336289 | 3.758453 |

**Panel C**

**
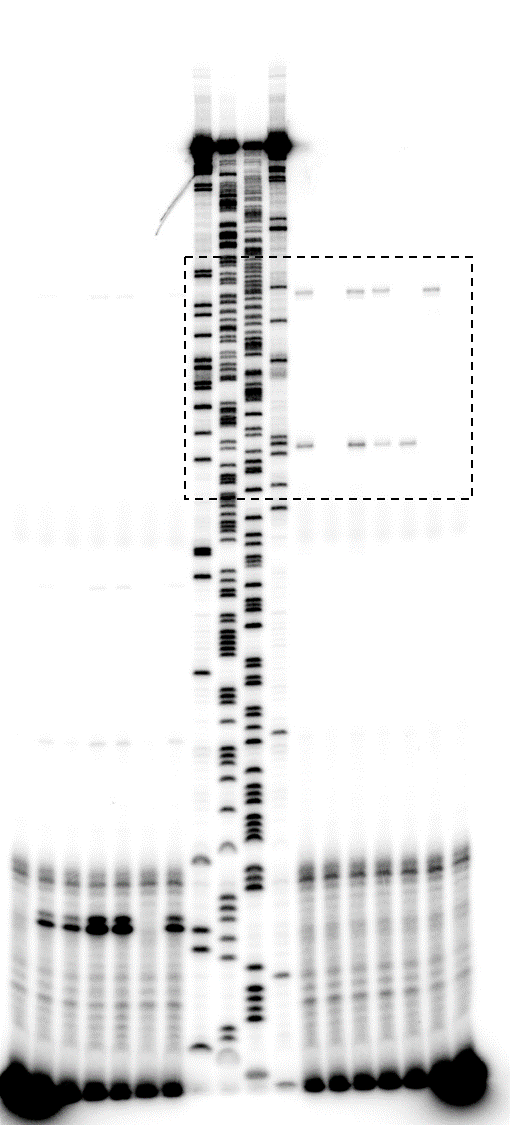
**

**Panel D**

NB124


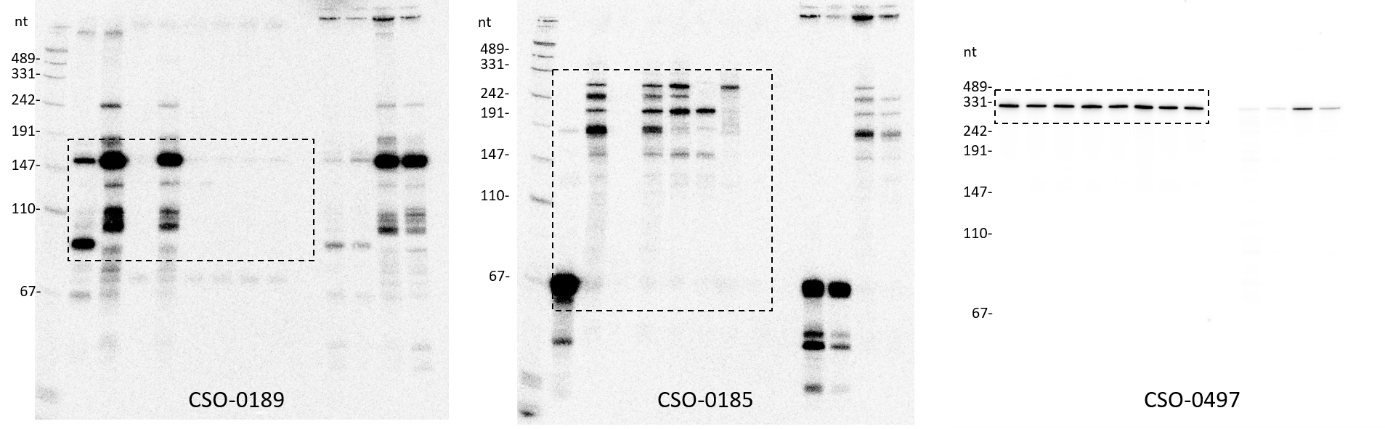

Supplement: Figure 3—source data 1. [file elife-69064-fig3-data1.zip › Source data - Figure 3/Source data - Figure 3.docx]
