# Supplementary material for: RNase III-mediated processing of a trans-acting bacterial sRNA and its cis-encoded antagonist
Source: eLife. 2021 Nov 29;10:e69064. doi: 10.7554/eLife.69064 (PMC8687705; doi:10.7554/eLife.69064)
Supplement: Figure 3—figure supplement 1—source data 1. [file elife-69064-fig3-figsupp1-data1.zip › Source data - Figure 3 - figure supplement 1 - Source Data 1/Source data - Figure 3 - Figure supplement 1.docx]

**Source data for Figure 3 – Figure supplement 1**

**Panel A**

NB BA88

**
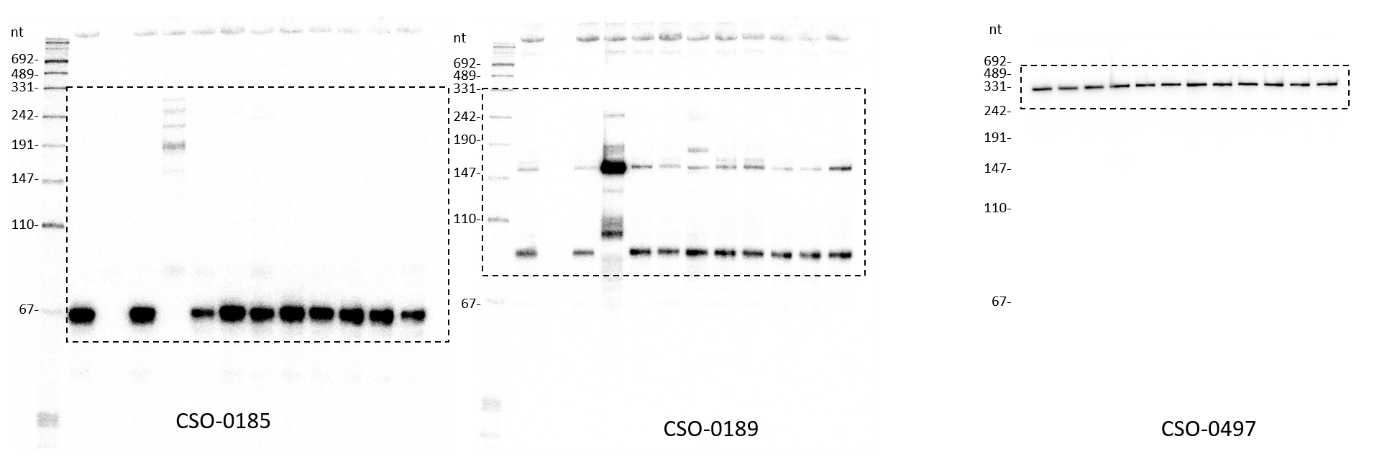
**

**Panel B**

**
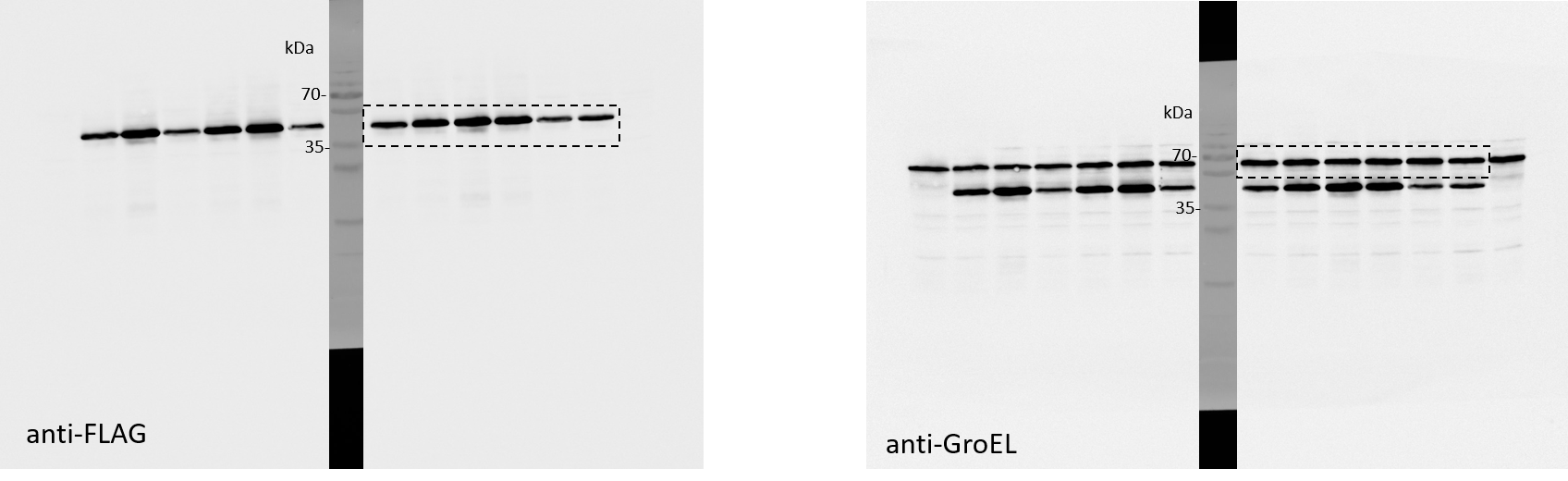
**

Western blot quantification raw values

|  | **PtmG-3xFLAG** |
| --- | --- |
|  | **anti-FLAG** |
|  | **Intensity-Bkg [%]** |
| WT | 6.455667641 |
| Δ*rnc* | 10.88086892 |
| Δ180/190 | 12.52153282 |
| Δ180/190 Δ*rnc* | 10.47306442 |
| C-190(Proc) | 3.65756295 |
| C-190(Proc) Δ*rnc* | 4.494295811 |

NB143

**
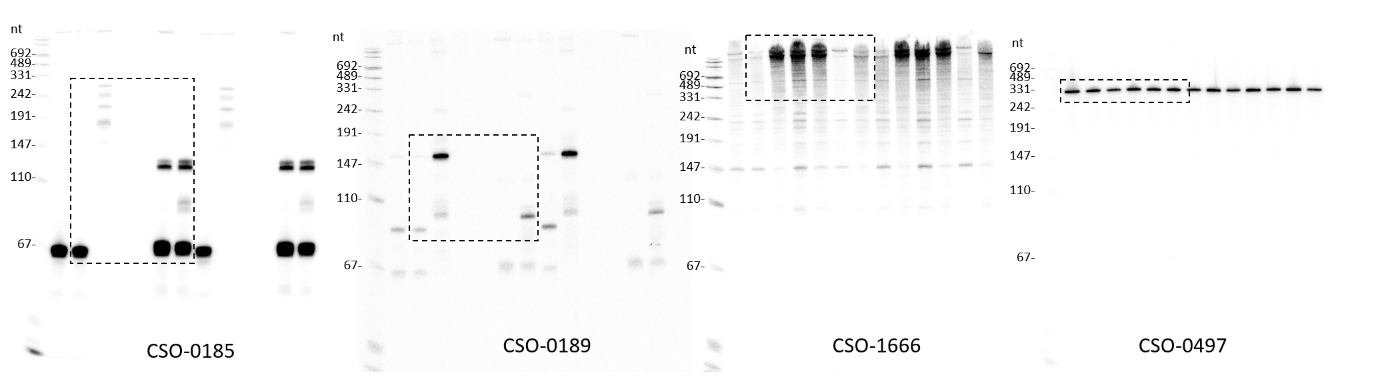
**

**Panel C**

NB199

|  | **WT (mature CJnc190) (CSO-0185)** | | | | | | **Δ*rnc* (most abundant) (CSO-0185)** | | | | | |
| --- | --- | --- | --- | --- | --- | --- | --- | --- | --- | --- | --- | --- |
|  | **Time after Rifampicin addition** | | | | | | | | | | | |
|  | **2'** | **4'** | **8'** | **16'** | **32'** | **64'** | **2'** | **4'** | **8'** | **16'** | **32'** | **64'** |
| **R1** | 18.12054 | 17.8628 | 20.02778 | 11.01008 | 14.57094 | 18.40786 | 48.93843 | 10.50089 | 7.67544 | 17.23378 | 11.87569 | 3.775774 |
| **R2** | 16.66518 | 31.11478 | 23.28152 | 6.349035 | 14.14501 | 8.44448 | 45.48093 | 19.90978 | 15.67253 | 10.22547 | 6.044944 | 2.66636 |

**
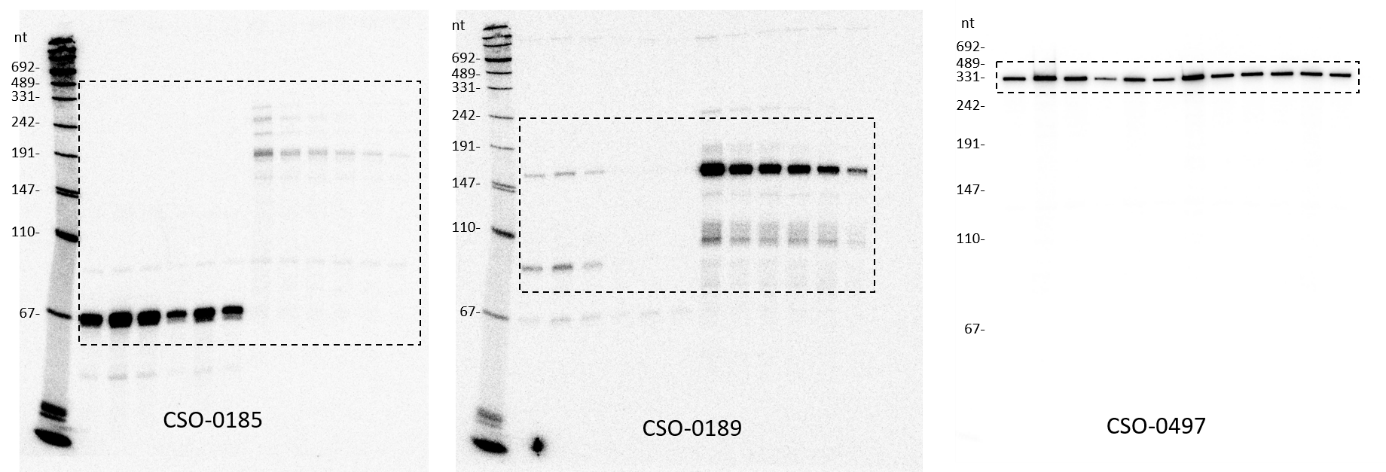
**

Northern blot quantification raw data
