# Supplementary material for: RNase III-mediated processing of a trans-acting bacterial sRNA and its cis-encoded antagonist
Source: eLife. 2021 Nov 29;10:e69064. doi: 10.7554/eLife.69064 (PMC8687705; doi:10.7554/eLife.69064)

**Source data for Figure 4**

**Panel A**

NB125


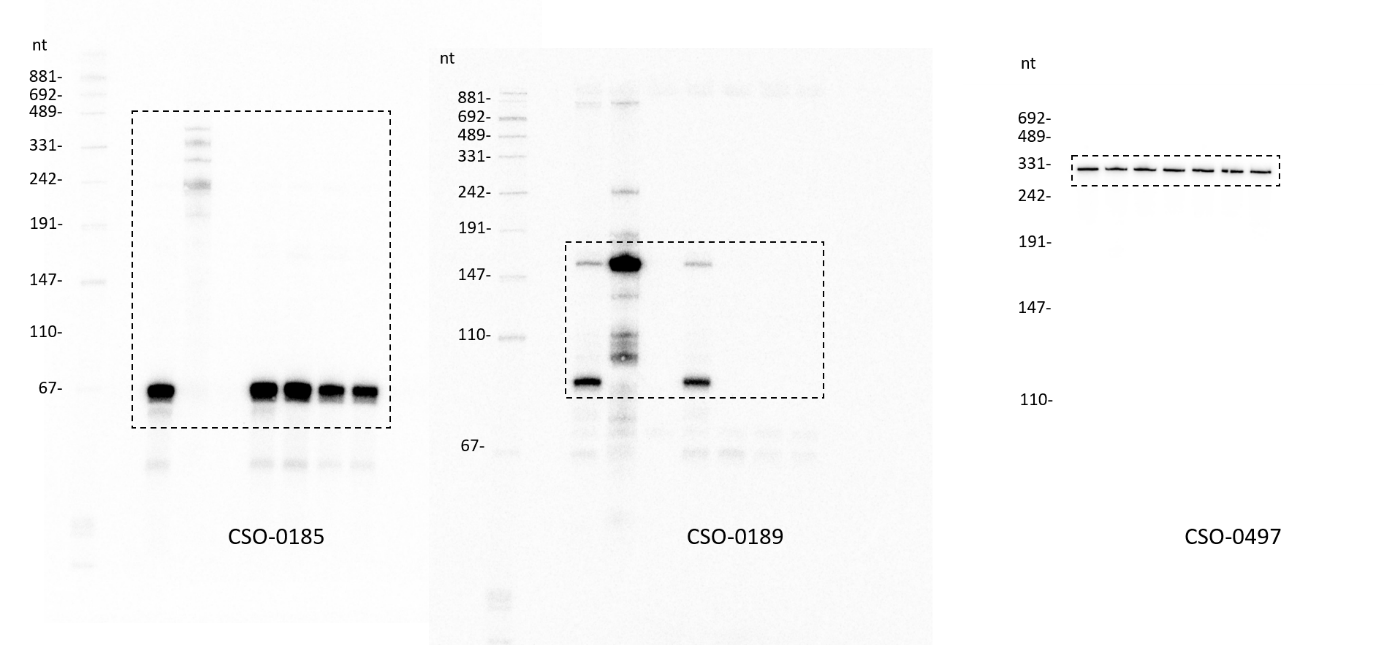


Northern blot quantification raw values

|  | **CJnc190 (mature)** |
| --- | --- |
|  | **CSO-0185** |
|  | **Intensity-Bkg [%]** |
| WT | 21.41504537 |
| C-180/190 | 25.64225436 |
| C-190 only | 27.56699731 |
| C-190-P2 only | 13.19840882 |
| C-190-P1 only | 12.17729415 |

**Panel B**

NB126


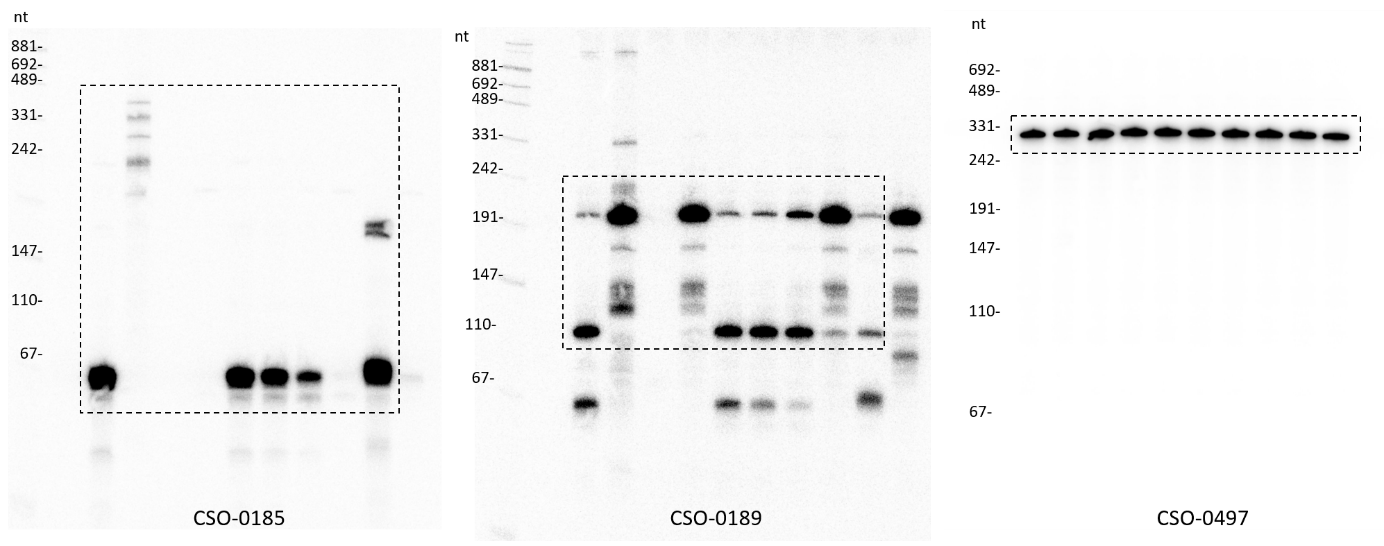

Supplement: Figure 4—source data 1. [file elife-69064-fig4-data1.zip › Source data - Figure 4/Source data - Figure 4.docx]
