# Supplementary material for: RNase III-mediated processing of a trans-acting bacterial sRNA and its cis-encoded antagonist
Source: eLife. 2021 Nov 29;10:e69064. doi: 10.7554/eLife.69064 (PMC8687705; doi:10.7554/eLife.69064)
Supplement: Figure 7—source data 1. [file elife-69064-fig7-data1.zip › Source data - Figure 7/Source data - Figure 7.docx]

**Source data for Figure 7**

**Panel A**

Western blot quantification raw values

|  | **PtmG-3xFLAG** | | | |
| --- | --- | --- | --- | --- |
|  | **anti-FLAG** | | | |
|  | **Intensity-Bkg [%]** | | | |
|  | **R2** | **R4** | **R5** | **R6** |
| **WT** | 7.27198574 | 9.33588195 | 8.42134231 | 7.45260327 |
| **Δ180/190** | 14.781051 | 19.1331433 | 16.7272554 | 17.4943978 |
| **OE-180** | 12.5278243 | 11.9377518 | 14.5095229 | 10.9857215 |
| **OE-180(Proc)** | 12.6033985 | 12.4089634 | 14.0030568 | 10.4061001 |

**Panel B**

Western blot quantification raw values

|  | |  | | **PtmG-3xFLAG** | |
| --- | --- | --- | --- | --- | --- |
|  | |  | | **anti-FLAG** | |
|  |  | **R1** | **R2** | **R3** | **R4** |
| PtmG-3F | WT | 6.7600 | 5.5000 | 5.2900 | 4.9714 |
| PtmG-3F | Δ180/190 | 10.4500 | 10.8700 | 8.5700 | 6.4098 |
| PtmG-3F | C-180/190 | 5.4800 | 6.3700 | 5.0700 | 4.2250 |
| PtmG-3F | C-190 only | 4.6300 | 6.0700 | 4.7300 | 4.3604 |
| PtmG-3F | C-190 P2 + 180 | 12.6900 | 11.1600 | 9.8200 | 6.8009 |
| PtmG-3F | C-190 P2 | 8.2400 | 6.4200 | 7.5800 | 5.8854 |
| PtmG-3F | C-190 P1 + 180 | 7.4900 | 5.4600 | 8.7900 | 9.5607 |
| PtmG-3F | C-190 P1 | 9.6600 | 7.9800 | 8.0600 | 5.6098 |
| PtmG-3F | C-3xmut | 13.0900 | 12.0500 | 11.1800 | 7.0781 |

**Panel C**

NB132


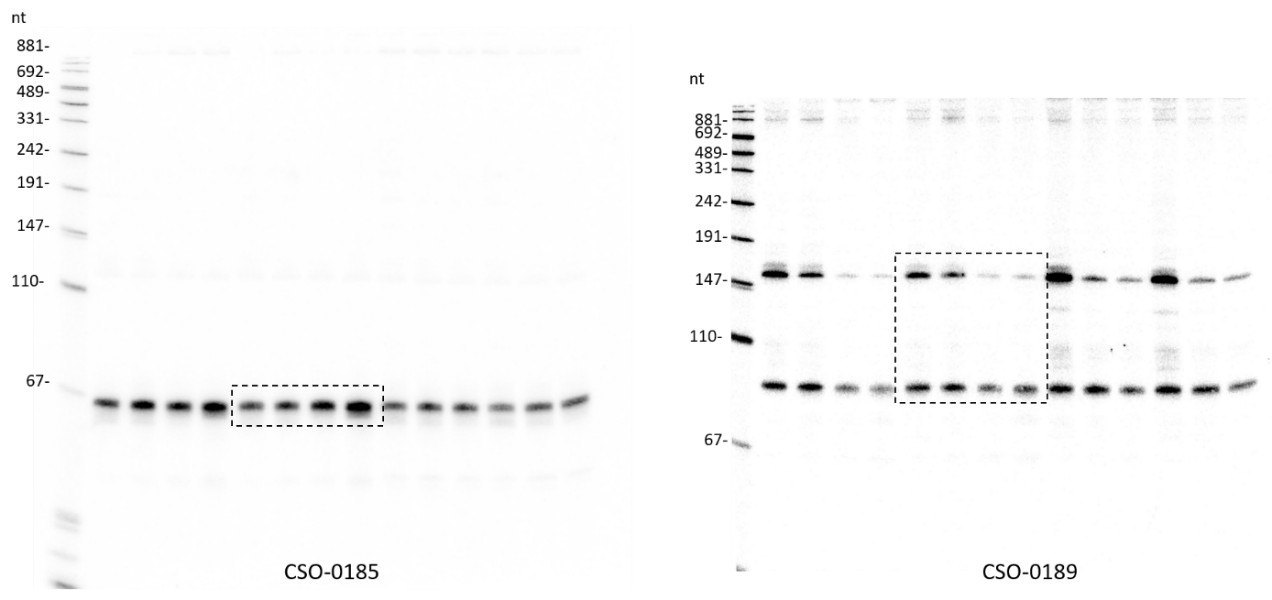


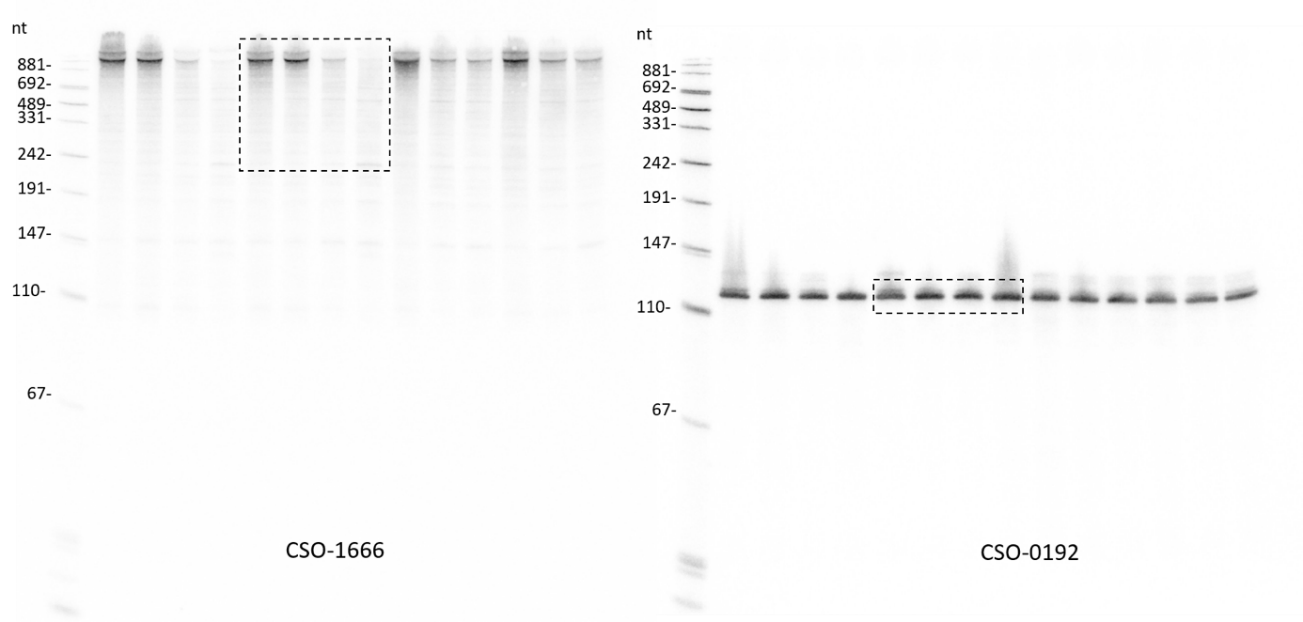


**Northern blot quantification raw values**

|  |  | **CJnc190 (mature)** | **pre-CJnc180** | **CJnc180 (mature)** | ***ptmG* mRNA** |
| --- | --- | --- | --- | --- | --- |
|  |  | **CSO-0185** | **CSO-0189** | **CSO-0189** | **CSO-1666** |
|  | **OD600** | **Intensity-Bkg [%]** | | | |
| **WT** | **0.1** | 17.46362749 | 20.13632249 | 18.00085642 | 45.48411418 |
| **WT** | **0.25** | 18.7099074 | 12.87282231 | 17.72184119 | 41.55266201 |
| **WT** | **0.5** | 25.32985733 | 2.452463233 | 10.90842262 | 8.9300634 |
| **WT** | **0.8** | 38.49660778 | 3.206784266 | 14.70048747 | 4.033160413 |

**Panel D**

|  |  | **PtmG-3xFLAG** | | |
| --- | --- | --- | --- | --- |
|  |  | **anti-FLAG** | | |
|  |  | **Intensity-Bkg [%]** | | |
|  |  | **R1** | **R2** | **R3** |
| **Δ180/190** | **0.25** | 14.42764256 | 6.796167923 | 11.66376855 |
| **C-180/190** |  | 5.220215686 | 6.947851237 | 6.176886144 |
| **C-190 only** |  | 4.449499996 | 6.398539864 | 6.355940682 |
| **OE-180(Proc)** |  | 4.096550798 | 8.227233953 | 6.686987044 |
| **Δ180/190** | **0.5** | 12.21462492 | 13.45760485 | 14.24625814 |
| **C-180/190** |  | 6.736782484 | 7.256972825 | 7.042237071 |
| **C-190 only** |  | 7.110139715 | 7.47010523 | 5.895625436 |
| **OE-180(Proc)** |  | 12.30876951 | 10.00241317 | 10.79775475 |
| **Δ180/190** | **0.8** | 13.97149383 | 11.80220154 | 13.09752644 |
| **C-180/190** |  | 8.15398757 | 6.442710434 | 6.587880714 |
| **C-190 only** |  | 3.115916866 | 5.839323947 | 4.585767066 |
| **OE-180(Proc)** |  | 8.19437607 | 9.358875032 | 6.86336797 |
