# Supplementary material for: RNase III-mediated processing of a trans-acting bacterial sRNA and its cis-encoded antagonist
Source: eLife. 2021 Nov 29;10:e69064. doi: 10.7554/eLife.69064 (PMC8687705; doi:10.7554/eLife.69064)
Supplement: Figure 7—figure supplement 1—source data 1. [file elife-69064-fig7-figsupp1-data1.zip › Source data - Figure 7 - figure supplement 1/Source data - Figure 7 - figure supplement 1.docx]

**Source data for Figure 7 – figure supplement 1**

**Panel A**


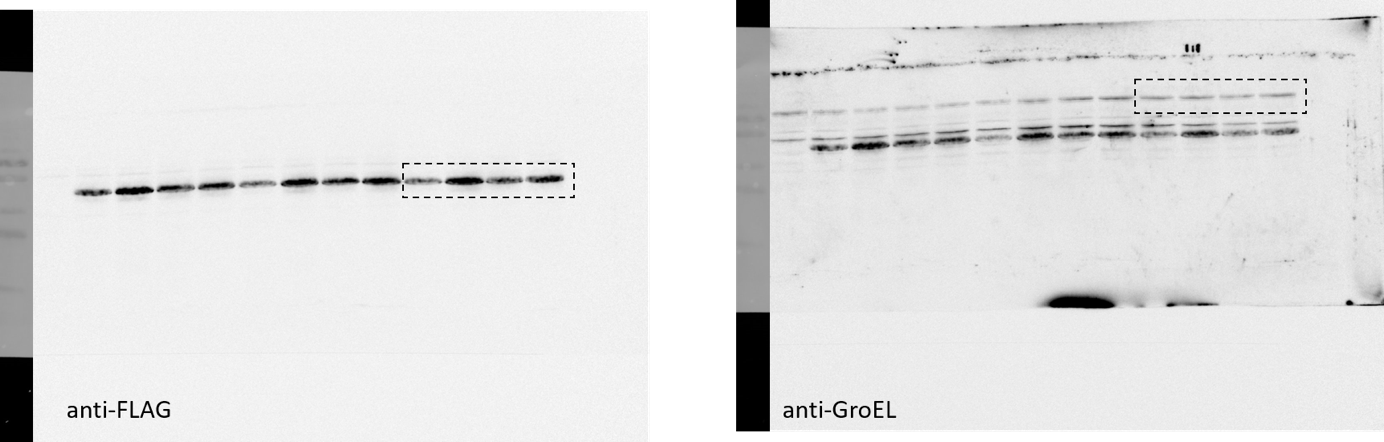


Western blot quantification raw values

|  | **PtmG-3xFLAG** |
| --- | --- |
|  | **anti-FLAG** |
|  | **Intensity-Bkg [%]** |
| **WT** | 4.63 |
| **Δ180/190** | 11.88 |
| **OE-180** | 6.42 |
| **OE-180(Proc)** | 8.10 |

NB122

**
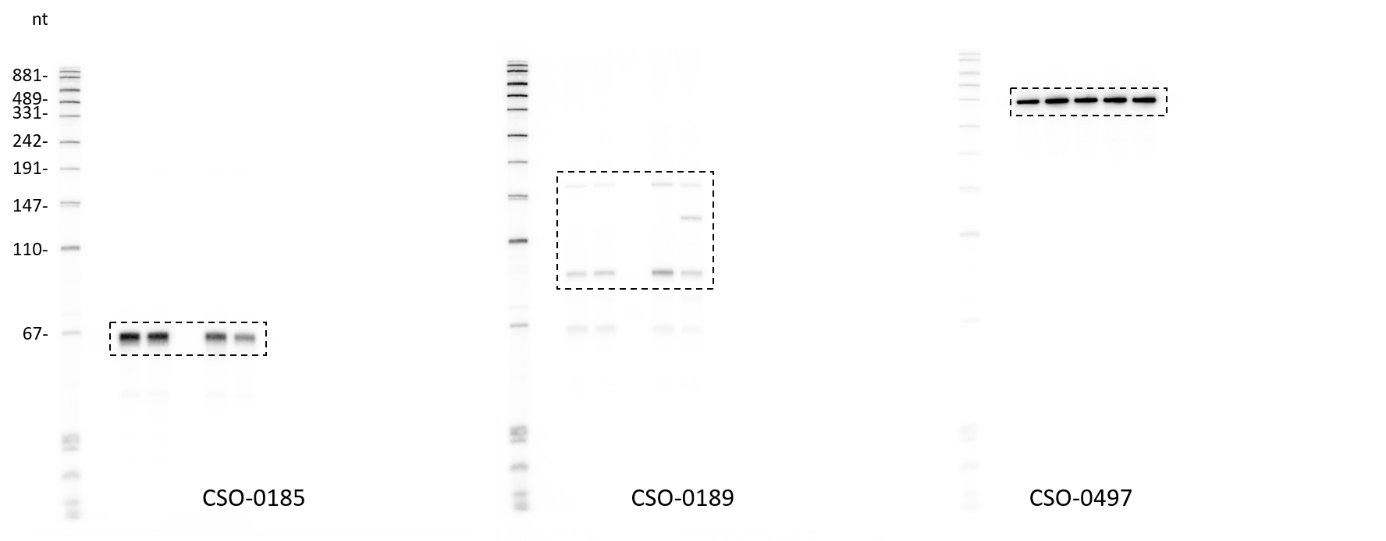
**

**Panel B**


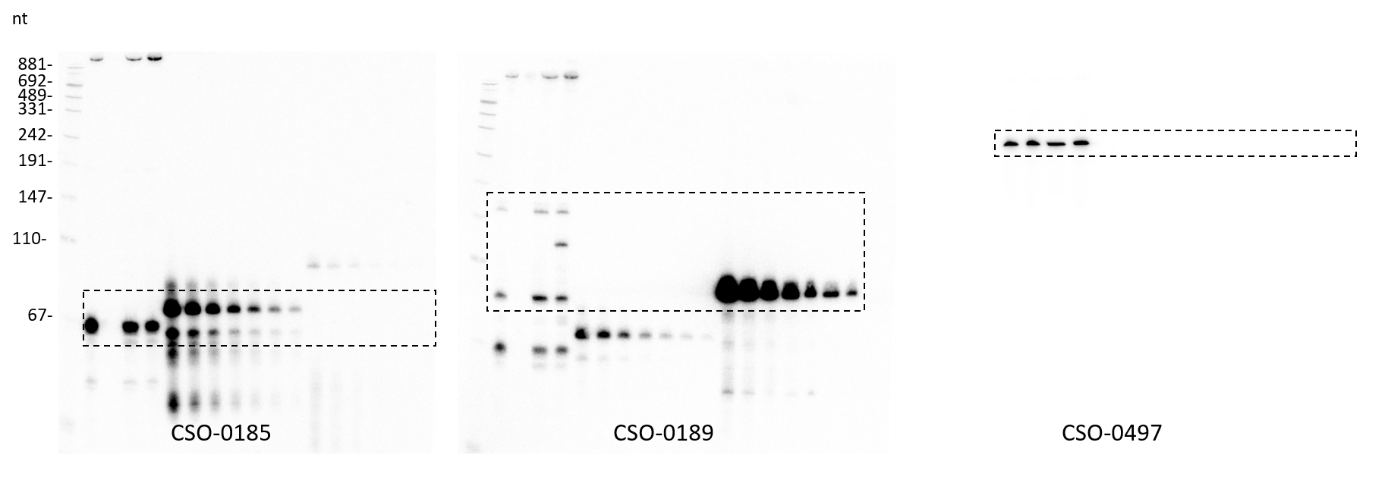


Northern blot quantification raw values

|  |  | **CJnc190 (approx 80-60 nt)** | **CJnc180 (mature + precursor, 160-90 nt)** |
| --- | --- | --- | --- |
|  |  | **CSO-0185** | **CSO-0189** |
|  |  | **Intensity-Bkg [%]** | |
| WT total RNA | 10 µg | 9.98 | 0.42 |
| D180/190 total RNA | 10 µg | - | - |
| OE-180 total RNA | 10 µg | 8.05 | 0.97 |
| OE-180(Proc) total RNA | 10 µg | 7.69 | 1.02 |
| CJnc190 in vitro transcript | 10 ng | 36.37 | - |
| CJnc190 in vitro transcript | 5 ng | 18.09 | - |
| CJnc190 in vitro transcript | 2.5 ng | 10.19 | - |
| CJnc190 in vitro transcript | 1.25 ng | 4.9 | - |
| CJnc190 in vitro transcript | 0.625 ng | 2.67 | - |
| CJnc190 in vitro transcript | 0.313 ng | 1.37 | - |
| CJnc190 in vitro transcript | 0.156 ng | 0.69 | - |
| CJnc180 in vitro transcript | 10 ng | - | 45.84 |
| CJnc180 in vitro transcript | 5 ng | - | 26.23 |
| CJnc180 in vitro transcript | 2.5 ng | - | 13.73 |
| CJnc180 in vitro transcript | 1.25 ng | - | 6.81 |
| CJnc180 in vitro transcript | 0.625 ng | - | 2.7 |
| CJnc180 in vitro transcript | 0.313 ng | - | 1.59 |
| CJnc180 in vitro transcript | 0.156 ng | - | 0 |

**Panel C**

Northern blot quantification raw values

|  |  | | | ***ptmG-3xFLAG* mRNA** | | |
| --- | --- | --- | --- | --- | --- | --- |
|  |  | | | **CSO-1666** | | |
|  |  | **R1** | **R2** | | **R3** | **R4** |
| PtmG-3F | WT | 3.9100 | 2.1100 | | 2.3300 | 5.8271 |
| PtmG-3F | Δ180/190 | 20.4400 | 19.8200 | | 25.6500 | 18.4769 |
| PtmG-3F | C-180/190 | 3.5000 | 2.0500 | | 2.0000 | 3.0489 |
| PtmG-3F | C-190 only | 22.1300 | 2.2500 | | 1.9300 | 3.6716 |
| PtmG-3F | C-190 P2 + 180 | 16.9800 | 10.9300 | | 8.2200 | 13.0763 |
| PtmG-3F | C-190 P2 | 10.5800 | 6.0900 | | 2.6700 | 5.2244 |
| PtmG-3F | C-190 P1 + 180 | 10.4300 | 8.4300 | | 5.3500 | 16.5261 |
| PtmG-3F | C-190 P1 | 5.8500 | 4.5500 | | 3.4500 | 7.8558 |
| PtmG-3F | C-3xmut | 2.5400 | 20.0800 | | 24.4300 | 20.7860 |

**Panel D**


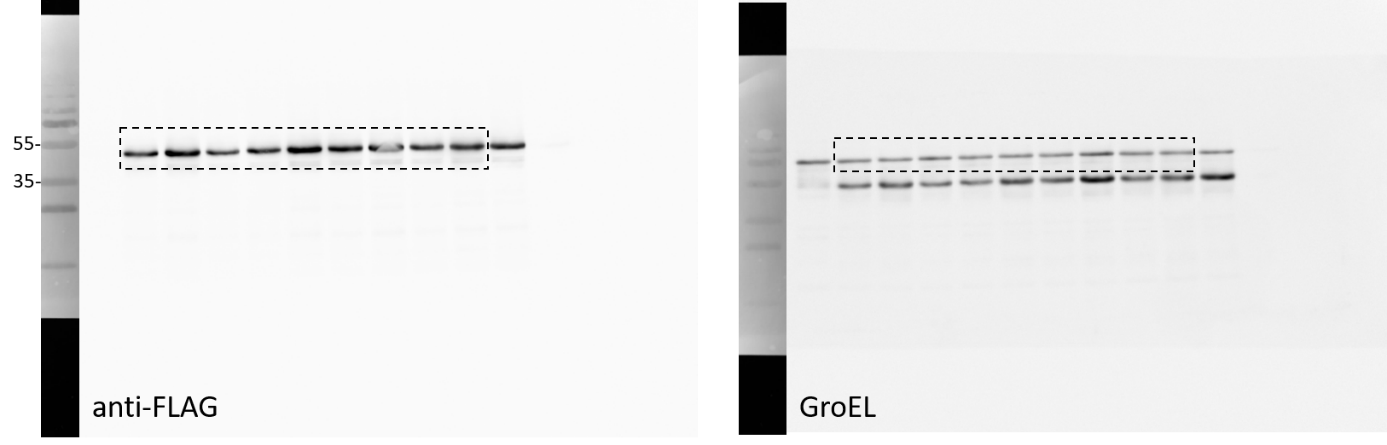


Western blot quantification raw values

|  |  | **PtmG-3xFLAG** | **GroEL** |
| --- | --- | --- | --- |
|  |  | **anti-FLAG** | **anti-GroEL** |
|  |  | **Intensity-Bkg [%]** | |
| **PtmG-3xFLAG** |  | 4.971350885 | 3.095344845 |
| **PtmG-3xFLAG** | **Δ180/190** | 6.409821248 | 2.759077755 |
| **PtmG-3xFLAG** | **C-180/190** | 4.225025729 | 3.209361844 |
| **PtmG-3xFLAG** | **C-190 only** | 4.360399366 | 2.758694851 |
| **PtmG-3xFLAG** | **C-180 + 190-P1** | 6.80086145 | 2.981101641 |
| **PtmG-3xFLAG** | **C-190-P1** | 5.88543428 | 3.088376851 |
| **PtmG-3xFLAG** | **C-180 + 190-P2** | 9.560708499 | 4.438116533 |
| **PtmG-3xFLAG** | **C-190-P2** | 5.609777522 | 3.504719813 |
| **PtmG-3xFLAG** | **C-3xmut** | 7.078128545 | 3.207950558 |

NB213


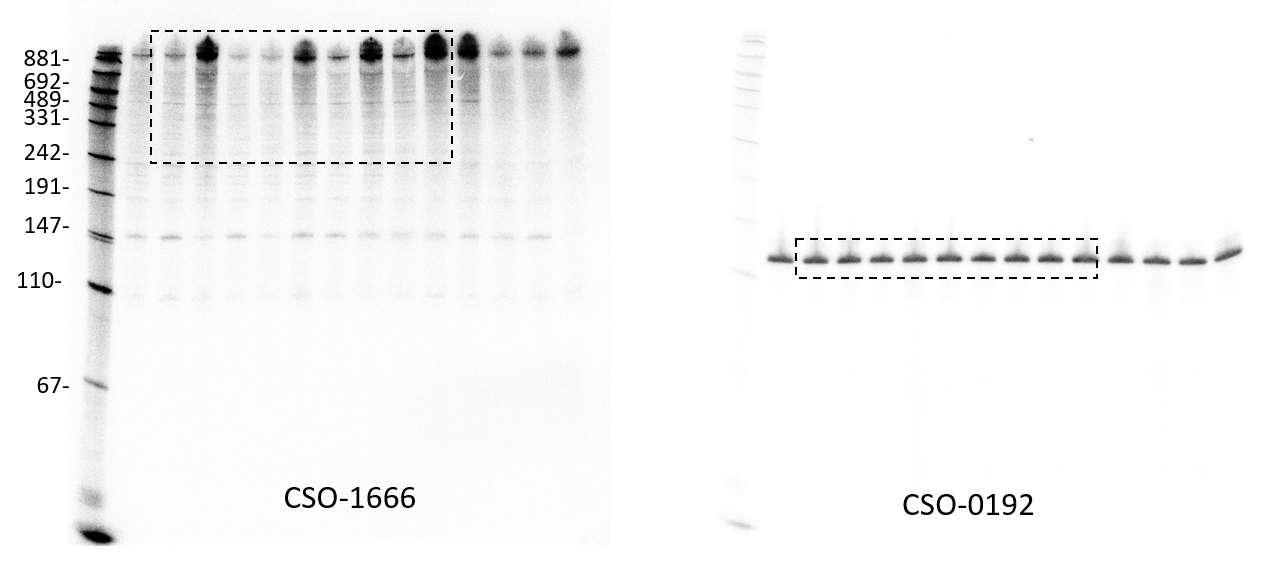


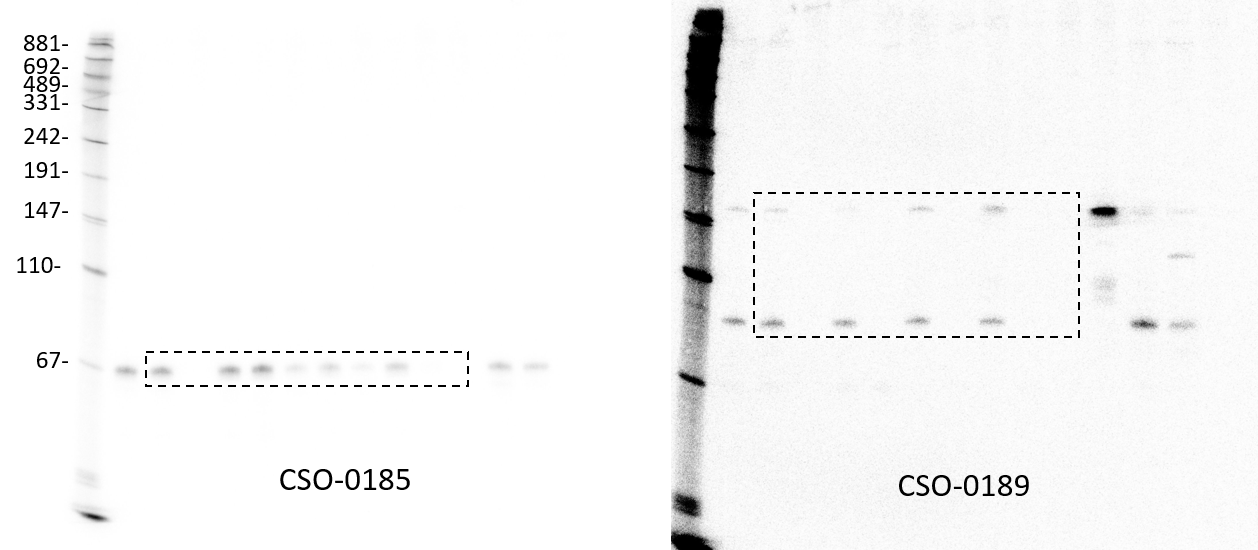


Northern blot quantification raw values

|  |  | ***ptmG* mRNA** | **CJnc190 (mature)** |
| --- | --- | --- | --- |
|  |  | **CSO-1666** | **CSO-0185** |
|  |  | **Intensity-Bkg [%]** | |
| **PtmG-3xFLAG** |  | 5.827133342 | 14.50341421 |
| **PtmG-3xFLAG** | **Δ180/190** | 18.47692929 | 16.57116621 |
| **PtmG-3xFLAG** | **C-180/190** | 3.048929268 | 17.71117892 |
| **PtmG-3xFLAG** | **C-190 only** | 3.671616917 | 4.298162038 |
| **PtmG-3xFLAG** | **C-180 + 190-P1** | 13.07626863 | 6.692226924 |
| **PtmG-3xFLAG** | **C-190-P1** | 5.224445005 | 2.316732738 |
| **PtmG-3xFLAG** | **C-180 + 190-P2** | 16.52610918 | 7.813159145 |
| **PtmG-3xFLAG** | **C-190-P2** | 7.855832392 | 0.332387762 |
| **PtmG-3xFLAG** | **C-3xmut** | 20.78599705 | 0.041217932 |
