# Supplementary material for: RNase III-mediated processing of a trans-acting bacterial sRNA and its cis-encoded antagonist
Source: eLife. 2021 Nov 29;10:e69064. doi: 10.7554/eLife.69064 (PMC8687705; doi:10.7554/eLife.69064)
Supplement: Figure 7—figure supplement 2—source data 1. [file elife-69064-fig7-figsupp2-data1.zip › Source data - Figure 7 - figure supplement 2/Source data - Figure 7 - figure supplement 2.docx]

**Source data for Figure 7 – figure supplement 2**

**
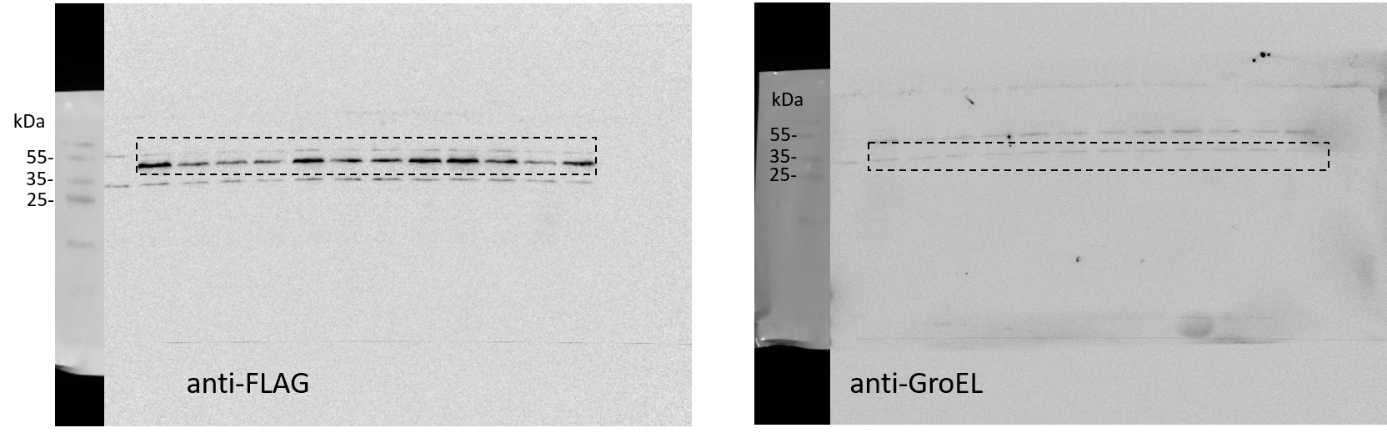
**

Western blot quantification raw values

|  |  | **PtmG-3xFLAG** |
| --- | --- | --- |
|  |  | **anti-FLAG** |
|  |  | **Intensity-Bkg [%]** |
|  |  | **R1** |
| **Δ180/190** | **0.25** | 14.42764256 |
| **C-180/190** |  | 5.220215686 |
| **C-190 only** |  | 4.449499996 |
| **OE-180(Proc)** |  | 4.096550798 |
| **Δ180/190** | **0.5** | 12.21462492 |
| **C-180/190** |  | 6.736782484 |
| **C-190 only** |  | 7.110139715 |
| **OE-180(Proc)** |  | 12.30876951 |
| **Δ180/190** | **0.8** | 13.97149383 |
| **C-180/190** |  | 8.15398757 |
| **C-190 only** |  | 3.115916866 |
| **OE-180(Proc)** |  | 8.19437607 |

NB145

**
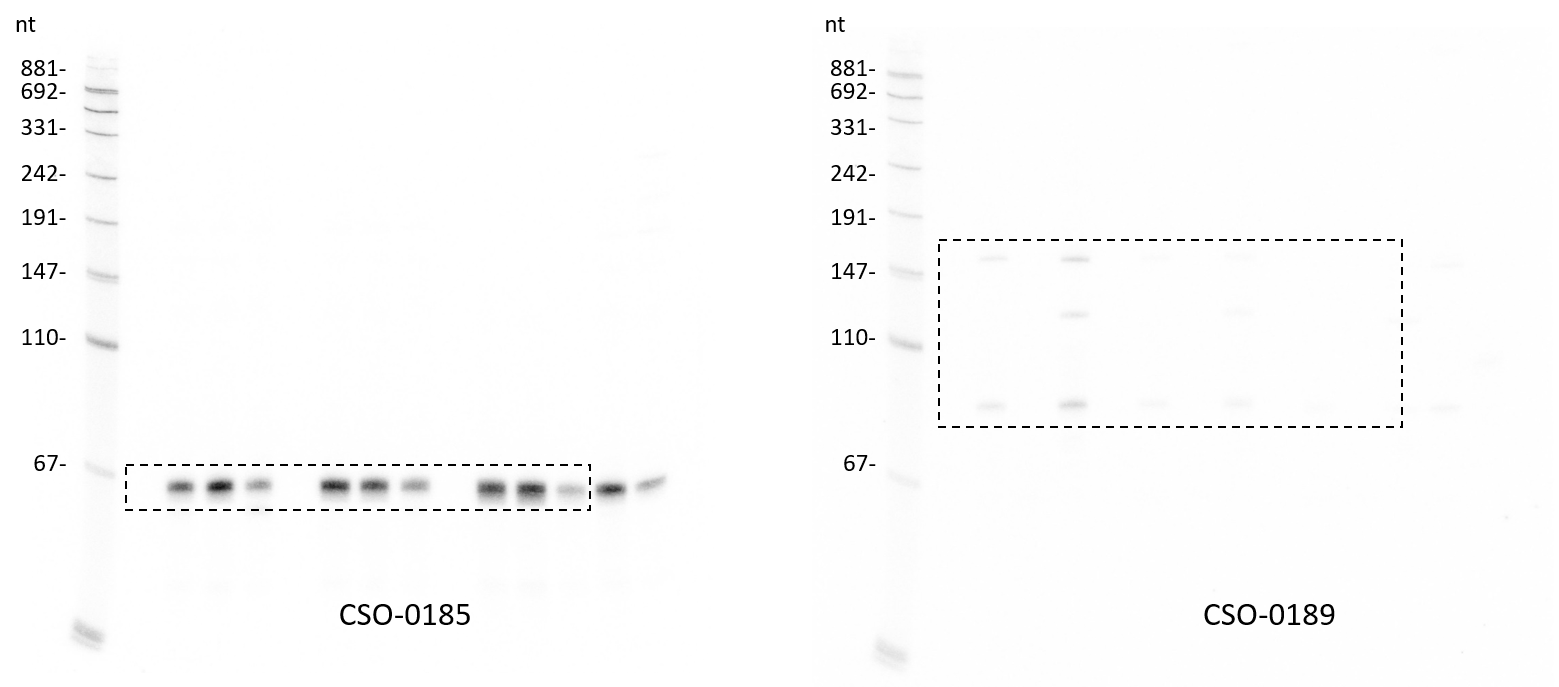
**

**
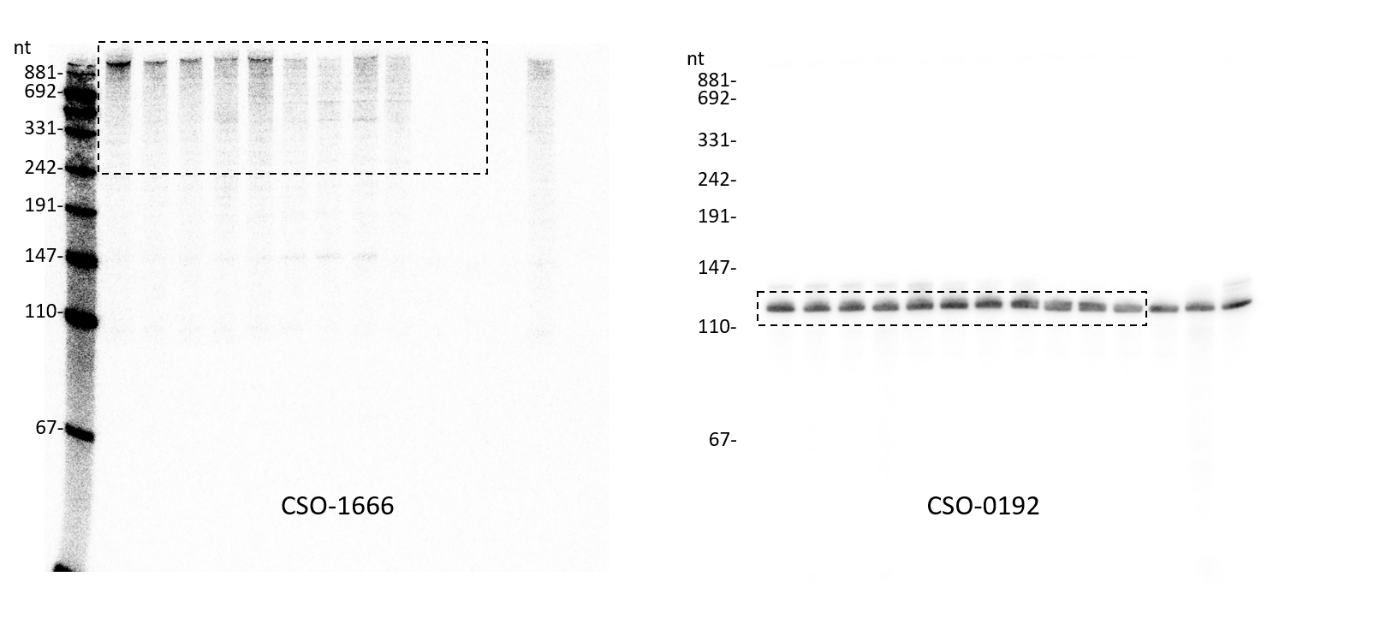
**

Northern blot quantification raw values

|  |  | ***ptmG* mRNA** |
| --- | --- | --- |
|  |  | **CSO-1666** |
|  |  | **Intensity-Bkg [%]** |
| **Δ180/190** | **0.25** | 20.3074 |
| **C-180/190** |  | 9.8624 |
| **C-190 only** |  | 9.3079 |
| **OE-180(Proc)** |  | 10.6566 |
| **Δ180/190** | **0.5** | 15.2840 |
| **C-180/190** |  | 5.9545 |
| **C-190 only** |  | 4.9419 |
| **OE-180(Proc)** |  | 9.4394 |
| **Δ180/190** | **0.8** | 6.1060 |
| **C-180/190** |  | 0.1243 |
| **C-190 only** |  | 0.1435 |
| **OE-180(Proc)** |  | 0.2731 |
