# Supplementary figures and images for: RNase III-mediated processing of a trans-acting bacterial sRNA and its cis-encoded antagonist (part 2 of 2)
Source: eLife. 2021 Nov 29;10:e69064. doi: 10.7554/eLife.69064 (PMC8687705; doi:10.7554/eLife.69064)

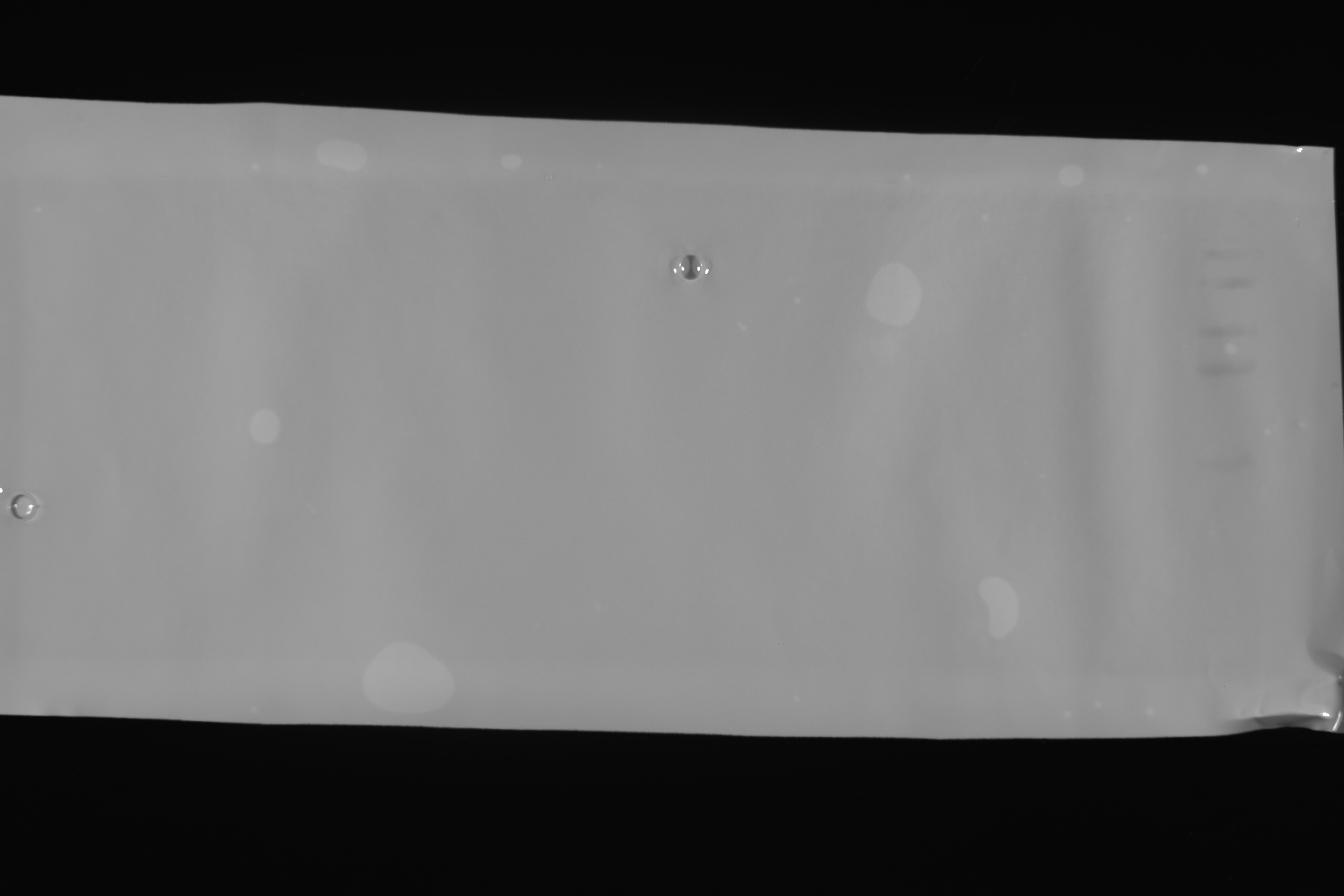

Supplement: Figure 7—figure supplement 2—source data 1. [file elife-69064-fig7-figsupp2-data1.zip › Source data - Figure 7 - figure supplement 2/Fig 7 - supp 2 - 20181015_1410_R1_GroEL_ladder.tif]

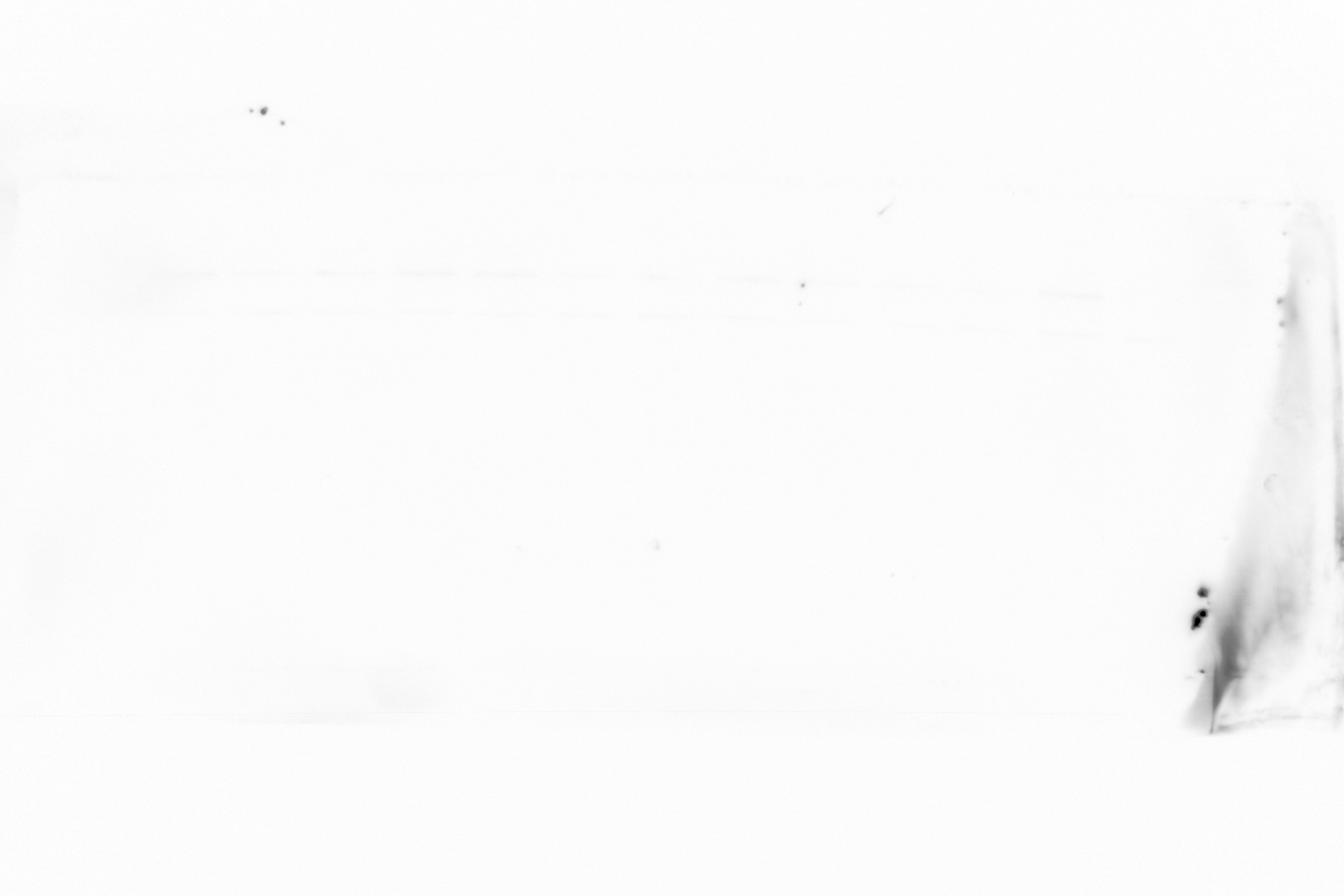

Supplement: Figure 7—figure supplement 2—source data 1. [file elife-69064-fig7-figsupp2-data1.zip › Source data - Figure 7 - figure supplement 2/Fig 7 - supp 2 - 20181015_1420_R1_GroEL_29.tif]

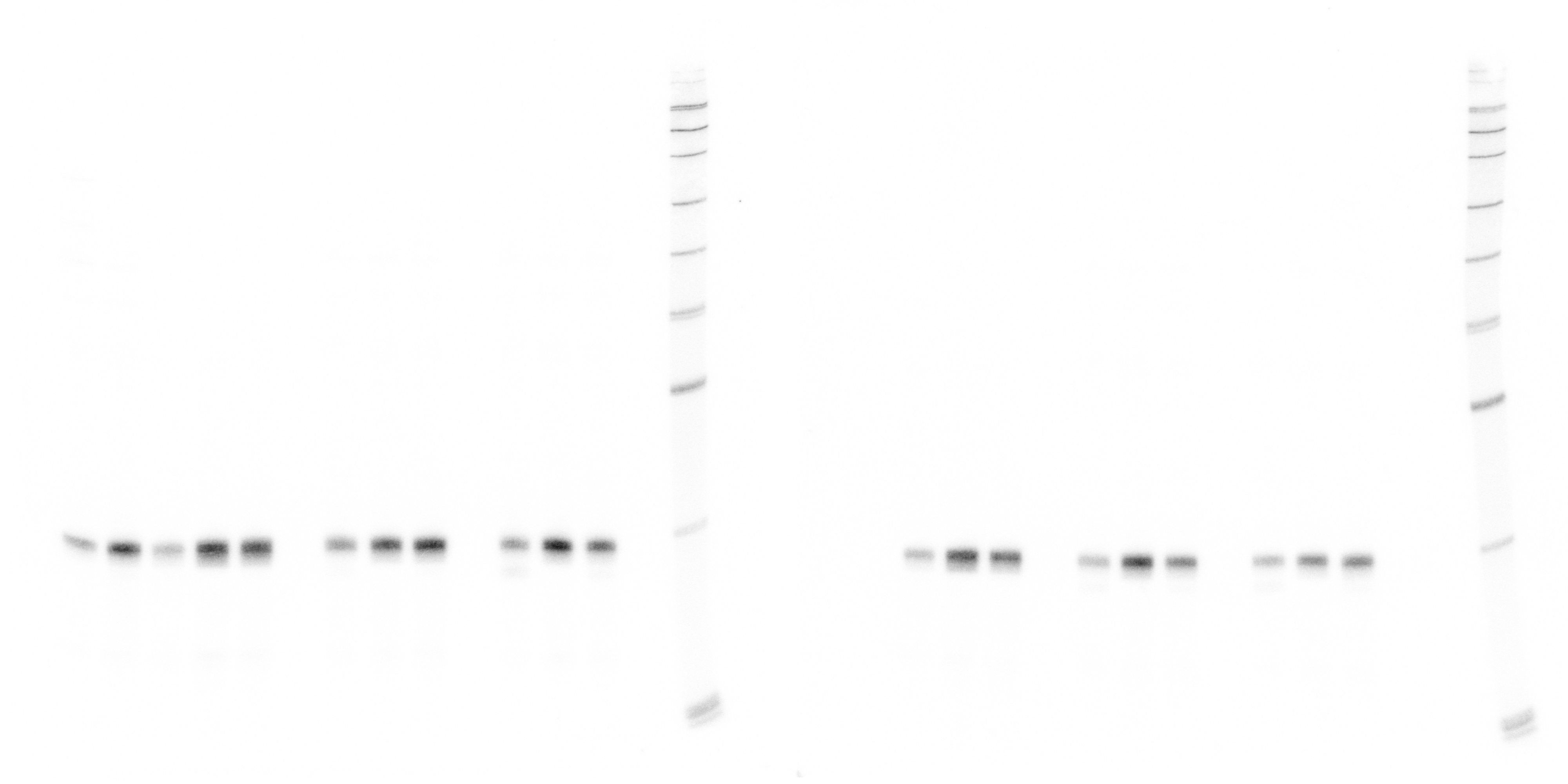

Supplement: Figure 7—figure supplement 2—source data 1. [file elife-69064-fig7-figsupp2-data1.zip › Source data - Figure 7 - figure supplement 2/Fig 7 - supp 2 - 20181018_NB145_146_CSO-0185_4d-[Phosphor].tif]

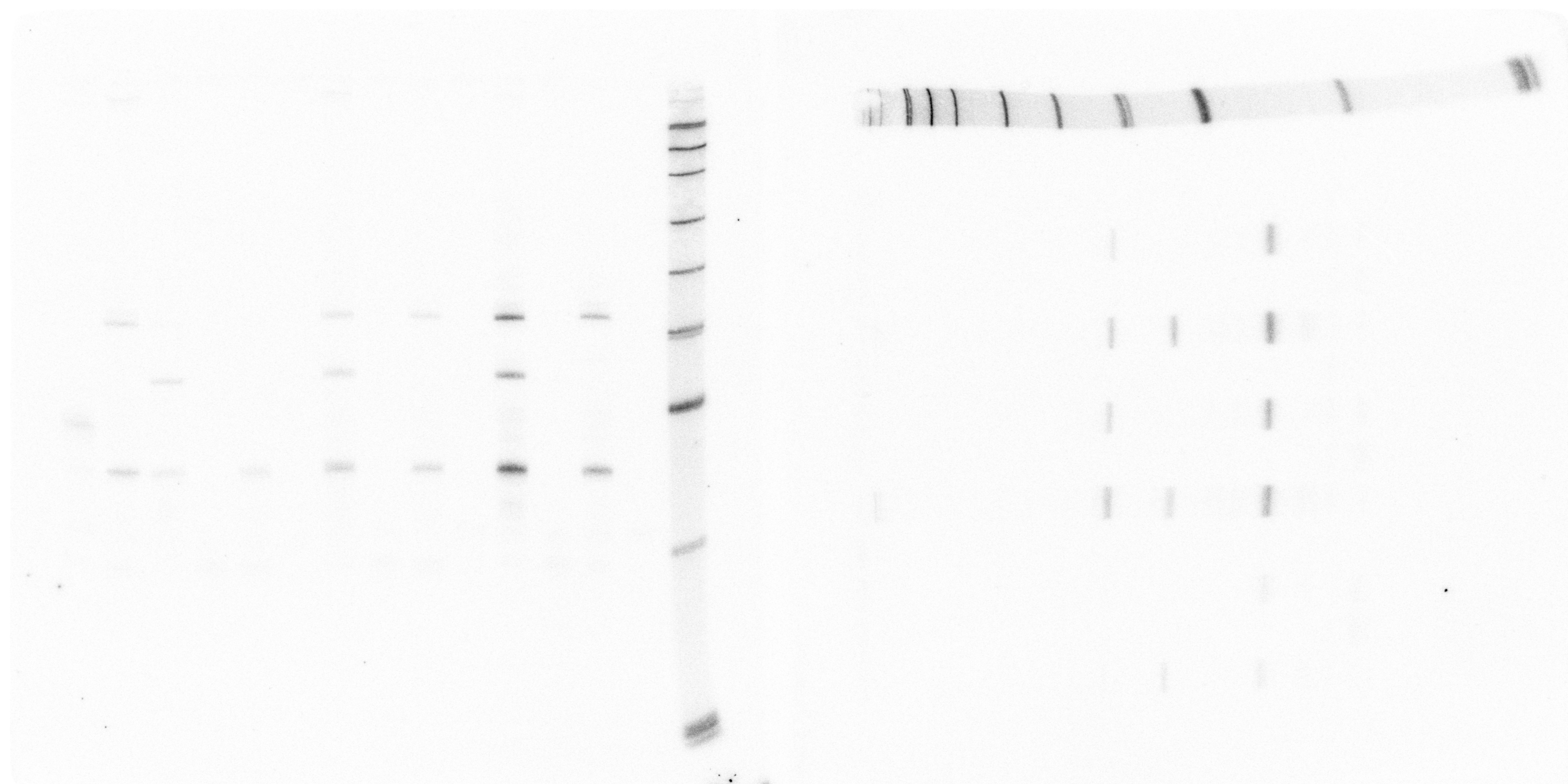

Supplement: Figure 7—figure supplement 2—source data 1. [file elife-69064-fig7-figsupp2-data1.zip › Source data - Figure 7 - figure supplement 2/Fig 7 - supp 2 - 2018_11_07_NB145_146_CSO-0189_7d-[Phosphor].tif]

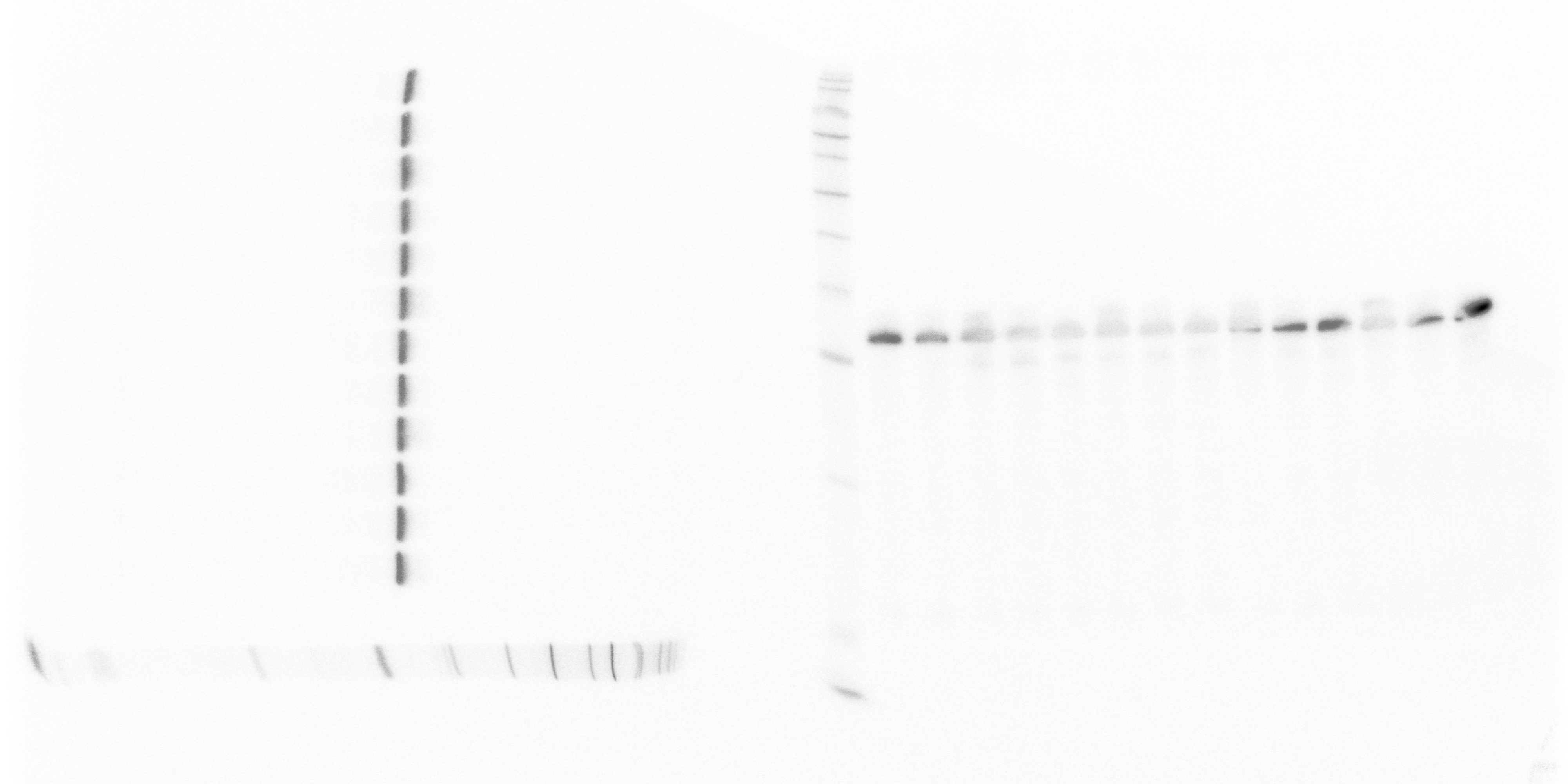

Supplement: Figure 7—figure supplement 3—source data 1. [file elife-69064-fig7-figsupp3-data1.zip › Source data - Figure 7 - figure supplement 3 - Source Data 1/Fig 7 - supp 3A - 18.8.2021_NB217_218_CSO-0192_1d-[Phosphor].tif]

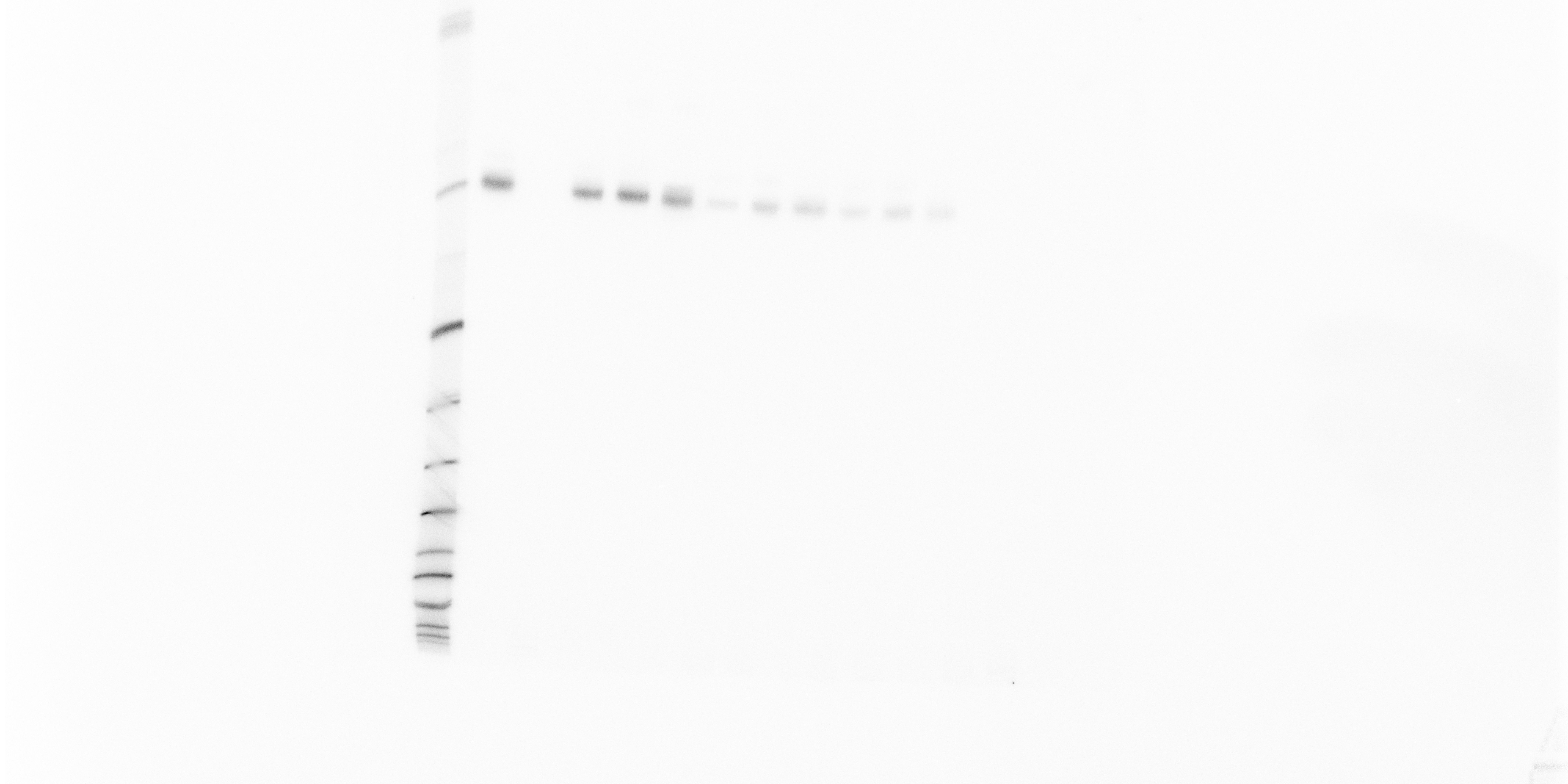

Supplement: Figure 7—figure supplement 3—source data 1. [file elife-69064-fig7-figsupp3-data1.zip › Source data - Figure 7 - figure supplement 3 - Source Data 1/Fig 7 - supp 3A - 2021.8.27_NB220_CSO-0185_10d-[Phosphor].jpg]

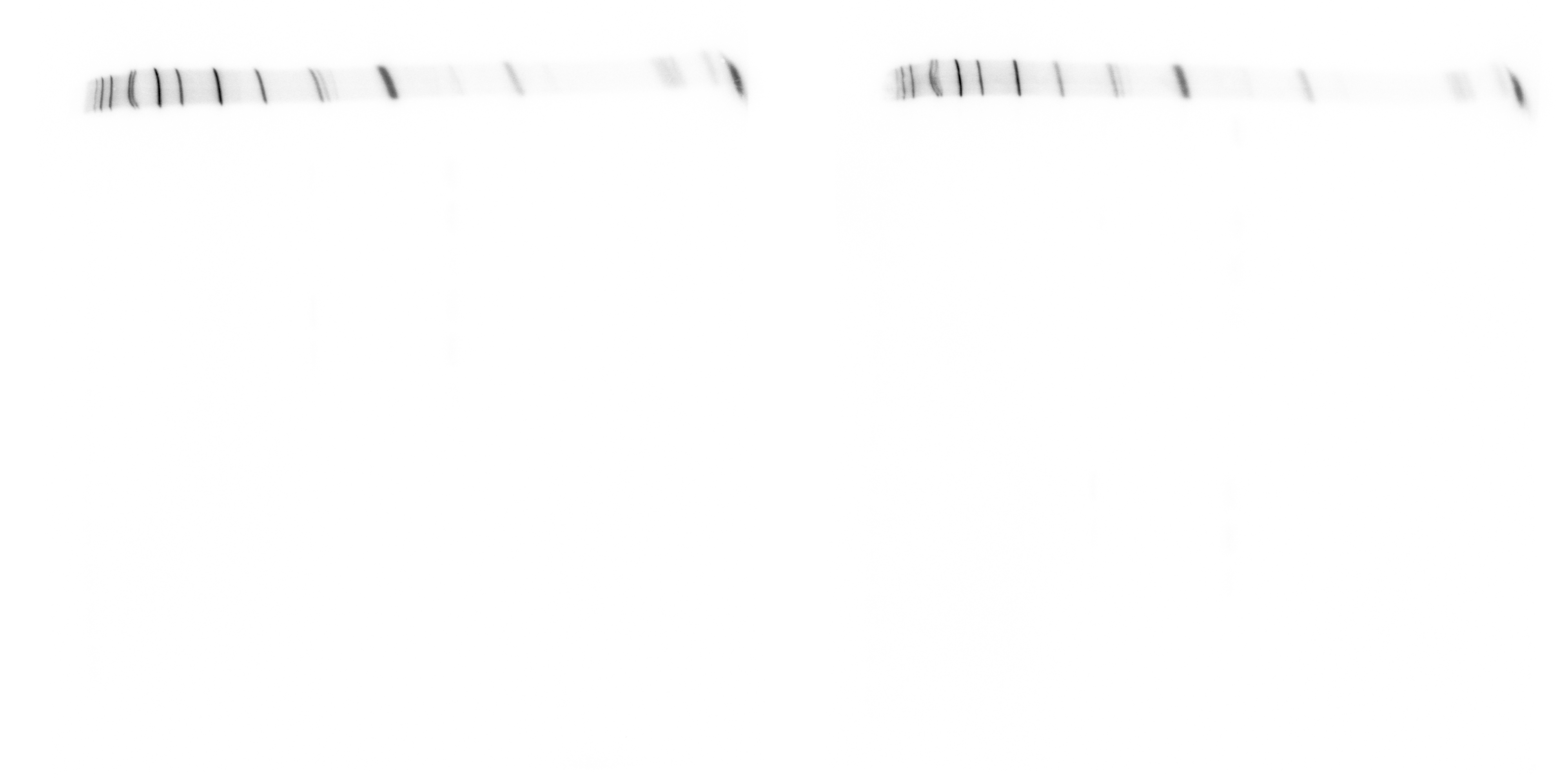

Supplement: Figure 7—figure supplement 3—source data 1. [file elife-69064-fig7-figsupp3-data1.zip › Source data - Figure 7 - figure supplement 3 - Source Data 1/Fig 7 - supp 3A - 24.7.2021_NB217_218_CSO-0189_3d-[Phosphor].tif]

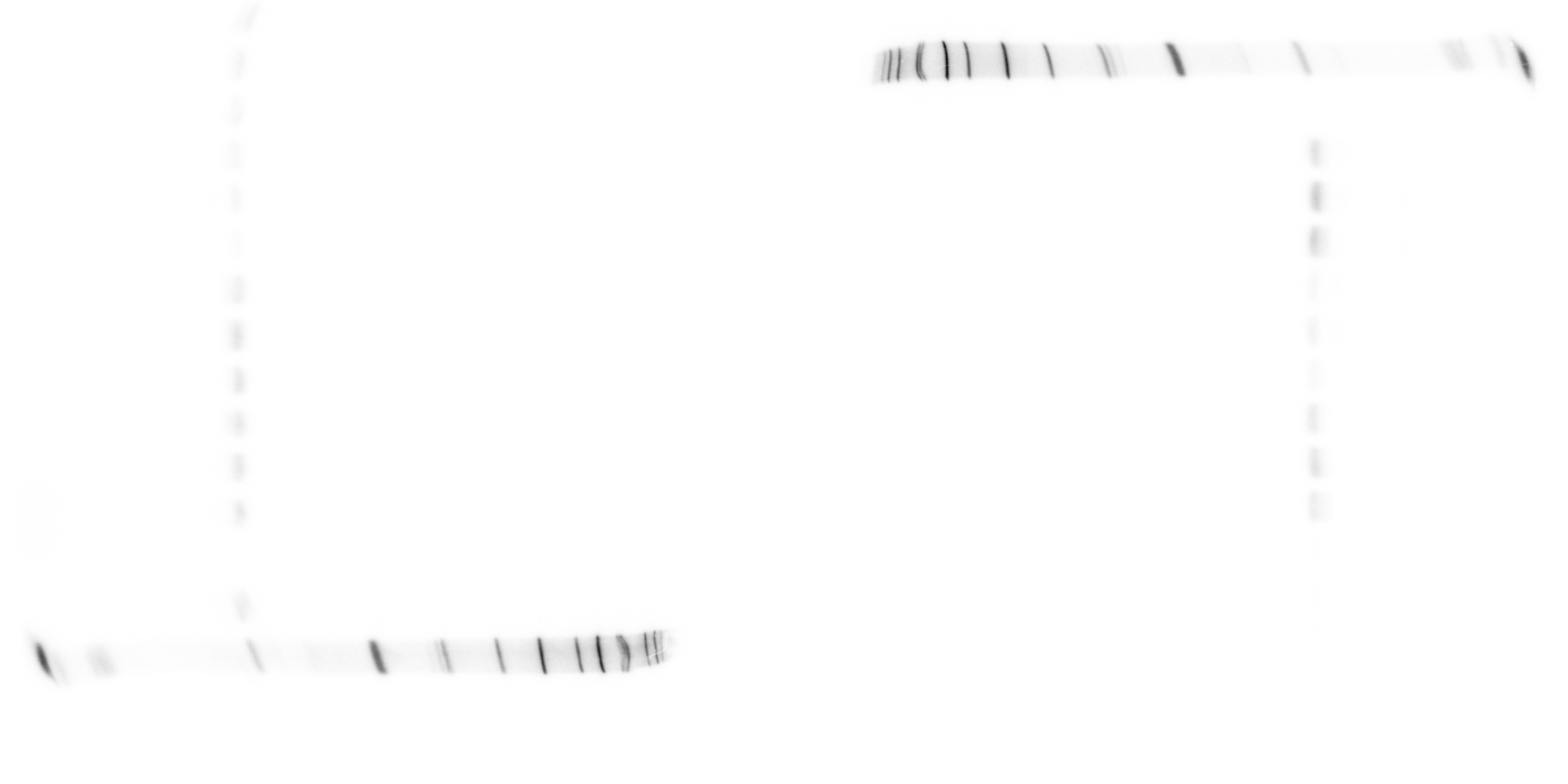

Supplement: Figure 7—figure supplement 3—source data 1. [file elife-69064-fig7-figsupp3-data1.zip › Source data - Figure 7 - figure supplement 3 - Source Data 1/Fig 7 - supp 3A - 28.7.2021_NB217_218_CSO-0185_3d-[Phosphor].tif]

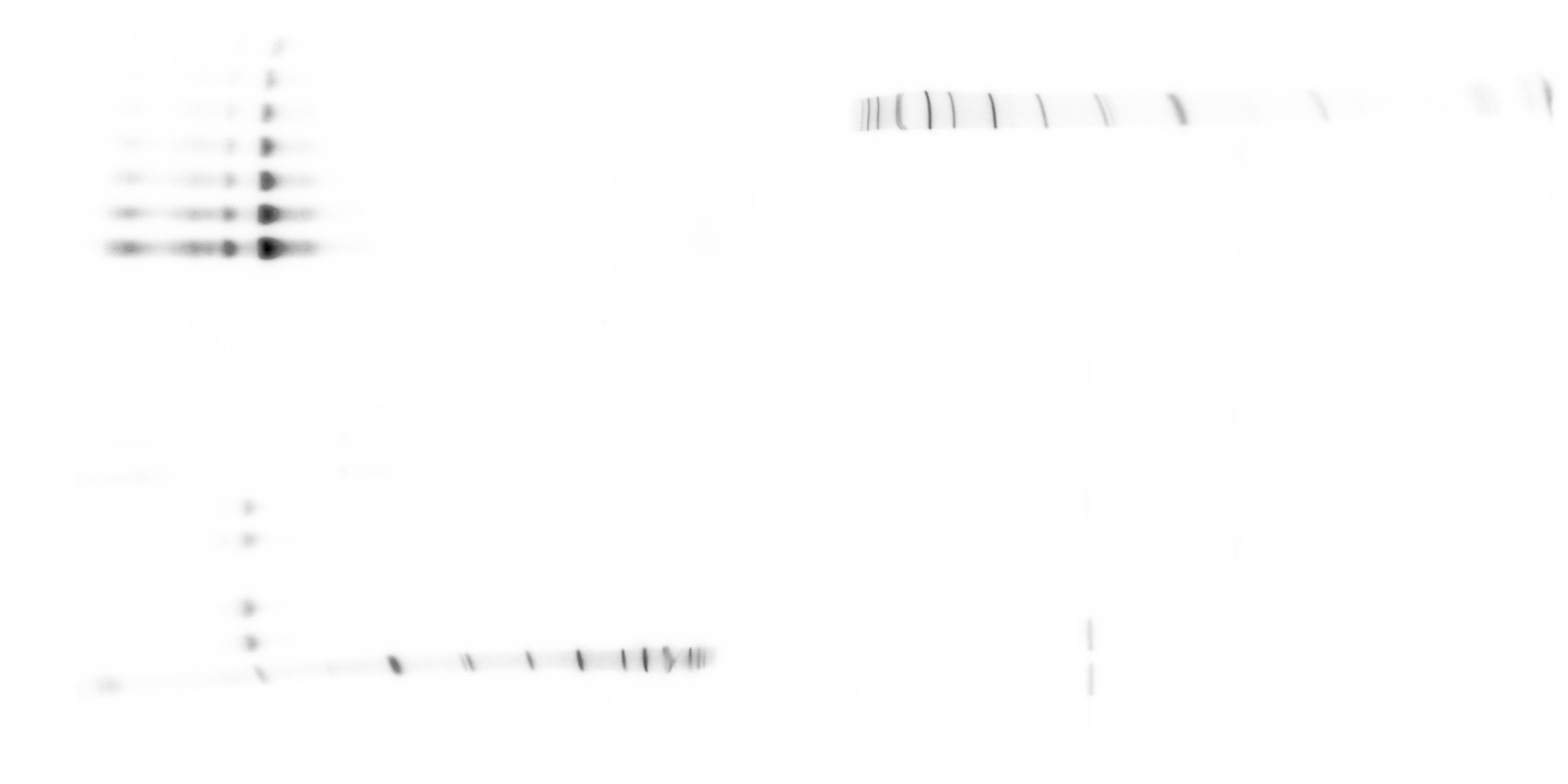

Supplement: Figure 7—figure supplement 3—source data 1. [file elife-69064-fig7-figsupp3-data1.zip › Source data - Figure 7 - figure supplement 3 - Source Data 1/Fig 7 - supp 3A - 31.7.2021_NB219_220_CSO-0189 _0185_4d-[Phosphor].tif]

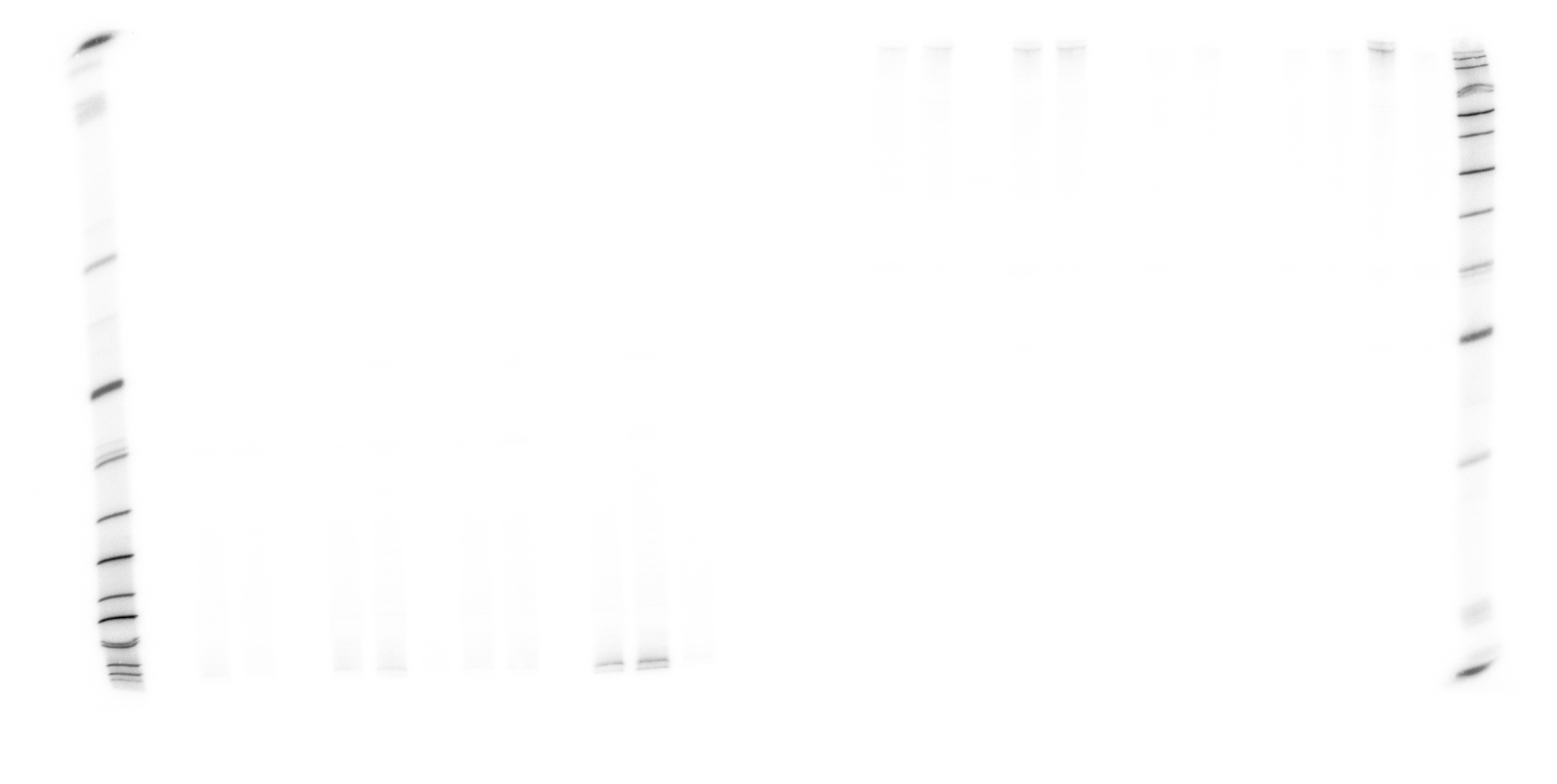

Supplement: Figure 7—figure supplement 3—source data 1. [file elife-69064-fig7-figsupp3-data1.zip › Source data - Figure 7 - figure supplement 3 - Source Data 1/Fig 7 - supp 3A - 5.8.2021_NB217_218_CSO-1666_7d-[Phosphor].tif]

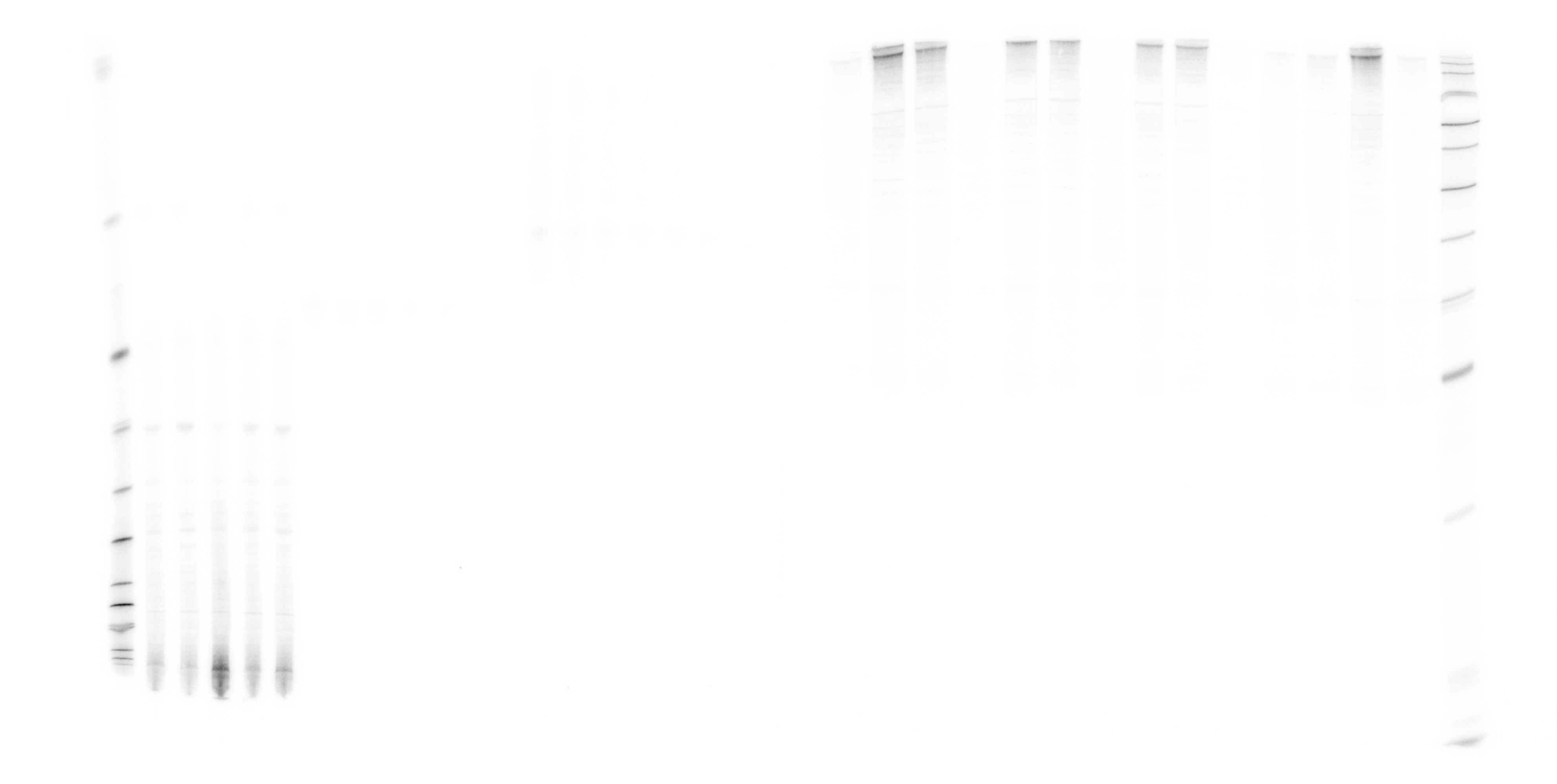

Supplement: Figure 7—figure supplement 3—source data 1. [file elife-69064-fig7-figsupp3-data1.zip › Source data - Figure 7 - figure supplement 3 - Source Data 1/Fig 7 - supp 3A - 5.8.2021_NB219_220_CSO-1666_7d-[Phosphor].tif]

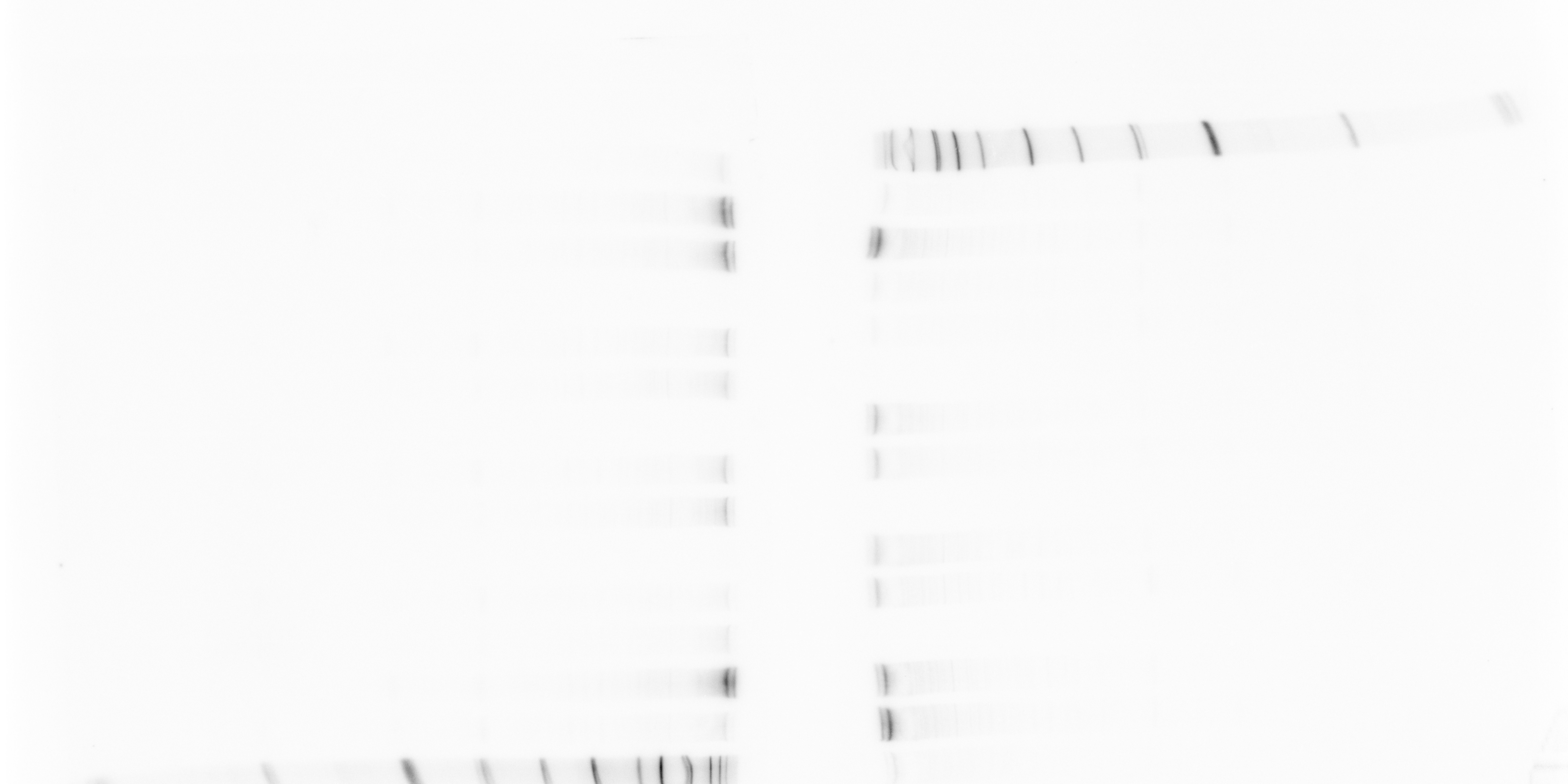

Supplement: Figure 7—figure supplement 3—source data 1. [file elife-69064-fig7-figsupp3-data1.zip › Source data - Figure 7 - figure supplement 3 - Source Data 1/Fig 7 - supp 3B - 20210826_NB215_216_CSO-1666_10d-[Phosphor].jpg]

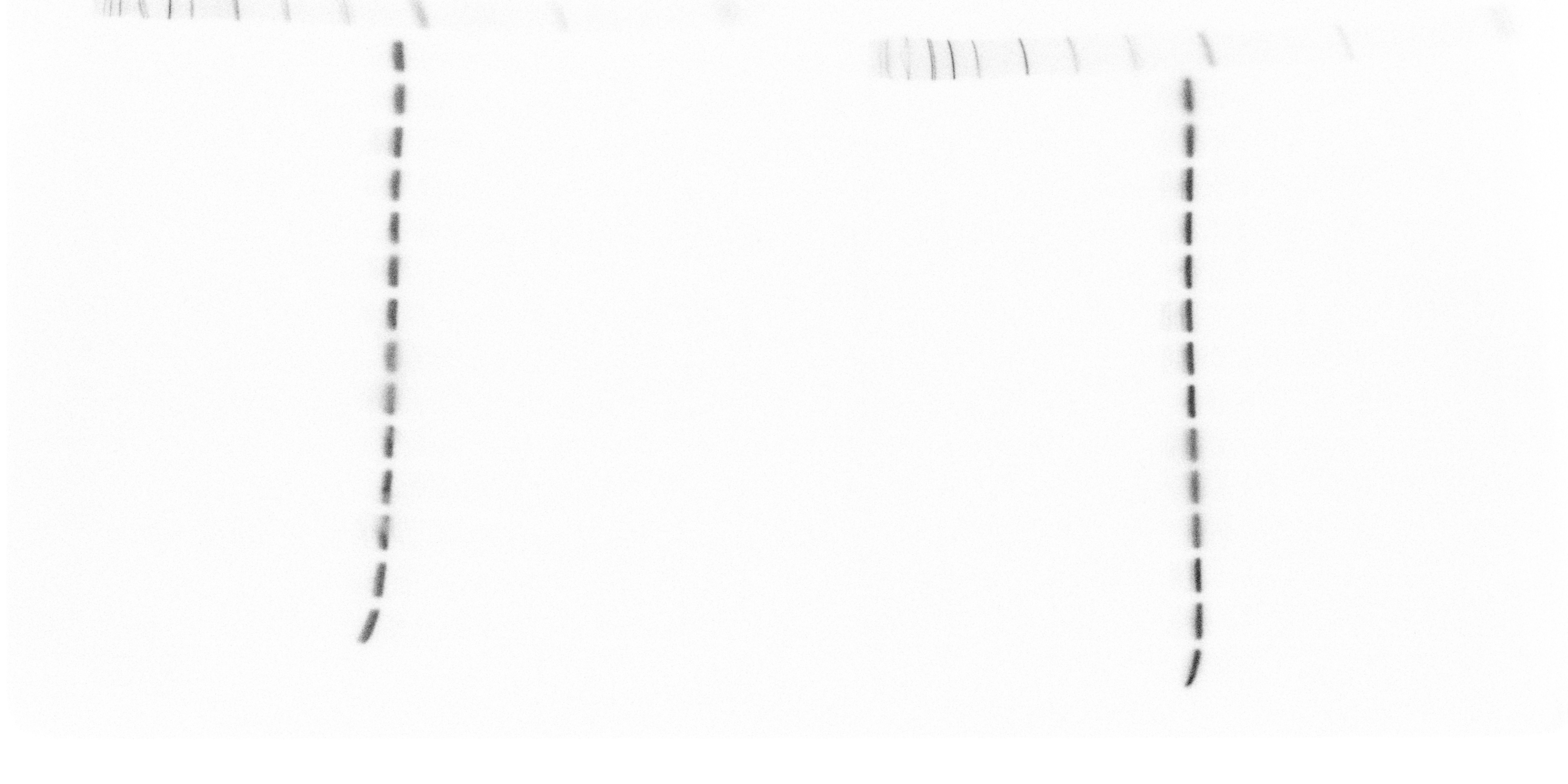

Supplement: Figure 7—figure supplement 3—source data 2. [file elife-69064-fig7-figsupp3-data2.zip › Source data - Figure 7 - figure supplement 3 - Source Data 2/Fig 7 - supp 3B - 2021.8.29_NB215_216_CSO-0192_1d-[Phosphor].tif]

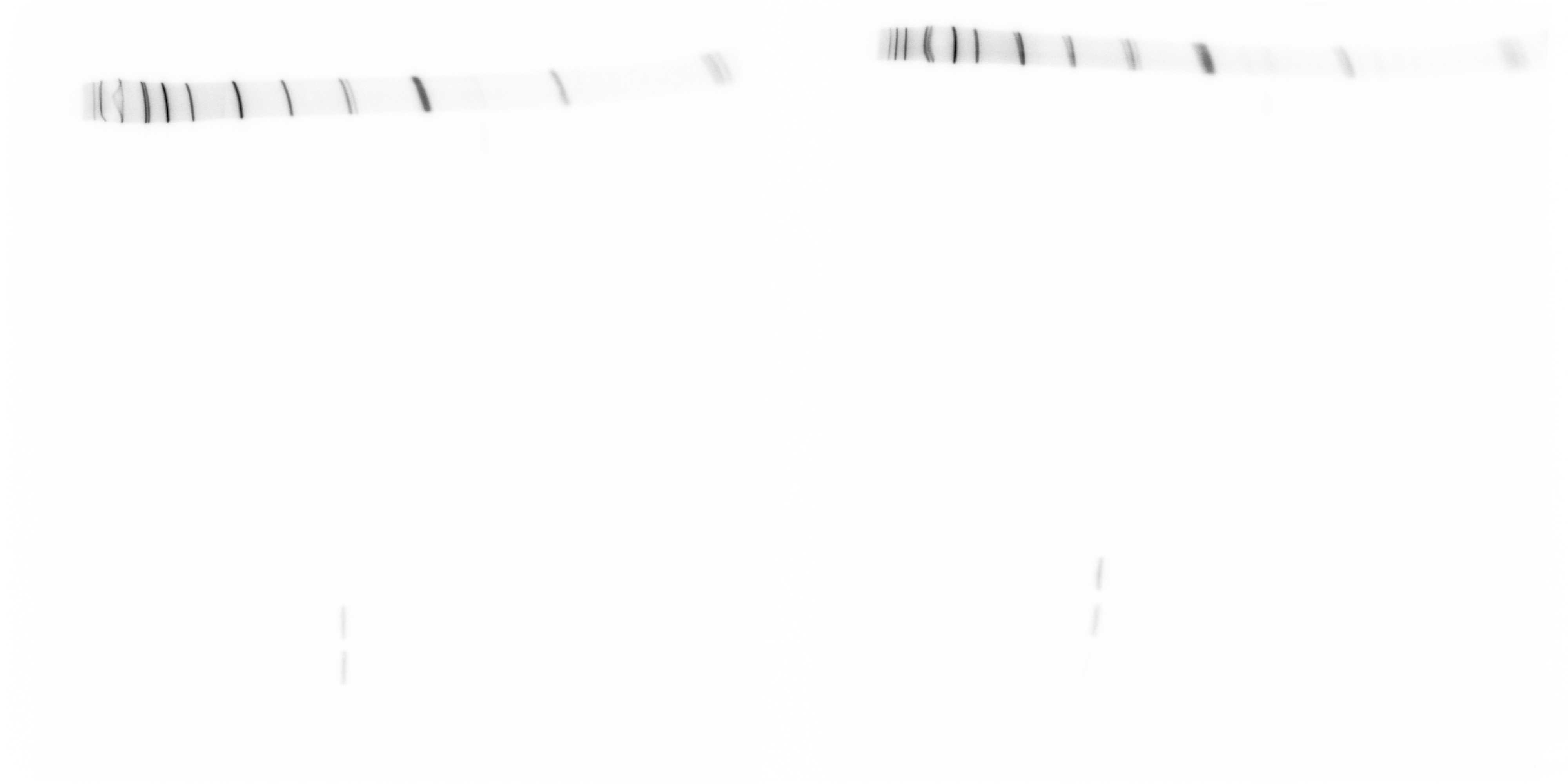

Supplement: Figure 7—figure supplement 3—source data 2. [file elife-69064-fig7-figsupp3-data2.zip › Source data - Figure 7 - figure supplement 3 - Source Data 2/Fig 7 - supp 3B - 23.7.2021_NB215_216_CSO-0189_3d-[Phosphor].tif]

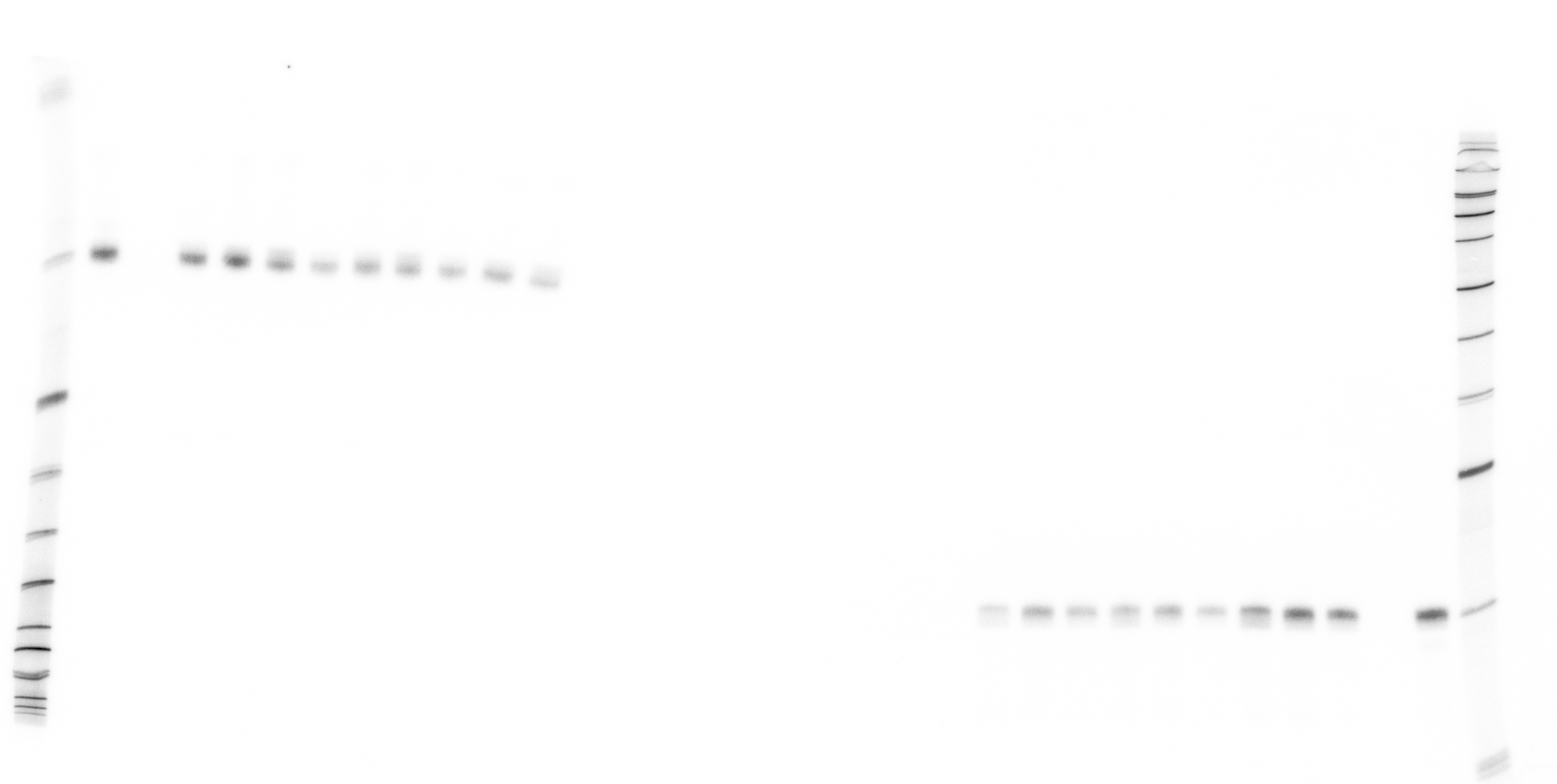

Supplement: Figure 7—figure supplement 3—source data 2. [file elife-69064-fig7-figsupp3-data2.zip › Source data - Figure 7 - figure supplement 3 - Source Data 2/Fig 7 - supp 3B - 5.8.2021_NB215_216_CSO-0185_7d-[Phosphor].tif]

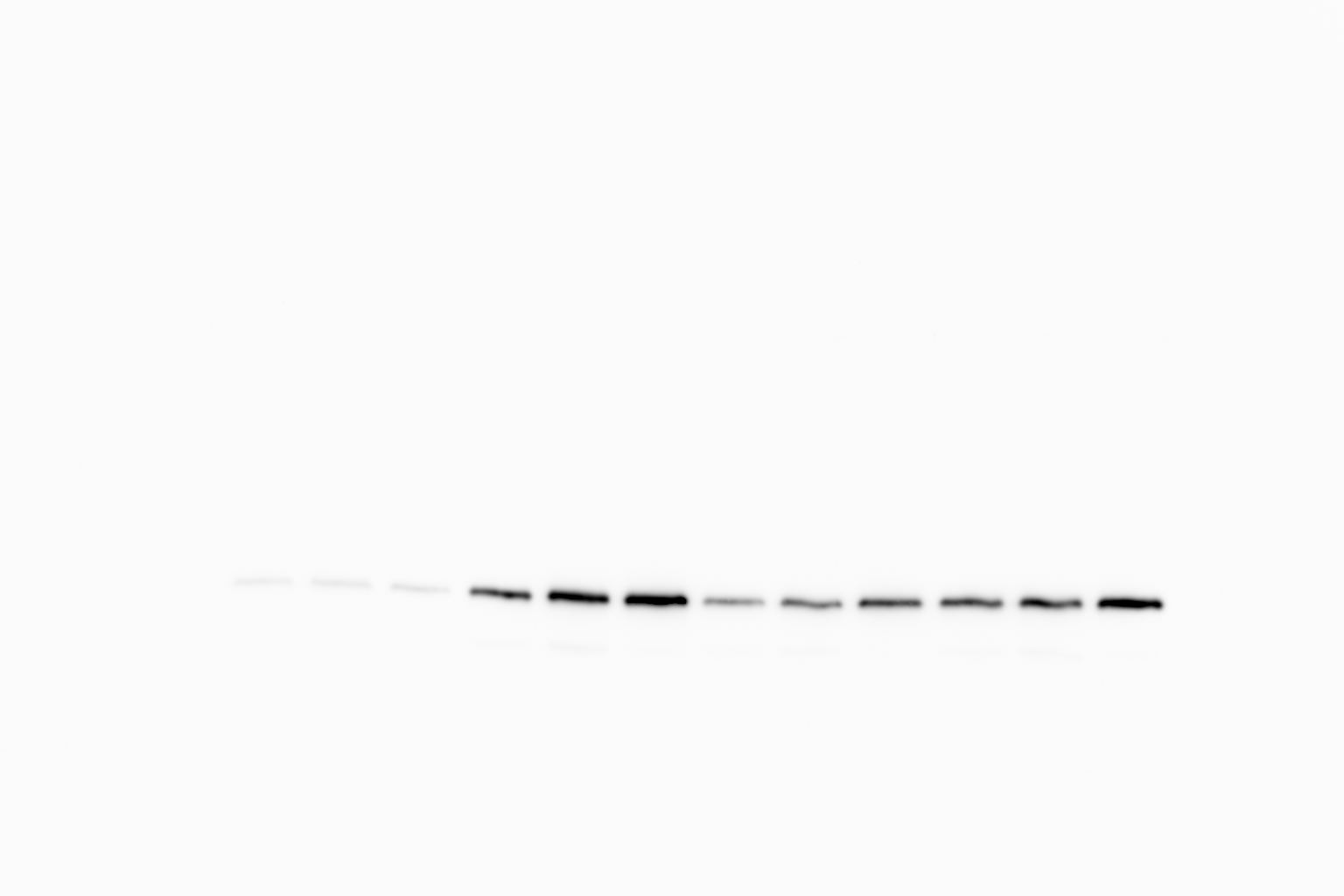

Supplement: Figure 7—figure supplement 3—source data 2. [file elife-69064-fig7-figsupp3-data2.zip › Source data - Figure 7 - figure supplement 3 - Source Data 2/Fig 7 - supp 3C - 20210723_dif.190strains_anti-GFP_standard_10.tif]

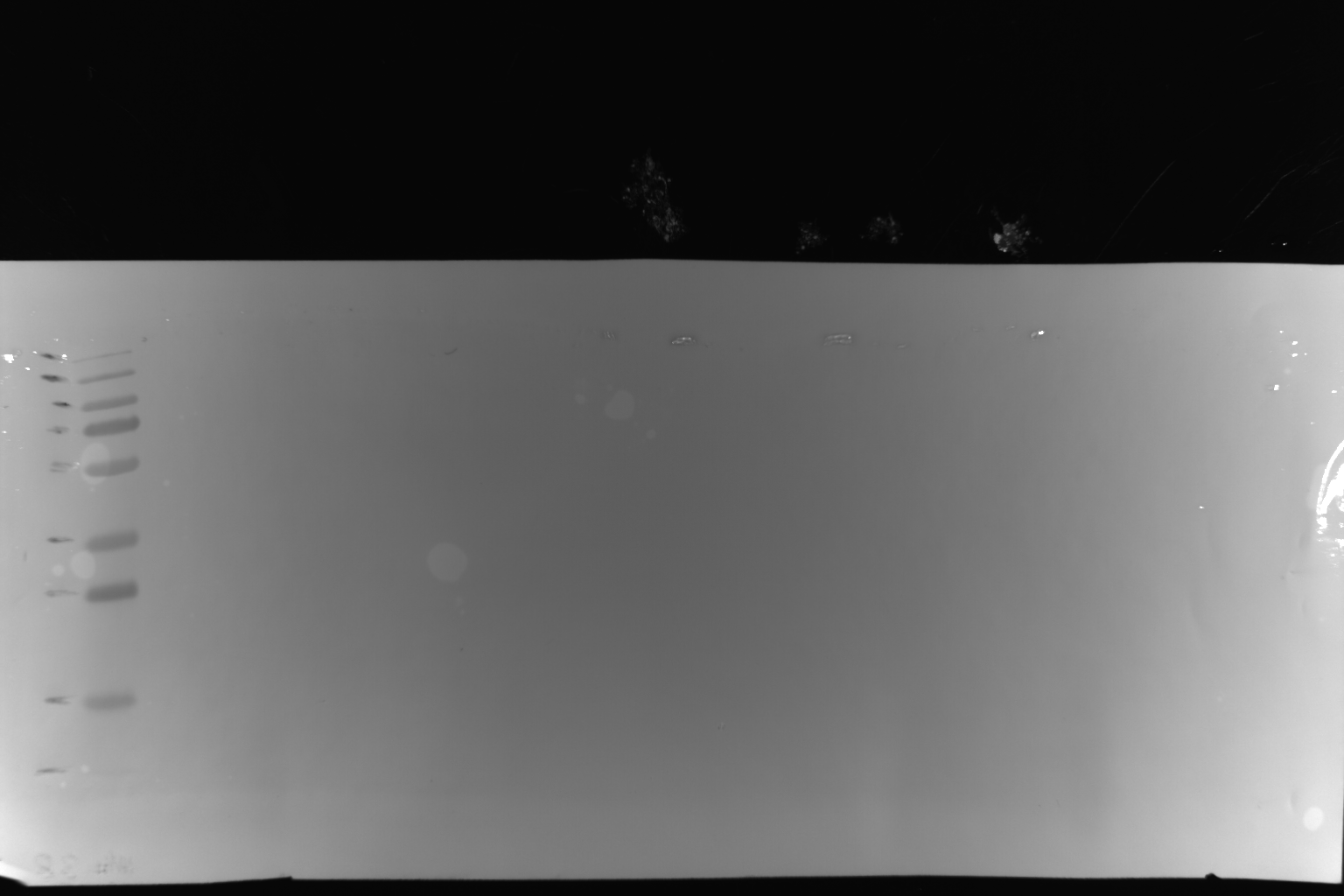

Supplement: Figure 7—figure supplement 3—source data 2. [file elife-69064-fig7-figsupp3-data2.zip › Source data - Figure 7 - figure supplement 3 - Source Data 2/Fig 7 - supp 3C - 20210723_dif.190strains_anti-GFP_standard_marker.tif]

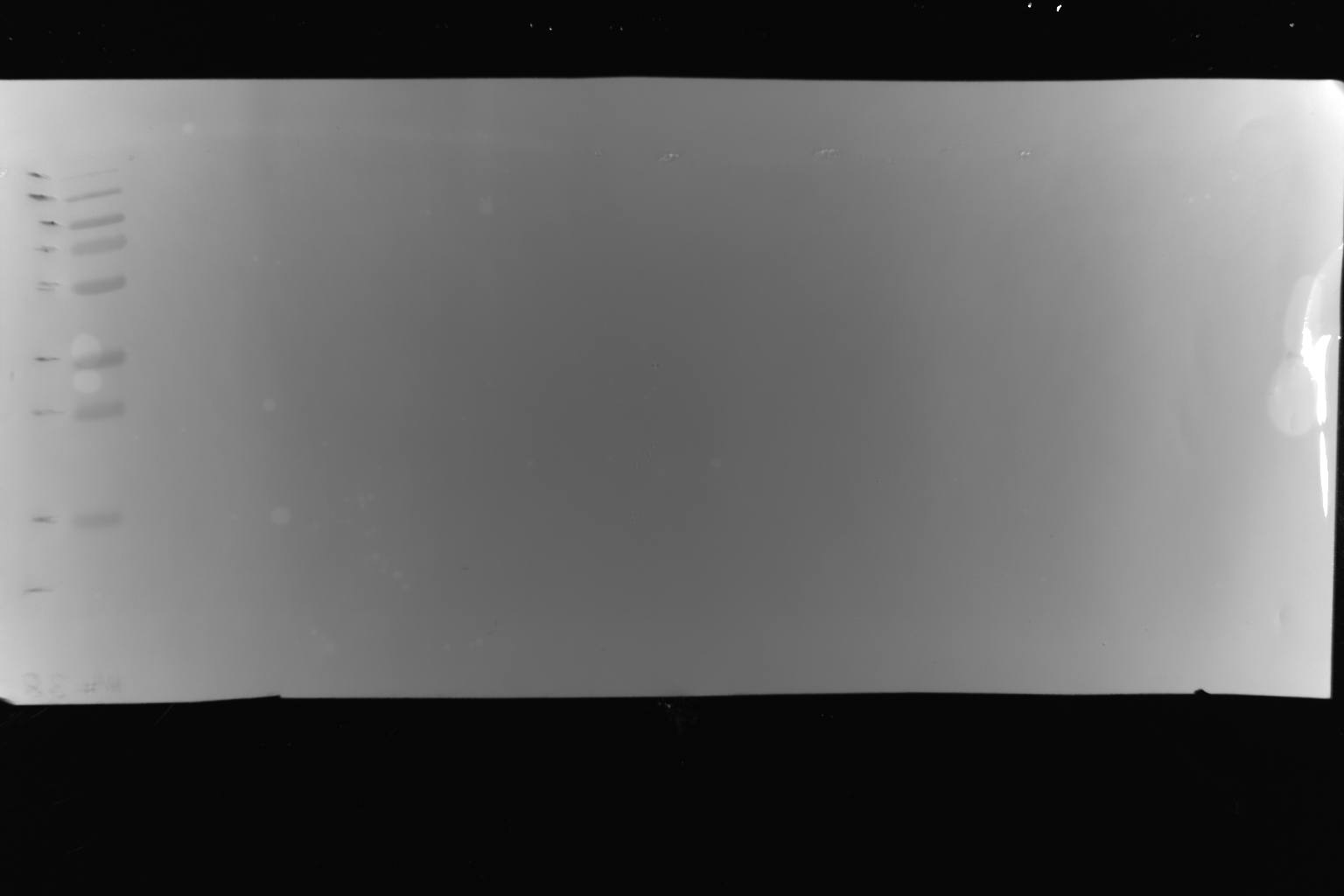

Supplement: Figure 7—figure supplement 3—source data 2. [file elife-69064-fig7-figsupp3-data2.zip › Source data - Figure 7 - figure supplement 3 - Source Data 2/Fig 7 - supp 3C - 20210724_1236 WB38 GroEL ladder.tif]

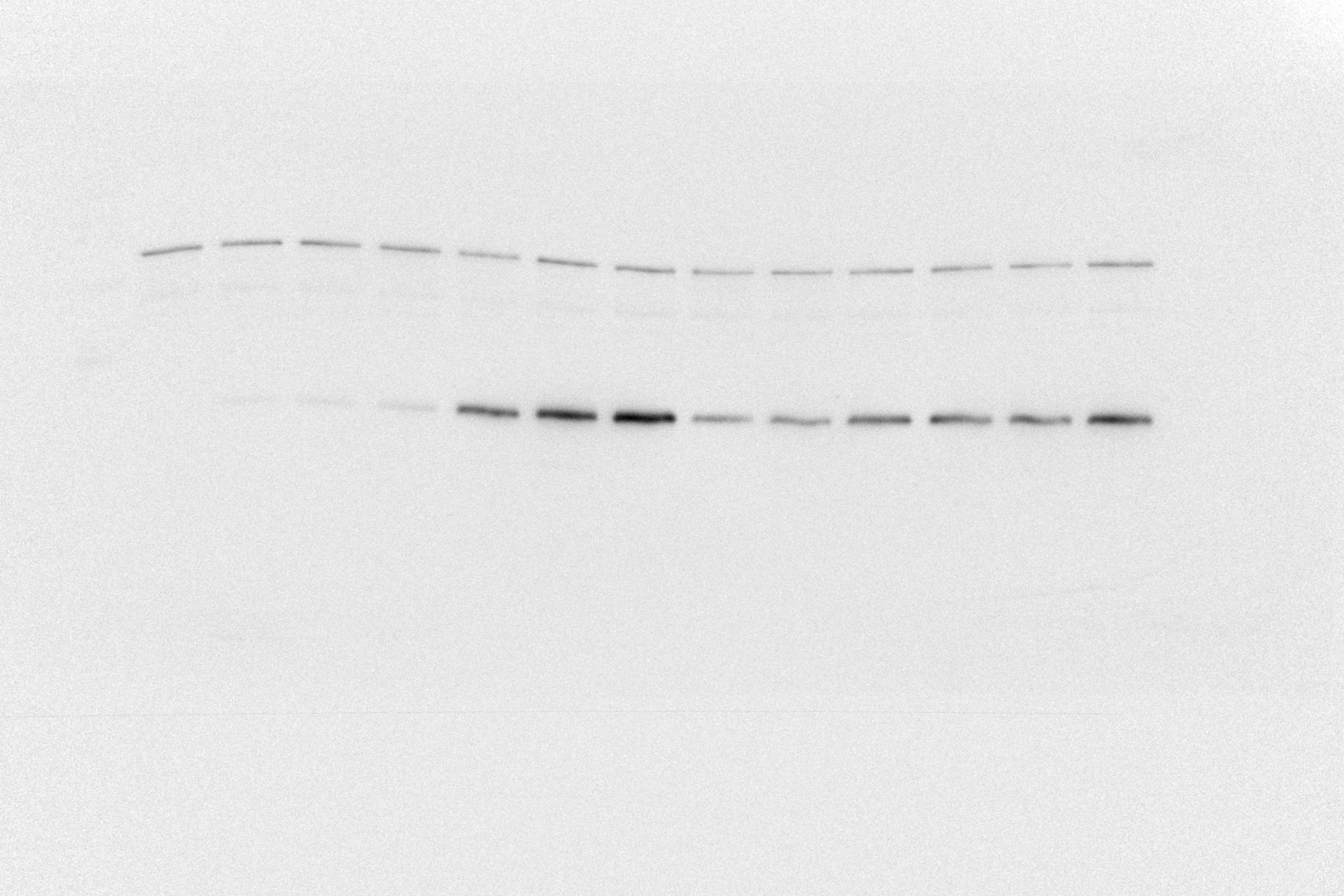

Supplement: Figure 7—figure supplement 3—source data 2. [file elife-69064-fig7-figsupp3-data2.zip › Source data - Figure 7 - figure supplement 3 - Source Data 2/Fig 7 - supp 3C - 20210724_1250 WB38 GroEL_44.tif]
