# Supplementary material for: RNase III-mediated processing of a trans-acting bacterial sRNA and its cis-encoded antagonist
Source: eLife. 2021 Nov 29;10:e69064. doi: 10.7554/eLife.69064 (PMC8687705; doi:10.7554/eLife.69064)
Supplement: Figure 7—figure supplement 3—source data 1. [file elife-69064-fig7-figsupp3-data1.zip › Source data - Figure 7 - figure supplement 3 - Source Data 1/Source data - Figure 7 - figure supplement 3.docx]

**Source data for Figure 7 – figure supplement 1**

**Panel A**

NB220


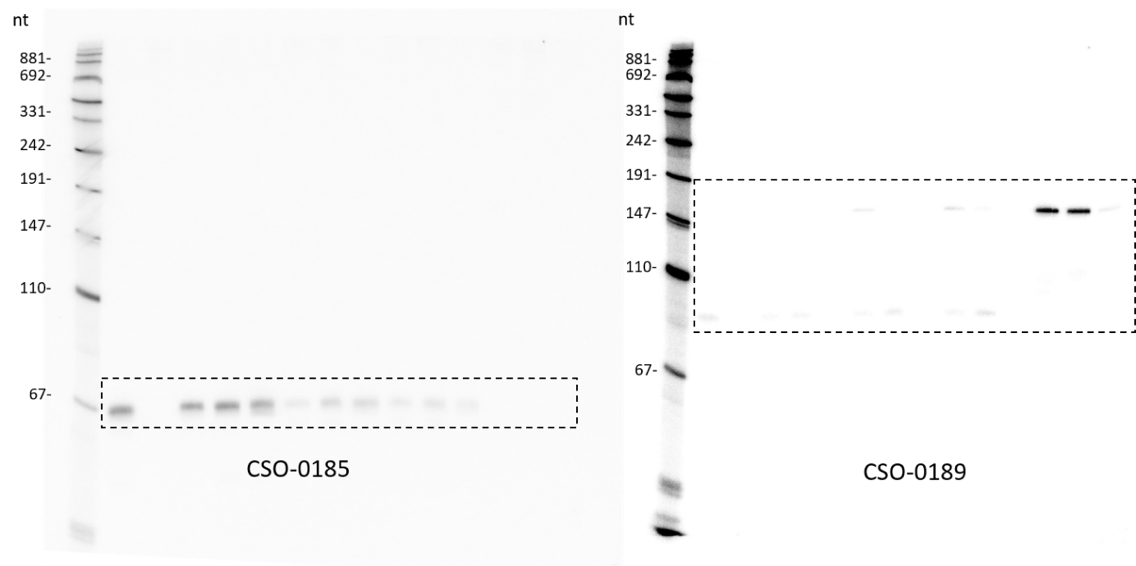


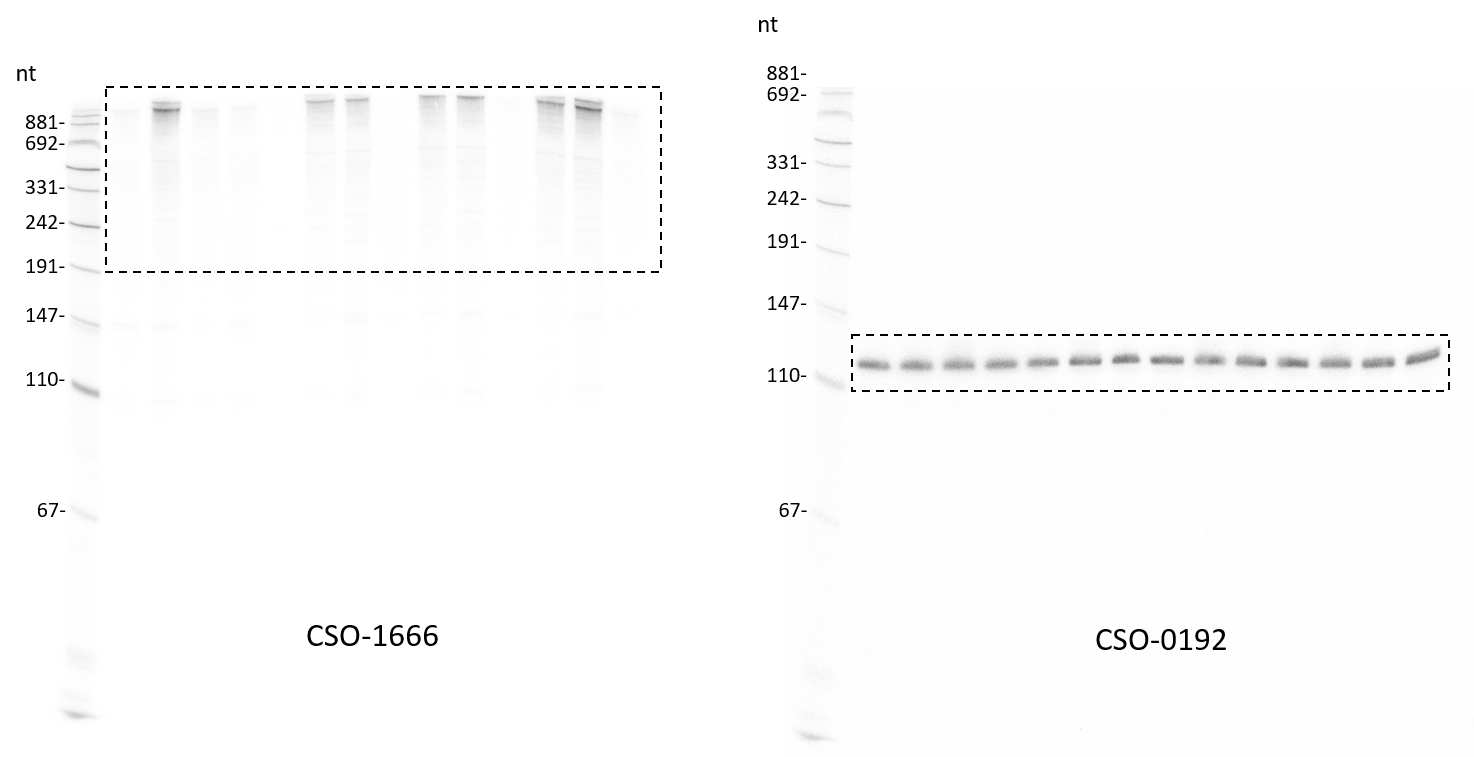


Northern blot quantification raw values

|  |  | **CJnc190 (mature)** | **pre-CJnc180** |
| --- | --- | --- | --- |
|  |  | **CSO-0185** | **CSO-0189** |
|  | OD600 | **Intensity-Bkg [%]** | |
| WT | 0.5 | - | - |
| Δ180/190 | 0.5 | - | - |
| C-190 only | 0.25 | 16.93344732 | - |
|  | 0.5 | 22.70037786 | - |
|  | 0.8 | 19.77724569 | - |
| C-190-P1 | 0.25 | 1.882366905 | - |
|  | 0.5 | 5.017710465 | - |
|  | 0.8 | 5.284687485 | - |
| C-190-P2 | 0.25 | 1.860766117 | - |
|  | 0.5 | 2.722615764 | - |
|  | 0.8 | 1.616611748 | - |
| C-180-P1 | 0.25 | - | 52.71520579 |
|  | 0.5 | - | 46.02655814 |
|  | 0.8 | - | 1.258236069 |

NB218


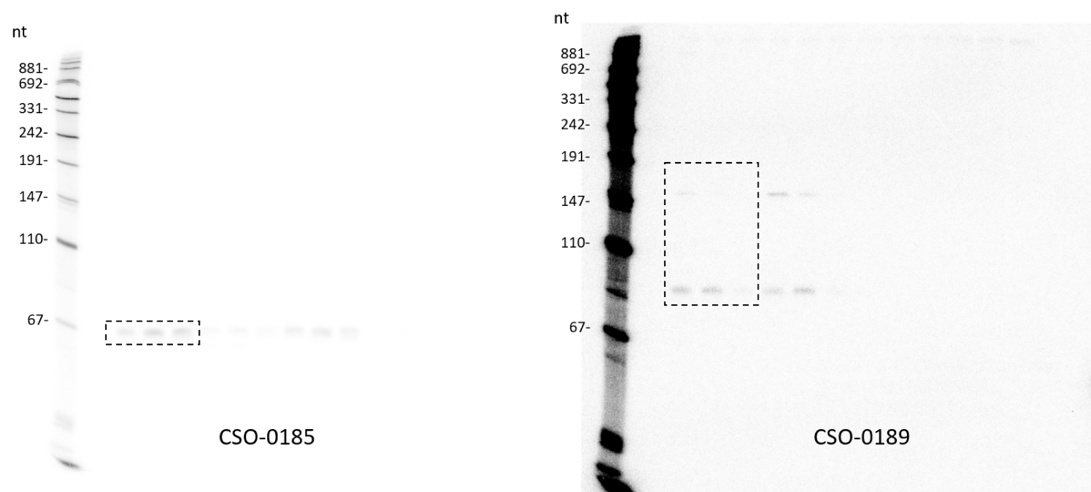


**
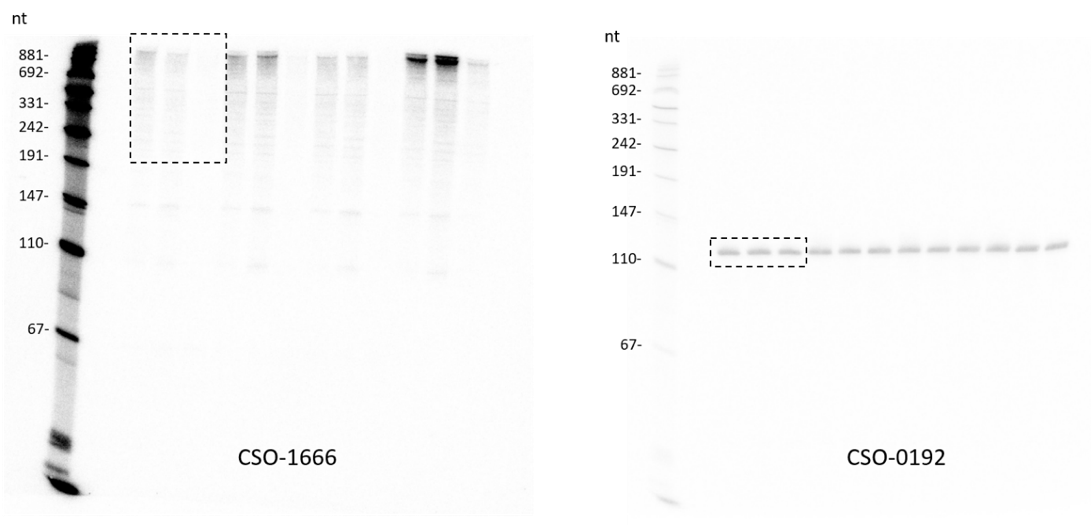
**

|  |  | **CJnc190 (mature)** |
| --- | --- | --- |
|  |  | **CSO-0185** |
|  | OD600 | **Intensity-Bkg [%]** |
| WT | 0.25 | 22.30199979 |
|  | 0.5 | 40.37609388 |
|  | 0.8 | 37.32190633 |

**Panel B**

NB216

**
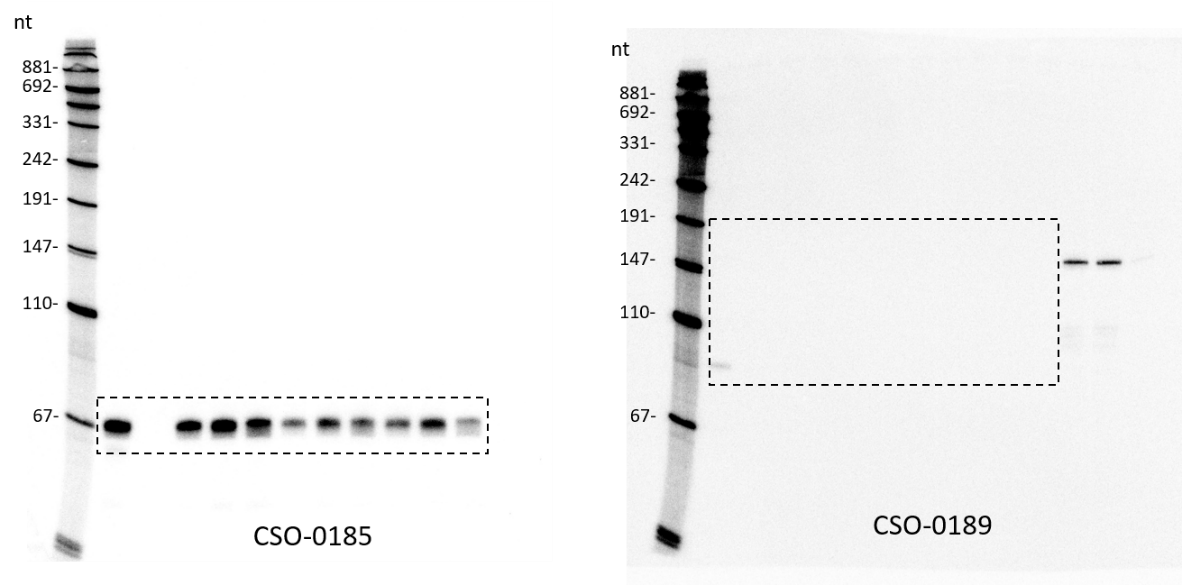
**

**
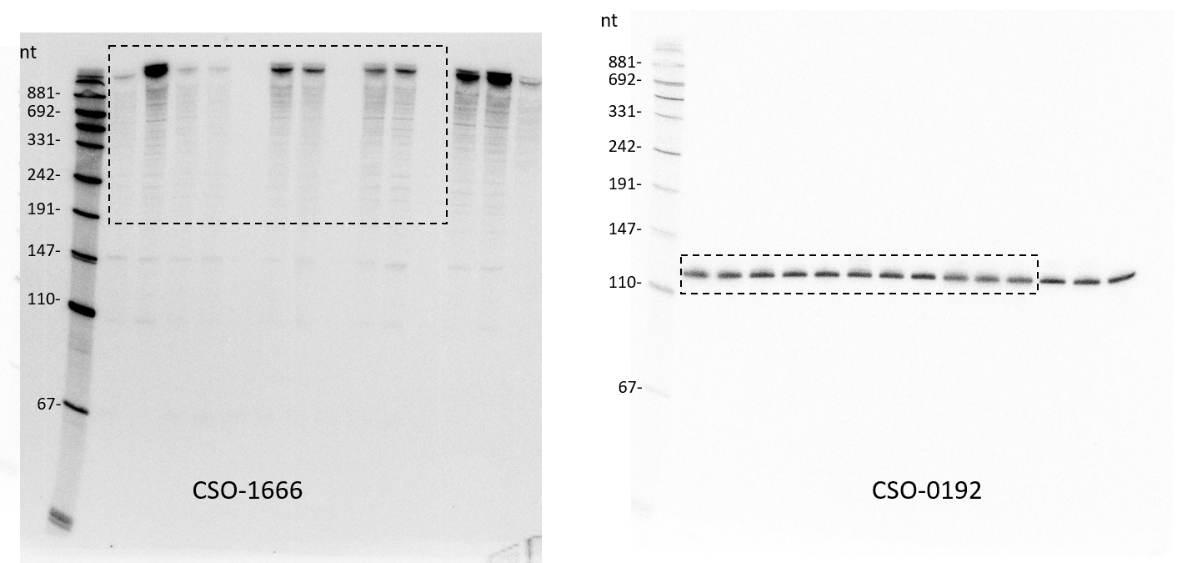
**

Northern blot quantification raw values

|  |  | **CJnc190 (mature)** |
| --- | --- | --- |
|  |  | **CSO-0185** |
|  | OD600 | **Intensity-Bkg [%]** |
| WT | 0.5 | - |
| Δ180/190 | 0.5 | - |
| C-190 only | 0.25 | 12.78526605 |
|  | 0.5 | 17.06359167 |
|  | 0.8 | 12.15486295 |
| C-190 -P1 | 0.25 | 4.806309274 |
|  | 0.5 | 7.801421146 |
|  | 0.8 | 6.577917329 |
| C-190 -P2 | 0.25 | 6.293561059 |
|  | 0.5 | 9.385857218 |
|  | 0.8 | 4.084279932 |

**Panel C**

**
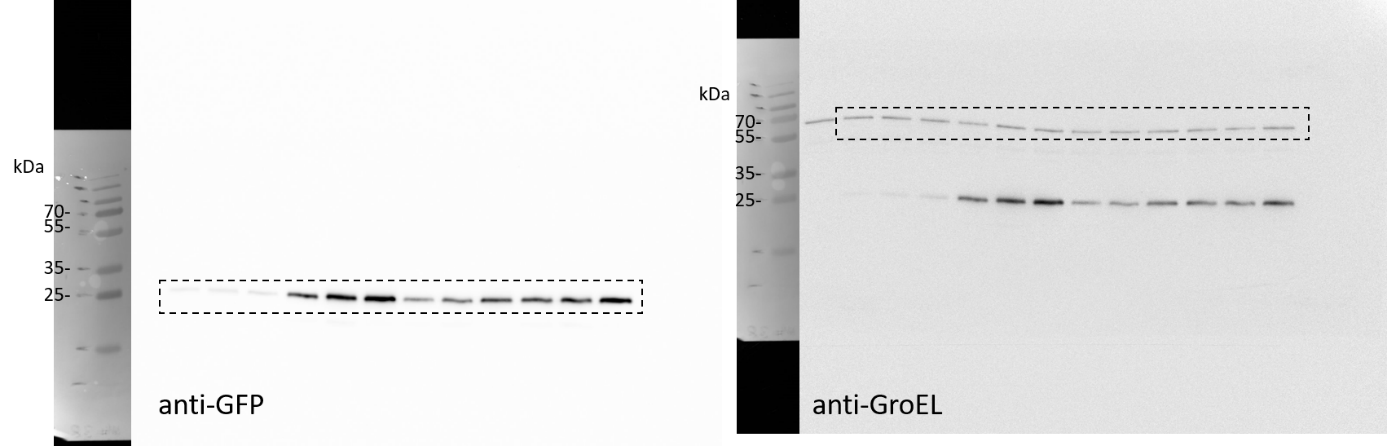
**

**Western blot quantification raw values**

|  |  | **sfGFP** |
| --- | --- | --- |
|  |  | **anti-GFP** |
|  | **OD600** | **Intensity-Bkg [%]** |
| 180P1 | 0.25 | 0.549874111 |
|  | 0.5 | 0.760339761 |
|  | 0.8 | 0.806898595 |
| 190P1/P2 | 0.25 | 8.302963133 |
|  | 0.5 | 14.87623163 |
|  | 0.8 | 18.24465607 |
| 190P1 | 0.25 | 4.668620246 |
|  | 0.5 | 6.559899856 |
|  | 0.8 | 9.677469064 |
| 190P2 | 0.25 | 9.358728915 |
|  | 0.5 | 10.64052145 |
|  | 0.8 | 15.55379717 |
